# Signaling Networks Associated with AKT Activation in Non-Small Cell Lung Cancer (NSCLC): New Insights on the Role of Phosphatydil-Inositol-3 kinase

**DOI:** 10.1371/journal.pone.0030427

**Published:** 2012-02-17

**Authors:** Marianna Scrima, Carmela De Marco, Fernanda Fabiani, Renato Franco, Giuseppe Pirozzi, Gaetano Rocco, Maria Ravo, Alessandro Weisz, Pietro Zoppoli, Michele Ceccarelli, Gerardo Botti, Donatella Malanga, Giuseppe Viglietto

**Affiliations:** 1 Biogem scarl, Institute for Genetic Research “Gaetano Salvatore”, Ariano Irpino (Avellino), Italy; 2 Department of Experimental and Clinical Medicine, University Magna Graecia Catanzaro, Italy; 3 Fondazione “G Pascale”, National Cancer Institute, Naples, Italy; 4 Molecular Medicine Laboratory, Faculty of Medicine and Surgery, University of Salerno, Baronissi, Italy; 5 Department of Biological and Environmental Studies, University of Sannio, Benevento, Italy; Consiglio Nazionale delle Ricerche (CNR), Italy

## Abstract

Aberrant activation of PI3K/AKT signalling represents one of the most common molecular alterations in lung cancer, though the relative contribution of the single components of the cascade to the NSCLC development is still poorly defined. In this manuscript we have investigated the relationship between expression and genetic alterations of the components of the PI3K/AKT pathway [KRAS, the catalytic subunit of PI3K (p110α), PTEN, AKT1 and AKT2] and the activation of AKT in 107 surgically resected NSCLCs and have analyzed the existing relationships with clinico-pathologic features. Expression analysis was performed by immunohistochemistry on Tissue Micro Arrays (TMA); mutation analysis was performed by DNA sequencing; copy number variation was determined by FISH. We report that activation of PI3K/AKT pathway in Italian NSCLC patients is associated with high grade (G3–G4 compared with G1–G2; n = 83; p<0.05) and more advanced disease (TNM stage III vs. stages I and II; n = 26; p<0.05). In addition, we found that PTEN loss (41/104, 39%) and the overexpression of p110α (27/92, 29%) represent the most frequent aberration observed in NSCLCs. Less frequent molecular lesions comprised the overexpression of AKT2 (18/83, 22%) or AKT1 (17/96, 18%), and KRAS mutation (7/63, 11%). Our results indicate that, among all genes, only p110α overexpression was significantly associated to AKT activation in NSCLCs (p = 0.02). Manipulation of p110α expression in lung cancer cells carrying an active PI3K allele (NCI-H460) efficiently reduced proliferation of NSCLC cells *in vitro* and tumour growth *in vivo*. Finally, RNA profiling of lung epithelial cells (BEAS-2B) expressing a mutant allele of PIK3 (E545K) identified a network of transcription factors such as MYC, FOS and HMGA1, not previously recognised to be associated with aberrant PI3K signalling in lung cancer.

## Introduction

Lung cancer is the leading cause of cancer deaths worldwide [1], [2]. Epithelial lung cancer is classified into two main groups: small-cell lung cancer (SCLC) (about 15% of all lung cancers) and non–small-cell lung cancer (NSCLC) (about 85% of all lung cancers) [3]. NSCLC comprises squamous-cell carcinoma (SCC), adenocarcinoma (ADC), and large-cell lung cancer (LCC) [3]. Despite advances in early detection and standard treatment, NSCLC is often diagnosed at an advanced stage and patients often have poor prognosis, with five-year survival rate less than 15% [4], [5]. For this reason a better understanding of the molecular origins of the disease will contribute to improve therapeutic treatment of lung cancer patients.

Recent studies have shown that the phosphatidylinositol 3-kinase (PI3K) signalling cascade is frequently overactivated in human cancer [6]–[8] playing a critical role both in the initiation and progression of NSCLC [9], [10]. The PI3K pathway regulates cellular functions such as proliferation, survival, motility and angiogenesis that are critical to the growth and/or maintenance of tumours [11], [12]. The end-point of the PI3K pathway is AKT, a serine/threonine protein kinase that mediates most signals funnelled through the PI3K pathway. AKT is activated by recruitment to cell membrane via binding of its PH domain to 3′-phosphorylated phosphatidylinositols generated by PI3K and subsequent phosphorylation at T308 and S473 [12], [13]. Conversely, the lipid phosphatase PTEN attenuates AKT activation by dephosphorylating the 3′ position of phosphatidylinositols [14].

Aberrant AKT activation contributes to lung carcinogenesis [9], [10]. Hyperactivation of AKT is detected in most NSCLC cell lines [15]–[17], and in 30–75% NSCLCs [18]–[22] and promotes resistance to chemotherapy and radiation therapy [16]. AKT activation in cancer is currently evaluated using phospho-specific antibodies against S473 in immunohistochemical analyses of tumour specimens. Although phosphorylation of AKT at S473 has been correlated with poor clinical outcomes in many tumour types, results in lung cancer are apparently inconsistent [7]–[10] having been associated with either poor or good prognosis [20]–[22]. AKT can be activated through several mechanisms, which result from distinct and often mutually exclusive events that include activating mutations (KRAS, PIK3CA or AKT1), increased expression (PIK3CA, AKT1, AKT2) or loss of PTEN [10]. However, the relative contribution of the single components within the PI3K pathway to AKT activation in NSCLCs is still unclear. In this manuscript we have investigated the relationship between the genetic alterations present in these genes and the activation of AKT in NSCLC.

## Materials and Methods

### Ethics Statement

Patient accrual was conducted according to internal Review Board of the INT Fondazione Pascale (Naples, Italy) (CEI 556/10 of 12/3/2010). The study was approved by the internal Review Board of the AOU Mater Domini/University Magna Graecia (Catanzaro, Italy) in the meeting of 16/3/2011. Written informed consent was obtained from all participants to the study. All animal work was conducted according to the relevant Italian guidelines and was approved by the Internal Committee for Animal Study (CESA) of the Institute for Genetic Research “Gaetano Salvatore on April 7^th^ 2008 (CESA 10-08).

### Patients

Archive material from 107 patients diagnosed of NSCLC [3] was obtained from INT Fondazione Pascale (Naples, Italy). Median age was 64 year old (range 28–82). Among patients with clinical data available, women were 18 and males 83. Stage was known for 81 patients: 67 patients had stage I–II disease and 14 had stage III–IV disease. Grade was known for 83 patients: 35 cases were G1–G2 and 48 were G3–G4. See Table S1, Table S2 and Table S3 for more detailed clinical characteristics of all patients.

TMA slides were deparaffinized, heated in a pressure cooker with 1 mM EDTA, pH 8.0 for 10 min, and incubated with pepsin at 37°C for 30 min. The slides were then dehydrated in increasing ethanol concentrations, and then air-dried. The probes were denatured at 96°C for 5 min, and hybridization solution was applied on each slide and incubated at 75°C for 1 min. After overnight incubation at 37°C in a humid chamber, slides were washed with 0.4× SSC and 0.3% NP40 for 2 min at 75°C, air-dried in darkness, counterstained with DAPI, and a coverslip was applied.

### Tissue Microarray (TMA) and Immunohistochemistry

TMAs were constructed in collaboration with the Unit of Immunostaining at the Centro Nacional de Investigaciones Oncologicas (Madrid, Spain) according to established methods [23] using a Tissue Arrayer (Beecher Instruments, Gene Micro-Array Technologies, Silver Spring, MD). Immunostaining was performed using the avidin-biotin-peroxidase method (LSAB kit; DAKO, Glostrup, Denmark) as described previously [24]. Antibodies used for immunostaining were selected according to previously published work [25]–[29]. Anti-pS473 (#9277), anti-AKT1 (#2938), anti-AKT2 (#4057), anti-PIK3CA (#4249), anti- PTEN (#9559) were all from Cell Signaling Technology (Danvers, MA, USA).

The anti-Akt1 and anti-Akt2 have been shown to be isoform-specific antibodies in previous work [25]. In addition, by using NCI-H460 cells interfered for Akt1 or Akt2, respectively, we confirmed that the anti-Akt1 antibody recognizes only the Akt1 isoform and the anti-Akt2 antibody recognizes only the Akt2 isoform (Figure S1A).

The immunohistochemical score of pAKT and PTEN used in this work was selected on the basis of the widely established criteria existing in the literature [28], [30], [31]: pAKT was scored as positive when >10% of tumour cells were positive with strong or diffuse immunopositivity. PTEN expression was classified as (+) when staining was detected in >50% of the cells, (+/−) when staining was detected in 25–50% of cells and (−) when staining was detected in 0–25% of cells. For statistical analysis PTEN expression was considered lost when samples were classified as (−).

Also for the immunostaining scores of AKT1, AKT2 and PIK3CA, we selected criteria described in previous reports [27], [28], [32]. Tumor specimens were divided into four groups according to the percentage of positive cells: (−) comprised completely negative samples; (+) comprised samples with up to 10% of positive cells; (++) comprised samples with 11–50% of positive cells; and (+++) comprised samples with >50% of positive cells, respectively. For statistical reasons, tumours were classified into a low expression group comprising (−) and (+) and a high expression group that comprises (++) and (+++).

For each one immunohistochemical round a negative control has been included, by replacing the primary antibody with solvent at the same volume of that with the primary antibody resuspended in it. All controls gave satisfactory results. Stained TMA sections were evaluated by two expert pathologists (RF, GB) using uniform criteria. Discrepancies were resolved through simultaneous inspection and discussion of the results. Discrepancies between two cores from the same case were resolved through a joint analysis of the two cores.

### Fluorescence In Situ Hybridization (FISH)

FISH analysis was performed on TMAs. BAC clones were designed according to the Ensembl database (www.ensembl.org). BAC clones covering the AKT1 gene were RP11-982M15, RP11-477I4 and RP11-556J09. Control BAC probes covering chromosome region 14q11 was RP11-324B11. BAC clones covering the AKT2 gene were RP11-36B02, RP11-688J23, RP11-725P04. Control BAC probes covering chromosome region 19p13.1 were RP11-737I1, RP11-520G3. BAC clones covering the PIK3CA gene were RP11-360P21 and RP11-245C23. Control BAC probes covering chromosome region 3p14.1 were RP11-175F9 and RP11-15B21. All BAC clones were labelled with dUTP-Sprectrum Orange (Vysis Inc., DownersGrove, IL; USA). All Control probes were labelled with dUTP-Sprectrum Green (Vysis Inc., DownersGrove, IL; USA).

Two different investigators that had no previous knowledge of the genetic, clinical and IHC results evaluated FISH analysis. All FISH were scored in an average of 130 (60–210) nuclei.

For evaluation of copy number of the genes encoding AKT1, AKT2 and PIK3CA, a gene-to-control ratio of 1.0 was classified as disomy; ratios between 1.0 and 2.0 were considered gene low-level gains; ratios >2.0 were considered as high polysomy and/or gene amplification [33], [34].

Accordingly, tumours were divided into different classes: disomy, trisomy (3 copies of chromosomes in >40% of cells), low polysomy (≥3 copies of chromosomes in >40% of cells), high polysomy (≥4 copies of chromosomes in ≥40% of cells), and gene amplification (presence of gene clusters with a ratio of gene-to-chromosome of ≥2 per cell in ≥40% of cells or presence of small or nonenumerable clusters of the gene signal). This allowed the classification of patients into two groups: FISH-negative (disomy and gains) and FISH-positive (high polysomy and/or gene amplification).

### PCR, RT-PCR and *mutation analysis*

Total RNA and genomic DNA were prepared as described [35], [36]. Q-RT-PCR and Q-PCR were performed using the Power SYBR Green PCR Master Mix in an ABI Prism 7300 thermocycler (Applied Biosystems, Foster City, CA, USA). cDNAs were synthesized from 1 µg of total RNA using QuantiTect Reverse Trascription (Qiagen, The Netherlands, Venlo). Normalization was performed to GAPDH mRNA content. The relative amounts of mRNA or DNA were calculated by the comparative cycle threshold (CT) method by Livak and Schmittgen [37]. Mutation analysis for PIK3CA using LightCycler was performed with DNA Master/Hybridization probes kit (Roche Molecular Biochemicals, Mannheim, Germany). Direct sequencing was performed using the BigDye v3.03 cycle sequencing kit (Applied Biosystems) in a capillary automatic sequencer (ABI PRISM 3100 Genetic Analyzer; Applied Biosystems). Protocols and primers for Q-PCR, Q-RT-PCR and sequencing KRAS (exons 2 and 3) and PIK3CA (exons 9 and 20) are reported in Appendix S1.

### Antibodies and Western Blot

Western blot analysis was carried out by standard methods [38]. Whole cell extracts were prepared by homogenizing cells in NP-40 lysis buffer (10 mM Tris–HCl (pH 7.5), 150 mM NaCl, 1% NP-40) containing protease inhibitors. Lysates were cleared by centrifugation and proteins were separated by SDS-PAGE. Antibodies used were from Cell Signaling Technology: anti-AKT1 (#2938); anti-S473 (#9277), anti-PIK3CA (#4249).

### Cell lines

NCI-H460 was purchased from ATCC-LGC Promochem (South West London, UK) and maintained in RPMI1640 (Gibco-Invitrogen, Carlsbad, CA, USA), supplemented with 10% of fetal bovine serum and 100 U/ml penicillin-streptomycin (Invitrogen, Carlsbad, CA, USA). BEAS-2B cells were purchased from Cambrex (Milan, Italy) and grown as suggested by the manufacturer [39].

### Virus generation and Infection

To generate p110α encoding lentivirus, the cDNA encoding human p110α (Addgene, Cambridge, MA, USA) was cloned in pENTR1A vector (Invitrogen) and recombined in pLenti6.2/C-Lumio™/V5-DEST Vector by making use of the Gateway Technology (Invitrogen). pLenti vector was used to generate lentiviral particles in HEK293T packaging cells as described [40]. Transduced BEAS-2B cells underwent three rounds of infection and were selected in medium containing 5 µg/ml blasticidin (Invitrogen). The Human PIK3CA (NM_006218), AKT1 (NM_005163) and AKT2 (NM_001626) MISSION shRNA set (Sigma-Aldrich, St.Luis, MO) and the Mission non-target control transduction viruses (SHC002V) were used to generate lentiviral particles in HEK293T packaging cells [40]. After transfection, supernatants were collected at 8-hour intervals, filtered and used for three rounds of transduction of NCI-H460 cells in the presence of 8 µg/ml of polybrene (Sigma).

### In Vitro Proliferation Assay

Cells proliferation was assayed by MTT [3-(4,5-dimethylthiazol-2-yl)-2,5-diphenyltetrazolium bromide; Sigma] reduction. Cells were plated in 96-well flat-bottomed microtiter plates (200 µl cell suspensions, 2×10^3^/well for NCI-H460) and incubated with MTT substrate (5 mg/ml) for 4 h. Every 24 hours, the culture medium was removed and anhydrous 2-propanol was added. The optical density was measured at 570 nm.

### Tumourigenic assays

Cells (1×10^6^) were suspended in 100 ml 10% FBS and 100 ml Matrigel (BD Biosciences, NJ, USA) and subcutaneously injected into the right flank of 6-week-old athymic nude mice (Charles River, West Germany) in triplicates. Every 7 days tumour size was measured with a caliper.

### RNA profiling analysis

RNA concentration was determined with a Nanodrop (NanoDrop, Wilmington, Delaware, USA) spectrophotometer and its quality was assessed with an Agilent 2100 Bioanalyzer (Agilent Technologies, Milano, Italy). For each sample, 500 ng of total RNA were synthesized to biotinylated cRNA using the Illumina RNA Amplification Kit (Ambion, Inc., Austin, TX). Synthesis was carried out according to the manufacturers' instructions. cRNA concentration and the quality were assessed out as described above. From each sample, technical replicates were produced and 750 ng cRNA were hybridized for 18 hrs to Human HT-12_V3_0_R1 Expression BeadChips (Illumina Inc., San Diego, CA, USA) according to the protocol provided by the manufacturer. Hybridized chips were washed and stained with streptavidin-conjugated Cy3 (GE Healthcare Milano, Italy). BeadChips were dried and scanned with an Illumina BeadArray Reader (Illumina Inc.).

### Microarrays data analysis: RNA profiling, genes' characterization, enriched pathways and bibliographic networks discovery

Expression files were normalized and analyzed using GeneSpring 10.1 (Agilent Technologies, Santa Clara, CA). Differentially expressed (DEGs) genes between BEAS-2B and BEAS-PI3K-CA cells were selected on the basis of the fold change (the ratio between the expression levels in the two conditions) and the statistical significance. We filtered the lists using fold change 1.5 and T-test (*p*-value (0.01) as threshold. The DEGs list (composed by 2126 probesets) was used to evaluate the functional behavior in terms of Biological Processes and Molecular Function, Development Function and Disease and Disorder terms. The degree of enrichment was statistically evaluated to determine whether an observed level of annotation for a group of genes is significant. In particular, for each term, a *q*-value was computed by the Hypergeometric test (*p*≤0.05) and corrected using False Discovery Rate (FDR) [41]. The terms with a *q*-value exceeding the significance threshold were then selected as representative. Pathway and network analysis were performed using Ingenuity Pathway Analysis (IPA, Ingenuity Systems).

The dataset was mined for significant pathways with the IPA library of canonical pathways, and networks were generated by using IPA as graphical representation of the molecular relationships between genes and gene products. The significance of the association between the list of DEGs and the Canonical Pathway was measured using a Fisher's exact test to calculate a *p*-value (*p*≤0.05). Fisher's exact test results were also corrected for multiple testing using FDR.

In networks, genes or gene products are represented as nodes, and the biological relationship between two nodes is represented as an edge (line). All edges are supported by at least one reference from the literature, from a textbook, or from canonical information stored in the IPA Knowledge Base. Human, mouse, and rat orthologs of a gene are stored as separate objects, but are represented as a single node in the network. The network building's algorithm determines a statistical score for each network. This is done by comparing the number of focus genes that contribute to a given network relative to the total number of occurrences of those genes in all networks or pathways stored in the IPA Knowledge Base. The intensity of genes (node) colour in the networks indicates the degree of downregulation (green) or upregulation (red) of gene expression. Nodes are displayed using various shapes that represent the functional class of gene products.

## Results

### AKT activation in NSCLCs

**Figure 1:**
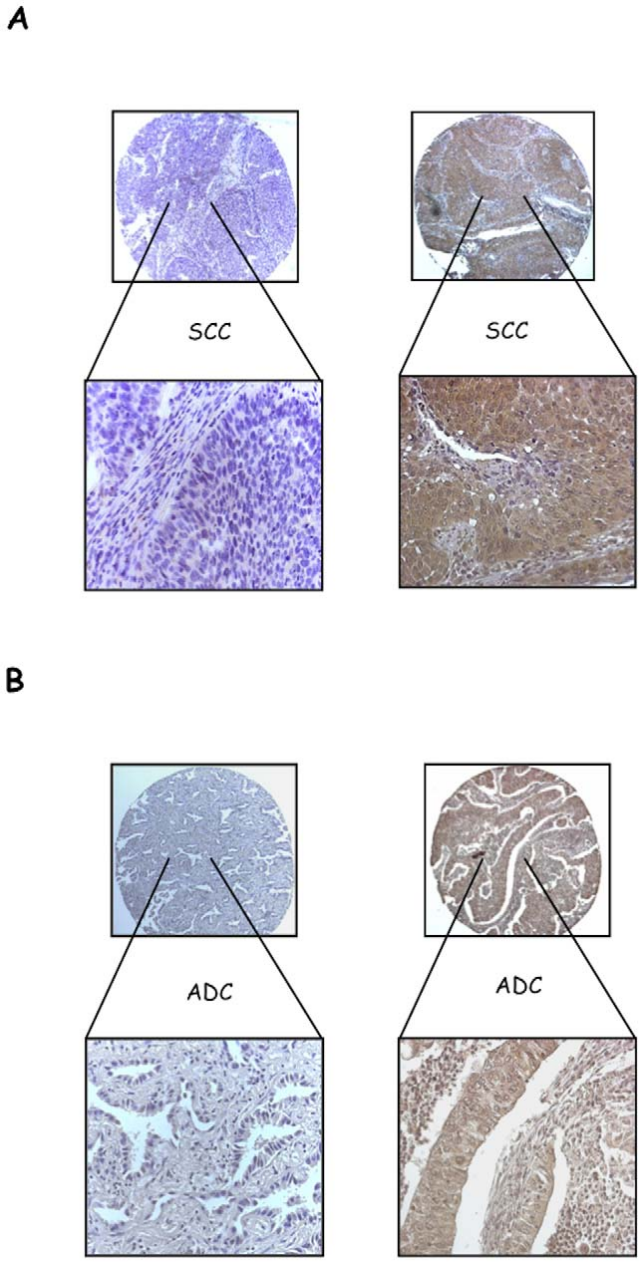
pS473 AKT immunostaining (IHC) in NSCLCs. A, left: SCC negative for pAKT phosphorylation; right: SCC positive for pS473 phosphorylation. B, left: ADC negative for pAKT phosphorylation; right: ADC positive for pS473 phosphorylation. Magnification 10× and 40×, respectively.

As a read-out of PI3K/AKT signalling in NSCLC we determined the phosphorylation status of residue S473 of AKT1 (pAKT). pAKT was evaluated on TMAs containing duplicated core biopsies of 107 NSCLCs. As controls 45 matched normal samples were used. Patients' clinico-pathological characteristics are described in Materials and Methods and summarized in Tables S1, S2 and S3. The results obtained from pAKT staining in NSCLC are summarized in Table 1. pAKT staining was barely detectable in the epithelial cells from normal alveolar epithelium and from upper airways (39 out of 45 samples) (See Figure S1). In contrast, AKT activation was observed in 60 out of 97 of NSCLC analysed (Table 1). Positive pAKT staining was significantly higher in the carcinoma samples than either normal alveolar or bronchial epithelium (P<0.001; Chi square test). pAKT staining was observed in 23/37 SCCs and 30/44 ADCs (Figure 1A and B, respectively). We found a significant association between pAKT staining and the grade or the stage of the disease (Table 2): pAKT staining was significantly more represented in patients with grades G3–G4 compared with patients with grades G1–G2 (p<0.05) and in patients with TNM stage III compared with patients with stage II disease (p<0.05). See Tables S4 and S5 for distribution of patients into SCCs and ADCs. These results demonstrate that, in agreement with work in other populations, in Italian NSCLC patients AKT activation occurs in tumour tissue and correlates with a more advanced stage of disease [20]–[22]. See also Table S9 and S10 for a detailed, patient-by-patient, list of pAKT positivity.

**Table 1:** AKT activation in NSCLCs.

	*AKT activation (pS473)* a		
HISTOLOGY	SAMPLE NUMBER	LOW	HIGH
NORMAL	45	39	6
ADC	44	14	30
SCC	37	14	23
ASQ	7	4	3
LCC	6	3	3
CAR	3	2	1
Total	97	37	60

a AKT activation was evaluated with phospho-specific antibodies (pS473) and scored as negative (<10% of the tumour cells with weak, focal immunopositivity or absence of staining) and high (>10% of tumour cells with strong or diffuse immunopositivity).

ADC (adenocarcinoma), SCC (squamous cell carcinoma), ASQ (adenosquamous carcinoma), LCC (large cell carcinoma) CAR (carcinoid tumour).

**Table 2:** Correlation between AKT activation and clinico-pathological features of NSCLC patients.

	Akt activation (pS473)^a^		
	Low (n)	High (n)	P value
Gender			
Male	27	49	
Female	9	9	
Grade			
G1–G2	19	16	0.035§
G3–G4	15	33	
TNM stage			
Stages I	20	35	0.049*
Stage II	6	6	
Stage III	2	12	

a AKT activation was evaluated with phospho-specific antibodies (pS473) and scored as negative (<10% of the tumour cells with weak, focal immunopositivity or absence of staining) and high (>10% of tumour cells with strong or diffuse immunopositivity).

§ G1–G2 vs G3–G4.

*Stage II vs Stage III.

### Mechanisms of AKT activation in NSCLCs: immunohistochemistry

To investigate the molecular mechanisms leading to AKT activation in Italian patients affected by NSCLC we performed a comprehensive analysis of the expression and/or the genetic status of AKT1 and AKT2 and their closest regulators (KRAS, PIK3CA and PTEN). Of the 107 cases present on the TMAs 96 could be properly analysed for AKT1, 83 for AKT2, 104 for PTEN and 92 for PIK3CA.

**Figure 2:**
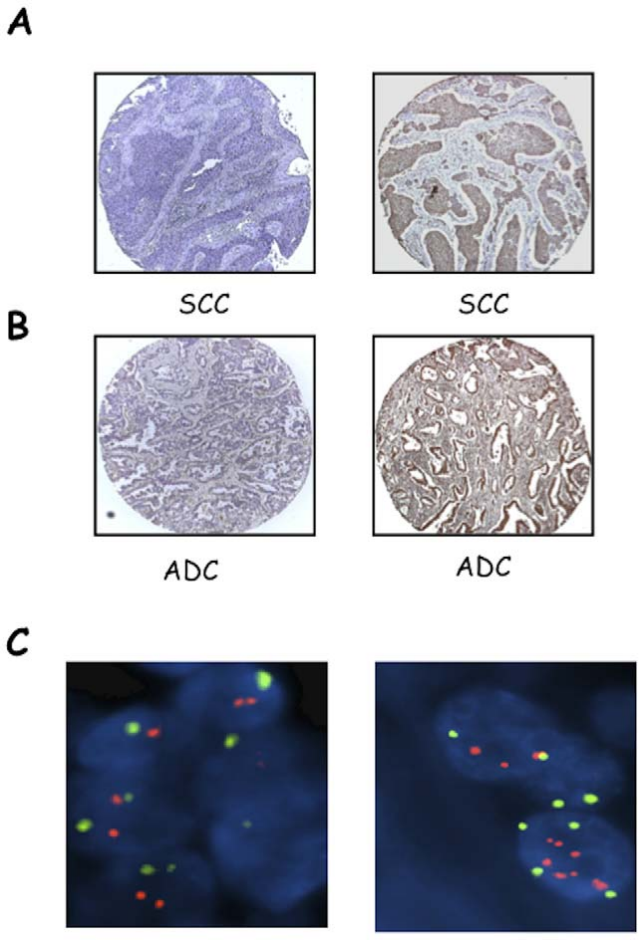
IHC and FISH analysis of AKT1 in NSCLCs. A, left: SCC negative for AKT1 expression; right: SCC positive for AKT1 expression. B, left: ADC negative for AKT1 expression; right: ADC positive for AKT1 expression. Magnification 10× and 40×, respectively. C. Dual-colour fluorescence in situ hybridization analysis of AKT1 gene copy number. FISH analysis of AKT1 (red signals) and centromere of chromosome 14 (green signals). Left, NSCLC sample with diploid cells; right, NSCLC sample with multiple clustered spots of red signals of AKT1 with 2 centromere signals (gene amplification). Original magnification 100×.

See Materials and Methods for the evaluation criteria used for AKT1. Briefly, samples defined (−) were completely negative for AKT1; samples defined (+) contained up to 10% of positive cells; samples defined (++) comprised 11–50% of positive cells; samples defined (+++) comprised >50% of positive cells, respectively. Figure S2 shows representative stainings of (+), (++) or (+++) AKT1 expression in SCCs and ADCs. Tumours were classified into a low expression group comprising (−) and (+) and a high expression group that comprises (++) and (+++). Analysis of TMAs 258.1 and 258.2 showed that AKT1 was over-expressed in 17/96 NSCLC cases (∼19%) (Figure 2), with SCCs and ADCs showing similar results: 7/37 AKT1 positive tumors were SCCs (19%) and 7/44 AKT1 positive tumours were ADCs (16%). See Figure 2A and B, respectively. Nine out of 15 (60%) NSCLCs overexpressing AKT1 showed AKT activation (Table 3).

**Table 3:** Correlation between AKT activation and expression of the different members of the PI3K pathway in NSCLCs.

		*pAKT negative* a	*pAKT positive* a	*Total number*	*P value*
PIK3CA^b^	negative	12	5	17	0.02§
	moderate	13	24	37	
	high	8	18	26	
PTEN^c^	positive	9	12	21	
	reduced	16	22	38	
	negative	13	23	36	
AKT1^d^	negative	21	23	45	
	moderate	8	27	35	
	high	6	9	15	
AKT2^e^	negative	20	19	39	
	moderate	5	17	22	
	high	5	12	17	

a AKT activation was evaluated with as pS473 positivity and scored as negative (<10% of positive tumour cells) and high (>10% of positive tumour cells).

b PIK3CA was graded as positive (>25% of tumour cells showed strong or diffuse immunopositivity) as moderate (>10% of tumour cells showed moderate immunopositivity) or negative (0–10% of the tumour cells showed weak, focal immunopositivity or absence of staining).

c PTEN expression was classified as (+) when staining was detected in >50% of the cells, (+/−) when staining was detected in 25–50% of cells and (−) when staining was detected in 0–25% of cells. For statistical analysis PTEN expression was considered lost when samples were classified as (−).

d AKT1 was graded as positive (>25% of positive tumour cells) as moderate (>10% of of positive tumour cells) or negative (0–10% of positive tumour cells).

e AKT2 was graded as positive (>25% of positive tumour cells) as moderate (>10% of positive tumour cells) or negative (0–10% of positive tumour cells).

§ Statistically significant.

**Figure 3:**
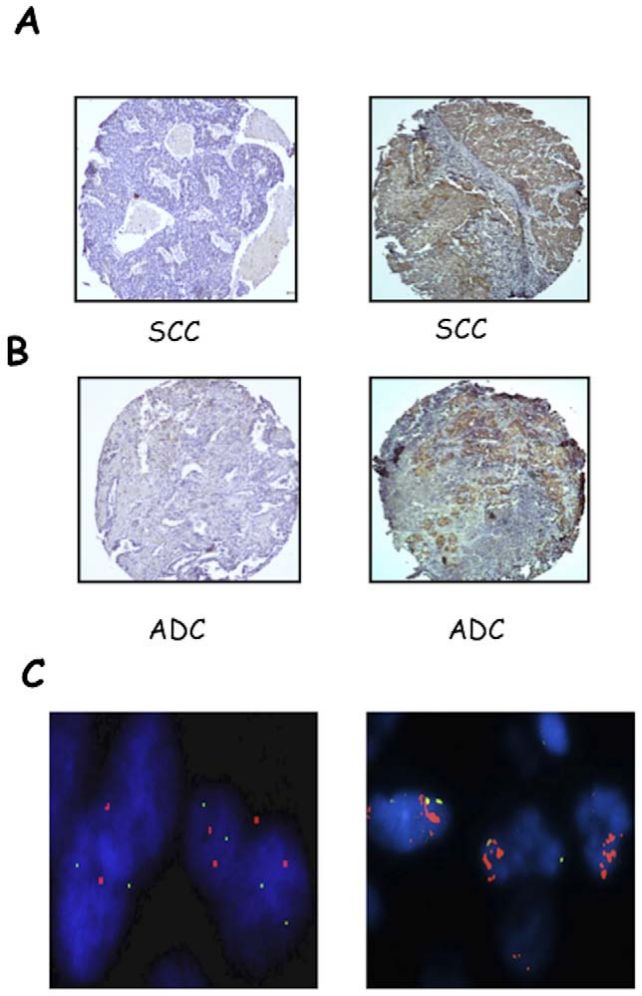
IHC and FISH analysis of AKT2 in NSCLCs. A, left: SCC negative for AKT2 expression; right: SCC positive for AKT2 expression. B, left: ADC negative for AKT2 expression; right: ADC positive for AKT2 expression. Magnification 10× and 40×, respectively. C. Dual-colour fluorescence in situ hybridization analysis of AKT2 gene copy number. FISH analysis of AKT2 (red signals) and chromosome region 19p13.1 (green signals). Left, NSCLC sample with diploid cells; right, NSCLC sample with multiple clustered spots of red signals of AKT2 with 2 chromosome region 19p13.1 signals (gene amplification). Original magnification 100×.

We then analysed the expression of AKT2 in NSCLCs. See Materials and Methods for the evaluation of AKT2 staining. Samples defined (−) were completely negative for AKT2; samples defined (+) were with up to 10% of positive cells; samples defined (++) comprised 11–50% of positive cells; and samples defined (+++) comprised >50% of positive cells, respectively. Figure S3 shows representative stainings of (+), (++) or (+++) AKT2 expression in SCCs and ADCs. Tumours were classified into a low expression group comprising (−) and (+) and a high expression group that comprises (++) and (+++). AKT2 was overexpressed in 18/83 NSCLCs (∼22%) (Figure 3). At difference with AKT1, AKT2 overexpression was observed more frequently in SCCs (10/31 SCCs, 32%; 4/33 ADCs, 12%). See Figure 3A and B, respectively. In addition, most AKT2 positive tumours (12/17, 71%) showed AKT activation (Table 3).

Patients accrued for this study had already been characterised for PTEN expression [38]: complete loss occurred in 41 of 104 (39%) NSCLCs and partial down-regulation was observed in 41 additional cases. PTEN loss was more frequently observed in SCCs (22/40, 55%) than in ADCs (14/51, 27%) (See Figure S5). However, when correlated with AKT activation, the loss or the reduction of the levels of PTEN protein was not associated with AKT activation (n = 95; p = 0.832) (Table 3).

**Figure 4:**
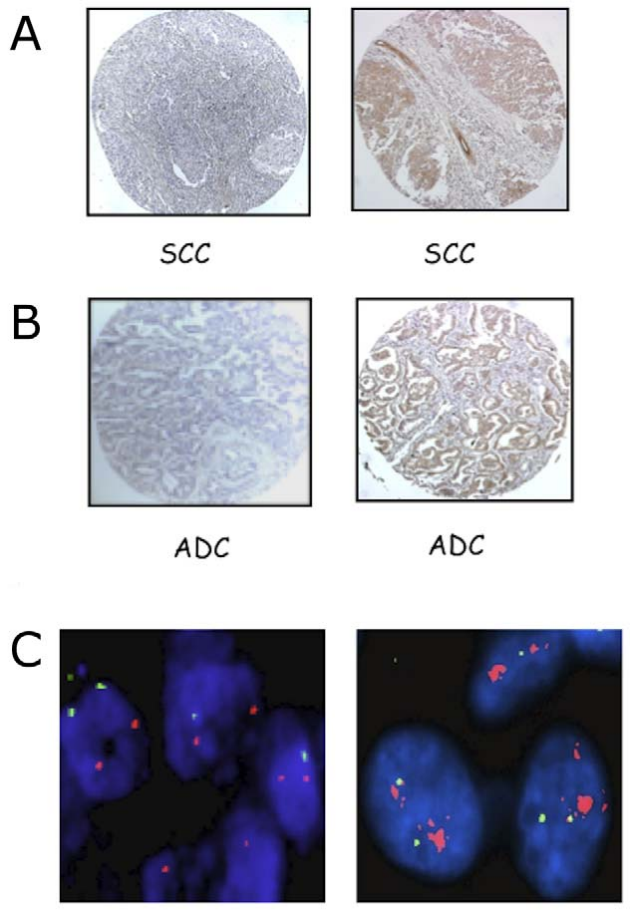
IHC and FISH analysis of PI3KCA in NSCLCs. A, left: SCC negative for PIK3CA expression; right: SCC positive for PIK3CA expression. B, left: ADC negative for PIK3CA expression; right: ADC positive for PI3KCA expression. Magnification 10× and 40×, respectively. C. Dual-color fluorescence in situ hybridization analysis of PIK3CA gene copy number. FISH analysis of PIK3CA (red signals) and chromosome region 3p14.1 (green signals). Left, NSCLC sample with diploid cells; right, NSCLC sample with multiple clustered spots of red signals of PIK3CA with 2 chromosome region 3p14.1 signals (gene amplification). Original magnification 100×.

Finally, we analysed the expression of the catalytic subunit of PI3K, p110α. Evaluation criteria are reported in Materials and Methods. Samples defined (−) were completely negative for p110α; samples defined (+) contained up to 10% of p110α positive cells; samples defined (++) comprised 11–50% of p110α positive cells; and samples defined (+++) comprised >50% of p110α positive cells. Figure S4 shows representative stainings of (+), (++) or (+++) AKT1 expression in SCCs and ADCs. Tumours were classified into a low expression group comprising (−) and (+) and a high expression group that comprises (++) and (+++). We observed p110α overexpression in ∼29% of NSCLCs (27/92): 12 out of 34 were SCCs (35%) and 12 out of 43 were ADCs (28%) (Figure 4A and 4B). At difference with other genes within the pathway that have been analysed, we found that NSCLCs with overexpressed p110α presented significantly activated AKT (18 out of 26; p = 0.02) (Table 3).

Notably, from the integrated analysis of the TMAs we found that AKT activation was more frequently observed in tumours showing aberrant expression of more than a single gene within the PI3K pathway (PTEN loss, or overexpression of AKT1, AKT2, p110α respectively). In fact, AKT activation was detected in 15–64% of tumours showing aberration in a single gene, 44–89% of tumours with aberrant expression of two genes, 67–100% of tumours with aberrant expression of three genes and 100% of tumours with aberrant expression of all four genes. Conversely, aberrant expression of the members of PI3K pathway was less common in tumours showing no activation of AKT signalling (see Table 4).

**Table 4:** Alteration in the expression of PTEN, PI3K, AKT1 and AKT2 in pAKT positive NSCLCs.

*Alteration*	*pAKT positive*	*pAKT negative*
AKT1^b^	9/59 (15%)	6/35 (17%)
AKT2^c^	12/48 (25%)	5/30 (17%)
PI3K^d^	18/47 (38%)	8/33 (24%)
PTEN^e^	23/36 (64%)	9/38 (24%)
AKT1, PTEN	4/7 (57%)	3/7 (43%)
AKT2, PTEN	8/9 (89%)	1/9 (11%)
PI3K, PTEN	4/9 (44%)	5/9 (56%)
AKT1, AKT2	3/4 (75%)	1/4 (25%)
PI3K, AKT1	3/6 (50%)	3/6 (50%)
PI3K, AKT2	5/8 (62%)	3/8 (37%)
AKT1, AKT2, PTEN	3/3 (100%)	0
AKT1, PI3K, PTEN	2/3 (67%)	1/3 (33%)
AKT2, PI3K, PTEN	3/3 (100%)	0
AKT1, AKT2, PI3K	2/3 (67%)	1/3 (33%)
AKT1, AKT2, PI3K, PTEN	2/2 (100%)	0

b Moderate and high AKT1 expression as defined in Table 3.

c Moderate and high AKT2 expression as defined in Table 3.

d Moderate and high PIK3CA expression as defined in Table 3.

e PTEN loss as defined in Table 3.

### Mechanisms of protein overexpression: FISH analysis

FISH analysis in NSCLCs was performed for AKT1, AKT2 and PIK3CA to determine the molecular mechanisms of the overexpression of the corresponding proteins. See Materials and Methods for classification of tumours by FISH. We found 20/82 NSCLC (24%) with copy number gain of the AKT1 gene at chromosome 14, of which 16 were high polysomy (>4 copies) and 4 focal amplification (SCC-11, SCC-12, SCC-14 and SCC-21) (Figure 2C). Expectedly, several AKT1 FISH-positive NSCLCs (12 out of 20 cases, 60%) showed moderate or high AKT1 expression. See Tables S9 and S10 for a detailed list of genetic alterations detected in single SCC and ADC patients. In the case of AKT2, we observed 24/73 NSCLCs (31%) with copy number gain of the gene at chromosome 19, of which 23 patients had high polysomy and 1 patient had focal amplification (SCC-11). See Figure 3C for a representative example. However, the significance of AKT2 amplification in lung cancer remains unclear, since 13/24 (54%) cases of AKT2 FISH-positive tumours did not show increased expression of the corresponding protein.

FISH analysis with chromosome 3q26.32 probes revealed the presence of an increase in the PIK3CA gene copy number in 19 cases (∼26%), all of which presented high polysomy, with 7 cases showing also focal amplification (ADC-5, SCC-4, SCC-14, SCC-16, SCC-19, SCC-30, SCC-34) (Figure 4C). The majority of NSCLCs with increased copy number of PIK3CA (13 out of 19 cases, 68%) showed moderate or high expression of p110α.

However, not all FISH-positive NSCLCs resulted in the activation of AKT signalling. As shown in Tables S6, S7 and S8, 11/18, 10/19 and 14/23 cases that were FISH-positive for PIK3CA, AKT1 and AKT2 resulted positive for pAKT, respectively.

### Mechanisms of AKT activation: mutation analysis of PIK3CA and KRAS

**Figure 5:**
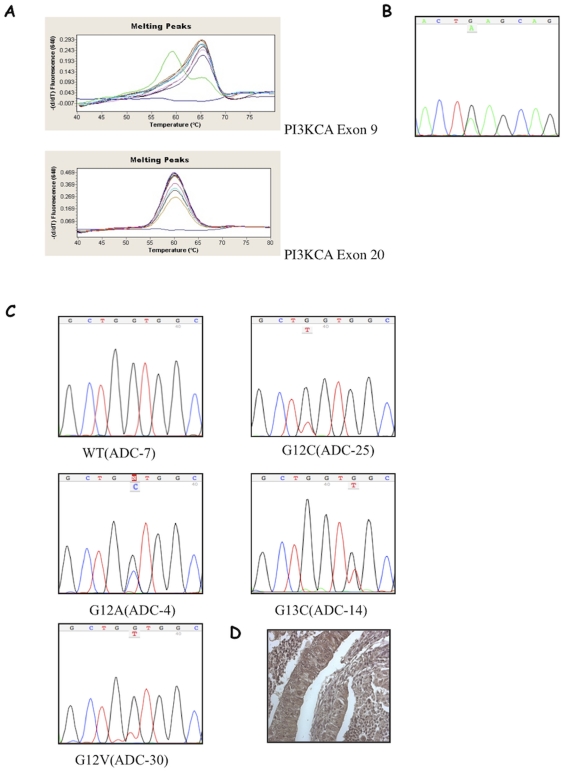
Mutation analysis of PIK3CA and KRAS genes in NSCLCs. **A.** Mutation detection in the exons 9 and 20 of PIK3CA from NSCLC. The negative derivative of the fluorescence (−dF/dT) versus temperature graph shows peaks with different Tm. The wild type sample showed a single Tm at 66°C. The heterozygous mutant sample showed an additional peak at 57°C. **B.** Point mutation in the PI3KCA gene involving a GAG→AAG transition in codon 545 of exon 9 inducing the substitution of a glutammic acid with a lysine (E545K). **C.** Point mutations in the KRAS gene involving a GGT→GCT, GGT→TGT, GGT→GTT; GGC→TGC transition in codon 12 of exon 2 inducing the substitution of a glycine by an alanine, a cysteine and a valine (G12A G12C G12V) transition in codon 13 of exon 2 inducing the substitution of a glycine by a cysteine (G13C). **D.** pAKT staining of sample ADC-30.

Patients accrued for this study had already been analysed for AKT1 mutations [24]. Patient SCC-29 presented a somatic mutation in the gene encoding AKT1 resulting in a glutamic acid to lysine substitution at amino acid 17 (E17K) [24]. The tumour from this patient showed increased AKT expression and activity. Similarly, missense mutations in PIK3CA have been rarely reported [42]–[45]. We found a GAG1633→AAG substitution that leads to the amino acid change E545K in one SCC (SCC-6) (Figure 5A and B). Conversely, 7 NSCLCs showed mutations in KRAS: G12A (GGT→GCT) (n = 1); G12C (GGT→TGT) (n = 4); G12V (GGT→GTT) (n = 1); G13C (GGC→TGC) (n = 1) (Figure 5C). KRAS mutations were detected mainly in ADCs (6 of 32; 19%) as described [46], [47]. Five out of 7 of cases with mutations in KRAS showed significant AKT activation (Figure 5D).

### Activated PI3K contributes to cell proliferation and tumourigenicity of NSCLC cells

**Figure 6:**
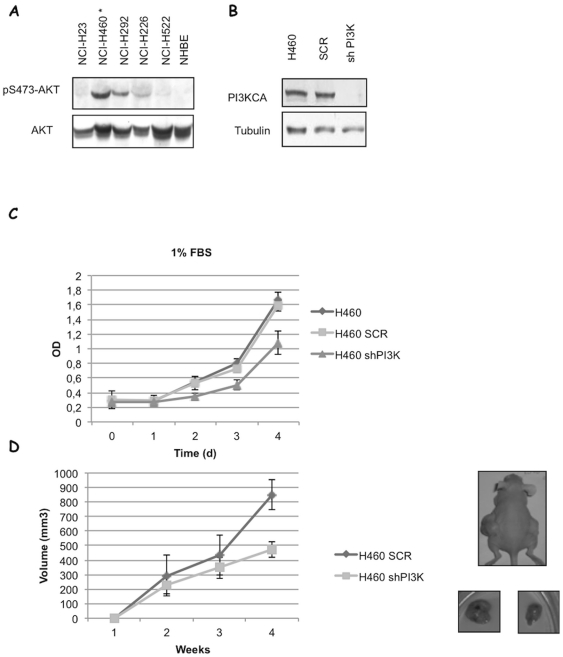
Interference with PIK3CA decreases growth and tumorigenesis of human NSCLC cells carrying activated p110α. **A.** Immunoblot analysis of phosphorylated S473 (pAKT) and total AKT in NSCLC cells. **B.** Immunoblot analysis of PI3KCA expression in parental (NCI-H460), scrambled-transduced (SCR) or PI3KCA-specific shRNA-transduced lentiviruses (shPIK3CA). **C.** NCI-H460 cells transduced with the control lentivirus (SCR) or with lentivirus carrying shRNA to PIK3CA (shPIK3CA) were seeded in flat-bottom 96-well plates and the relative number of viable cells was measured by MTT assay. Absorbance was read at 570 nm and the data are mean of triplicates. **D.** SCR- and shPIK3CA transduced NCI-H460 cells were subcutaneously injected into the flank of athymic nude mice and the growth of xenotransplated tumour was measured as described in Material and Methods.

Given the importance of PI3K signalling in NSCLCs, we investigated the role of constitutive PIK3CA activation on the tumourigenic potential of human lung epithelial cells. To this aim, we made use of a mutant cell line (NCI-H460) that harbours a heterozygous activating mutation (E545K) in PIK3CA. Cells were transduced with a lentivirus expressing shRNA for PIK3CA (Figure 6A). Silencing of p110α expression was assessed by immunoblot (Figure 6B). Here we show that suppression of p110α expression in NCI-H460 cells markedly reduced *in vitro* anchorage-dependent and *in vivo* tumour growth of cells subcutaneously injected into immunodeficient mice (n = 6/group) (Figure 6C and D, respectively), indicating that PI3K activation plays a significant role in the malignant behaviour of NSCLC cells.

### Molecular profiling of PI3K activation in lung epithelial cells

**Figure 7:**
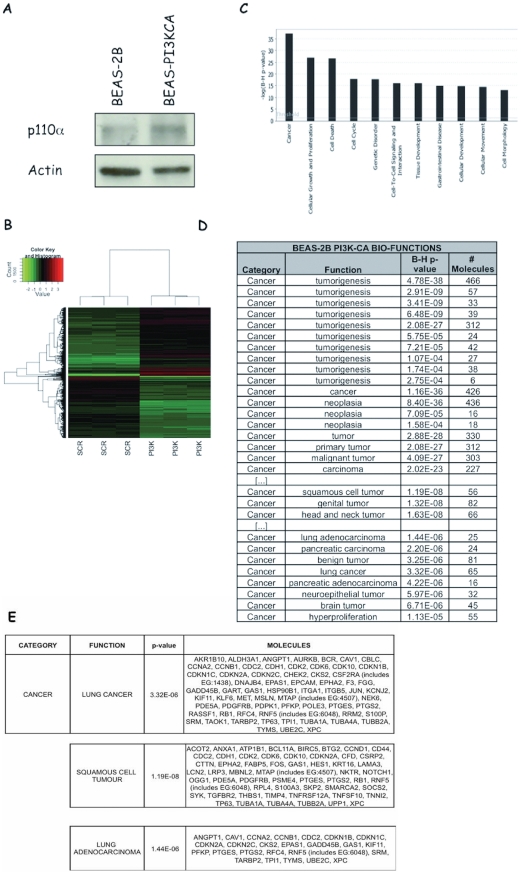
The top 11 canonical signalling pathways influenced by constitutive PI3K signaling. Active PIK3CA (E545K)-expressing lentivirus was transduced in non-transformed lung epithelial cells (BEAS-2B). Transduced cells were selected in blasticydin, checked for expression of the exogenous PIK3CA-E545K, were analysed for their transcriptomes as described in Materials and methods. **A.** Immunoblot for PIK3CA expression in transfected BEAS-2B cells. **B.** Heat map showing fold change patterns of DEGs induced by constitutive PI3K signalling. The heat map was generated in Matlab (Mathworks), and compares fold change patterns of DEGs in BEAS-2B-PI3K-E545K cells compared to parental BEAS-2B (p<0.01). Red: up-regulated genes; green: down-regulated genes. Fold changes of all down-regulated DEGs and all but one up-regulated DEG are #8 (central color spectrum bar). **C.** The top 11 functional categories determined by IPA, that were significantly up-regulated or down-regulated in BEAS-2B-PI3K-E545K cells compared to parental BEAS-2B are shown. The 2126 DEGs in BEAS-2B-PI3K-E545K were mapped to the IPA-defined network. The significance p-values that determine the probability that the association between the genes in the dataset and the canonical pathway is by chance alone were calculated by Fisher's exact test, and are expressed as −log (p-value). **D.** Bio-functions identified by IPA in the 2126 DEGs from BEAS-2B-PI3K-E545K compared with BEAS-2B. **E.** Sub-Categories and Functions identified through IPA showing the genes associated to lung cancer in the 2126 DEGs from BEAS-2B-PI3K-E545K compared with BEAS-2B.

To further characterize the role played by PIK3CA in development of NSCLC, we performed RNA profiling analysis of human lung epithelial cells expressing an active PI3KCA mutant (E545K) to identify cellular targets of constitutive PI3K signalling. Expression of exogenous PI3KCA allele was determined by immunoblot (Fig. 7A). Expression values obtained were filtered for fold change greater than 1.5 and subjected to *t*-test (*p*-value cut-off of 0.01) with Benjamini-Hochberg (B–H) FDR correction [41], obtaining a total of 2126 differentially expressed probe sets, of which 1005 were down-regulated and 1121 were up-regulated. The complete microarray data for all probe sets with the respective normalised values will be available at ArrayExpress and are provided in additional files (Table S11).

We used Ingenuity Pathway Analysis (Ingenuity®Systems, http://www.ingenuity.com, IPA) to investigate the biological relevance of the PI3K-dependent expression changes by categorizing our dataset into biological pathways and/or functions and diseases (Figure 7B, 7C, 7D, 7E; Figure S6; Table S11). The function “Cancer” was most frequent and associated with 466 genes, followed by “Cell Death” (392 genes), “Cellular Growth and Proliferation” (357 genes), “Cellular Movement” (196 genes), “Cell Cycle” (161 genes), “Cell-to-cell Signalling and Interaction” (112 genes), and “Cellular Morphology” (97 genes), respectively. We found that active PI3K regulates expression of most cell cycle molecules such as CCND1, CCND2, Cdk6 and Cdk inhibitors as well as of several apoptosis-related genes such as BAG3, IGFBP7, IGFBP3, TRADD and TRIB1. As to “Cell movement” function, IPA analysis of PI3K-regulated Functions identified growth factors (TGFA), cytokines (IL1A, IL1B, IL6 and IL8) and chemokines (CXCL2) that are involved in stromal-to-epithelial signalling, invasion, angiogenesis and metastasis. It is of note that IPA analysis of DEGs in PIK3CA-transduced BEAS-2B cells retrieved several functions linked to ADCs and SCCs (Figure 7D, 7E), indicating that the adoptive expression of active PIK3CA elicit a transcriptional response that is associated to lung cancer.

### Activated PI3KCA regulates the expression of the oncogenic transcription factors HMGA1, FOS and MYC

IPA analysis of DEGs demonstrated that PI3K activation induced the up-regulation of several oncogenic transcription factors (i.e. MYC, JUN, JUN-B, FOS, HMGA1, HES1), with each transcription factor being the node of networks involving 30–40 down-regulation or up-regulation events (Figures S7A, S7B, S7C, S7D, S7E, S7F, S7G, S7H).

**Figure 8:**
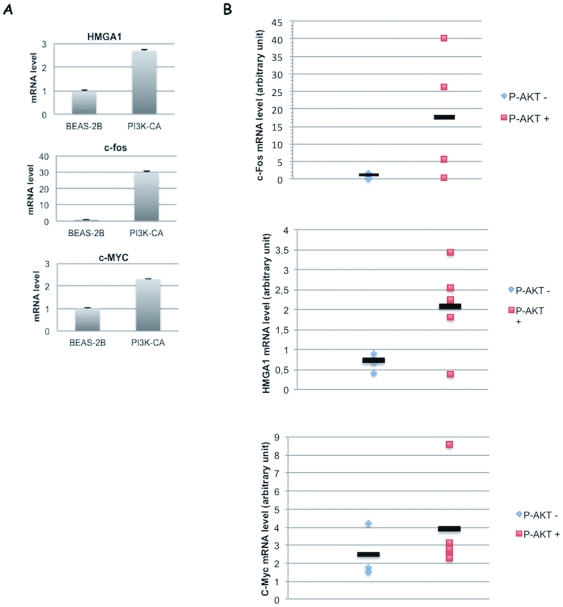
Activation of PI3KCA regulates expression of HMGA1, c-Fos, c-MYC in NSCLC cell lines. **A.** Quantitative RT-PCR analysis of HMGA1, c-Fos, c-MYC gene expression in control BEAS-2B cells and in the corresponding cells transduced with active PIK3CA. **B.** Quantitative real-time RT-PCR analysis of c-Fos, HMGA1, c-Myc gene expression in different NSCLC cell lines.

We confirmed the results obtained from the array analysis by quantitative RT-PCR on a selected panel of genes (HMGA1, FOS, MYC) (Figure 8A). Subsequently, we performed Q-RT-PCR analysis of the mRNA expression of HMGA1, FOS and MYC genes in ADC- or SCC-derived cell lines (n = 8) and correlated them with the AKT activation (S473 phosphorylation) status as a read-out of PI3K activity. The cell lines used were as follows: ADC-derived cell lines, A549, NCI-H522, NCI-H23, NCI-H460, NCI-H596; SCC-derived cell lines, NCI-H226, CALU1, BEN1. Q-RT-PCR analysis demonstrated that NSCLC-derived cell lines with activated AKT (A549, NCI-H460, NCI-H596, NCI-H226, CALU1) presented, on average, increased expression of HMGA1, FOS and MYC compared with cells with low AKT activation (NCI-H522, NCI-H23, BEN1). For HMGA1 the average values were: 0.6 in the case of AKT negative cells and 2 in the case of AKT positive cells; for FOS the average values were: 0.9 in the case of AKT negative cells and 18 in the case of AKT positive cells; for MYC the average values were: 2.5 in the case of AKT negative cells and 3.9 in the case of AKT positive cells (Figure 8B).

**Figure 9:**
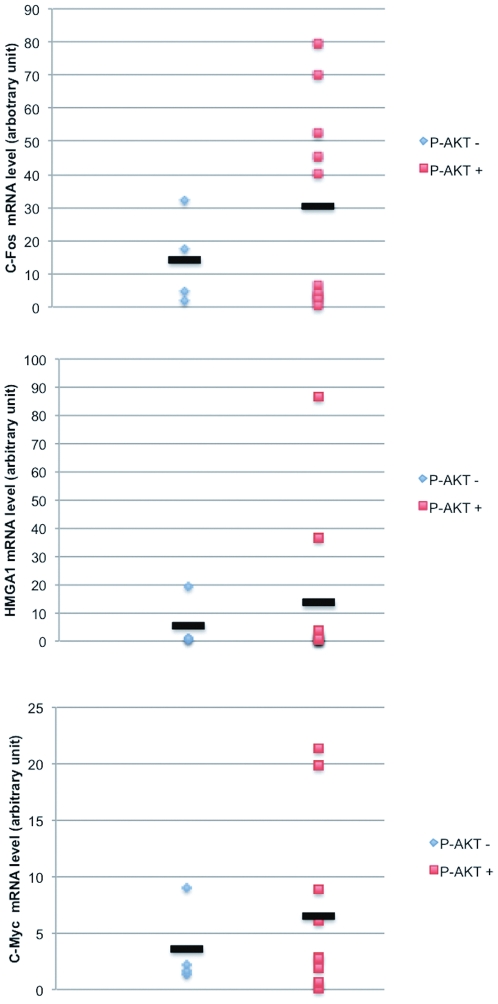
Activation of PI3KCA regulates expression of HMGA1, c-Fos, c-MYC in primary NSCLC. Quantitative real-time RT-PCR analysis of c-Fos, HMGA1, c-Myc gene expression in primary NSCLCs.

Subsequently, we extended the analysis performed in cancer cell lines to primary NSCLC. To this aim, expression of HMGA1, FOS and MYC was determined in a representative panel of NSCLC (n = 14; 4 ADC, 8 SCC and 2 ADS) and correlated with the status of AKT activation (Figure 9). Primary NSCLC with activated AKT presented (n = 10), on average, increased expression of HMGA1, FOS and MYC compared with tumors that showed low AKT activation (n = 4). For HMGA1 the average values were: 5.4 in the case of AKT negative cells and 13.5 in the case of AKT positive cells; for FOS the average values were: 14.1 in the case of AKT negative cells and 30.5 in the case of AKT positive cells; for MYC the average values were: 3.5 in the case of AKT negative cells and 6.5 in the case of AKT positive cells (Figure 8B). However, it is to be noted that in both cell lines and tumours, data showed a trend that was not statistically significant given the low number of samples analysed and the huge heterogeneity of expression shown by HMGA1, FOS and MYC in tumors.

## Discussion

We report a detailed analysis of the contribution of the different members of PI3K/AKT pathway to AKT deregulation in lung cancer. The most interesting findings of this study were that in Italian NSCLC patients activation of AKT was associated with advanced stage and higher grade and that, in these tumours, the major determinant of AKT activation was the over-expression of the catalytic subunit of phosphatidylinositol 3-kinase, p110α. Experimental evidence obtained by manipulation of PI3K signalling in NSCLC cells also indicated that p110α is required for *in vitro* and *in vivo* growth and disclosed a network of PI3K-regulated transcription factors that may be responsible for the oncogenic effects exerted by aberrant PI3K signalling in cancer [48].

To our knowledge this is the first comprehensive analysis aimed at determining the role of AKT signalling performed on a cohort of Italian NSCLC patients. So far, little information concerning AKT activation in Italian NSCLC patients was available. In the cohort of NSCLC patients studied here, AKT pathway is activated in 62% of cases, with significant S473 phosphorylation detected more frequently in patients with advanced disease (TNM stage III vs. stage II; n = 26; p<0.05) and higher grade (G3–G4 compared with G1–G2; n = 83; p<0.05). Several NSCLCs analysed in this study over-expressed PIK3CA, implying that the deregulated expression of wild type p110α might represent an oncogenic event during cancer development in the lung. Conversely, we found PIK3CA mutation in only one SCC patient, confirming that, although frequent in breast, gastric and hepatocellular cancers, PIK3CA mutations are rare in NSCLCs [49]. Other molecular lesions detected in NSCLC patients comprise PTEN loss (39%) and AKT1 or AKT2 over-expression (18% and 22%, respectively). It is of note that although PTEN loss in NSCLCs is more common than overexpression of p110α, our results indicate that the latter is the unique alteration that is significantly associated to AKT activation (p = 0.02).

Interestingly, simultaneous aberrant expression of two or more members within the PI3K pathway was relatively infrequent in unselected NSCLCs but was significantly more frequent in NSCLCs with activated AKT (see Table 4 for details). This observation suggests that p110α over-expression alone is not sufficient to activate AKT signalling and hence requires other alterations to be fully oncogenic in NSCLCs. Moreover, at difference with the significant AKT activation shown by NSCLCs with mutant KRAS or AKT1, the tumour that harboured mutant PIK3CA was negative for pAKT, suggesting that the type or the position of the alteration within the pathway may influence mechanisms and effects of pathway deregulation [45], [49]–[51]. Accordingly, KRAS mutations were mutually exclusive with other genetic alterations (except for ADC-23 who presented simultaneous presence of KRAS mutation and polysomy of AKT1 and AKT2) whereas copy number variations of PIK3CA, AKT1 and AKT2 were not [52]. These findings are reminiscent of breast or endometrial cancer, in which PIK3CA mutations are frequently detected in settings of low PTEN expression or mutations [53], [54], and suggest that genetic alterations of the PI3K/AKT pathway in NSCLCs are not functionally redundant.

In addition, this manuscript provides novel experimental evidence to the observation that SCCs and ADCs develop by different genetic alterations: i) mutations in PIK3CA and AKT1 (3% altogether) were detected only in SCCs [this manuscript; 24] whereas KRAS mutations were observed in ADCs (19%); ii) SCC patients (85%) presented at least one genetic alteration in PI3KCA, AKT1, AKT2 or PTEN more frequently than ADC patients (50%); iii) PIK3CA copy gains occurred more frequently in SCCs (25%) than in ADCs (9%) as described previously [15], [49]; iv) coexistence of at least two alterations in the members of the PI3K pathway occurred more frequently in SCC patients (45%) than in ADC patients (8%).

FISH results indicated that gene amplification of the PIK3CA gene at 3p21 is responsible for ∼20% of cases with enhanced p110α expression, in agreement with previous reports indicating that gains of part or of the entire long arm of chromosome 3, where the PIK3CA gene maps, are recurrent in NSCLCs [15], [55], [56]. Yet, since several NSCLCs overexpress p110α in the absence of gene amplification other mechanisms must be involved in the dysregulation of PIK3CA expression in NSCLCs.

The functional effects of mutant or amplified PIK3CA in lung cancer are unclear [49]. Our data indicated that in NSCLC cells, PIK3 signalling is required *in vitro* and *in vivo* since suppression of p110α expression inhibits the growth of xenografted cells carrying an activated PIK3CA allele. However, it is likely that PI3K might act in concert with other oncogenic hits to promote malignant transformation of lung epithelial cells since several NSCLCs present aberrant expression of AKT1, AKT2 or loss of PTEN in addition to PIK3CA overexpression (7%, 10% and 21%, respectively) and PIK3CA mutations are not mutually exclusive with EGFR and KRAS mutations in lung cancer [49]–[51], [54].

Finally, RNA profiling experiments led to the identification of >2000 differentially regulated transcripts that likely contributes to the oncogenic effects of aberrant PI3K signalling in lung epithelial cells. Categorization of differentially expressed genes into biological pathways and/or functions identified gene expression changes induced by the constitutive activation of PI3K-dependent signalling in lung epithelial cells. Interestingly, analysis of DEGs retrieved several functions linked to lung cancer of both ADC and SCC histotypes, suggesting that the activation of PI3K signalling induces a transcriptional programme that is characteristic of lung cancer cells. In this sense, it is worth noting that IPA analysis identified a network of transcription factors that are linked to PI3K activation - such as MYC, JUN, JUN-B, FOS, HMGA1 and HES1- that are the central nodes of multiple molecular networks up-regulated by constitutive PI3K signalling. These findings suggest that part of the oncogenic activity exerted by PI3K in lung epithelial cells is dependent on the ability of PI3K to reprogram transcription. The existence of a correlation between PI3K signalling and the expression of oncogenic transcription factors is confirmed by the finding that cell lines and primary tumours with high AKT activation present, on average, consistently higher expression of MYC, FOS and HMGA1 than cell lines and tumours with low AKT activation.

It is of note that in agreement with its role in promoting cells cycle progression, active PI3K up-regulates the expression of several cell cycle promoting molecules – CCND1, CCND2, Cdk6 – as well as down-regulates Cdk inhibitors. To this regard, it is worth noting that PI3K-dependent regulation of CCND2 expression may occur indirectly through MYC [57].

In conclusion, the results reported in this manuscript indicate that PI3KCA over-expression occur at a much higher frequency in lung cancers than do activating mutations, apparently representing the major determinant of AKT activation in NSCLC. PI3KCA overexpression in NSCLCs occurs, at least in part, through gene copy gains, which occur more often in SCCs than in ADCs. Finally, it is of particular interest the identification of a network of transcription factors that are upregulated by constitutive PI3K signalling and may represent critical mediators of the oncogenic effects exerted by aberrant PI3K.

## Supporting Information

Figure S1 **IHC for pAKT, AKT1, AKT2 and PI3KCA in normal lung.** **A.** Immunoblot for anti-AKT1 and anti-AKT2 antibodies in NCI-H460 cells interfered for AKT1 and AKT2, respectively. **B.** Top left: normal lung negative for pAKT pS473 phosphorylation; top right: normal lung negative for AKT1. Bottom left: normal lung negative for AKT2; bottom right: normal lung negative for PIK3CA. Magnification 10×.
(TIF)

Figure S2 **Immunostaining analysis of AKT1 in NSCLCs.** **A.** AKT1 expression in SCCs: from left to right, negative, (+), (++), (+++). SCC positive for AKT1 expression. **B.** AKT1 expression in ADCs: from left to right, negative, (+), (++), (+++). Magnification 10× and 40×, respectively.
(TIFF)

Figure S3 **Immunostaining analysis of AKT2 in NSCLCs.** **A.** AKT2 expression in SCCs: from left to right, negative, (+), (++), (+++). SCC positive for AKT1 expression. **B.** AKT2 expression in ADCs: from left to right, negative, (+), (++), (+++). Magnification 10× and 40×, respectively.
(TIFF)

Figure S4 **Immunostaining analysis of PIK3CA in NSCLCs.** **A.** PIK3CA expression in SCCs: from left to right, negative, (+), (++), (+++). SCC positive for AKT1 expression. **B.** PIK3CA expression in ADCs: from left to right, negative, (+), (++), (+++). Magnification 10× and 40×, respectively.
(TIFF)

Figure S5 **PTEN expression in NSCLCs.** **A.** PTEN expression in normal lung tissue. **B.** Left, SCC negative for PTEN expression; right: ADC positive for PTEN expression. Magnification 10× and 40×, respectively. **C.** Q-reverse transcriptase PCR analysis of PTEN mRNA expression in normal lung tissues and NSCLC. **D.** Q-PCR analysis of PTEN gene number in normal lung tissues and NSCLC. DNA from peripheral blood leukocytes (PBL) was used as control. PTEN copy number in PBL was set arbitrarily as 2. The average value of the PTEN gene in normal tissues was similar to the PBL value (2).
(TIFF)

Figure S6 Top Bio-functions identified by IPA in the 2122 DEGs from BEAS-2B-PI3K-E545K compared with BEAS-2B.
(TIFF)

Figure S7 **Network analysis was performed to provide a graphical representation of genes having known biological relationships.** Green icons indicate down-regulated genes and red icons indicates up-regulated genes.
(PDF)

Table S1 **Clinico-pathological features of NSCLC patients.**
(DOCX)

Table S2 **Clinico-pathological features of SCC patients.**
(DOCX)

Table S3 **Clinico-pathological features of ADC patients.**
(DOCX)

Table S4 **Correlation between AKT activation (pS473 AKT positivity) and clinico-pathological features of SCC patients.**
(DOCX)

Table S5 **Correlation between AKT activation (pS473 AKT positivity) and clinico-pathological features of ADC patients.**
(DOCX)

Table S6 **Correlation between AKT activation and the presence of genetic alterations in PI3K, AKT1 and AKT2 in NSCLCs.**
(DOCX)

Table S7 **Correlation between AKT activation and the presence of genetic alterations of PI3K, AKT1 and AKT2 in SCCs.**
(DOCX)

Table S8 **Correlation between AKT activation and the presence of genetic alterations in PI3K, AKT1 and AKT2 in ADCs.**
(DOCX)

Table S9 **Summary of the genetic alterations in the PI3K/AKT pathway in SCC patients.** Copy number gains in AKT1, AKT2, PI3KCa genes were determined by FISH: high polysomy (HP) and gene amplification (A). Mutation analysis identified activating mutations of PI3KCA (E545K), KRAS (G12C, G12V, G12A, G13C) and AKT1(E17K). PTEN expression was classified as (+) when staining was detected in >50% of the cells, (+/−) when staining was detected in 25–50% of cells and (−) when staining was detected in 0–25% of cells. AKT activation was evaluated with phospho-specific antibodies (pS473), scored as negative (<10% of the tumour cells with weak, focal immunopositivity or absence of staining) and high (>10% of tumour cells with strong or diffuse immunopositivity).
(DOCX)

Table S10 **Summary of the genetic alterations in the PI3K/AKT pathway in ADC patients.** Copy number gains in AKT1, AKT2, PI3KCa genes were determined by FISH: high polysomy (HP) and gene amplification (A). Mutation analysis identified activating mutation of PI3KCA (E545K), KRAS (G12C, G12V, G12A, G13C) and AKT1(E17K). PTEN expression was classified as (+) when staining was detected in >50% of the cells, (+/−) when staining was detected in 25–50% of cells and (−) when staining was detected in 0–25% of cells. AKT activation was evaluated with phospho-specific antibodies (pS473), scored as negative (<10% of the tumour cells with weak, focal immunopositivity or absence of staining) and high (>10% of tumour cells with strong or diffuse immunopositivity.
(DOCX)

Table S11 **Genes significantly increased or decreased in BEAS-2B vs BEAS-PI3KCA-E545K.** Expression microarray (HT-12_V3_0_R1) data were prefiltered to remove genes changing less than 1.5 fold, and a t-test was run to determine significant (p<0.01) changers. A multiple testing correction using the algorithm of Benjamini and Hochberg was used to reduce the false discovery rate. See file attached.
(XLS)

Appendix S1 Protocols and primers for Q-PCR (PTEN), Q-RT-PCR (PTEN, c-Fos, HMGA-1, c-Myc, Jun-B) and sequencing KRAS (exons 2 and 3) and PIK3CA (exons 9 and 20).
(DOC)
