# Expression of Concern: Signaling Networks Associated with AKT Activation in Non-Small Cell Lung Cancer (NSCLC): New Insights on the Role of Phosphatydil-Inositol-3 kinase

**DOI:** 10.1371/journal.pone.0349359

**Published:** 2026-05-14

**Authors:** The PLOS One Editors

After this article [1] was published, the *PLOS One* Editors identified concerns about the article’s peer review and with Figs 1-6 and S1-S5. Specifically:

- There appear to be vertical discontinuities between lanes 2 and 3 in the PI3KCA and Tubulin panels of Fig 6B.
- There appear to be vertical discontinuities between lanes 2 and 3 in the left AKT1 and left Actin panels of Fig S1A.
- Multiple panels across Figs 2-6 and S2-5 appear similar to each other, including with 180° rotation in some cases.

The corresponding author stated that lanes were removed between lanes 2 and 3 in the left AKT1 and left Actin panels of Fig S1A in order to cut out additional interfered clones that were not described in [1], and that the two spliced images for the left AKT1 and left Actin panels in Fig S1A originated from single AKT1 and Actin blots respectively (S7 File). PLOS therefore considers the concerns with Fig S1A resolved.

The corresponding author stated it is unlikely there is a discontinuity between lanes 2 and 3 of the PI3KCA and Tubulin panels in Fig 6B. They provided original underlying data for Figs 1-6 and S1-S5 (S1–S11 Files) but stated that the original films of the PI3KCA and Tubulin western blots in Fig 6B, and the data underlying the PTEN mRNA analysis and PTEN CN analysis executed by PCR and RT-PCR in Figs S5C and S5D, are no longer available. In the absence of the underlying data for Fig 6B, this concern cannot be fully resolved.

Regarding the panel similarities across Figs 1-5 and S2-5, the corresponding author stated that some cases intentionally show the same image in multiple panels, and that in each pair, the sample was used to compare a negative and a positive in [1], while the same sample was used to show how IHC staining was graded in the Supporting Information of [1]. For other cases they stated that they are different images of two serially cut, adjacent slides stained with the indicated antibody. The corresponding author also stated that the pAKT staining in Figs 1B and 5D are derived from the same patient. The original underlying image data for Figs 1-5 and S2-5 are provided here in S1-S5 and S8–S11 Files. PLOS therefore considers these concerns resolved.

In light of the peer review concerns which were not resolved in our discussions with the authors, the *PLOS One* Editors issue this Expression of Concern. Readers are advised to interpret the article [1] with caution.

We regret that the peer review issues were not addressed prior to the article’s publication.

## Supporting information

S1 File Underlying data in support of Figure 1. (ZIP)

S2 File Underlying data in support of Figure 2. (ZIP)

S3 File Underlying data in support of Figure 3. (ZIP)

S4 File Underlying data in support of Figure 4. (ZIP)

S5 File Underlying data in support of Figure 5. The electropherograms were derived from an ABIPRISM 3100 sequencer. The melting curve for PI3KCA mutations are images taken from a Roche Lightcycler machine.
(ZIP)

S6 File Underlying data in support of Figs 6A, C and D. (ZIP)

S7 File Underlying data in support of Figure S1. (ZIP)

S8 File Underlying data in support of Figure S2. (ZIP)

S9 File Underlying data in support of Figure S3A. (ZIP)

S10 File Underlying data in support of Figure S3B. (ZIP)

S11 File Underlying data in support of Figure S4. (ZIP)

S12 File Underlying data in support of Figs S5A and S5B. (ZIP)
