# Correction: Expression of Concern: Signaling Networks Associated with AKT Activation in Non-Small Cell Lung Cancer (NSCLC): New Insights on the Role of Phosphatydil-Inositol-3 kinase

**DOI:** 10.1371/journal.pone.0350591

**Published:** 2026-06-01

**Authors:** The PLOS One Editors

There is an error in the second sentence of the third paragraph. The correct sentence is: They provided original underlying data for Figs 1-6 and S1-S5 (S1–S12 Files) but stated that the original films of the PI3KCA and Tubulin western blots in Fig 6B, and the data underlying the PTEN mRNA analysis and PTEN CN analysis executed by PCR and RT-PCR in Figs S5C and S5D, are no longer available.

There is an error in the fourth sentence of the third paragraph. The correct sentence is: The original underlying image data for Figs 1-5 and S2-5 are provided here in S1-S5 and S8–S12 Files.

The publisher apologizes for the errors.
