# Supplementary figures and images for: Signaling Networks Associated with AKT Activation in Non-Small Cell Lung Cancer (NSCLC): New Insights on the Role of Phosphatydil-Inositol-3 kinase
Source: PLoS One. 2012 Feb 17;7(2):e30427. doi: 10.1371/journal.pone.0030427 (PMC3281846; doi:10.1371/journal.pone.0030427)

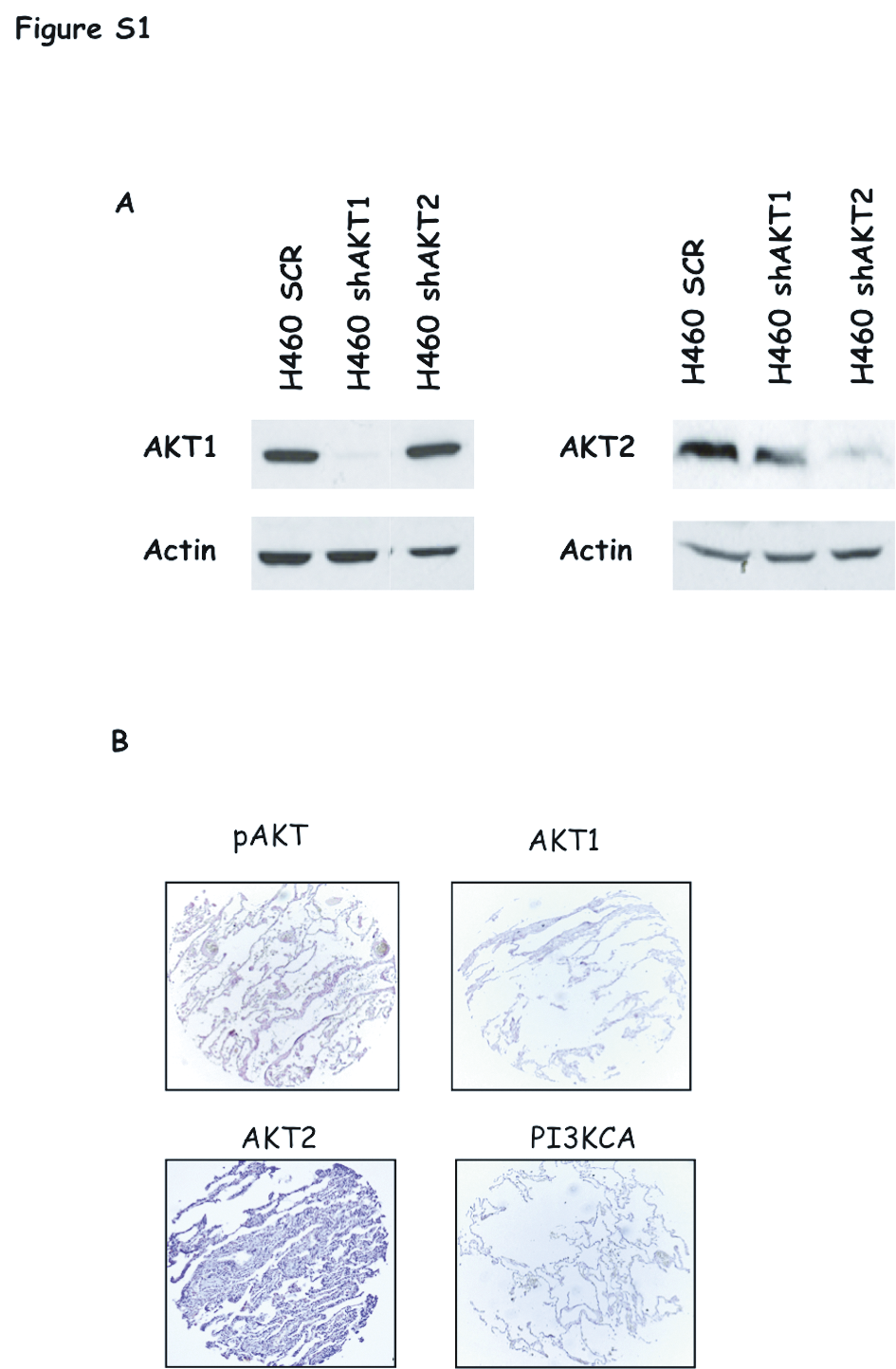

Supplement: Figure S1 — IHC for pAKT, AKT1, AKT2 and PI3KCA in normal lung. A. Immunoblot for anti-AKT1 and anti-AKT2 antibodies in NCI-H460 cells interfered for AKT1 and AKT2, respectively. B. Top left: normal lung negative for pAKT pS473 phosphorylation; top right: normal lung negative for AKT1. Bottom left: normal lung negative for AKT2; bottom right: normal lung negative for PIK3CA. Magnification 10×. (TIF) [file pone.0030427.s001.tif]

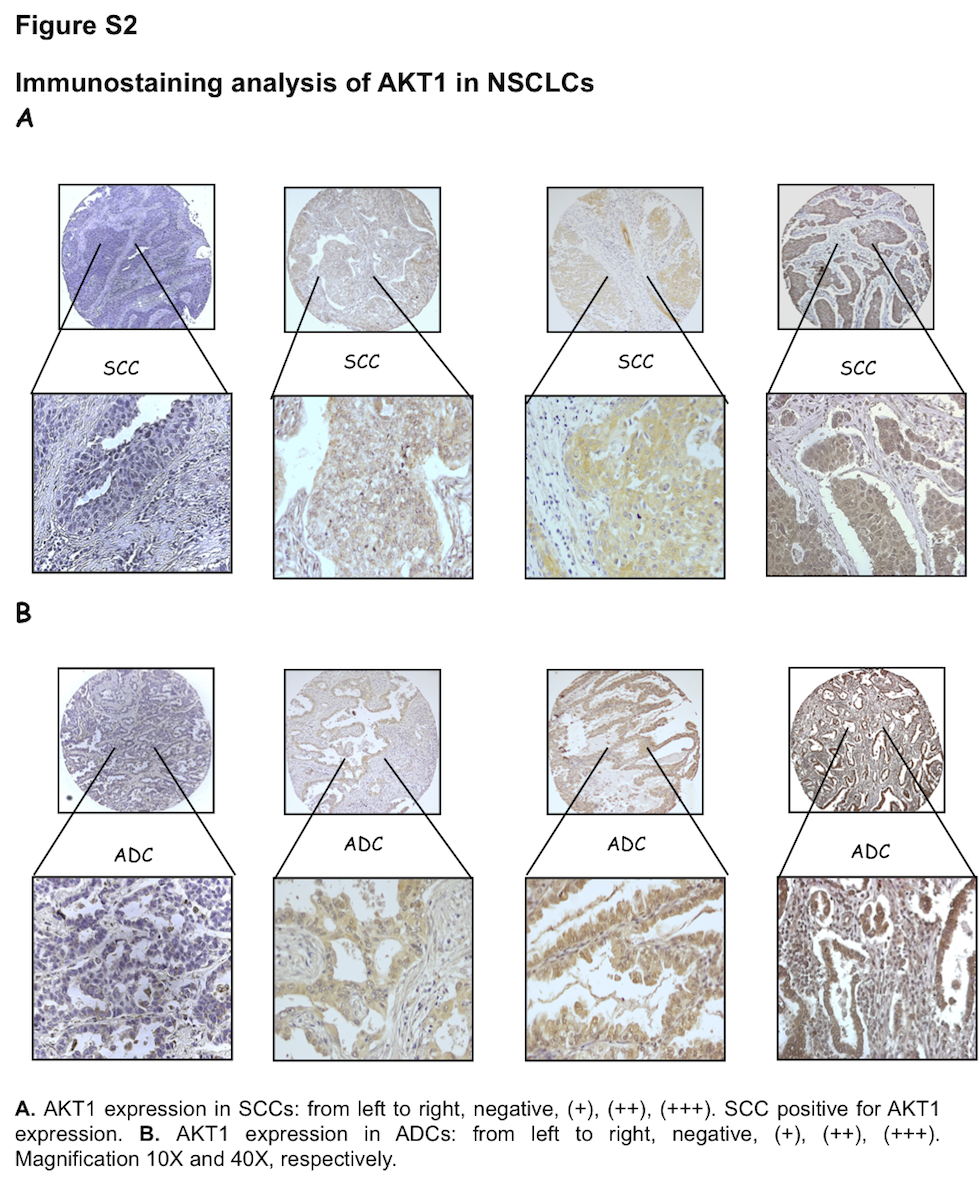

Supplement: Figure S2 — Immunostaining analysis of AKT1 in NSCLCs. A. AKT1 expression in SCCs: from left to right, negative, (+), (++), (+++). SCC positive for AKT1 expression. B. AKT1 expression in ADCs: from left to right, negative, (+), (++), (+++). Magnification 10× and 40×, respectively. (TIFF) [file pone.0030427.s002.tiff]

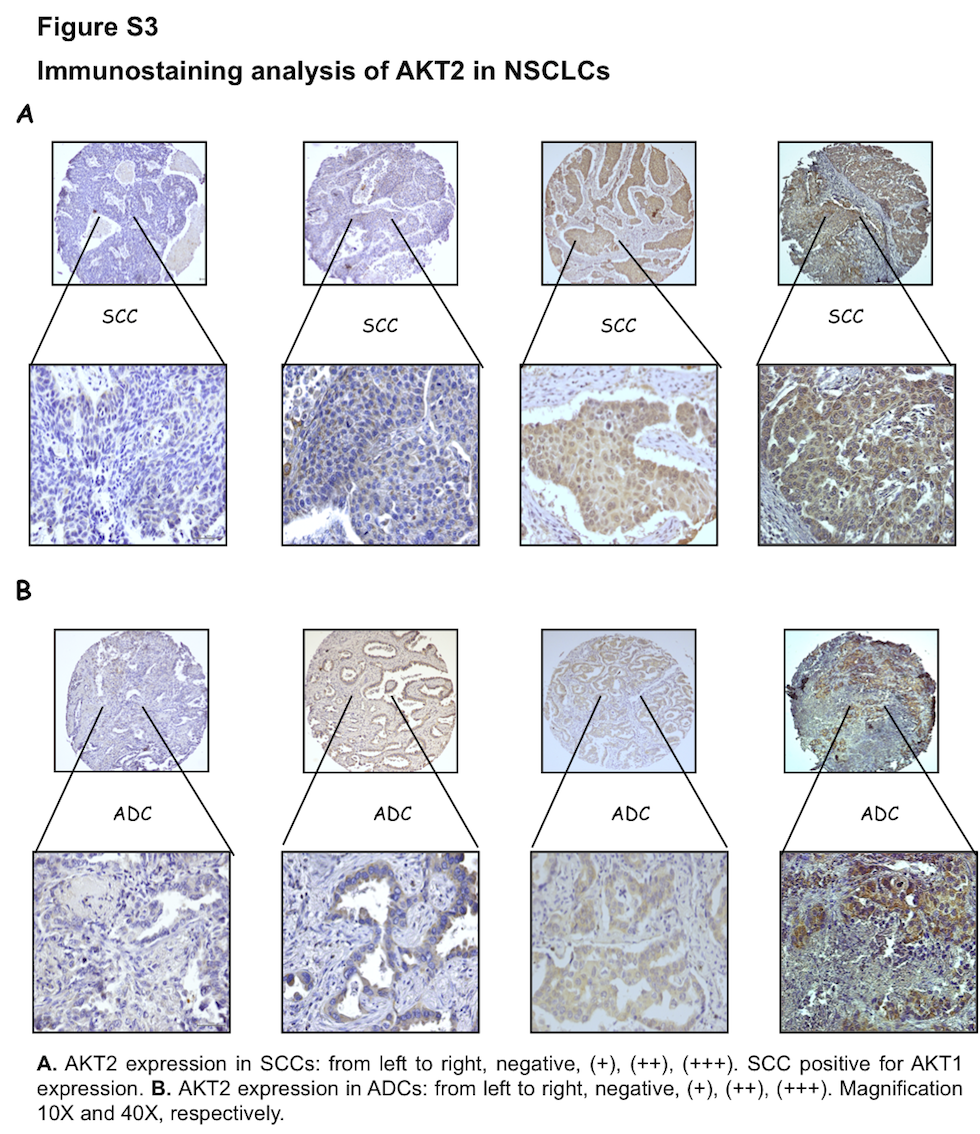

Supplement: Figure S3 — Immunostaining analysis of AKT2 in NSCLCs. A. AKT2 expression in SCCs: from left to right, negative, (+), (++), (+++). SCC positive for AKT1 expression. B. AKT2 expression in ADCs: from left to right, negative, (+), (++), (+++). Magnification 10× and 40×, respectively. (TIFF) [file pone.0030427.s003.tiff]

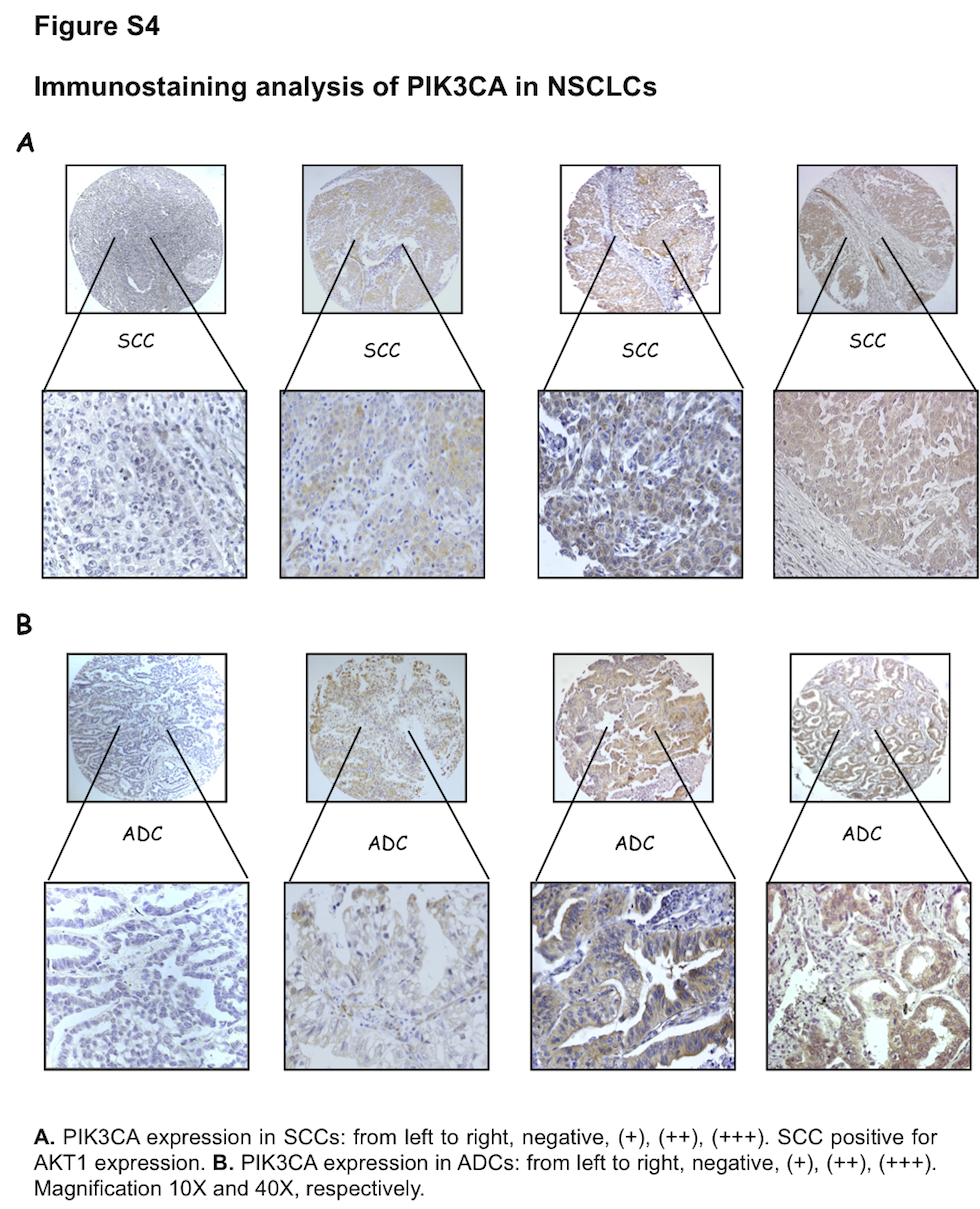

Supplement: Figure S4 — Immunostaining analysis of PIK3CA in NSCLCs. A. PIK3CA expression in SCCs: from left to right, negative, (+), (++), (+++). SCC positive for AKT1 expression. B. PIK3CA expression in ADCs: from left to right, negative, (+), (++), (+++). Magnification 10× and 40×, respectively. (TIFF) [file pone.0030427.s004.tiff]

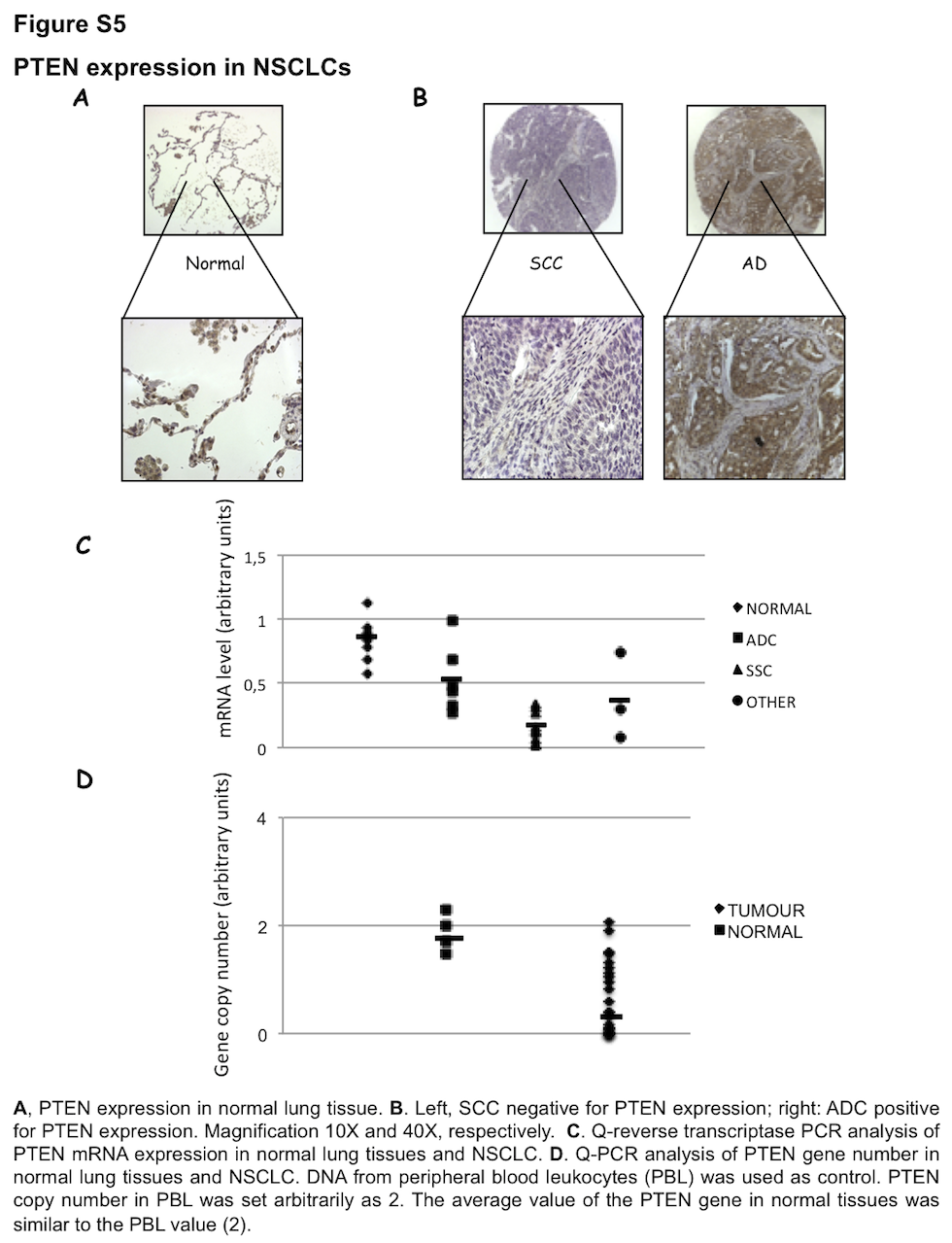

Supplement: Figure S5 — PTEN expression in NSCLCs. A. PTEN expression in normal lung tissue. B. Left, SCC negative for PTEN expression; right: ADC positive for PTEN expression. Magnification 10× and 40×, respectively. C. Q-reverse transcriptase PCR analysis of PTEN mRNA expression in normal lung tissues and NSCLC. D. Q-PCR analysis of PTEN gene number in normal lung tissues and NSCLC. DNA from peripheral blood leukocytes (PBL) was used as control. PTEN copy number in PBL was set arbitrarily as 2. The average value of the PTEN gene in normal tissues was similar to the PBL value (2). (TIFF) [file pone.0030427.s005.tiff]

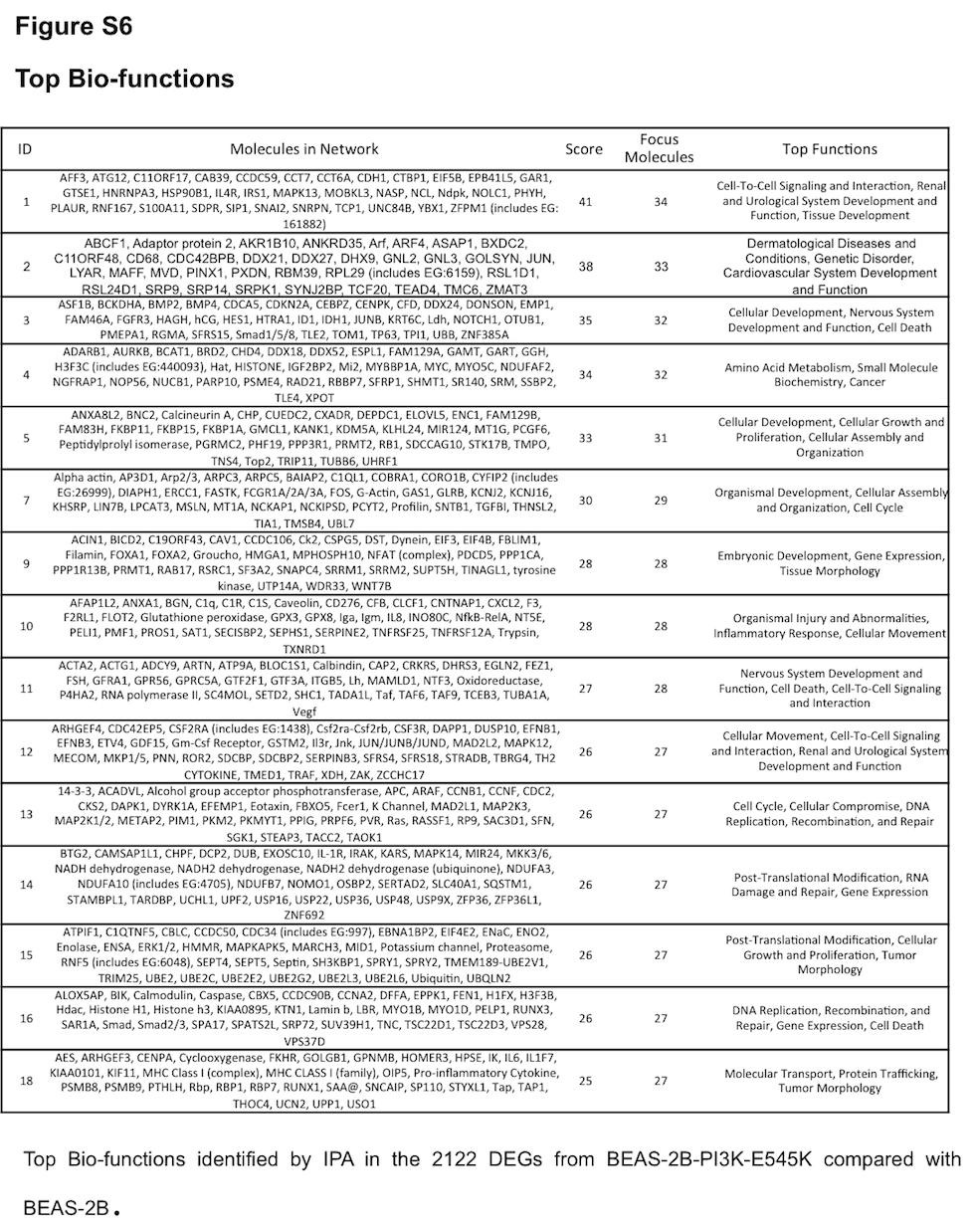

Supplement: Figure S6 — Top Bio-functions identified by IPA in the 2122 DEGs from BEAS-2B-PI3K-E545K compared with BEAS-2B. (TIFF) [file pone.0030427.s006.tiff]
