# Supplementary material for: Signaling Networks Associated with AKT Activation in Non-Small Cell Lung Cancer (NSCLC): New Insights on the Role of Phosphatydil-Inositol-3 kinase
Source: PLoS One. 2012 Feb 17;7(2):e30427. doi: 10.1371/journal.pone.0030427 (PMC3281846; doi:10.1371/journal.pone.0030427)
Supplement: Figure S7 — Network analysis was performed to provide a graphical representation of genes having known biological relationships. Green icons indicate down-regulated genes and red icons indicates up-regulated genes. (PDF) [file pone.0030427.s007.pdf]

Figure S7

Network analysis was performed to provide a graphical representation of genes having known biological relationships.

A

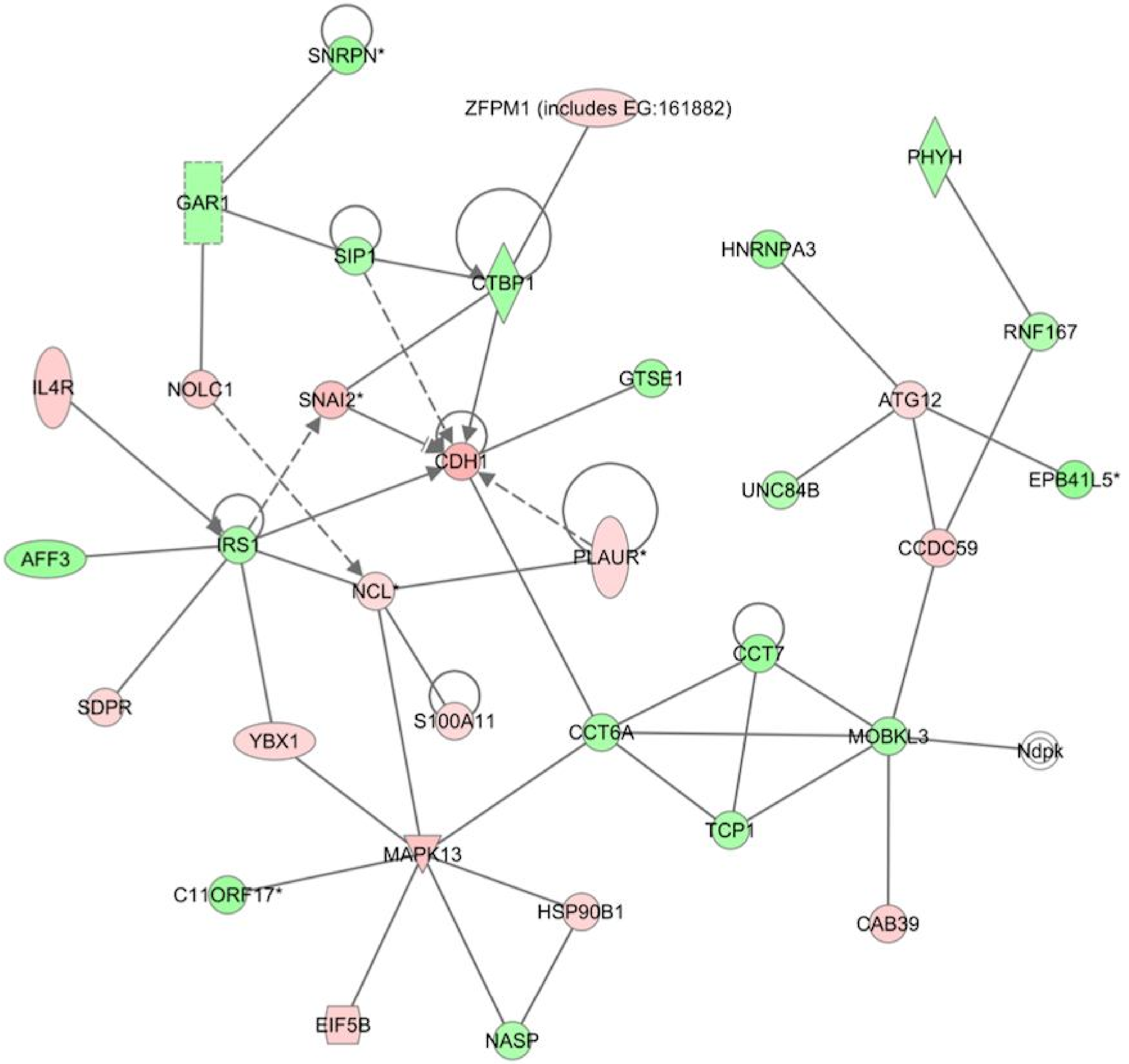

© 2000-2010 Ingenuity Systems, Inc. All rights reserved.

NETWORK 1

Green icons indicate down-regulated genes and red icons indicates up-regulated genes.

Figure S7

Network analysis was performed to provide a graphical representation of genes having known biological relationships.

B

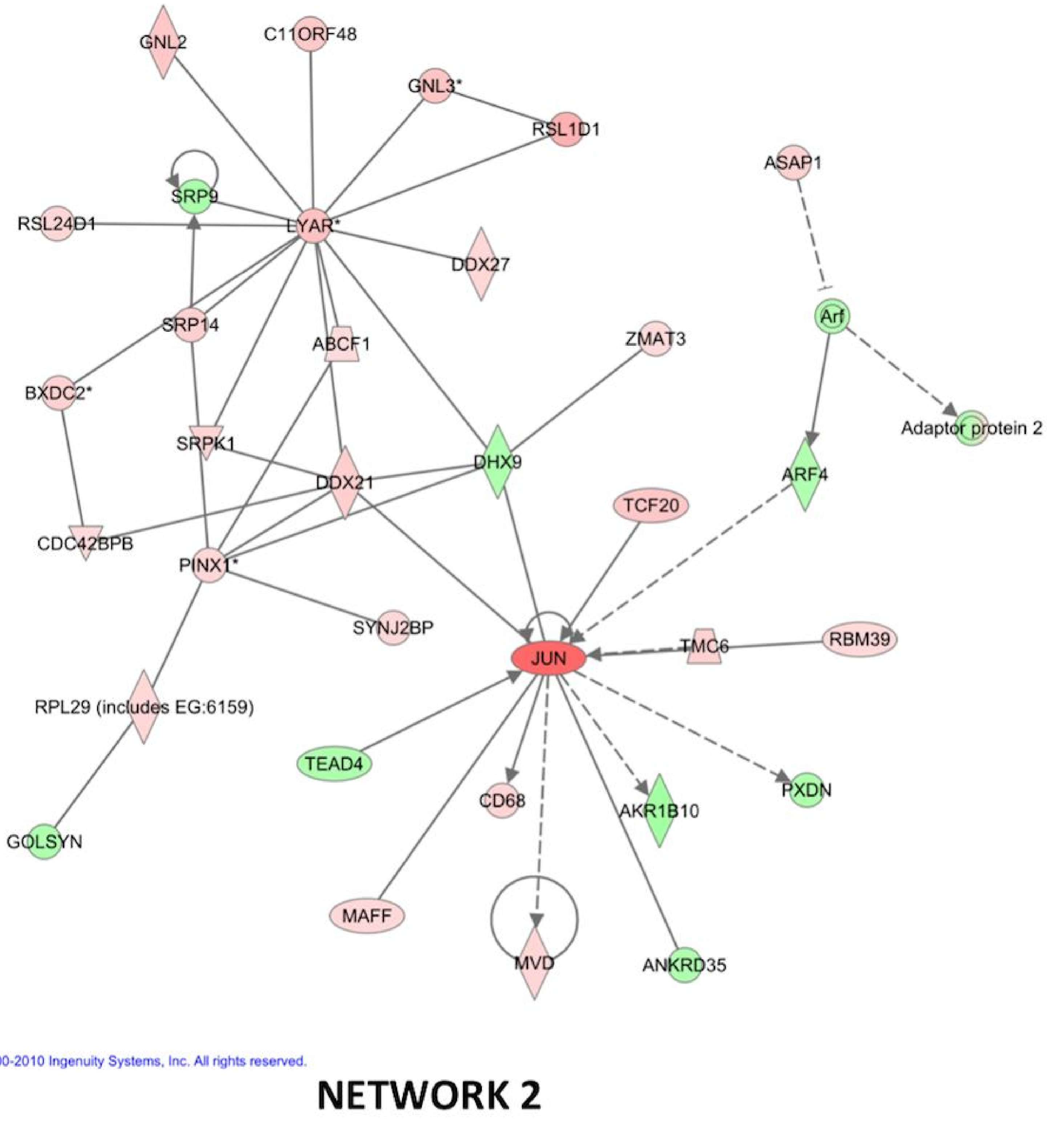

© 2000-2010 Ingenuity Systems, Inc. All rights reserved.

NETWORK 2

Figure S7

Network analysis was performed to provide a graphical representation of genes having known biological relationships.

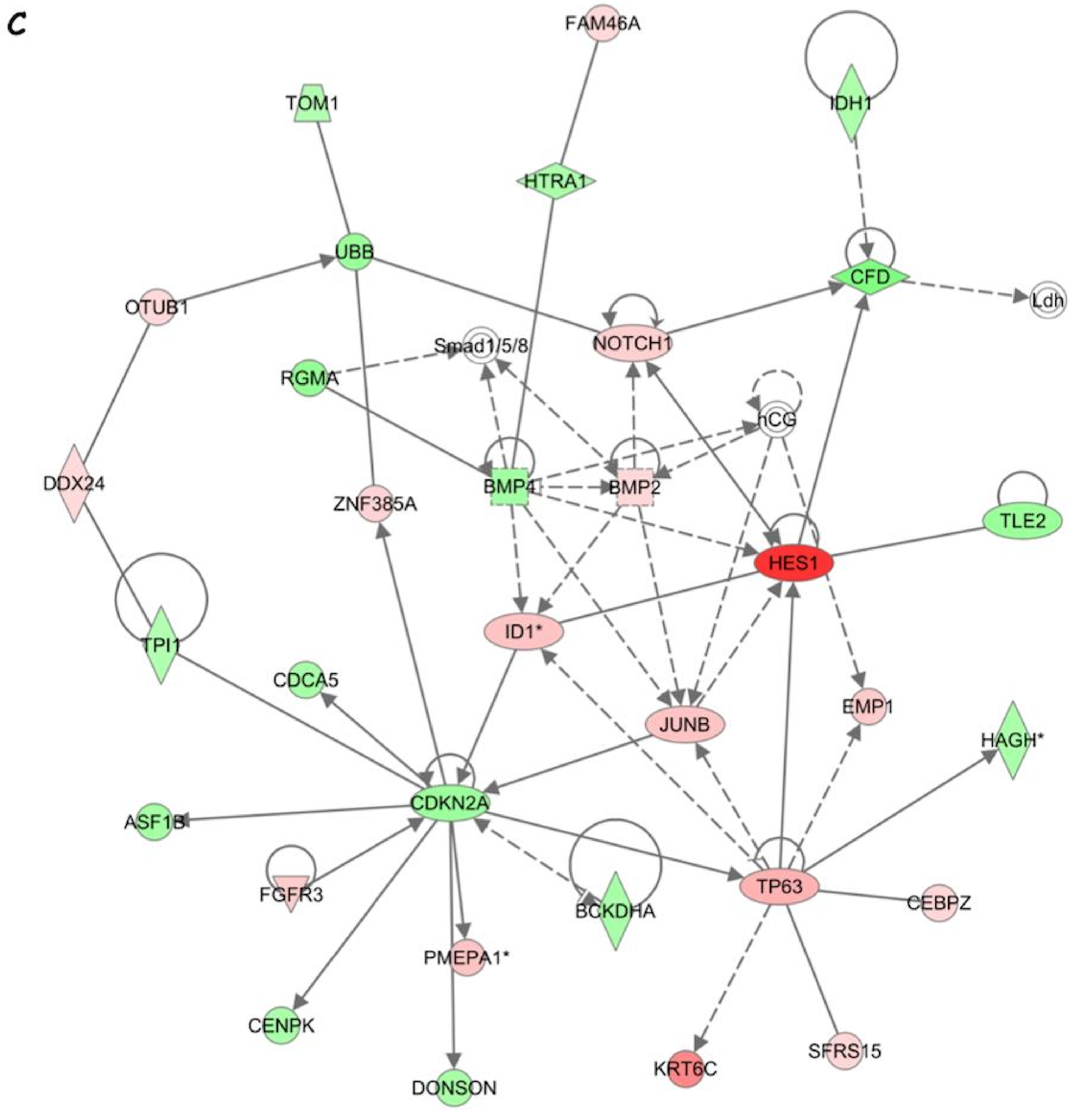

© 2000-2010 Ingenuity Systems, Inc. All rights reserved.

NETWORK 3

Figure S7

Network analysis was performed to provide a graphical representation of genes having known biological relationships.

D

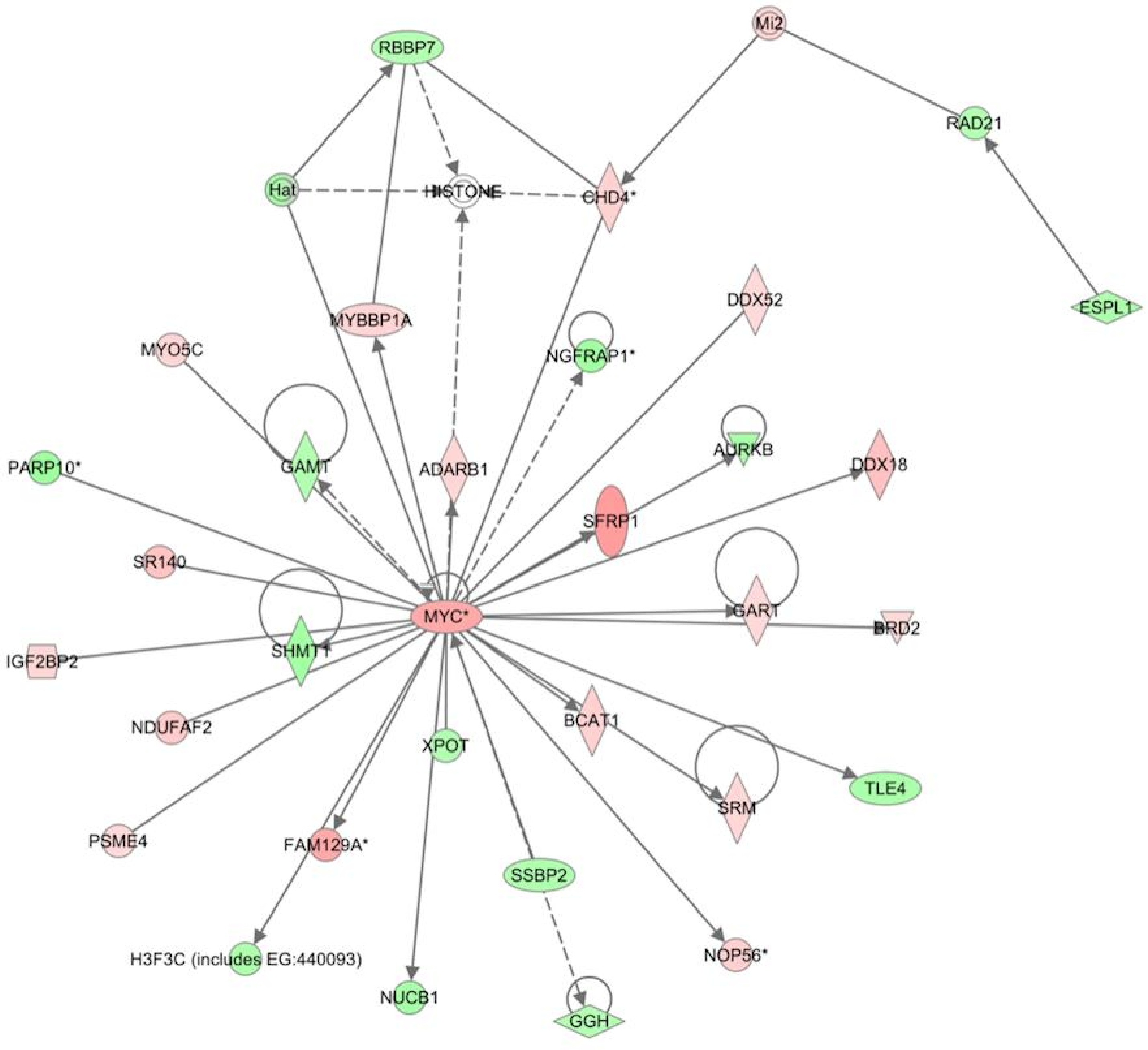

© 2000-2010 Ingenuity Systems, Inc. All rights reserved.

NETWORK 4

Figure S7

Network analysis was performed to provide a graphical representation of genes having known biological relationships.

E

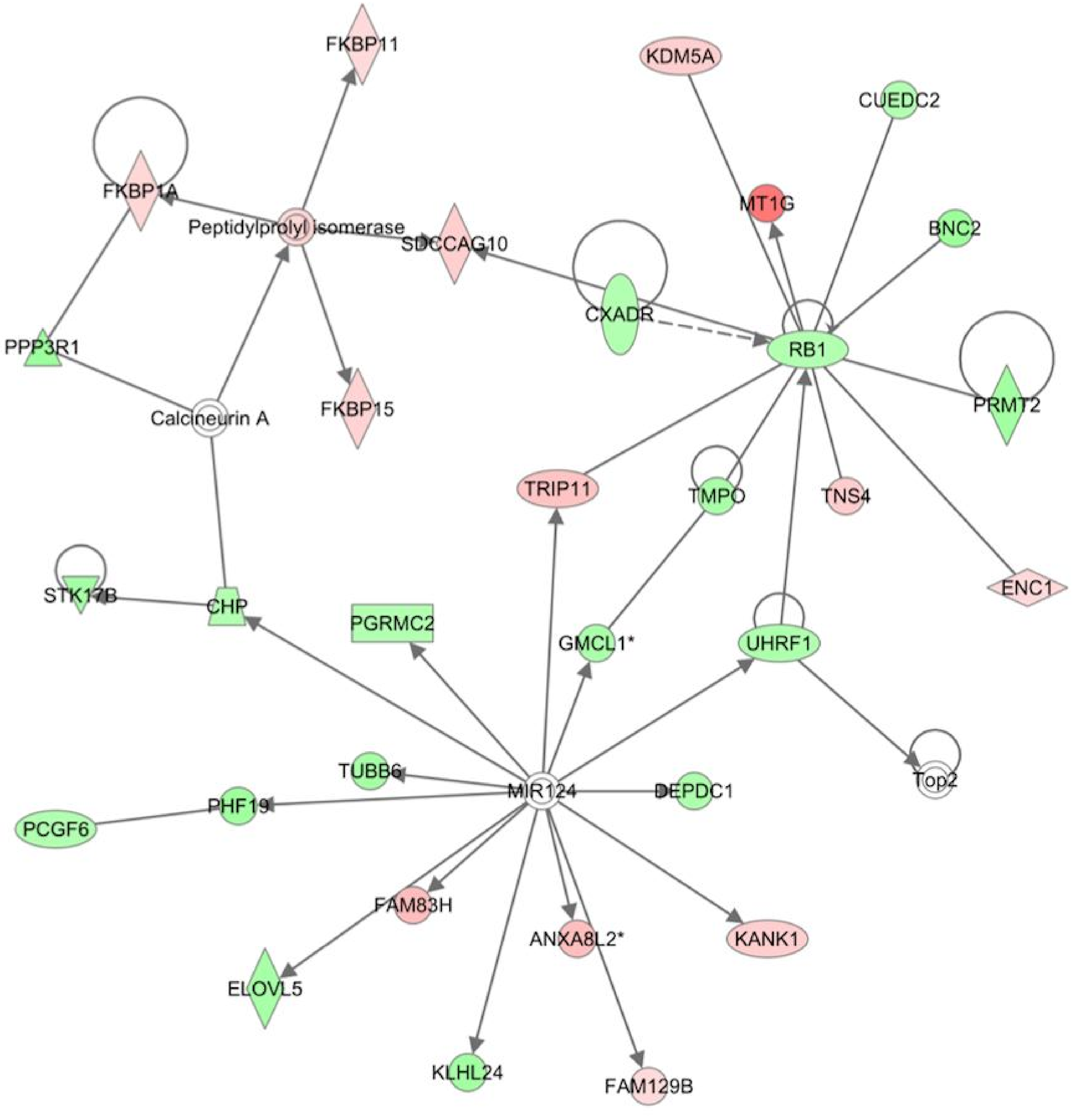

© 2000-2010 Ingenuity Systems, Inc. All rights reserved.

NETWORK 5

Figure S7

Network analysis was performed to provide a graphical representation of genes having known biological relationships.

F

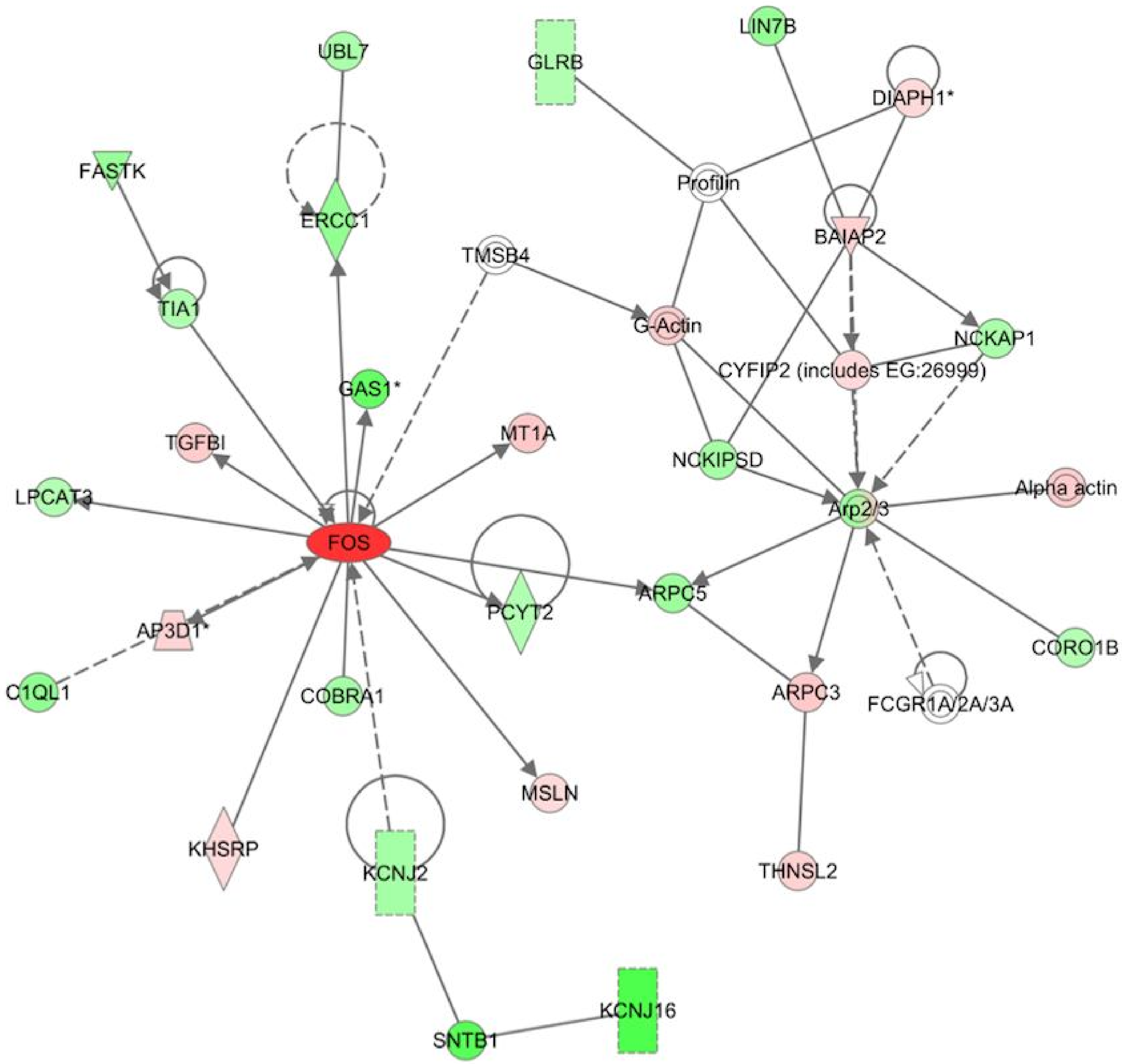

© 2000-2010 Ingenuity Systems, Inc. All rights reserved.

NETWORK 7

Figure S7

Network analysis was performed to provide a graphical representation of genes having known biological relationships.

G

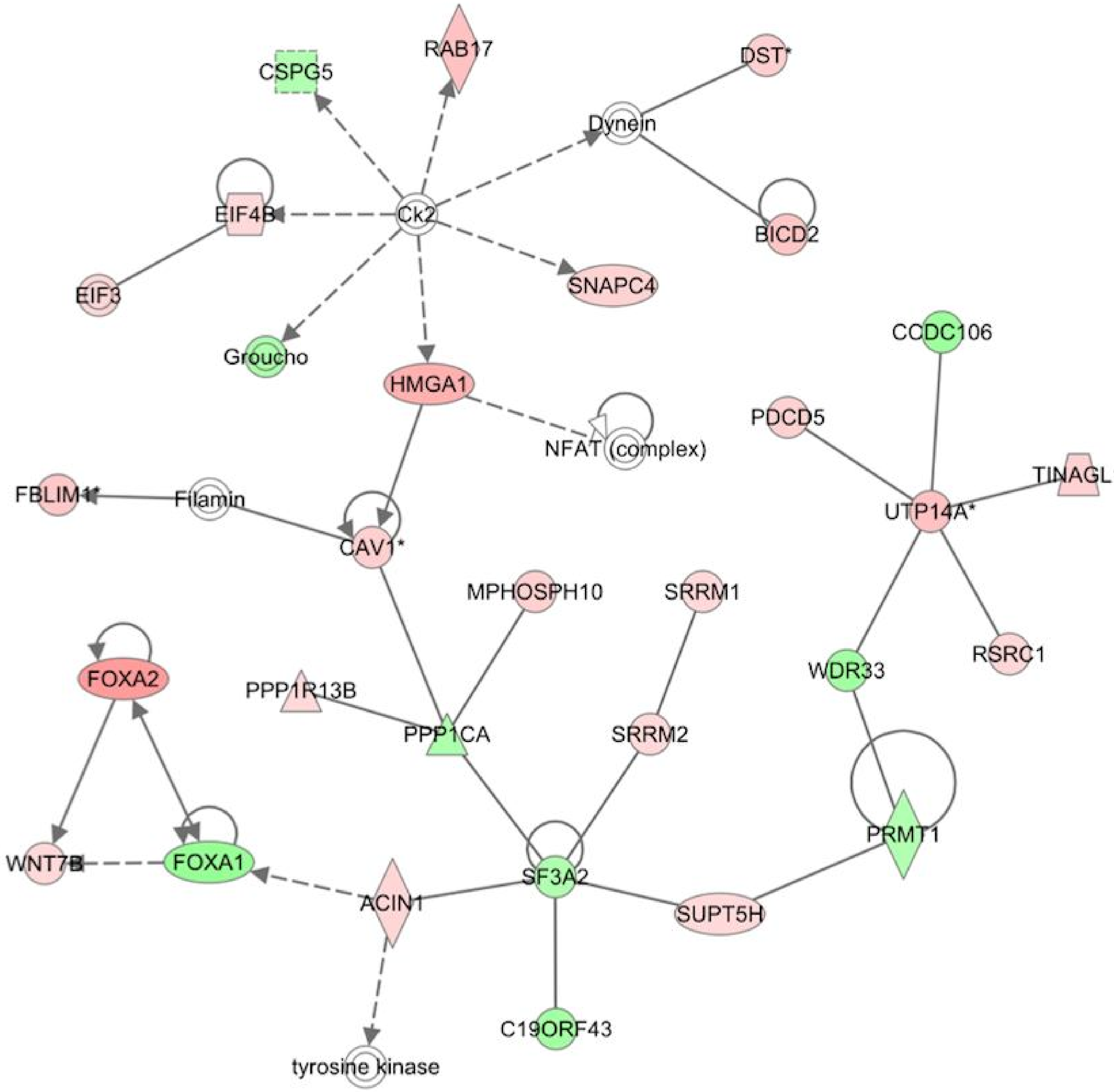

© 2000-2010 Ingenuity Systems, Inc. All rights reserved.

NETWORK 9

Figure S7

Network analysis was performed to provide a graphical representation of genes having known biological relationships.

H

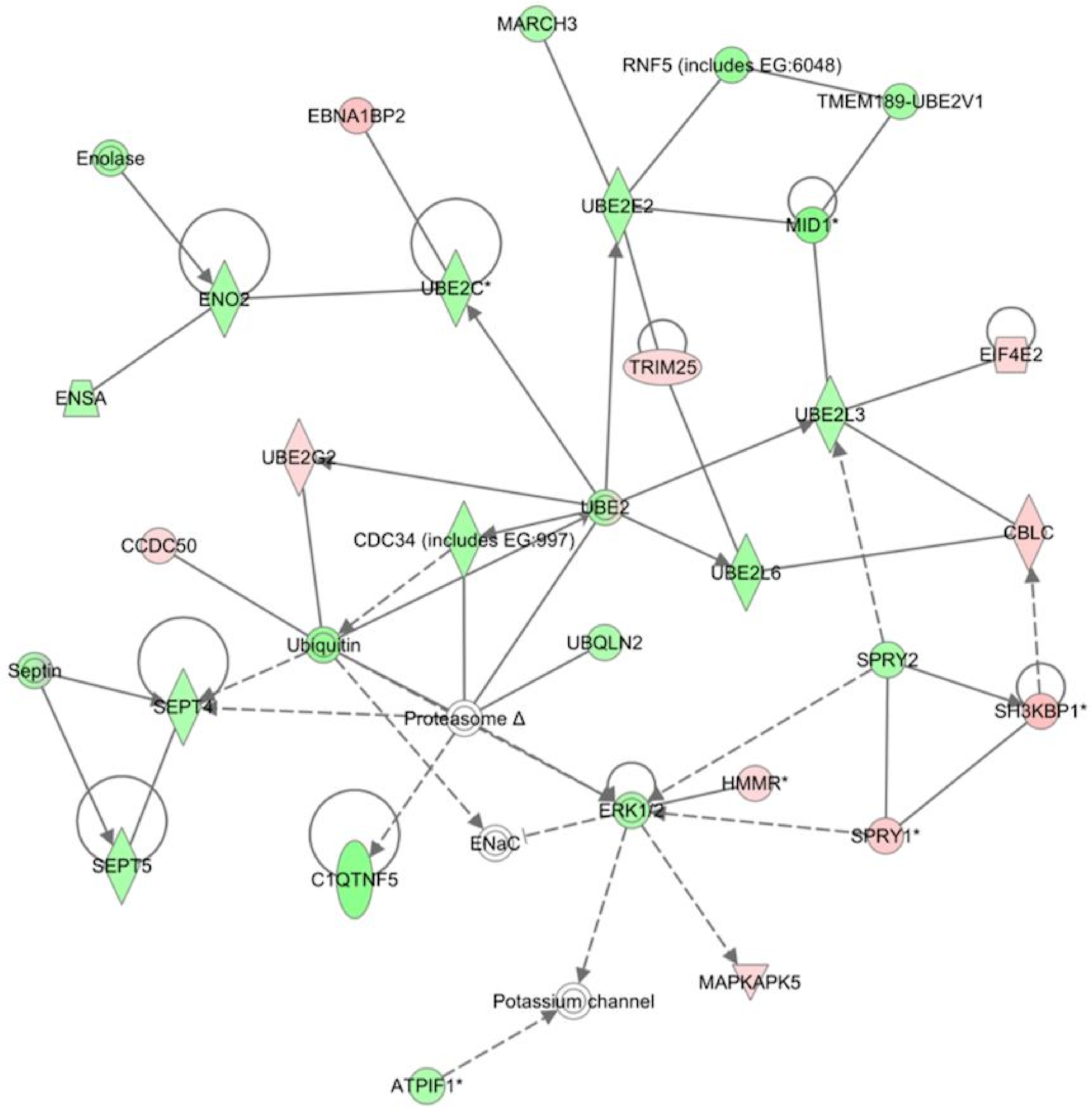

© 2000-2010 Ingenuity Systems, Inc. All rights reserved.

NETWORK 15
