# Supplementary material for: Signaling Networks Associated with AKT Activation in Non-Small Cell Lung Cancer (NSCLC): New Insights on the Role of Phosphatydil-Inositol-3 kinase
Source: PLoS One. 2012 Feb 17;7(2):e30427. doi: 10.1371/journal.pone.0030427 (PMC3281846; doi:10.1371/journal.pone.0030427)
Supplement: Table S1 — Clinico-pathological features of NSCLC patients. (DOCX) [file pone.0030427.s008.docx]

**Table S1. Clinico-pathological features of NSCLC patients**

| **Characteristics** | **Patients n** | **%** |
| --- | --- | --- |
| **Age** |  |  |
| < 60 y.o. | 28 | 30% |
| > 60 y.o. | 66 | 70% |
| **Gender** |  |  |
| Male < 60 y.o | 19 | 19% |
| Male > 60 y.o | 64 | 63% |
| Female < 60 y.o. | 10 | 10% |
| Female > 60 y.o. | 8 | 8% |
| **Histopathology** |  |  |
| Adenocarcinoma | 44 | 46% |
| Adenosquamous carcinoma | 7 | 7% |
| Squamous cell carcinoma | 37 | 38% |
| Carcinoid tumor | 3 | 3% |
| Large cell carcinoma | 6 | 6% |
| **Tumour Grade** |  |  |
| G1-G2 | 35 | 42% |
| G3-G4 | 48 | 58% |
| **Tumour Volume** |  |  |
| T1 | 28 | 33% |
| T2 | 49 | 57% |
| T3 | 4 | 5% |
| T4 | 4 | 5% |
| **Lynph Node Involvement** |  |  |
| N0 | 66 | 79% |
| N1 | 7 | 8% |
| N2-N3 | 11 | 13% |
| **Tumour Stage** |  |  |
| Ia | 20 | 25% |
| Ib | 35 | 43% |
| IIa | 0 | 0% |
| IIb | 12 | 15% |
| IIIa | 10 | 12% |
| IIIb | 4 | 5% |
