# Supplementary material for: Signaling Networks Associated with AKT Activation in Non-Small Cell Lung Cancer (NSCLC): New Insights on the Role of Phosphatydil-Inositol-3 kinase
Source: PLoS One. 2012 Feb 17;7(2):e30427. doi: 10.1371/journal.pone.0030427 (PMC3281846; doi:10.1371/journal.pone.0030427)
Supplement: Table S2 — Clinico-pathological features of SCC patients. (DOCX) [file pone.0030427.s009.docx]

**Table S2. Clinico-pathological features of SCC patients**

|  | **Characteristics** | **Patients n** | **%** |  |
| --- | --- | --- | --- | --- |
|  | **Age** |  |  |  |
|  | < 60 y.o. | 7 | 19% |  |
|  | > 60 y.o. | 29 | 81% |  |
|  | **Gender** |  |  |  |
|  | Male < 60 y.o | 5 | 14% |  |
|  | Male > 60 y.o | 27 | 75% |  |
|  | Female < 60 y.o. | 2 | 5.5% |  |
|  | Female > 60 y.o. | 2 | 5.5% |  |
|  | **Tumour Grade** |  |  |  |
|  | G1-G2 | 12 | 35% |  |
|  | G3-G4 | 22 | 65% |  |
|  | **Tumour Volume** |  |  |  |
|  | T1 | 8 | 23% |  |
|  | T2 | 22 | 63% |  |
|  | T3 | 3 | 9% |  |
|  | T4 | 2 | 5% |  |
|  | **Lynph Node Involvement** |  |  |  |
|  | N0 | 27 | 82% |  |
|  | N1 | 4 | 12% |  |
|  | N2-N3 | 2 | 6% |  |
|  | **Tumour Stage** |  |  |  |
|  | Ia | 6 | 18% |  |
|  | Ib | 17 | 50% |  |
|  | IIa | 0 | 0% |  |
|  | IIb | 7 | 21% |  |
|  | IIIa | 2 | 5.5% |  |
|  | IIIb | 2 | 5.5% |  |
