# Supplementary material for: Signaling Networks Associated with AKT Activation in Non-Small Cell Lung Cancer (NSCLC): New Insights on the Role of Phosphatydil-Inositol-3 kinase
Source: PLoS One. 2012 Feb 17;7(2):e30427. doi: 10.1371/journal.pone.0030427 (PMC3281846; doi:10.1371/journal.pone.0030427)
Supplement: Table S3 — Clinico-pathological features of ADC patients. (DOCX) [file pone.0030427.s010.docx]

**Table S3: Clinico-pathological features of ADC patients**

|  | **Characteristics** | **Patients n** | **%** |  |
| --- | --- | --- | --- | --- |
|  | **Age** |  |  |  |
|  | < 60 y.o. | 13 | 32% |  |
|  | > 60 y.o. | 28 | 68% |  |
|  | **Gender** |  |  |  |
|  | Male < 60 y.o | 9 | 22% |  |
|  | Male > 60 y.o | 24 | 58% |  |
|  | Female < 60 y.o. | 4 | 10% |  |
|  | Female > 60 y.o. | 4 | 10% |  |
|  | **Tumour Grade** |  |  |  |
|  | G1-G2 | 16 | 48% |  |
|  | G3-G4 | 17 | 52% |  |
|  | **Tumour Volume** |  |  |  |
|  | T1 | 17 | 47% |  |
|  | T2 | 17 | 47% |  |
|  | T3 | 0 | 0% |  |
|  | T4 | 2 | 6% |  |
|  | **Lynph Node Involvement** |  |  |  |
|  | N0 | 27 | 75% |  |
|  | Nx-N1 | 2 | 5% |  |
|  | N2-N3 | 7 | 19% |  |
|  | **Tumour Stage** |  |  |  |
|  | Ia | 13 | 40% |  |
|  | Ib | 10 | 30% |  |
|  | IIa | 0 | 0% |  |
|  | IIb | 2 | 6% |  |
|  | IIIa | 6 | 18% |  |
|  | IIIb | 2 | 6% |  |
