# Supplementary material for: Signaling Networks Associated with AKT Activation in Non-Small Cell Lung Cancer (NSCLC): New Insights on the Role of Phosphatydil-Inositol-3 kinase
Source: PLoS One. 2012 Feb 17;7(2):e30427. doi: 10.1371/journal.pone.0030427 (PMC3281846; doi:10.1371/journal.pone.0030427)
Supplement: Table S4 — Correlation between AKT activation (pS473 AKT positivity) and clinico-pathological features of SCC patients. (DOCX) [file pone.0030427.s011.docx]

**Table S4. Correlation between AKT activation (pS473 AKT positivity) and clinico-pathological features of SCC patients**

|  | **AKT activation (pS473)** | | |
| --- | --- | --- | --- |
|  | **Low (*n*)** | **High (*n*)** | ***P value*** |
| **Gender** |  |  |  |
| Male | 11 | 21 | 0.116 |
| Female | 3 | 1 |  |
| **Grade*^a^*** |  |  |  |
| G1-G2 | 5 | 7 | 0.966 |
| G3-G4 | 9 | 13 |  |
| **TNM stage *^b^*** |  |  |  |
| Stages I | 9 | 14 | *0.17 |
| Stage II | 4 | 3 | **0.4 |
| Stage III | 0 | 4 | ***0.125 |
|  |  |  | ****0.058 |

*Stage I vs Stage II vs StageIII

**Stage I vs Stage II

***Stage I vs Stage III

****Stage II vs Stage III
