# Supplementary material for: Signaling Networks Associated with AKT Activation in Non-Small Cell Lung Cancer (NSCLC): New Insights on the Role of Phosphatydil-Inositol-3 kinase
Source: PLoS One. 2012 Feb 17;7(2):e30427. doi: 10.1371/journal.pone.0030427 (PMC3281846; doi:10.1371/journal.pone.0030427)
Supplement: Table S5 — Correlation between AKT activation (pS473 AKT positivity) and clinico-pathological features of ADC patients. (DOCX) [file pone.0030427.s012.docx]

**Table S5. Correlation between AKT activation (pS473 AKT positivity) and clinico-pathological features of ADC patients**

| **Characteristics** | **AKT activation (pS473)** | | |
| --- | --- | --- | --- |
|  | **Low (*n*)** | **High (*n*)** | ***P value*** |
| **Gender** |  |  |  |
| Male | 10 | 23 | 0.695 |
| Female | 3 | 5 |  |
| **Grade*^a^*** |  |  |  |
| G1-G2 | 8 | 8 | §0.049 |
| G3-G4 | 3 | 14 |  |
| **TNM stage *^b^*** |  |  |  |
| Stages I | 7 | 16 | *0.642 |
| Stage II | 0 | 2 | **0.358 |
| Stage III | 2 | 6 | ***0.771 |
|  |  |  | ****0.429 |

§ G3-G4 vs G1-G2

*Stage I vs Stage II vs StageIII

**Stage I vs Stage II

***Stage I vs Stage III

****Stage II vs Stage III
