# Supplementary material for: Signaling Networks Associated with AKT Activation in Non-Small Cell Lung Cancer (NSCLC): New Insights on the Role of Phosphatydil-Inositol-3 kinase
Source: PLoS One. 2012 Feb 17;7(2):e30427. doi: 10.1371/journal.pone.0030427 (PMC3281846; doi:10.1371/journal.pone.0030427)
Supplement: Table S6 — Correlation between AKT activation and the presence of genetic alterations in PI3K, AKT1 and AKT2 in NSCLCs. (DOCX) [file pone.0030427.s013.docx]

**Table S6. Correlation between AKT activation and the presence of genetic alterations in PI3K, AKT1 and AKT2 in NSCLCs**

|  |  | **pAKT negative ^a^** | **pAKT Positive ^a^** | **Total number** |
| --- | --- | --- | --- | --- |
| **PIK3CA^b^** | Negative | 16 | 29 | 45 |
|  | Positive | 7 | 11 | 18 |
| **AKT1^c^** | Negative | 21 | 38 | 59 |
|  | Positive | 9 | 10 | 19 |
| **AKT2^d^** | Negative | 16 | 29 | 45 |
|  | Positive | 9 | 14 | 23 |

*^a^* AKT activation was evaluated with as pS473 positivity and scored as negative (<10% of the tumour cells with weak, focal immunopositivity or absence of staining) and high (>10% of tumour cells with strong or diffuse immunopositivity).

^b^ PIK3CA: FISH-negative samples were disomy, trisomy and low polysomy; FISH-positive samples were high polysomy and/or gene amplification.

^c^ AKT1: FISH-negative samples were disomy, trisomy and low polysomy; FISH-positive samples were high polysomy and/or gene amplification.

^d^ AKT2: FISH-negative samples were disomy, trisomy and low polysomy; FISH-positive samples were high polysomy and/or gene amplification.
