# Supplementary material for: Signaling Networks Associated with AKT Activation in Non-Small Cell Lung Cancer (NSCLC): New Insights on the Role of Phosphatydil-Inositol-3 kinase
Source: PLoS One. 2012 Feb 17;7(2):e30427. doi: 10.1371/journal.pone.0030427 (PMC3281846; doi:10.1371/journal.pone.0030427)
Supplement: Table S7 — Correlation between AKT activation and the presence of genetic alterations of PI3K, AKT1 and AKT2 in SCCs. (DOCX) [file pone.0030427.s014.docx]

**Table S7. Correlation between AKT activation and the presence of genetic alterations of PI3K, AKT1 and AKT2 in SCCs**

|  |  | **pAKT negative^a^** | **pAKT Positive^a^** | **Total number** | **P value** |
| --- | --- | --- | --- | --- | --- |
| **PI3KCA**^b^ | Negative | 6 | 9 | 15 | 0.61 |
|  | Positive | 3 | 7 | 10 |  |
| **AKT1**^c^ | Negative | 7 | 17 | 24 | 0.092 |
|  | Positive | 5 | 3 | 8 |  |
| **AKT2**^d^ | Negative | 5 | 12 | 17 | 0.494 |
|  | Positive | 5 | 7 | 12 |  |

*^a^* Akt activation was evaluated with as pS473 positivity and scored as negative (<10% of the tumour cells with weak, focal immunopositivity or absence of staining) and high (>10% of tumour cells with strong or diffuse immunopositivity).

^b^ PIK3: FISH-negative samples were disomy, trisomy and low polysomy; FISH-positive samples were high polysomy and/or gene amplification.

^c^ AKT1: FISH-negative samples were disomy, trisomy and low polysomy; FISH-positive samples were high polysomy and/or gene amplification.

^d^ AKT2: FISH-negative samples were disomy, trisomy and low polysomy; FISH-positive samples were high polysomy and/or gene amplification.
