# Supplementary material for: Signaling Networks Associated with AKT Activation in Non-Small Cell Lung Cancer (NSCLC): New Insights on the Role of Phosphatydil-Inositol-3 kinase
Source: PLoS One. 2012 Feb 17;7(2):e30427. doi: 10.1371/journal.pone.0030427 (PMC3281846; doi:10.1371/journal.pone.0030427)
Supplement: Table S8 — Correlation between AKT activation and the presence of genetic alterations in PI3K, AKT1 and AKT2 in ADCs. (DOCX) [file pone.0030427.s015.docx]

**Table S8. Correlation between AKT activation and the presence of genetic alterations in PI3K, AKT1 and AKT2 in ADCs**

|  |  | **pAKT negative^a^** | **pAKT positive^a^** | **Total number** | **P value** |
| --- | --- | --- | --- | --- | --- |
| **PI3KCA**^b^ | Negative | 8 | 17 | 25 | 0.729 |
|  | Positive | 2 | 3 | 5 |  |
| **AKT1**^c^ | Negative | 10 | 21 | 31 | 0.850 |
|  | Positive | 2 | 5 | 7 |  |
| **AKT2**^d^ | Negative | 8 | 16 | 24 | 0.643 |
|  | Positive | 3 | 4 | 7 |  |

*^a^* AKT activation was evaluated with as pS473 positivity and scored as negative (<10% of the tumour cells with weak, focal immunopositivity or absence of staining) and high (>10% of tumour cells with strong or diffuse immunopositivity).

^b^ PI3KCA: FISH-negative samples were disomy, trisomy and low polysomy; FISH-positive samples were high polysomy and/or gene amplification.

^c^ AKT1: FISH-negative samples were disomy, trisomy and low polysomy; FISH-positive samples were high polysomy and/or gene amplification.

^d^ AKT2: FISH-negative samples were disomy, trisomy and low polysomy; FISH-positive samples were high polysomy and/or gene amplification.
