# Supplementary material for: Signaling Networks Associated with AKT Activation in Non-Small Cell Lung Cancer (NSCLC): New Insights on the Role of Phosphatydil-Inositol-3 kinase
Source: PLoS One. 2012 Feb 17;7(2):e30427. doi: 10.1371/journal.pone.0030427 (PMC3281846; doi:10.1371/journal.pone.0030427)
Supplement: Table S9 — Summary of the genetic alterations in the PI3K/AKT pathway in SCC patients. Copy number gains in AKT1, AKT2, PI3KCa genes were determined by FISH: high polysomy (HP) and gene amplification (A). Mutation analysis identified activating mutations of PI3KCA (E545K), KRAS (G12C, G12V, G12A, G13C) and AKT1(E17K). PTEN expression was classified as (+) when staining was detected in >50% of the cells, (+/−) when staining was detected in 25–50% of cells and (−) when staining was detected in 0–25% of cells. AKT activation was evaluated with phospho-specific antibodies (pS473), scored as negative (<10% of the tumour cells with weak, focal immunopositivity or absence of staining) and high (>10% of tumour cells with strong or diffuse immunopositivity). (DOCX) [file pone.0030427.s016.docx]

**Table S9. Summary of the genetic alterations in the PI3K-AKT pathway in SCC patients**

| **Sample** | **Grade** | **Stage** | **Copy number gain** | **Mutation** | **PTEN** | **Akt pS473** |
| --- | --- | --- | --- | --- | --- | --- |
| SCC-1 | G3 | IB | AKT1 (HP) |  | +/- | - |
| SCC-2 | G2 |  |  |  | +/- | ND |
| SCC-3 | G3 | IB |  |  | - | + |
| SCC-4 | G2 | IA | PI3KCa (A) |  | - | - |
| SCC-5 | G2 | IIB |  |  | - | + |
| SCC-6 | G3 | IB |  | PI3KCa (ex 9) | - | - |
| SCC-7 | G2 |  |  |  | - | ND |
| SCC-8 | G2 | IIIA |  |  | - | + |
| SCC-9 | G3 | IB |  |  | +/- | + |
| SCC-10 | G2 | IA |  |  | + | + |
| SCC-11 | G2 | IIB | AKT1 (A), AKT2 (A) |  | - | + |
| SCC-12 | G3 | IB | AKT1 (A), AKT2 (HP) |  | - | - |
| SCC-13 | G3 | IIIB | PI3KCa (HP) |  | - | + |
| SCC-14 | G3 | IIB | PI3KCa (A) AKT1 (A), AKT2 (HP) |  | +/- | - |
| SCC-15 | G3 | IIB | AKT2 (HP) |  | - | - |
| SCC-16 | G3 | IB | PI3KCa (A), AKT2 (HP) |  | - | + |
| SCC-17 | G3 | IIB |  |  | - | + |
| SCC-18 | G2 | IA | AKT2 (HP) |  | - | - |
| SCC-19 | G3 | IB | PI3KCa (A), AKT2 (HP) |  | - | + |
| SCC-20 | G3 | IA |  |  | +/- | - |
| SCC-21 | G2 | IA | AKT1 (A) |  | - | - |
| SCC-22 | G3 | IB | PI3KCa (HP) |  | - | + |
| SCC-23 | G3 | IB |  |  | +/- | + |
| SCC-24 | G2 | IA | PI3KCa (HP) |  | - | ND |
| SCC-25 | G2 |  | PI3KCa (HP) |  | +/- | - |
| SCC-26 | G3 | IA | AKT1 (HP) |  | +/- | + |
| SCC-27 | G3 | IB |  |  | - | + |
| SCC-28 | G2 | IIB |  | KRAS G12C | +/- | - |
| SCC-29 | G1 | IB | AKT1 (HP) | AKT1 E17K | + | + |
| SCC-30 | G1 | IB | PI3KCa (A), AKT2 (HP) |  | +/- | + |
| SCC-31 | G3 | IB | HER2 (HP) |  | - | + |
| SCC-32 |  |  |  |  | + | - |
| SCC-33 | G3 | IB |  |  | - | + |
| SCC-34 | G1 |  | PI3KCa (A), AKT2(HP) |  | +/- | + |
| SCC-35 | G3 | IB | PI3KCa (HP), AKT2 (HP) |  | + | + |
| SCC-36 | G3 | IB |  |  | - | - |
| SCC-37 |  | IIIB |  |  | - | + |
| SCC-38 | G3 | IB |  |  | - | - |
| SCC-39 | G3 | IIB | AKT1 (HP), AKT2 (HP) |  | +/- | - |
| SCC-40 |  | IIIA | AKT2 (HP) |  | +/- | + |
