# Supplementary material for: Signaling Networks Associated with AKT Activation in Non-Small Cell Lung Cancer (NSCLC): New Insights on the Role of Phosphatydil-Inositol-3 kinase
Source: PLoS One. 2012 Feb 17;7(2):e30427. doi: 10.1371/journal.pone.0030427 (PMC3281846; doi:10.1371/journal.pone.0030427)
Supplement: Table S10 — Summary of the genetic alterations in the PI3K/AKT pathway in ADC patients. Copy number gains in AKT1, AKT2, PI3KCa genes were determined by FISH: high polysomy (HP) and gene amplification (A). Mutation analysis identified activating mutation of PI3KCA (E545K), KRAS (G12C, G12V, G12A, G13C) and AKT1(E17K). PTEN expression was classified as (+) when staining was detected in >50% of the cells, (+/−) when staining was detected in 25–50% of cells and (−) when staining was detected in 0–25% of cells. AKT activation was evaluated with phospho-specific antibodies (pS473), scored as negative (<10% of the tumour cells with weak, focal immunopositivity or absence of staining) and high (>10% of tumour cells with strong or diffuse immunopositivity. (DOCX) [file pone.0030427.s017.docx]

**Table S10. Summary of the genetic alterations in the PI3K-AKT pathway in ADC patient**

| **Samples** | **Grade** | **Stage** | **Copy number gain** | **Mutation** | **PTEN** | **Akt pS473** |
| --- | --- | --- | --- | --- | --- | --- |
| ADC-1 | G2 | IIIA | PI3KCa (HP) |  | +/- | - |
| ADC -2 | G3 |  |  |  | + | - |
| ADC -3 | G2 | IB |  |  | +/- | + |
| ADC -4 | G3 | IIIA |  | KRAS G12A | +/- | + |
| ADC -5 | G3 | IA | PI3KCa (A) AKT1 (HP) |  | - | + |
| ADC -6 |  | IA |  |  | - | - |
| ADC -7 |  |  |  |  | + | - |
| ADC -8 |  | IA |  |  | +/- | + |
| ADC -9 | G3 | IIB | AKT1 (HP) |  | +/- | + |
| ADC -10 | G2 | IA |  | KRAS G12C | + | - |
| ADC -11 | G3 | IB | AKT2 (HP) |  | - | + |
| ADC -12 |  | IA |  |  | +/- | + |
| ADC -13 |  |  |  |  | + | + |
| ADC 14 |  | IA |  | KRAS G13C | - | + |
| ADC -15 |  |  | AKT2 (HP) |  | + | ND |
| ADC -16 | G3 | IIB | AKT1 (HP), AKT2 (HP) |  | - | ND |
| ADC -17 | G3 | IA |  |  | +/- | ND |
| ADC -18 |  | IB | AKT1 (HP) |  | + | + |
| ADC -19 | G2 | IB |  |  | + | ND |
| ADC -20 |  | IIIA |  |  | +/- | + |
| ADC -21 | G3 | IB | AKT2 (HP) |  | +/- | ND |
| ADC -22 | G3 | IA |  |  | + | + |
| ADC -23 |  | IB | AKT1 (HP), AKT2 (HP) | KRAS G12C | - | + |
| ADC -24 |  |  |  |  | ND | + |
| ADC -25 | G3 | IIIA |  | KRAS G12C | +/- | + |
| ADC -26 | G3 | IIIB | AKT2 (HP) |  | + | - |
| ADC -27 | G3 | IIIA |  |  | + | + |
| ADC -28 |  | IB |  |  | - | ND |
| ADC -29 |  | IA |  |  | +/- | - |
| ADC -30 | G2 | IIB |  | KRAS G12V | +/- | + |
| ADC -31 | G2 | IA |  |  | +/- | - |
| ADC -32 |  | IB |  |  | - | + |
| ADC -33 | G3 | IIIA |  |  | + | + |
| ADC -34 | G3 |  | PI3KCa (HP) |  | +/- | - |
| ADC -35 | G2 |  | AKT1 (HP) |  | - | + |
| ADC -36 | G3 | IB |  |  | +/- | + |
| ADC -37 | G3 | IB |  |  | - | + |
| ADC -38 | G3 | IB |  |  | +/- | + |
| ADC-39 | G2 |  |  |  | +/- | - |
| ADC-40 | G2 |  |  |  | + | + |
| ADC-41 | G3 |  |  |  | +/- | + |
| ADC-42 | G2 |  | AKT2 (HP) |  | +/- | + |
| ADC-43 | G3 | IIIB | PI3KCa (HP) |  | - | + |
| ADC-44 | G2 | IA |  |  | - | - |
| ADC-45 | G2 | IA | AKT1 (HP), AKT2 (HP) |  | +/- | - |
| ADC-46 | G2 | IB | AKT2 (HP) |  | +/- | + |
| ADC-47 | G2 |  |  |  | - | + |
| ADC-48 | G2 | IIIA |  |  | - | ND |
| ADC-49 | G2 | IA |  |  | +/- | - |
| ADC-50 | G2 | IB | PI3KCa (HP) |  | + | + |
| ADC-51 | G2 | IA | AKT1 (HP), AKT2 (HP) |  | **-** | - |
