# Supplementary material for: Signaling Networks Associated with AKT Activation in Non-Small Cell Lung Cancer (NSCLC): New Insights on the Role of Phosphatydil-Inositol-3 kinase
Source: PLoS One. 2012 Feb 17;7(2):e30427. doi: 10.1371/journal.pone.0030427 (PMC3281846; doi:10.1371/journal.pone.0030427)
Supplement: Appendix S1 — Protocols and primers for Q-PCR (PTEN), Q-RT-PCR (PTEN, c-Fos, HMGA-1, c-Myc, Jun-B) and sequencing KRAS (exons 2 and 3) and PIK3CA (exons 9 and 20). (DOC) [file pone.0030427.s019.doc]

**Primers list**

**KRAS**

Fw:5’-GACTGAATATAAACTTGTGG-3’

Rev:5’-CTGTATCAAAGAATGGTCCT-3’

Fw:5’-TTTTTGAAGTAAAAGGTGCACTGTA-3’

Rev:5’-ATATTATATGCATGGCATTAGCAAAG-3’

**PIK3CA**

Primers for PIK3CA exon 9 were as follows: forward, 5’-CAGAGTAACAGACTAGCTAG -3’; reverse, 5’-TAGCACTTACCTGTGACTCC -3’ (PCR product, 138 bp). For exon 9 genotyping primers the following sensor (LC Red 640 AGGATCTCGTGTAGAAATTGCTTTGAGCTGTTCTT-phoshate) and anchor (TTTCTCCTGCTCAGTGATTTCAGAGA-Fluorescein) probes were used. The cycling conditions were as follows: initial denaturation at 95 °C for 10 min, followed by 45 cycles at 95 °C for 10 s, 58 °C for 15 s, and 72 °C for 8 s. Primers for PIK3CA exon 20 were as follows: forward, 5-CTCTGGAATGCCAGAACTAC-3; reverse, 5-ATGCTGTTTAATTGTGTGGAAG-3 (175 bp). For exon 20 genotyping, the following sensor (ACCCTAGCCTTAGATAAAACTGAGCAAGAGGCTTT-Fluorescein) and anchor (LC Red 640 GAGTATTTCATGAAACAAATGAATGCACATC) probes were used. The cycling conditions were as follows: initial denaturation at 95 °C for 10 min, followed by 45 cycles at 95 °C for 10 s, 59 °C for 15 s, and 72 °C for 7 s.

**PTEN**

PTEN forward: 5’-AATCCTCAGTTTGTGGTCT-3’;

PTEN reverse: 5’ GGTAACGGCTGAGGGAACT-3’;

GAPDH forward: 5’-GAGTCAACGGATTTGGTCGT-3’;

GAPDH reverse: 5’-GACAAGCTTCCCGTTCTCAG-3’.

Oligonucleotides sequences used as primers for Q-PCR were:

PTEN CN forward 5’-GTTTGATTGCTGCATATTTCAG-3’

PTEN CN reverse 5’-CCTGTATACGCCTTCAAGTC-3’

GAPDH CN forward: 5’-GGGCTGCTCACATATTCTGGA-3’

GAPDH CN reverse: 5’-CGCCCAATACGACCAAATCT-3’

***c-Fos***

c-Fos forward 5’-CCGGGGATAGCCTCTCTTAC-3’

c-Fos reverse 5’-GTGGGAATGAAGTTGGCACT-3’

***HMGA-1***

HMGA-1 forward 5’- GAAGGAGCCCAGCGAAGTG-3’

HMGA-1 reverse 5’- TTCTCCAGTTTTTTGGGTCTGC-3’

***c-Myc***

c-Myc forward 5′-TCAAGAGGCGAACACACAAC-3′

c-Myc reverse 5′-GGCCTTTTCATTGTTTTCCA-3′

***Jun-B***

Jun-B forward 5’-ACTCATACACAGCTACGGGATACG-3’

Jun-B reverse 5’-GGCTCGGTTTCAGGAGTTTG-3’
