# Supplementary material for: Expression of Concern: Signaling Networks Associated with AKT Activation in Non-Small Cell Lung Cancer (NSCLC): New Insights on the Role of Phosphatydil-Inositol-3 kinase
Source: PLoS One. 2026 May 14;21(5):e0349359. doi: 10.1371/journal.pone.0349359 (PMC13175380; doi:10.1371/journal.pone.0349359)
Supplement: S1 File — (ZIP) [file pone.0349359.s001.zip › FIGURE 1 LIST OF CONTENTS.docx]

Figure 1A pAKT left SCC 10x

Figure 1A pAKT left SCC 10x.tif

Figure 1A pAKT left SCC 40x

Figure 1A pAKT left SCC 40x.tiff

Figure 1A pAKT right SCC 10x

Figure 1A pAKT right SCC 10x.jpg

Figure 1A pAKT right SCC 40x

Figure 1A pAKT right SCC 40x.jpg

Figure 1B pAKT left ADC 10x

Figure 1B pAKT left ADC 10x.tif

Figure 1B pAKT left ADC40x

Figure 1B pAKT left ADC40x.tif

Figure 1B pAKT right ADC 10x.jpeg

Figure 1B pAKT right ADC 10x

Figure 1B pAKT right ADC 40x

Figure 1B pAKT right ADC 40x.jpg

FIGURES FOR SUBMISSION.ppt
