# Supplementary figures and images for: Expression of Concern: Signaling Networks Associated with AKT Activation in Non-Small Cell Lung Cancer (NSCLC): New Insights on the Role of Phosphatydil-Inositol-3 kinase (part 1 of 2)
Source: PLoS One. 2026 May 14;21(5):e0349359. doi: 10.1371/journal.pone.0349359 (PMC13175380; doi:10.1371/journal.pone.0349359)

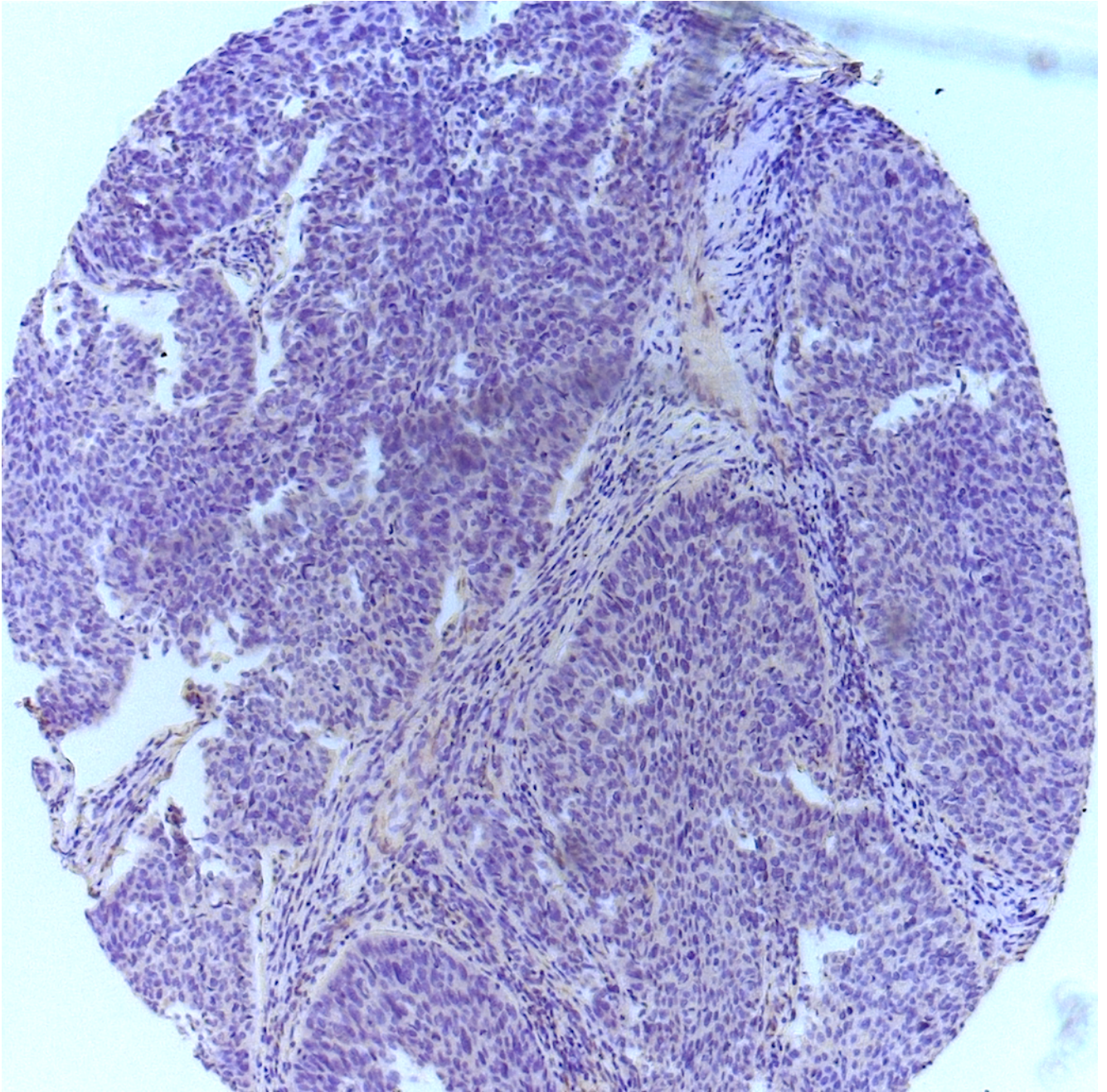

Supplement: S1 File — (ZIP) [file pone.0349359.s001.zip › Figure 1A pAKT left SCC 10x.pdf]

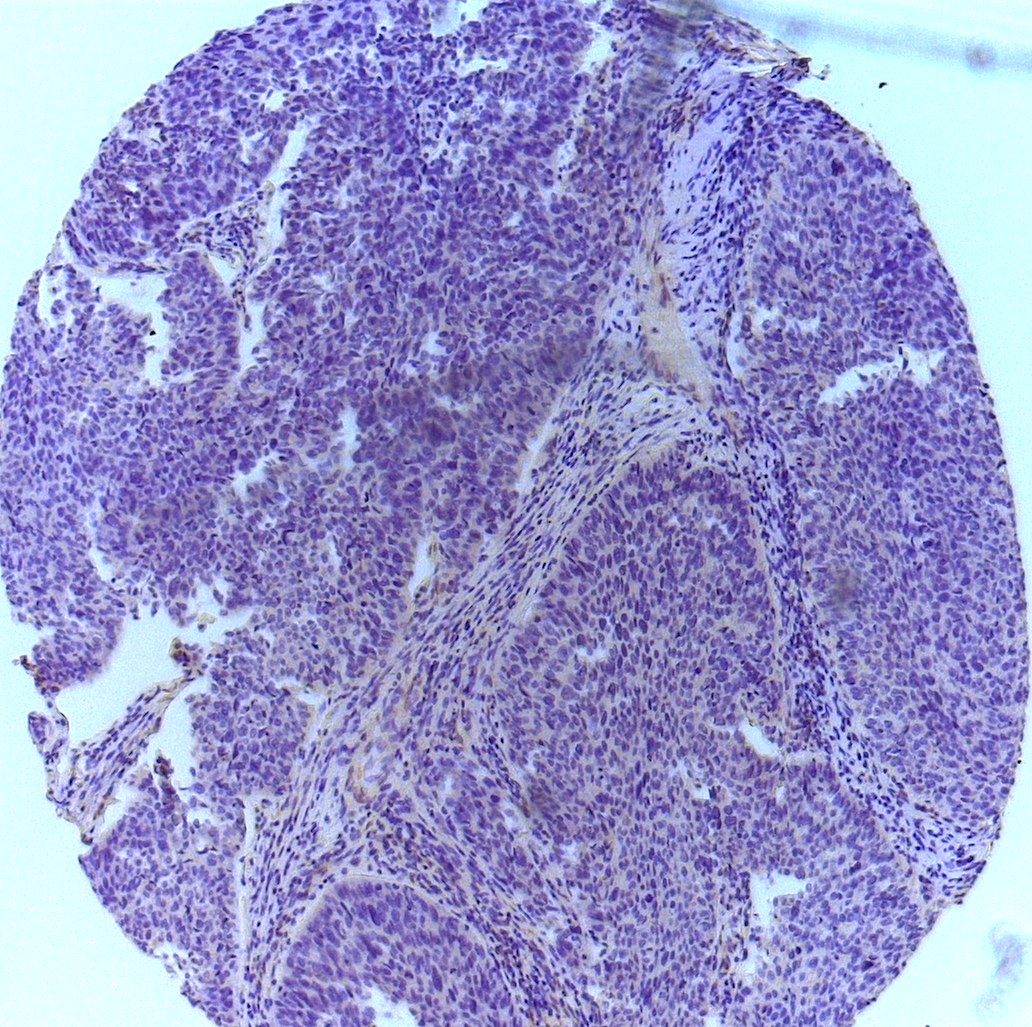

Supplement: S1 File — (ZIP) [file pone.0349359.s001.zip › Figure 1A pAKT left SCC 10x.tif]

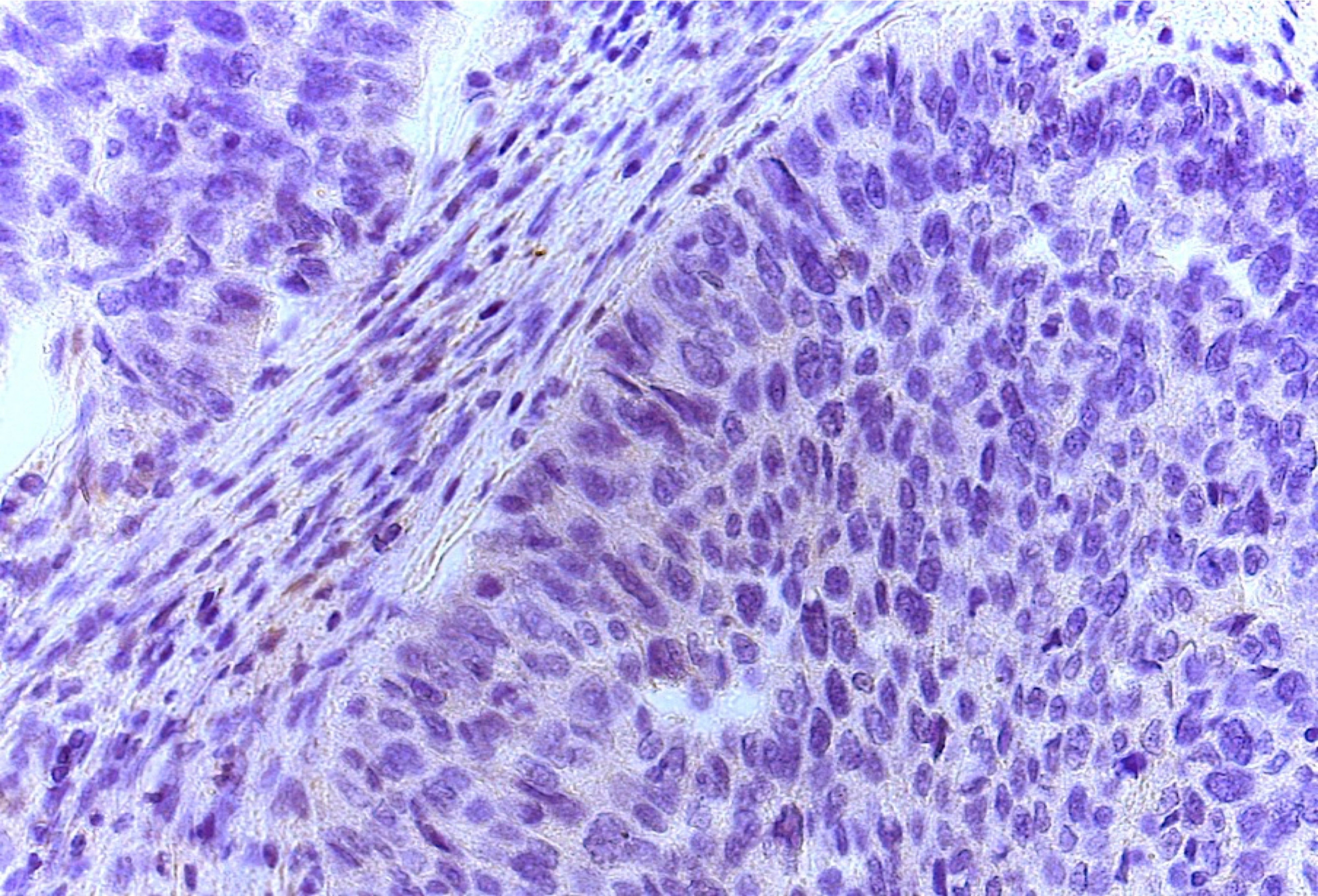

Supplement: S1 File — (ZIP) [file pone.0349359.s001.zip › Figure 1A pAKT left SCC 40x.pdf]

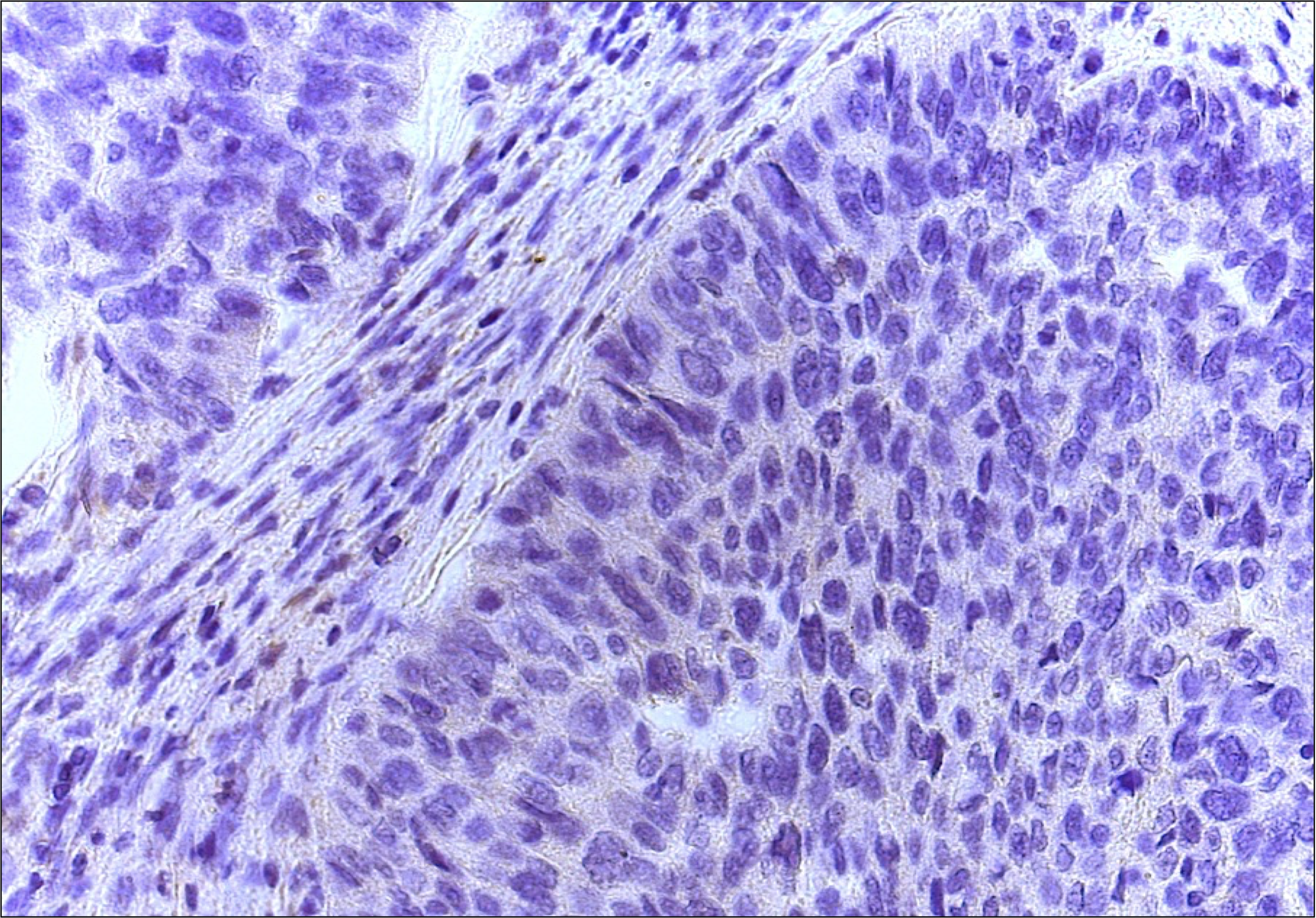

Supplement: S1 File — (ZIP) [file pone.0349359.s001.zip › Figure 1A pAKT left SCC 40x.tiff]

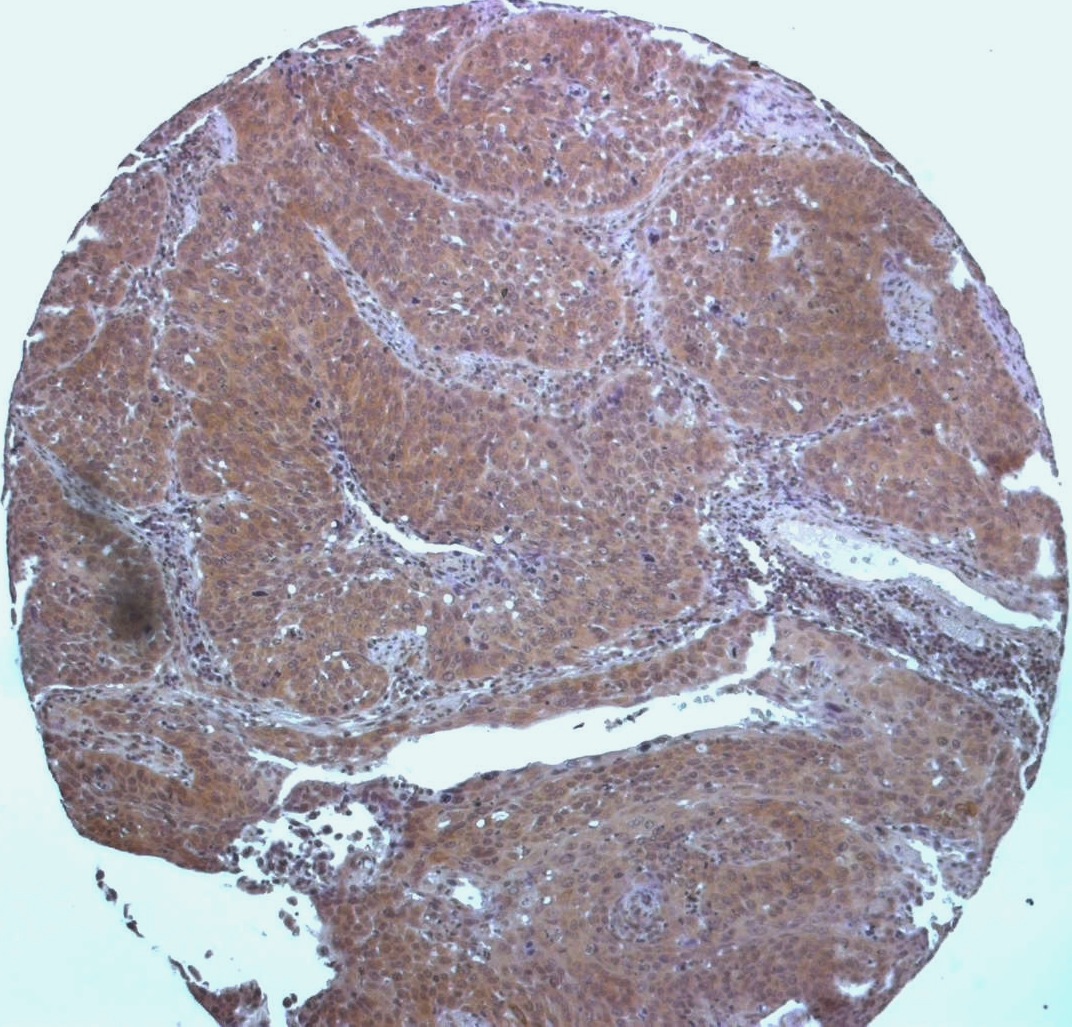

Supplement: S1 File — (ZIP) [file pone.0349359.s001.zip › Figure 1A pAKT right SCC 10x.jpg]

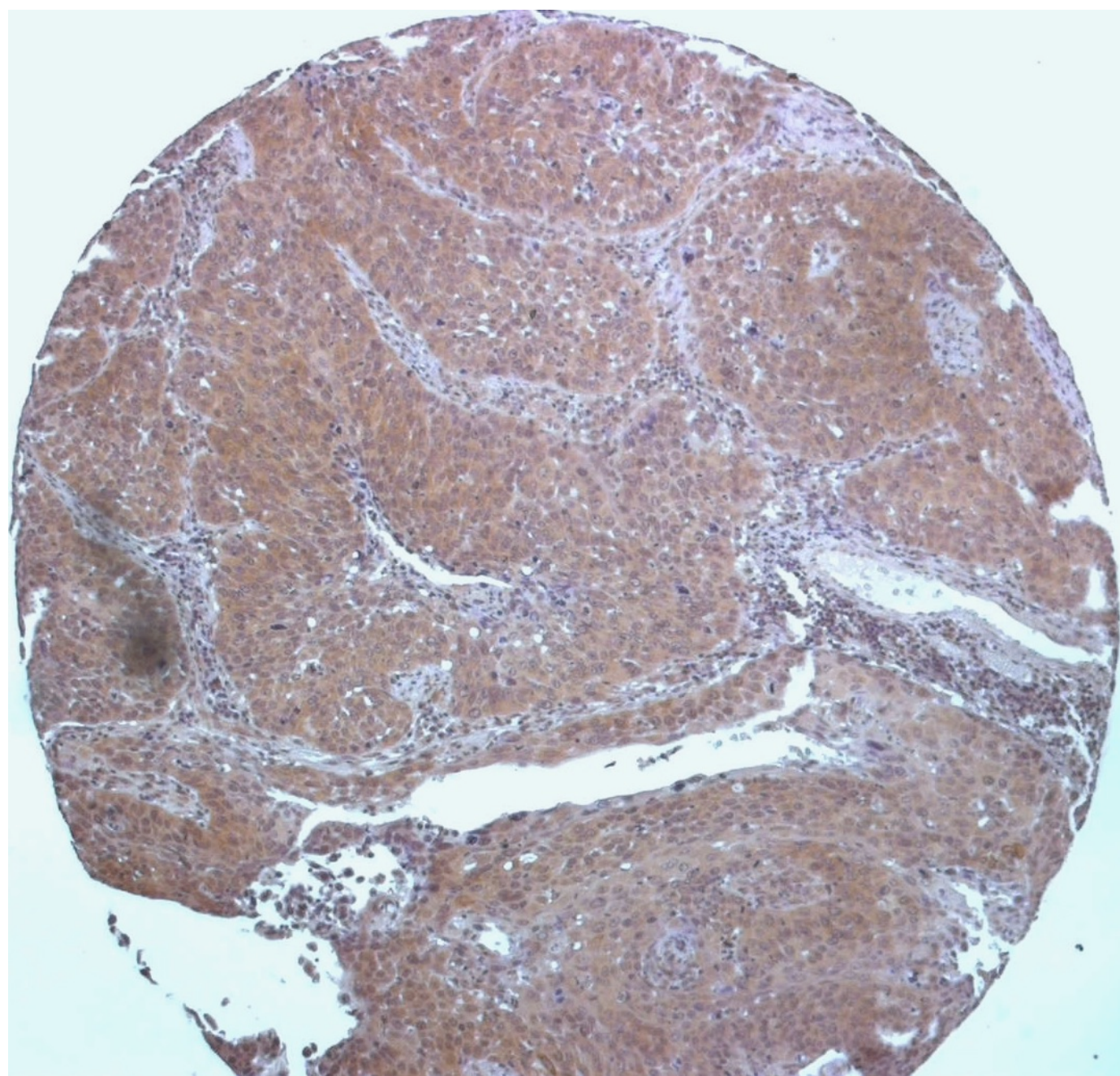

Supplement: S1 File — (ZIP) [file pone.0349359.s001.zip › Figure 1A pAKT right SCC 10x.pdf]

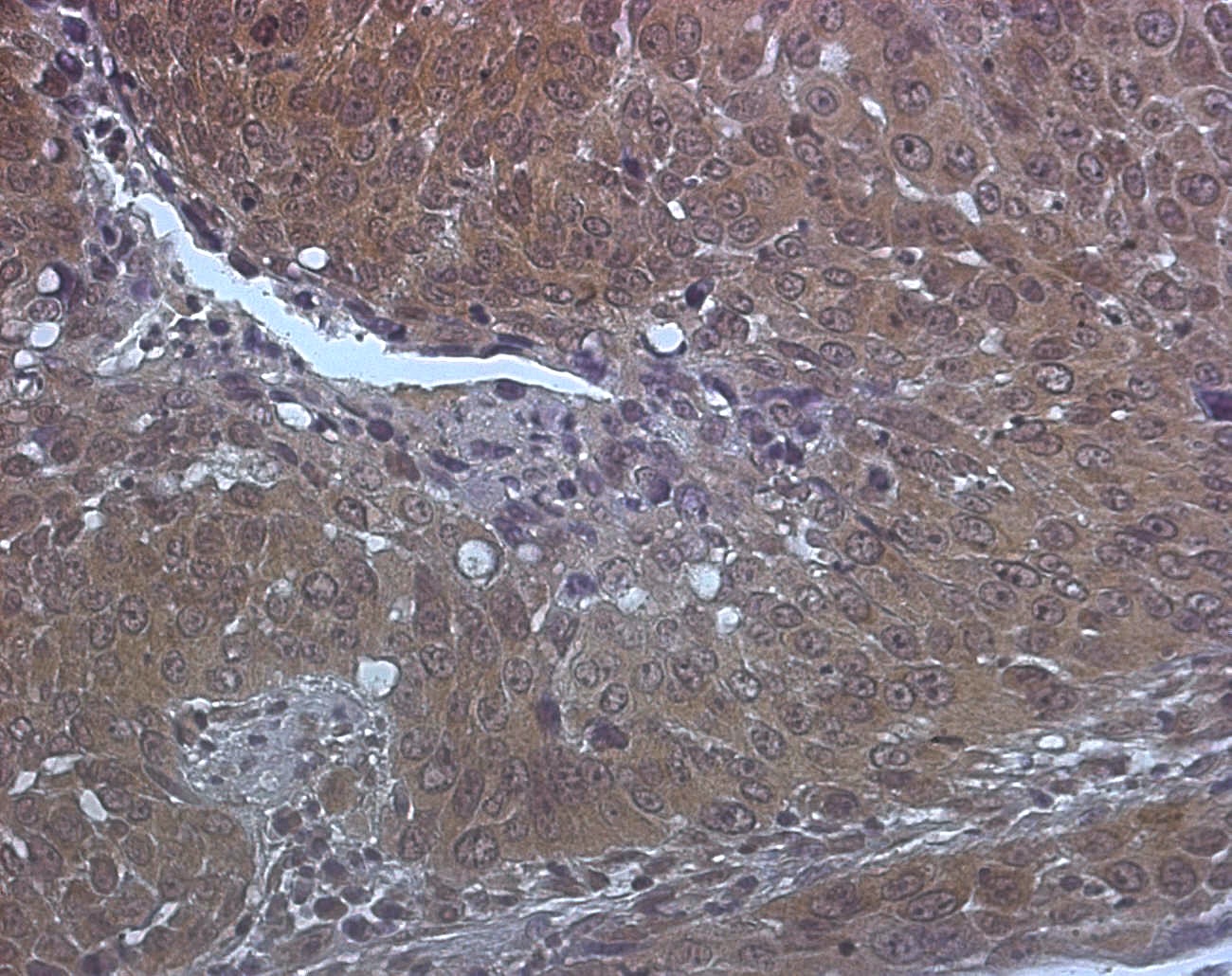

Supplement: S1 File — (ZIP) [file pone.0349359.s001.zip › Figure 1A pAKT right SCC 40x.jpg]

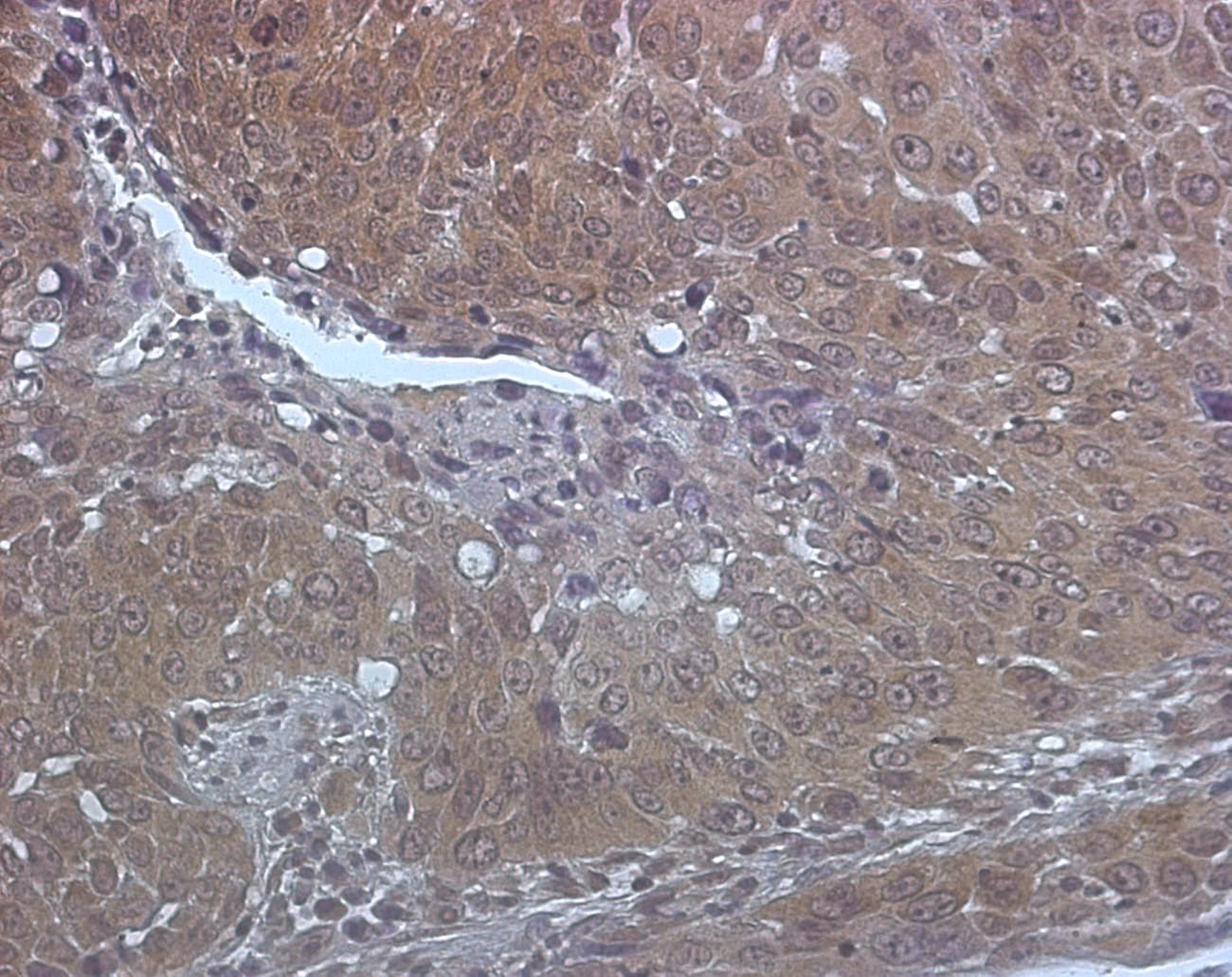

Supplement: S1 File — (ZIP) [file pone.0349359.s001.zip › Figure 1A pAKT right SCC 40x.pdf]

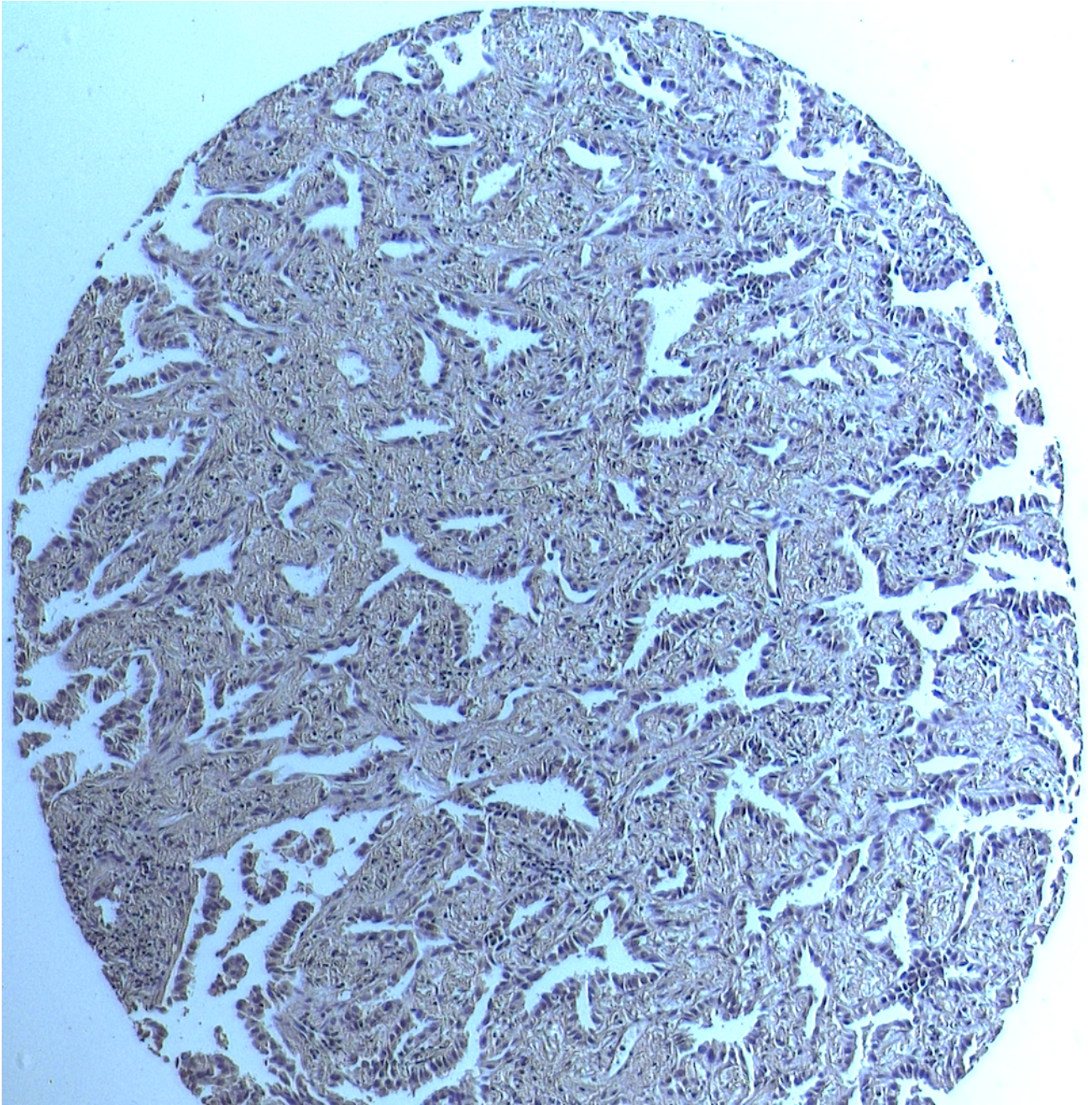

Supplement: S1 File — (ZIP) [file pone.0349359.s001.zip › Figure 1B pAKT left ADC 10x.pdf]

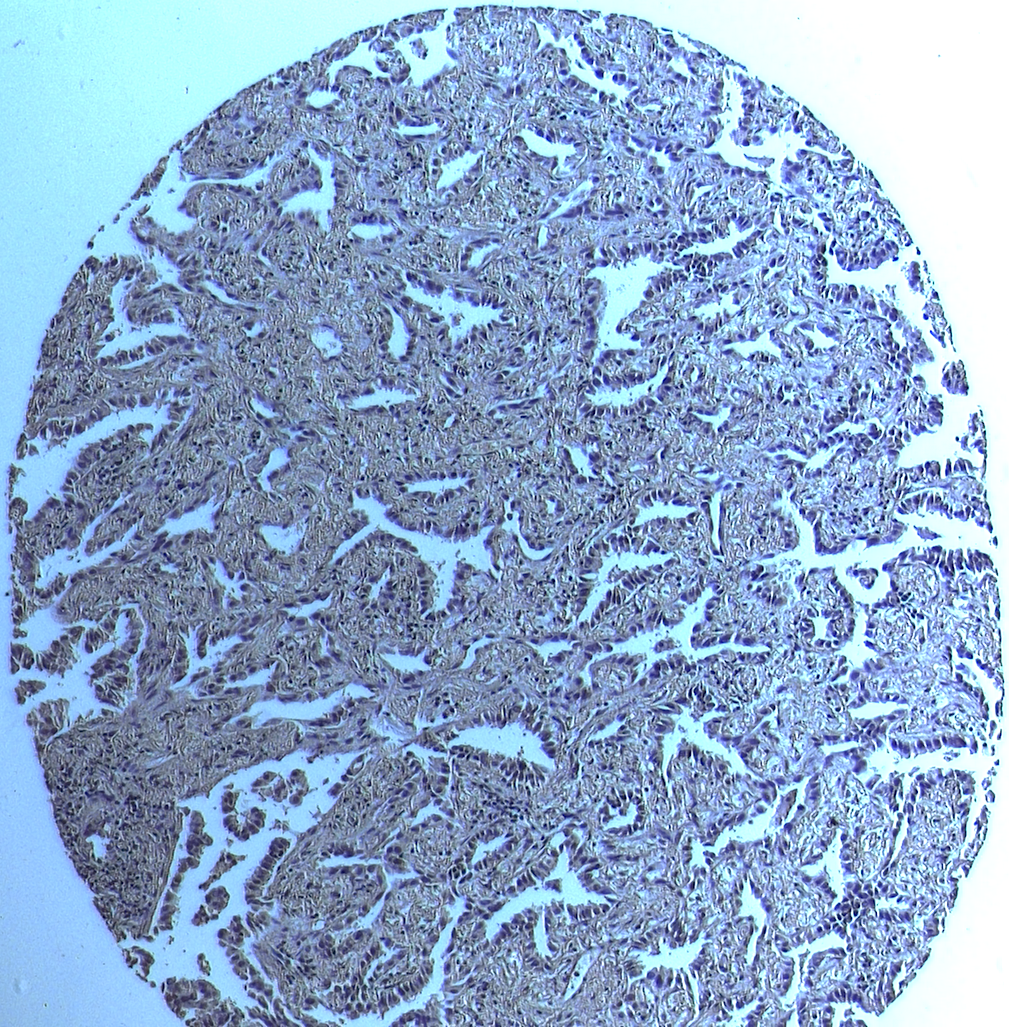

Supplement: S1 File — (ZIP) [file pone.0349359.s001.zip › Figure 1B pAKT left ADC 10x.tif]

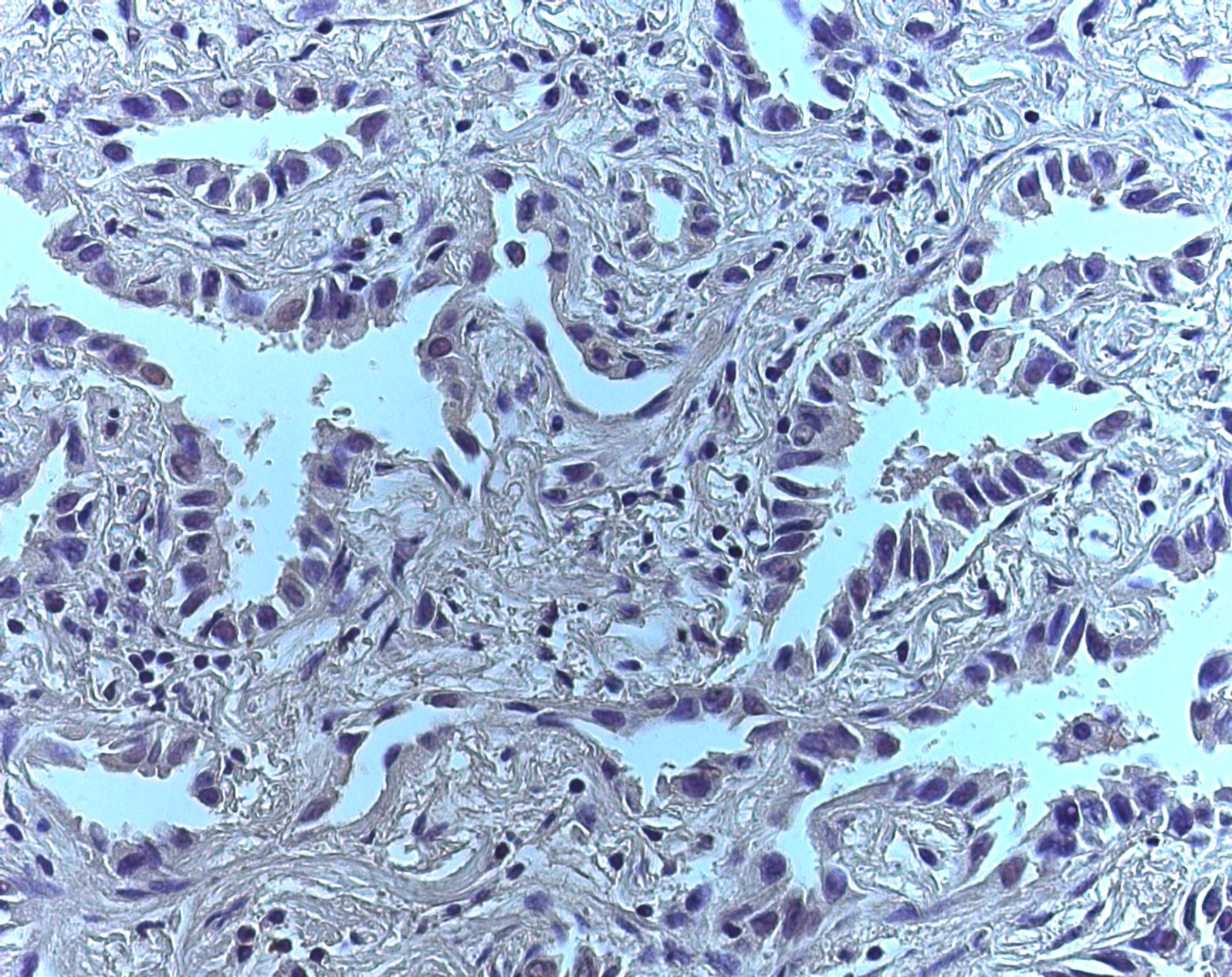

Supplement: S1 File — (ZIP) [file pone.0349359.s001.zip › Figure 1B pAKT left ADC40x.pdf]

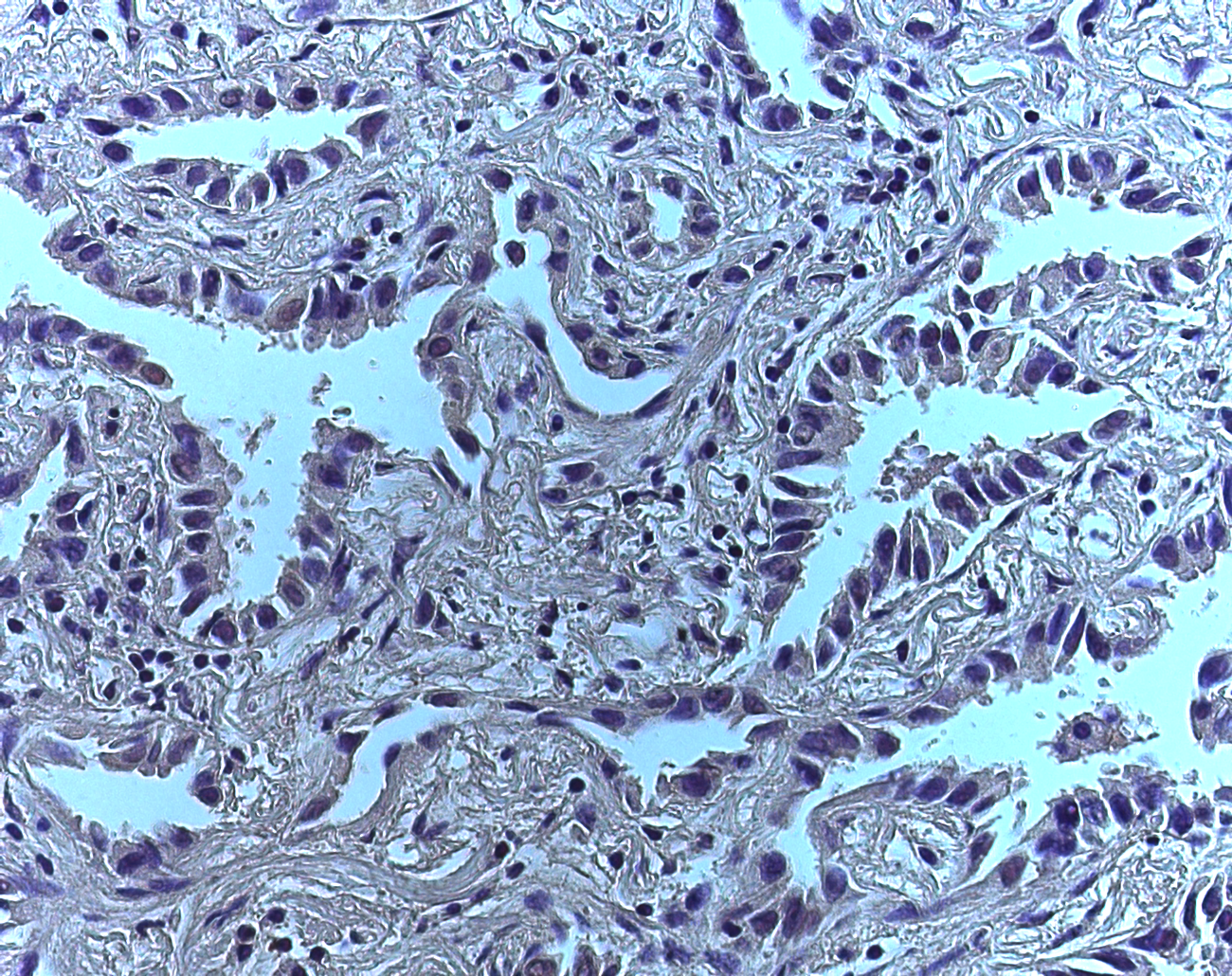

Supplement: S1 File — (ZIP) [file pone.0349359.s001.zip › Figure 1B pAKT left ADC40x.tif]

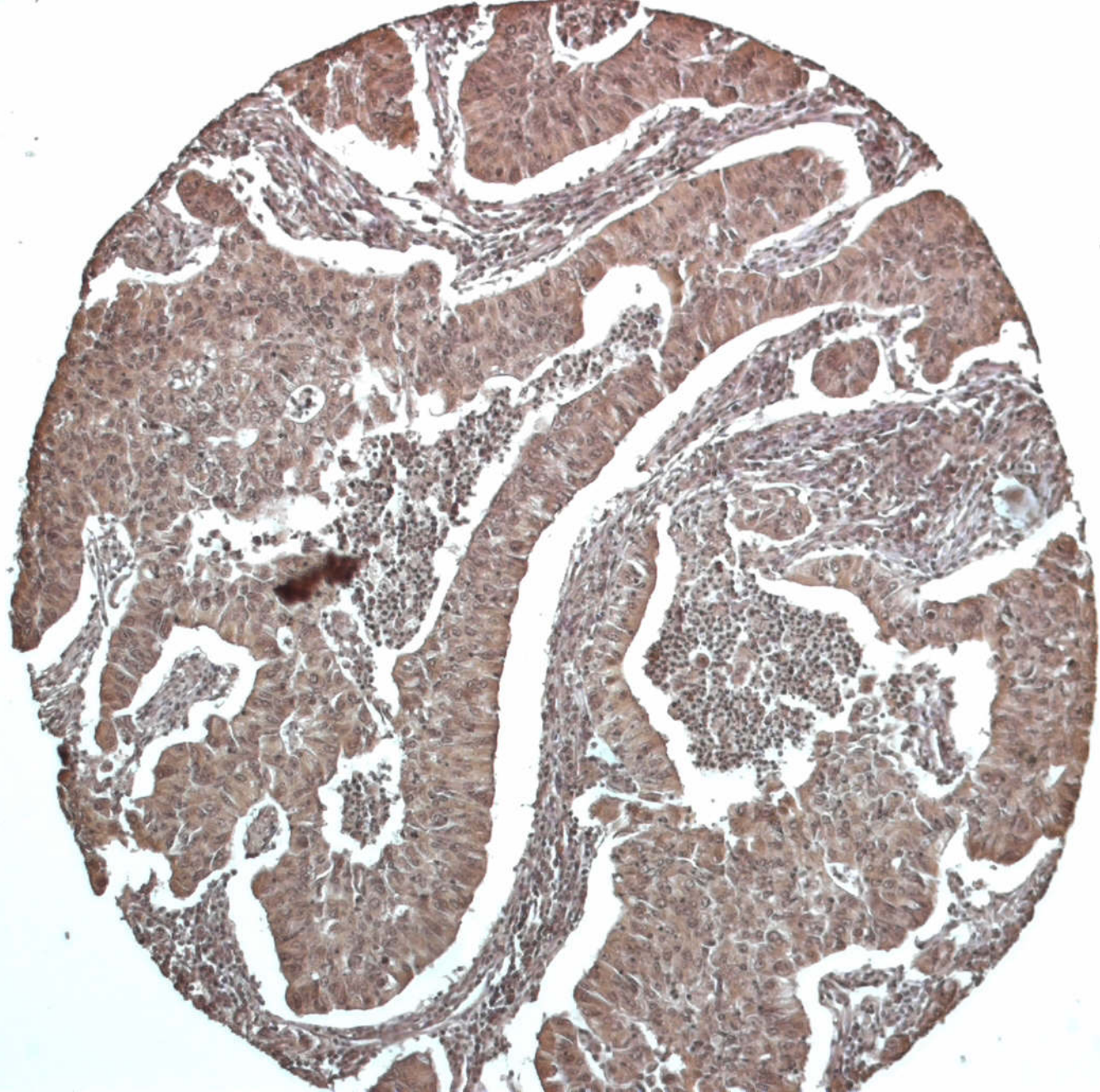

Supplement: S1 File — (ZIP) [file pone.0349359.s001.zip › Figure 1B pAKT right ADC 10x .pdf]

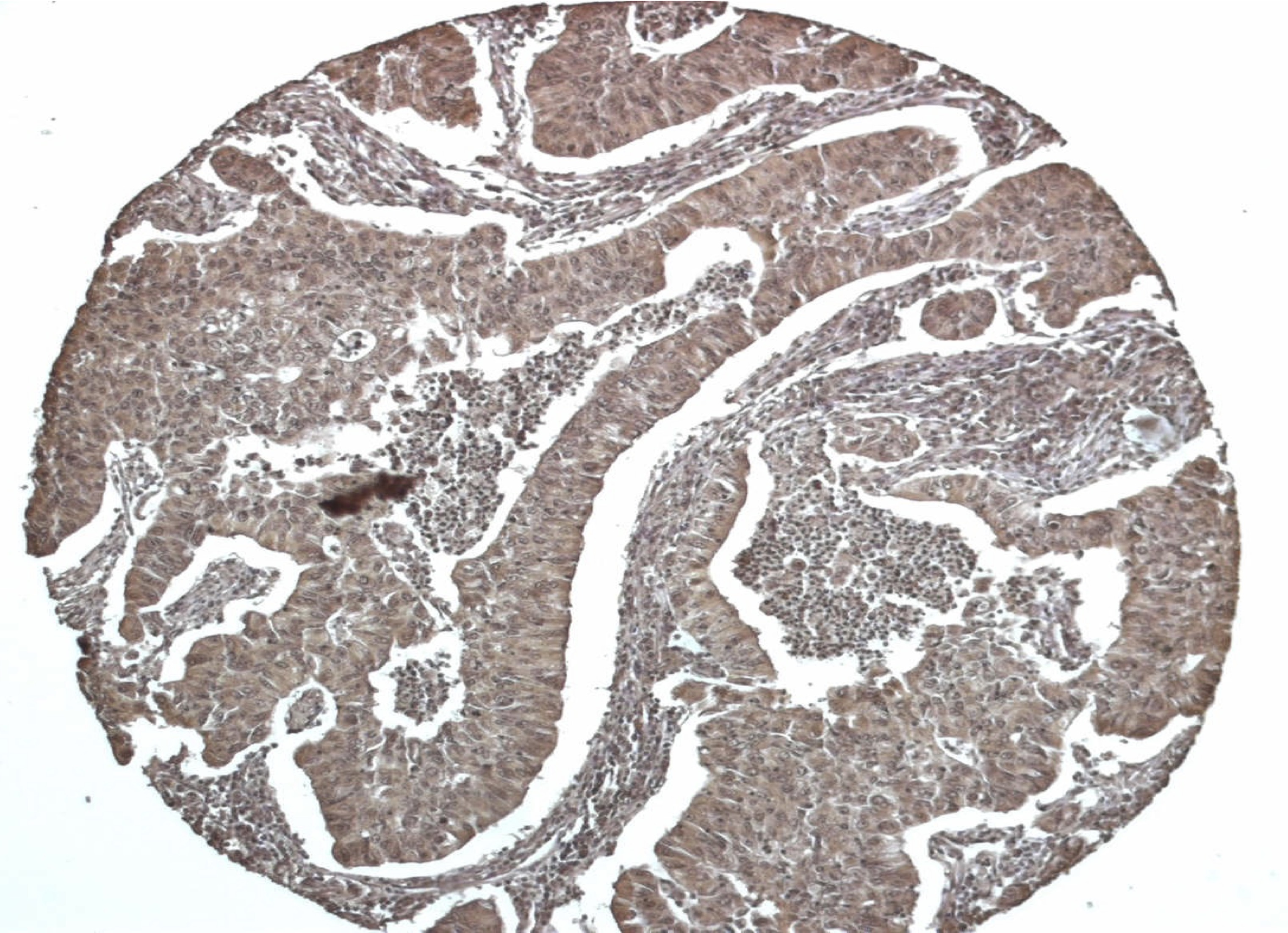

Supplement: S1 File — (ZIP) [file pone.0349359.s001.zip › Figure 1B pAKT right ADC 10x.jpg]

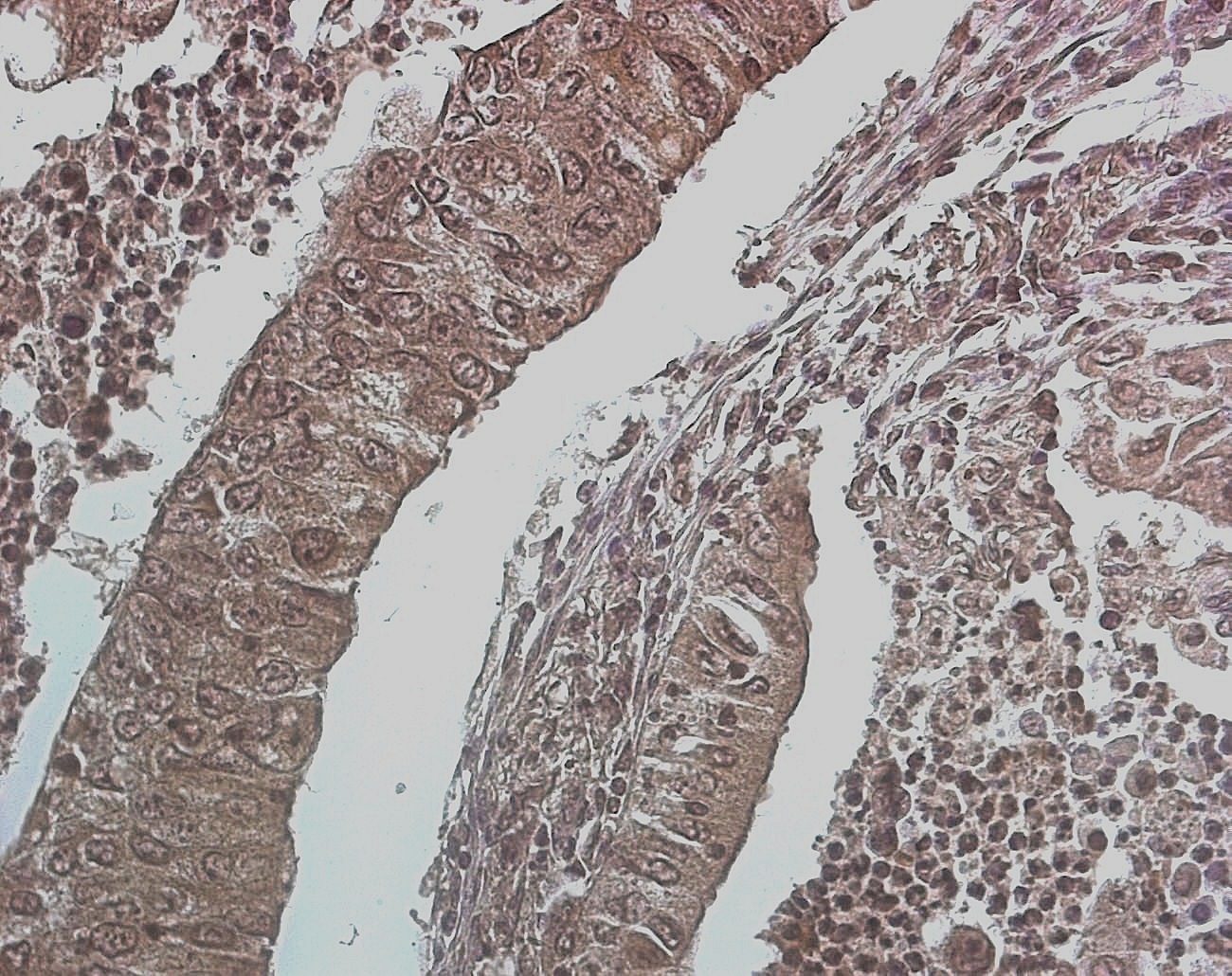

Supplement: S1 File — (ZIP) [file pone.0349359.s001.zip › Figure 1B pAKT right ADC 40x.jpg]

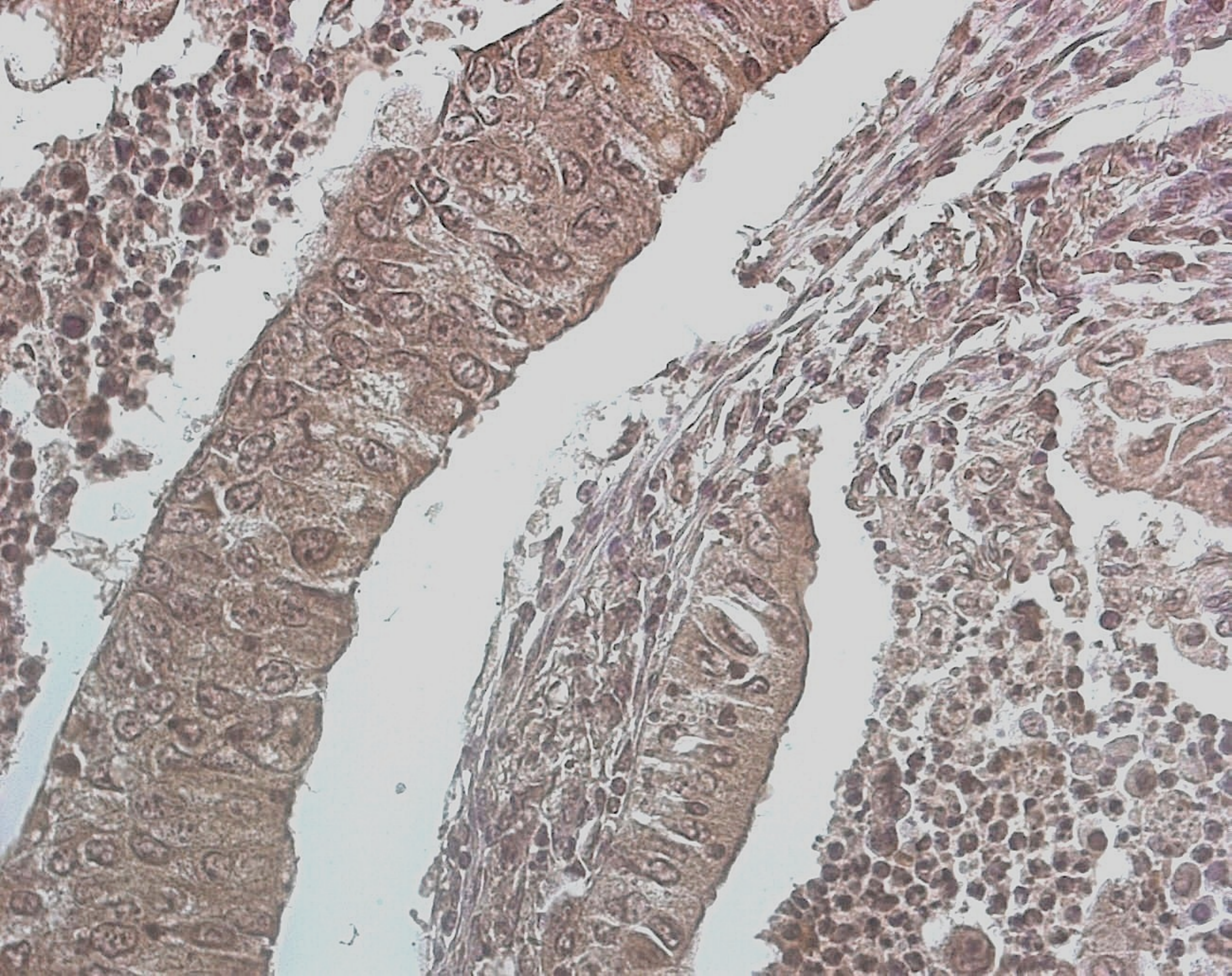

Supplement: S1 File — (ZIP) [file pone.0349359.s001.zip › Figure 1B pAKT right ADC 40x.pdf]

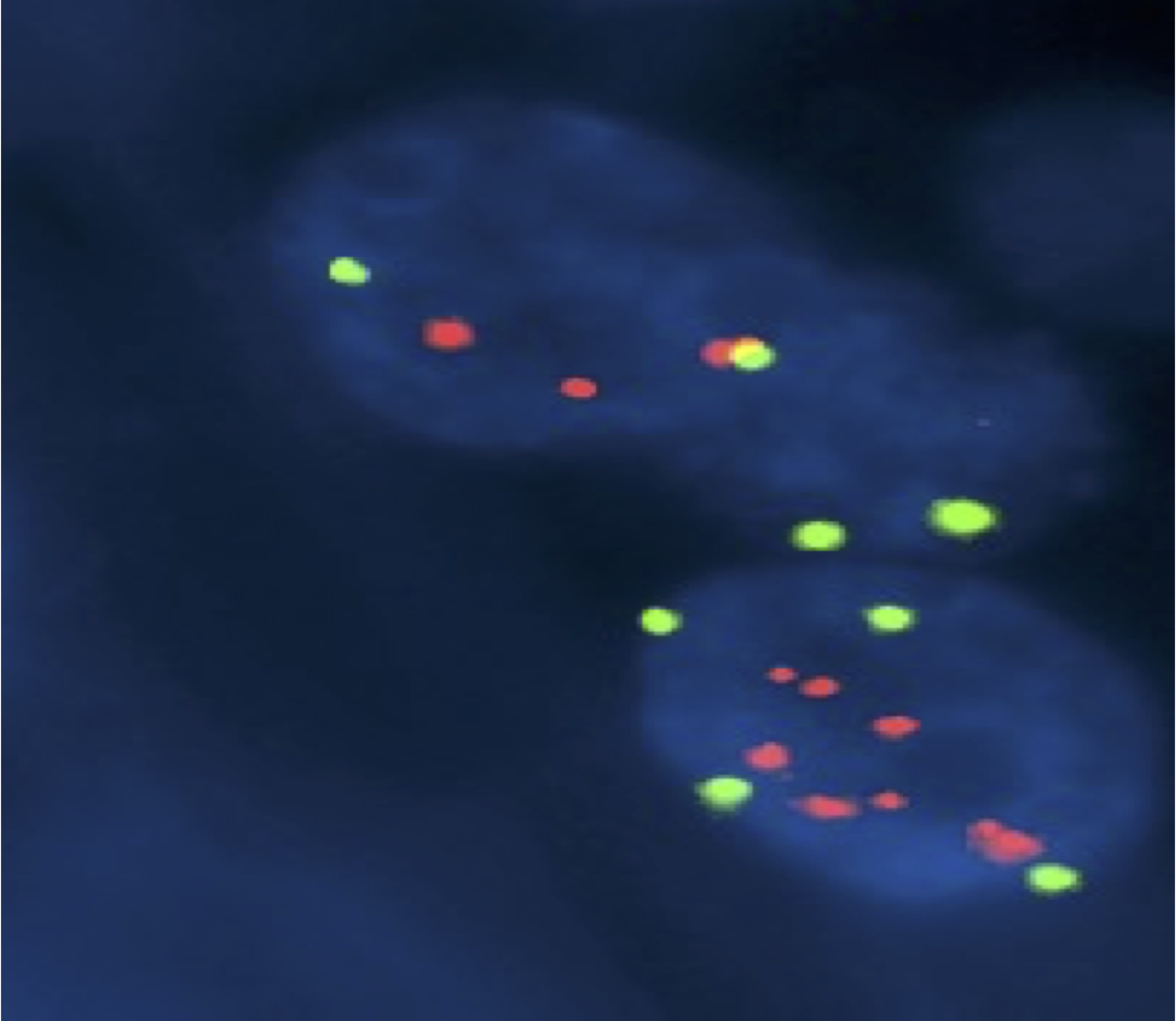

Supplement: S2 File — (ZIP) [file pone.0349359.s002.zip › Figure 2C AKT1 right .tiff]

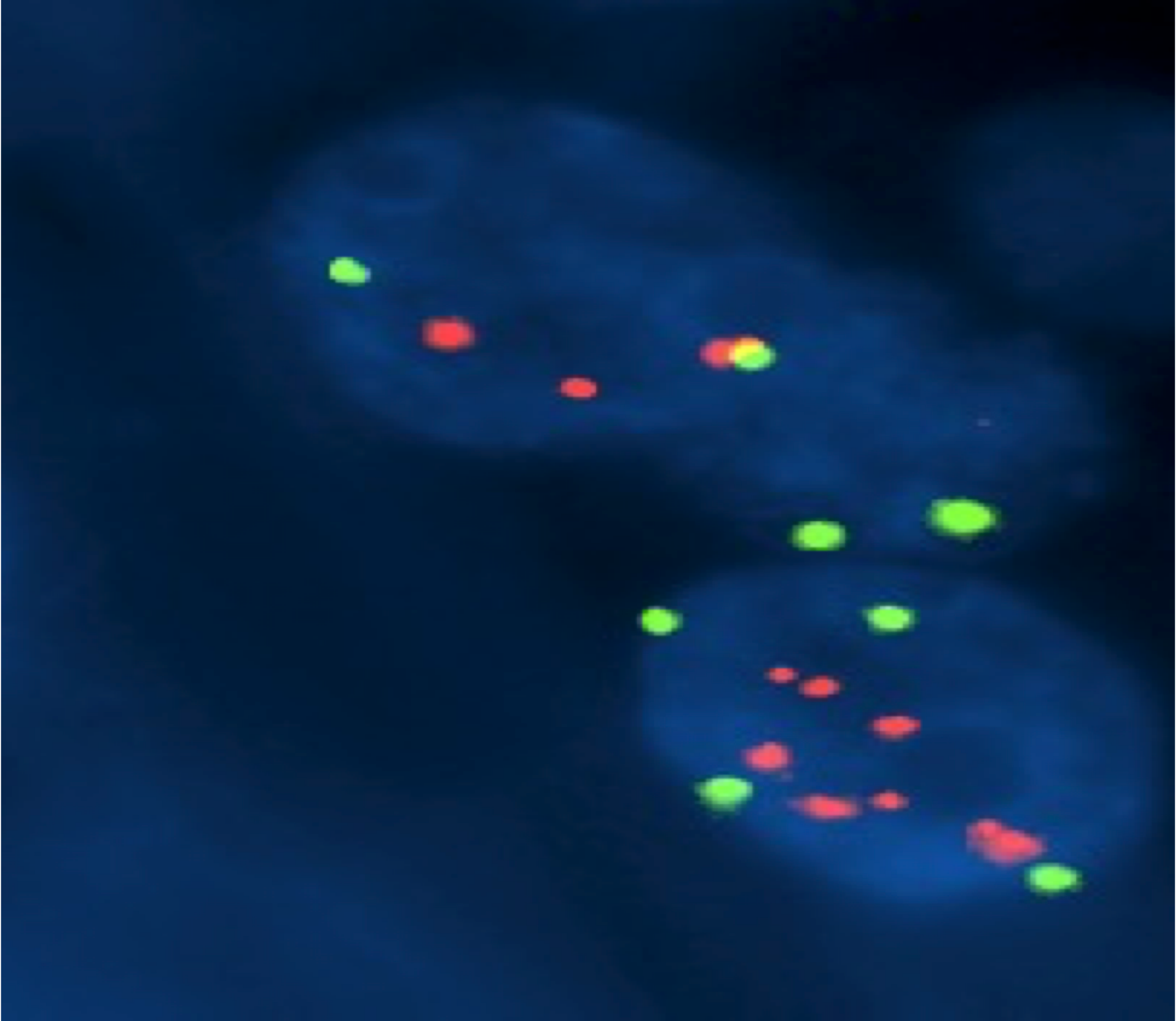

Supplement: S2 File — (ZIP) [file pone.0349359.s002.zip › Figure 2C AKT1 right.pdf]

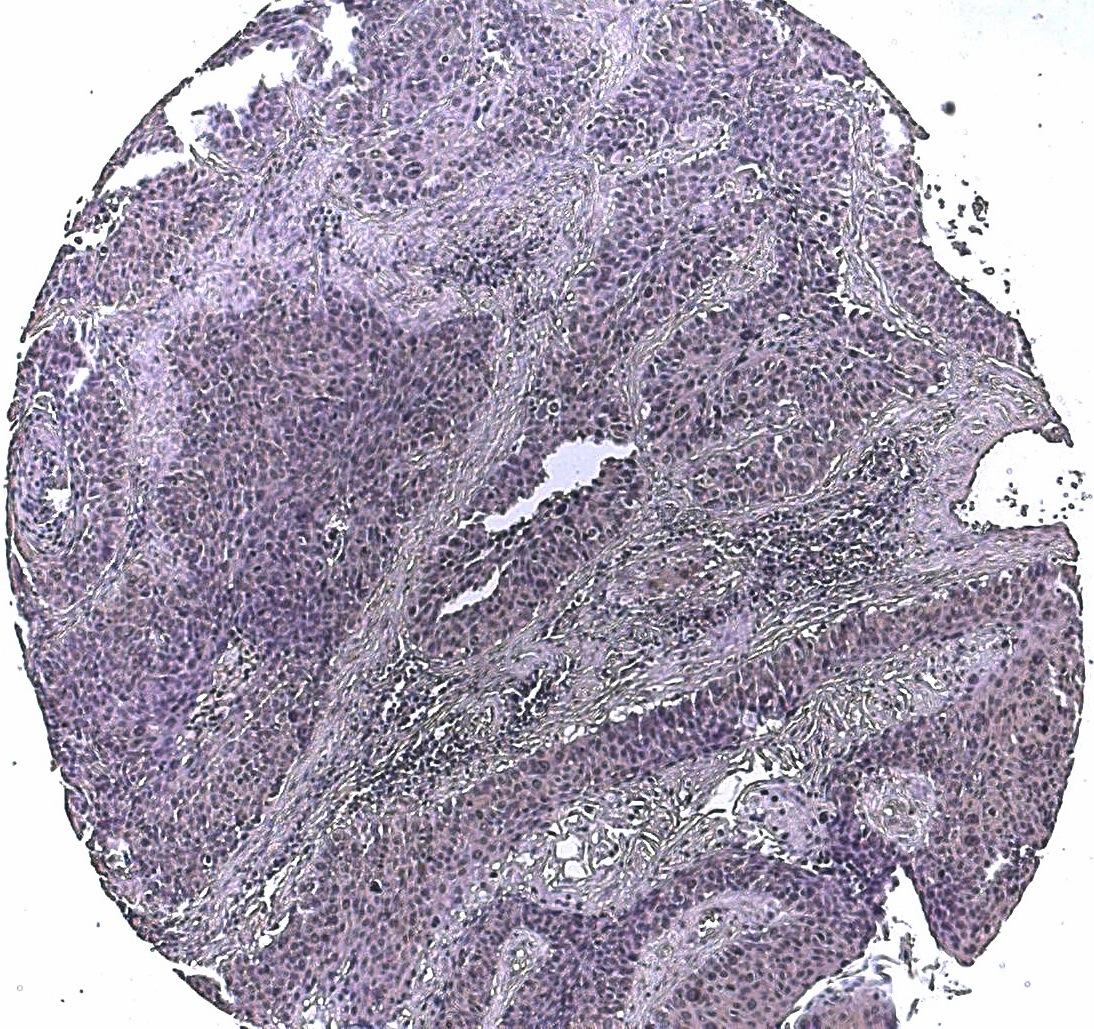

Supplement: S2 File — (ZIP) [file pone.0349359.s002.zip › Figure 2A AKT1 SCC left 10x.jpg]

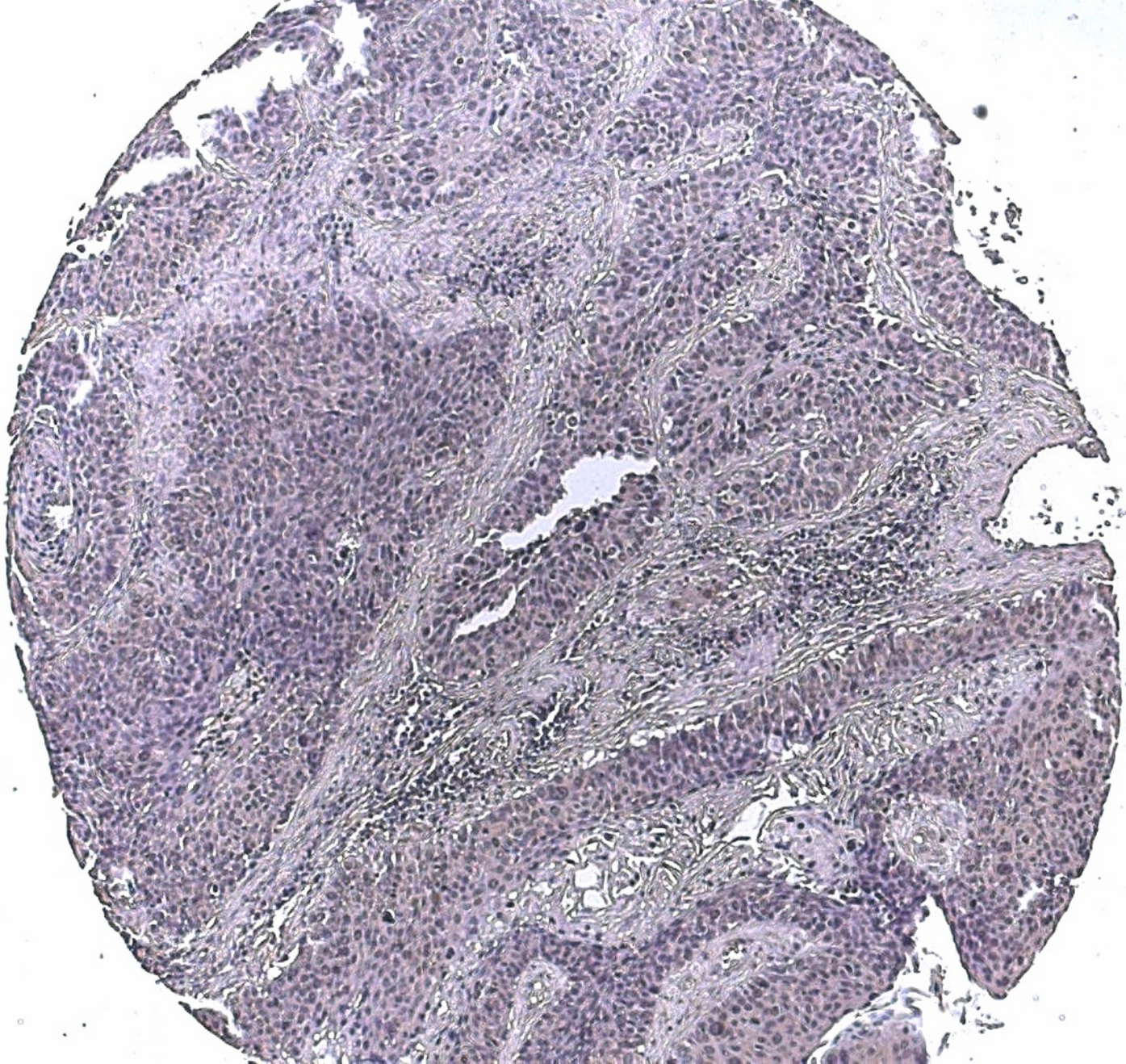

Supplement: S2 File — (ZIP) [file pone.0349359.s002.zip › Figure 2A AKT1 SCC left 10x.pdf]

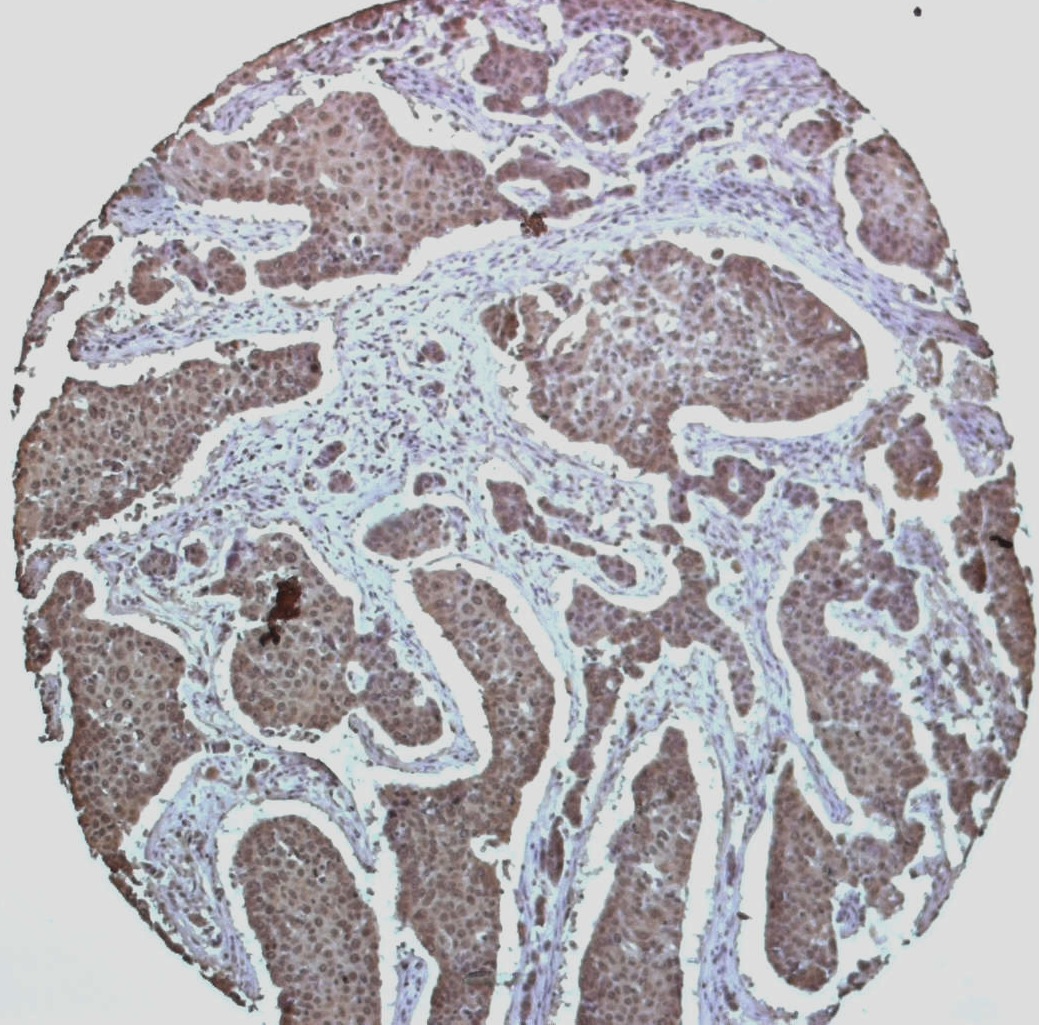

Supplement: S2 File — (ZIP) [file pone.0349359.s002.zip › Figure 2A AKT1 SCC right 10x.jpg]

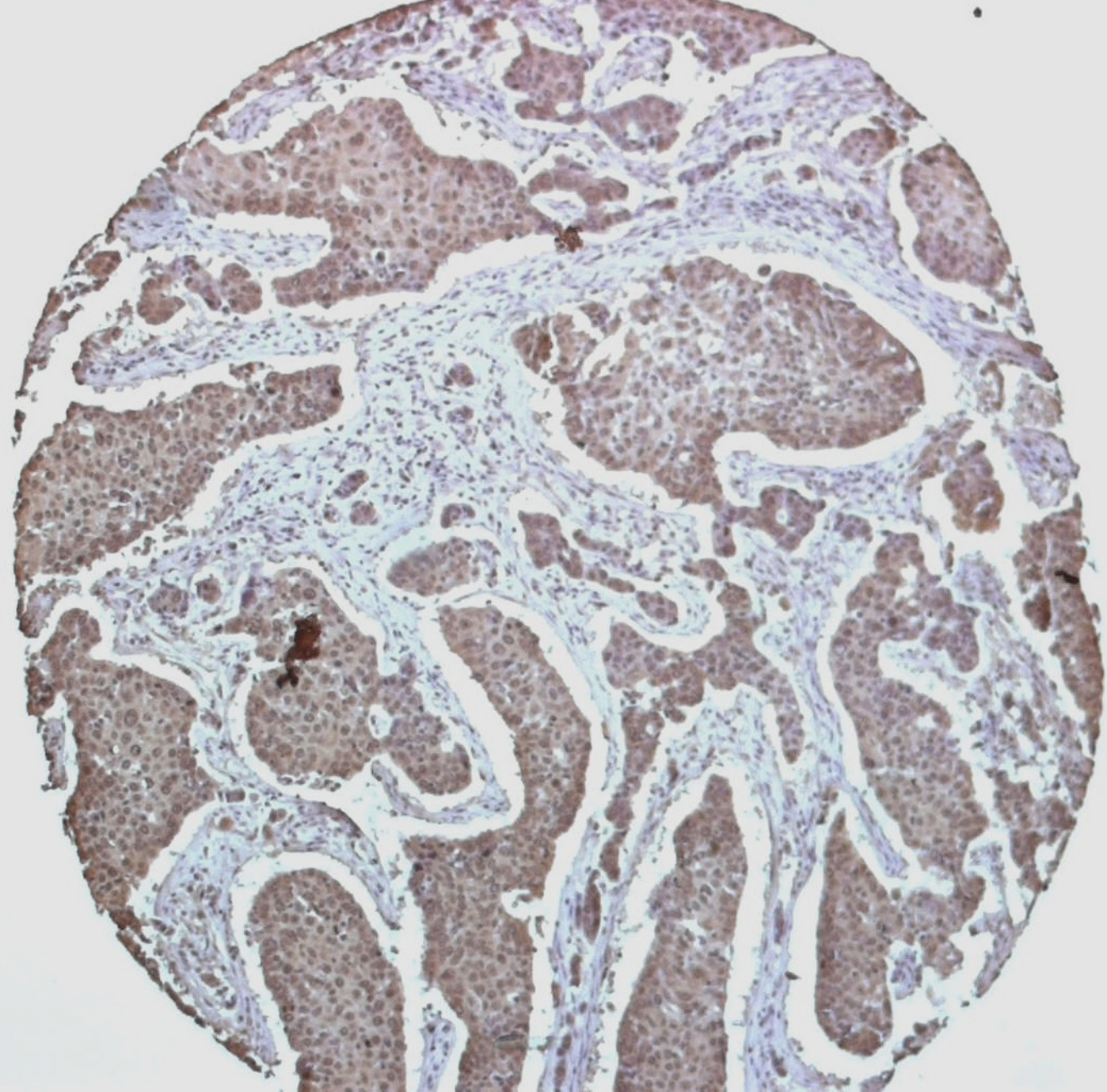

Supplement: S2 File — (ZIP) [file pone.0349359.s002.zip › Figure 2A AKT1 SCC right 10x.pdf]

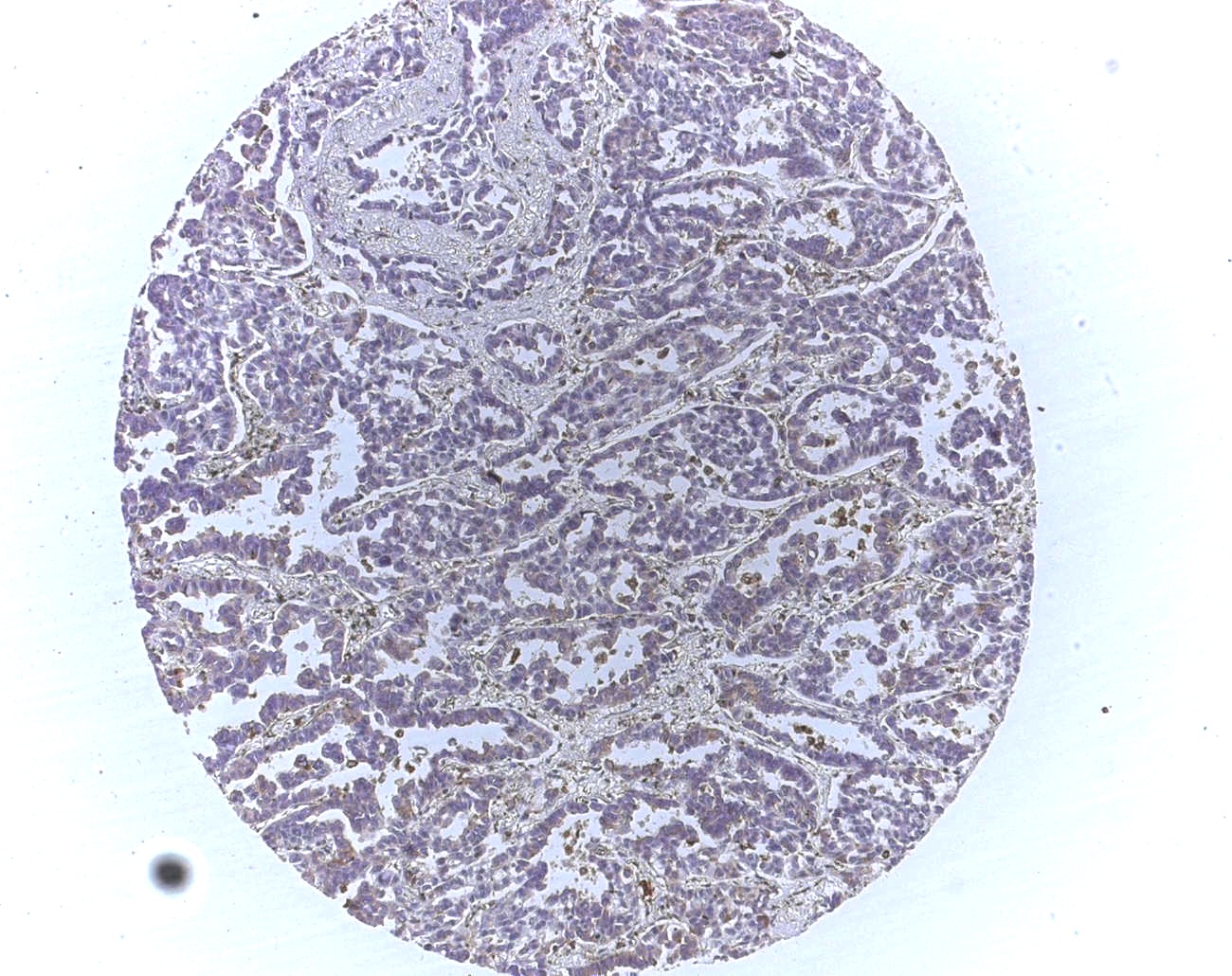

Supplement: S2 File — (ZIP) [file pone.0349359.s002.zip › Figure 2B AKT1 ADC left 10x.jpg]

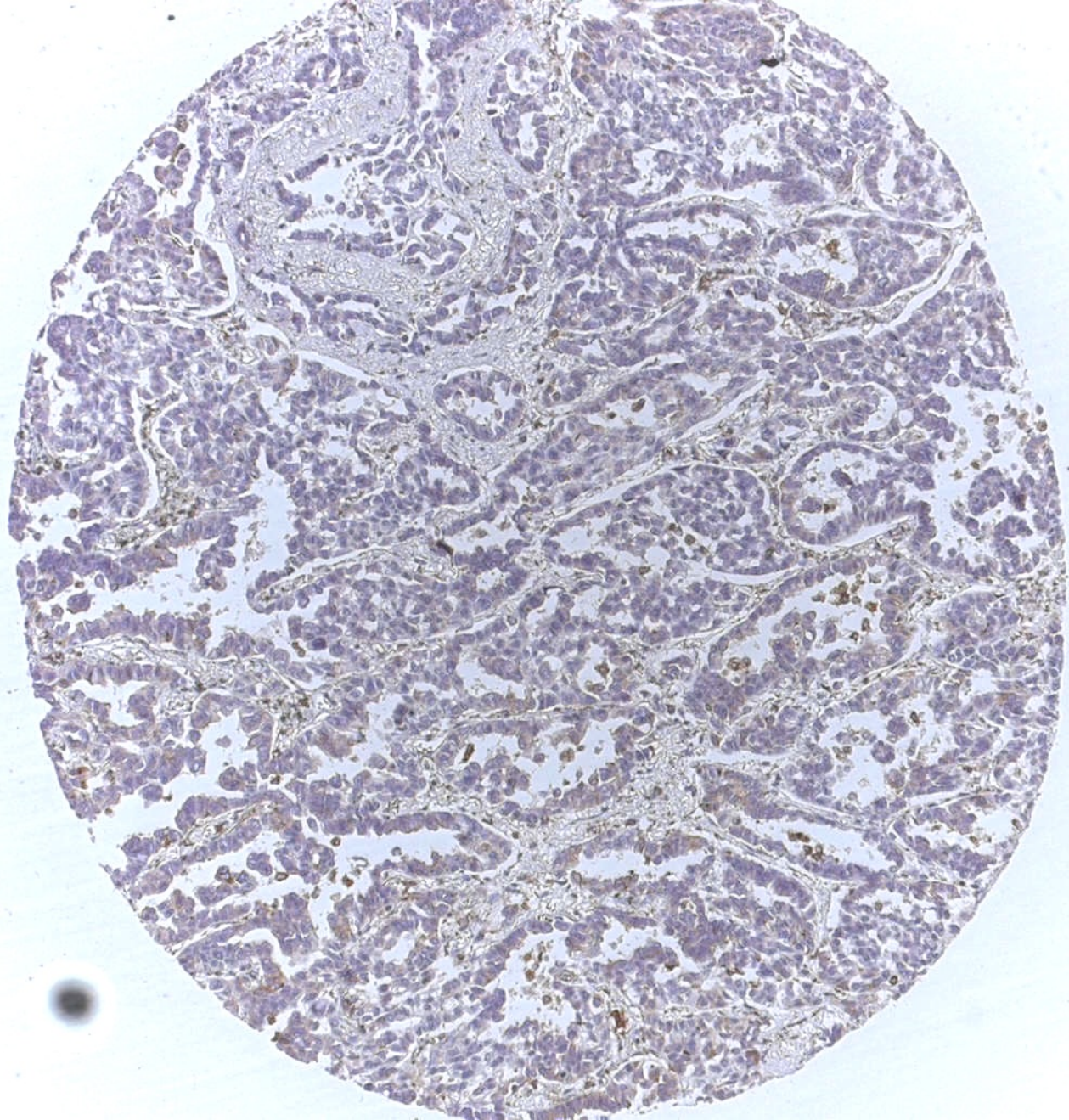

Supplement: S2 File — (ZIP) [file pone.0349359.s002.zip › Figure 2B AKT1 ADC left 10x.pdf]

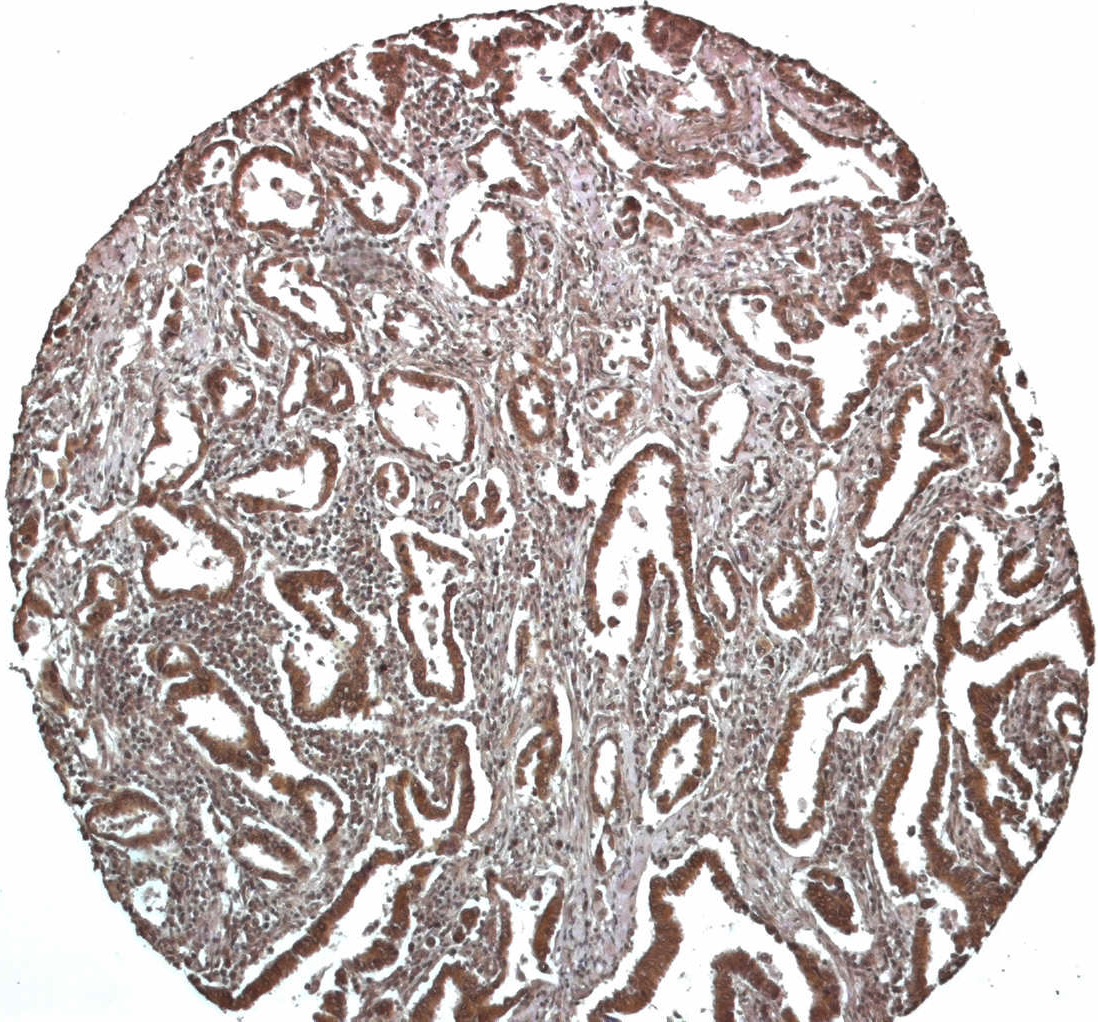

Supplement: S2 File — (ZIP) [file pone.0349359.s002.zip › Figure 2B AKT1 ADC right 10x.jpg]

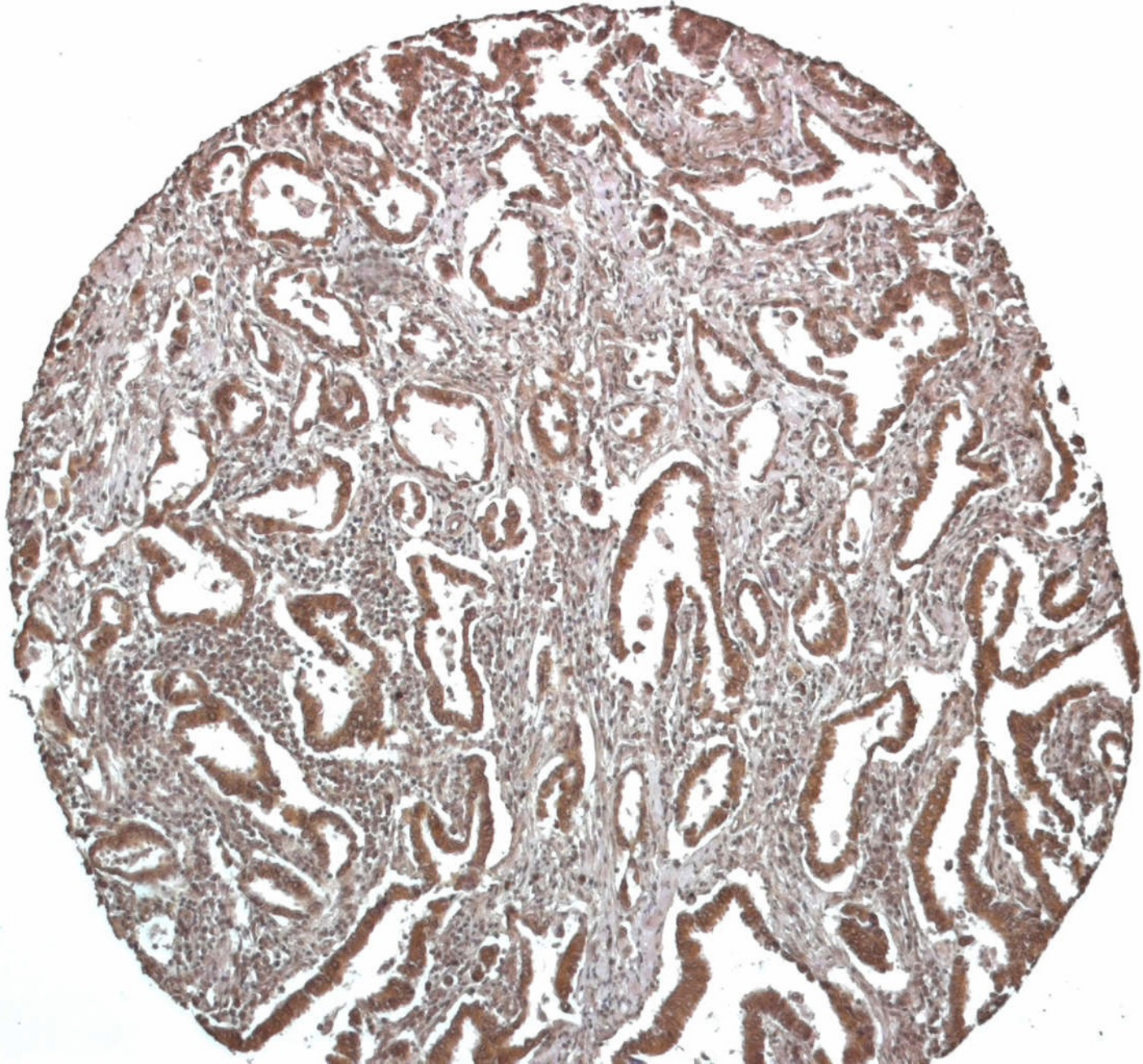

Supplement: S2 File — (ZIP) [file pone.0349359.s002.zip › Figure 2B AKT1 ADC right 10x.pdf]

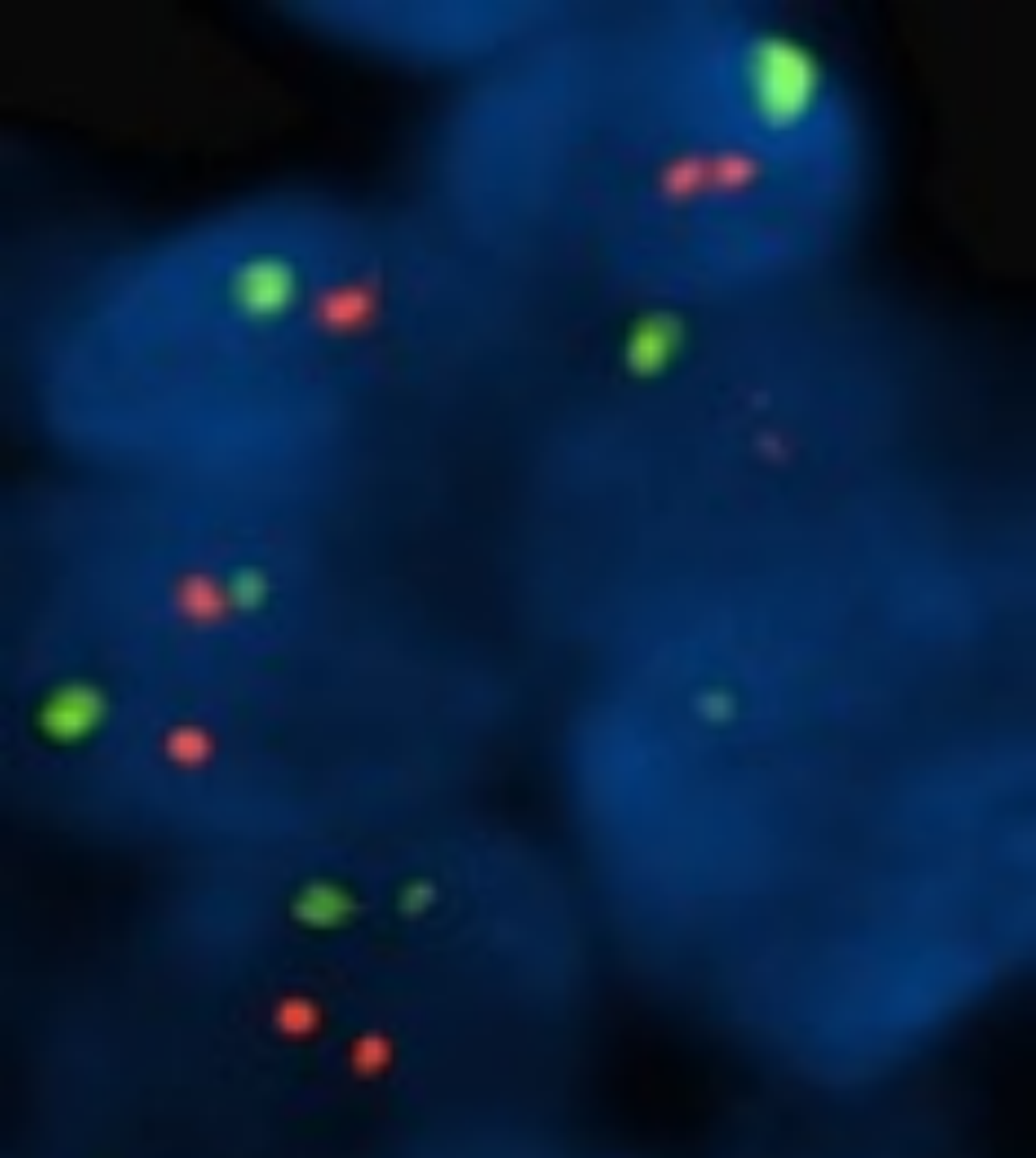

Supplement: S2 File — (ZIP) [file pone.0349359.s002.zip › Figure 2C AKT1 left.pdf]

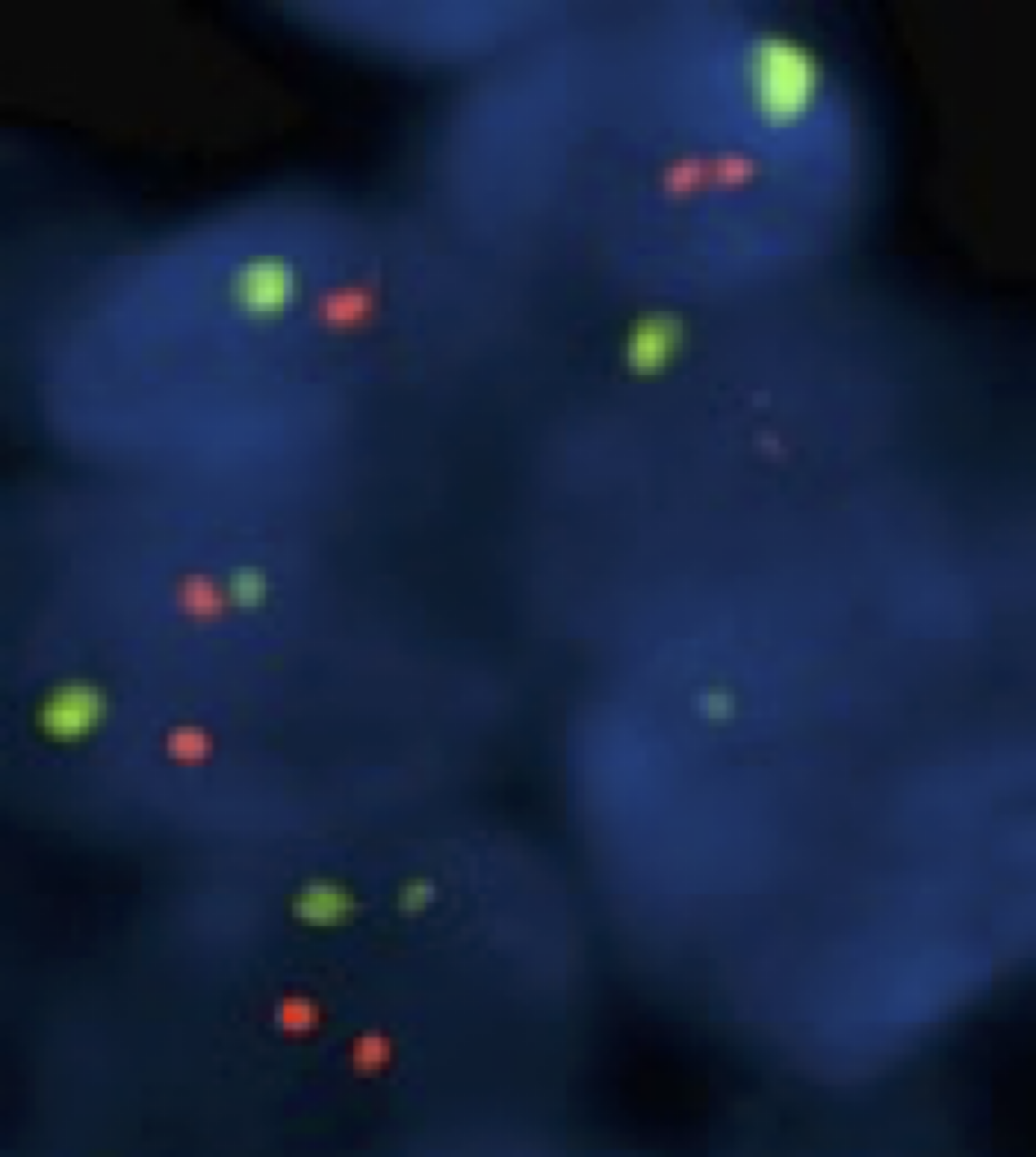

Supplement: S2 File — (ZIP) [file pone.0349359.s002.zip › Figure 2C AKT1 left.tiff]

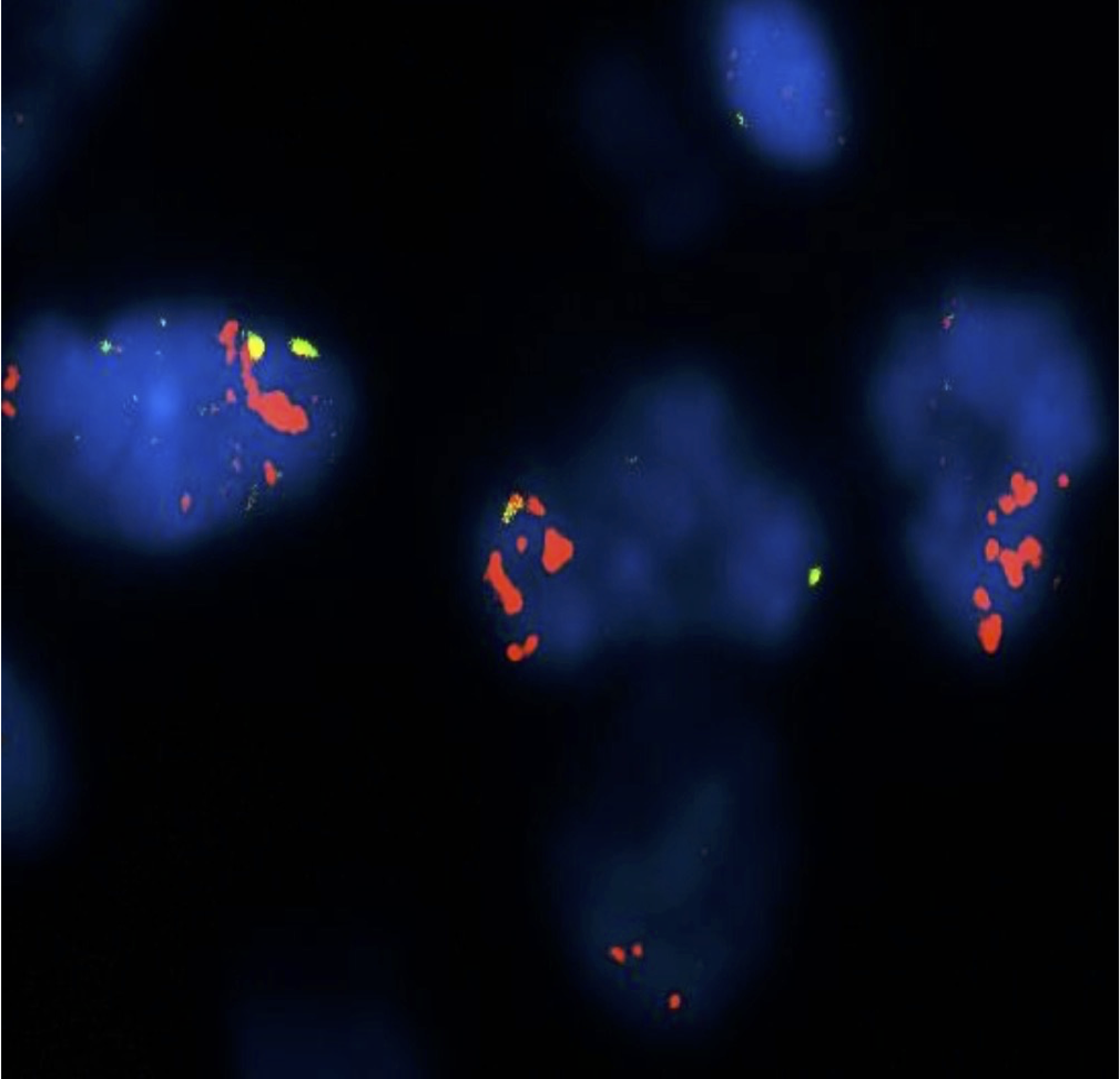

Supplement: S3 File — (ZIP) [file pone.0349359.s003.zip › Figure 3C AKT2 gene amplification right.tiff]

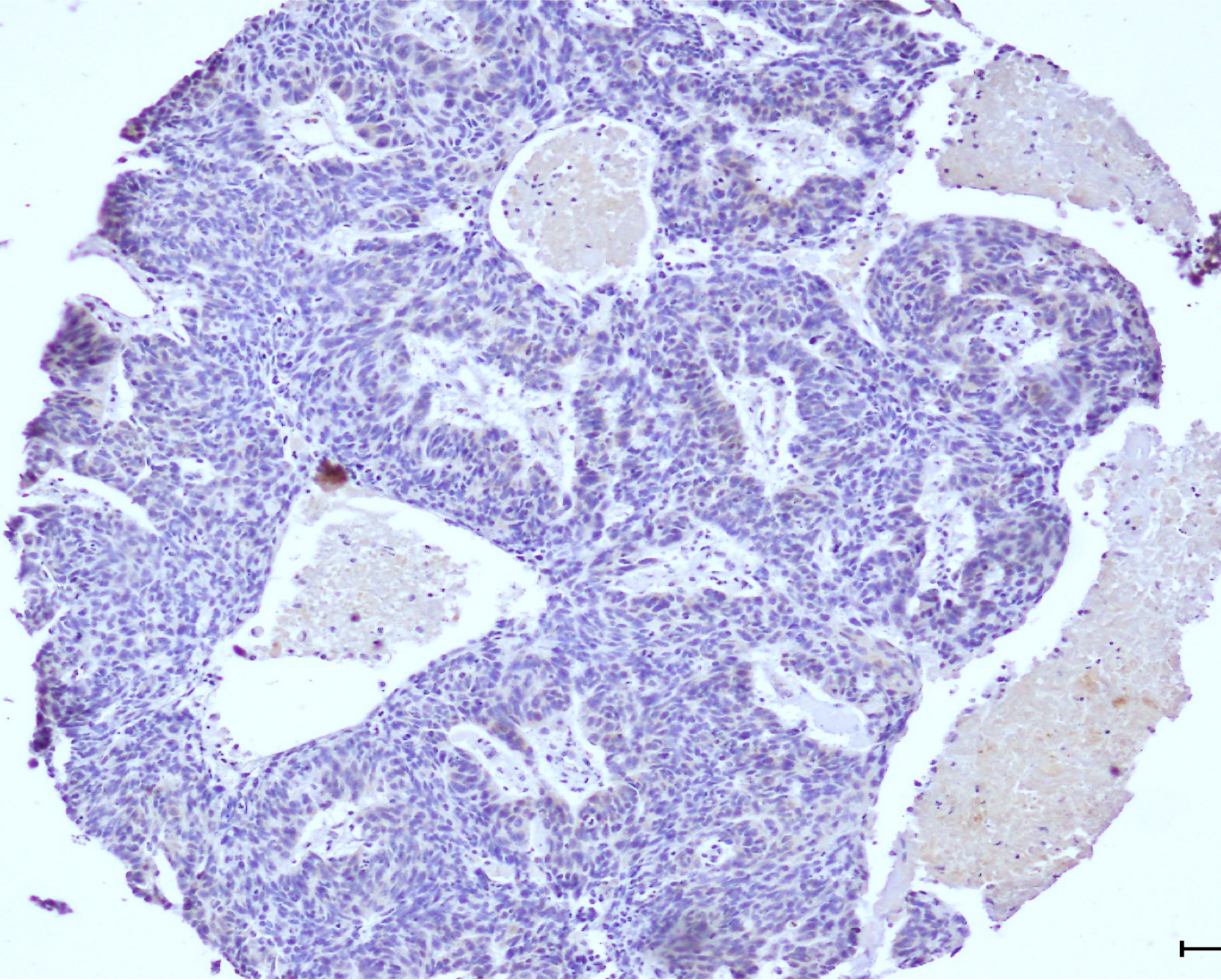

Supplement: S3 File — (ZIP) [file pone.0349359.s003.zip › Figure 3A AKT2 SCC left 10x.pdf]

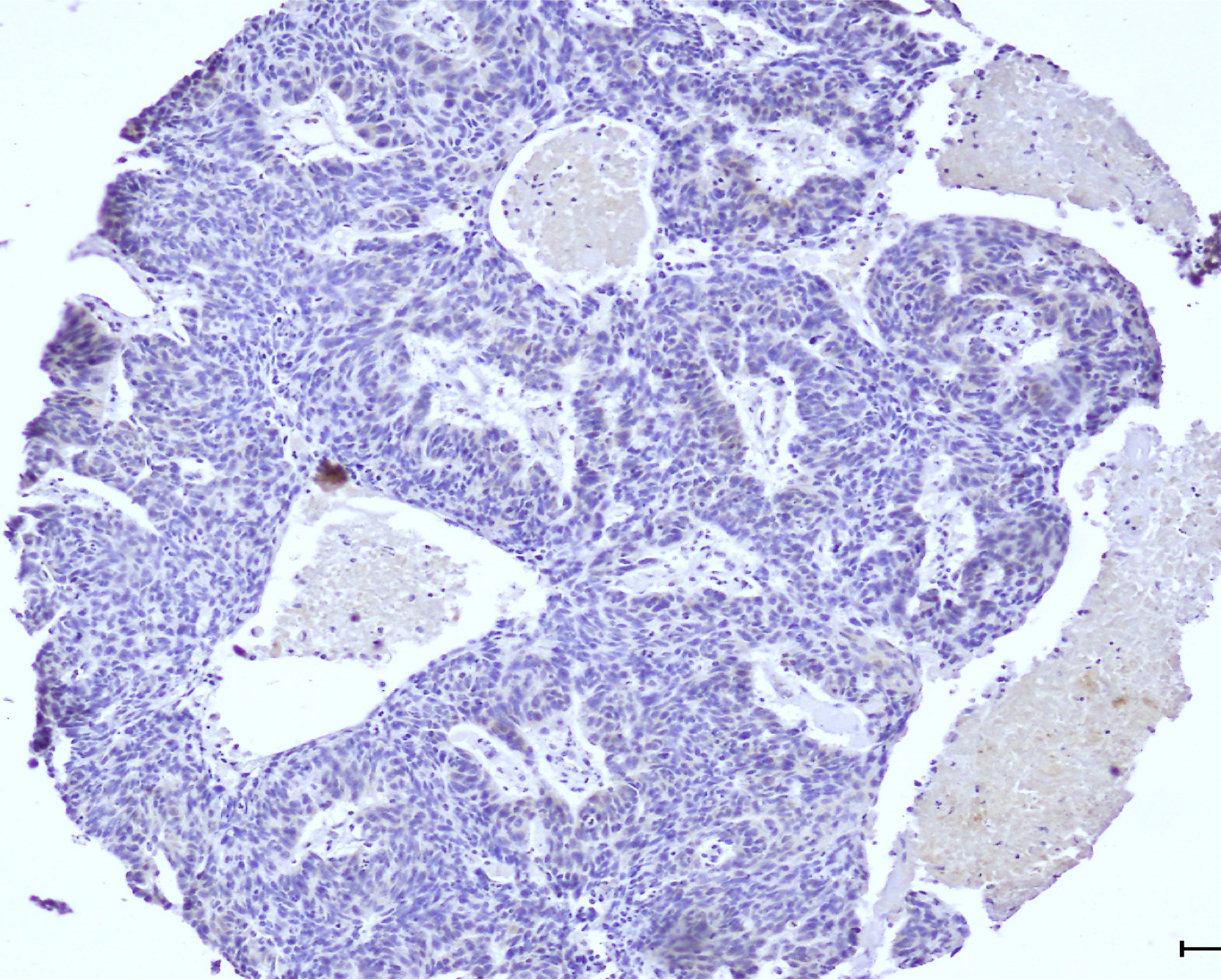

Supplement: S3 File — (ZIP) [file pone.0349359.s003.zip › Figure 3A AKT2 SCC left 10x.tiff]

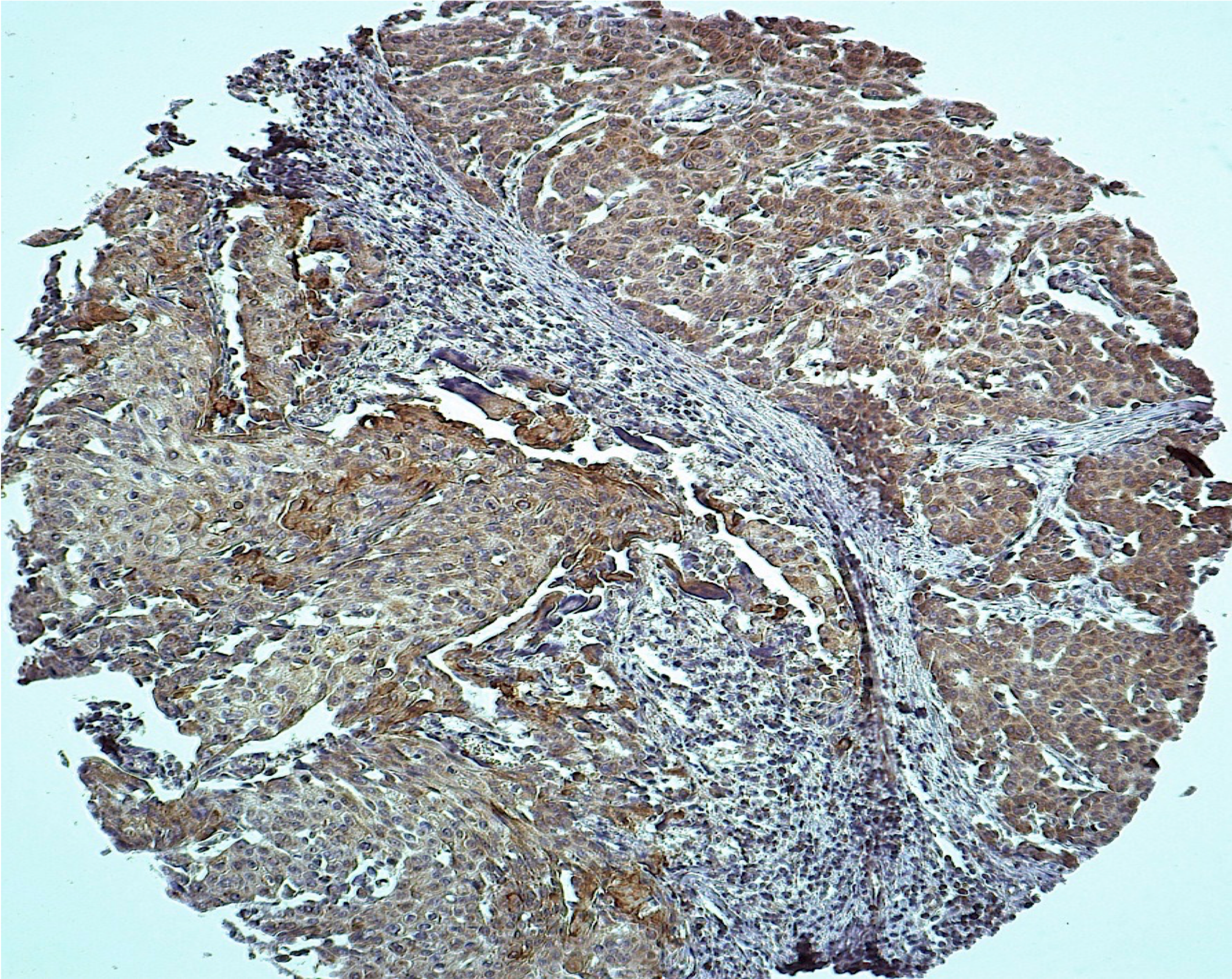

Supplement: S3 File — (ZIP) [file pone.0349359.s003.zip › Figure 3A AKT2 SCC right 10x.pdf]

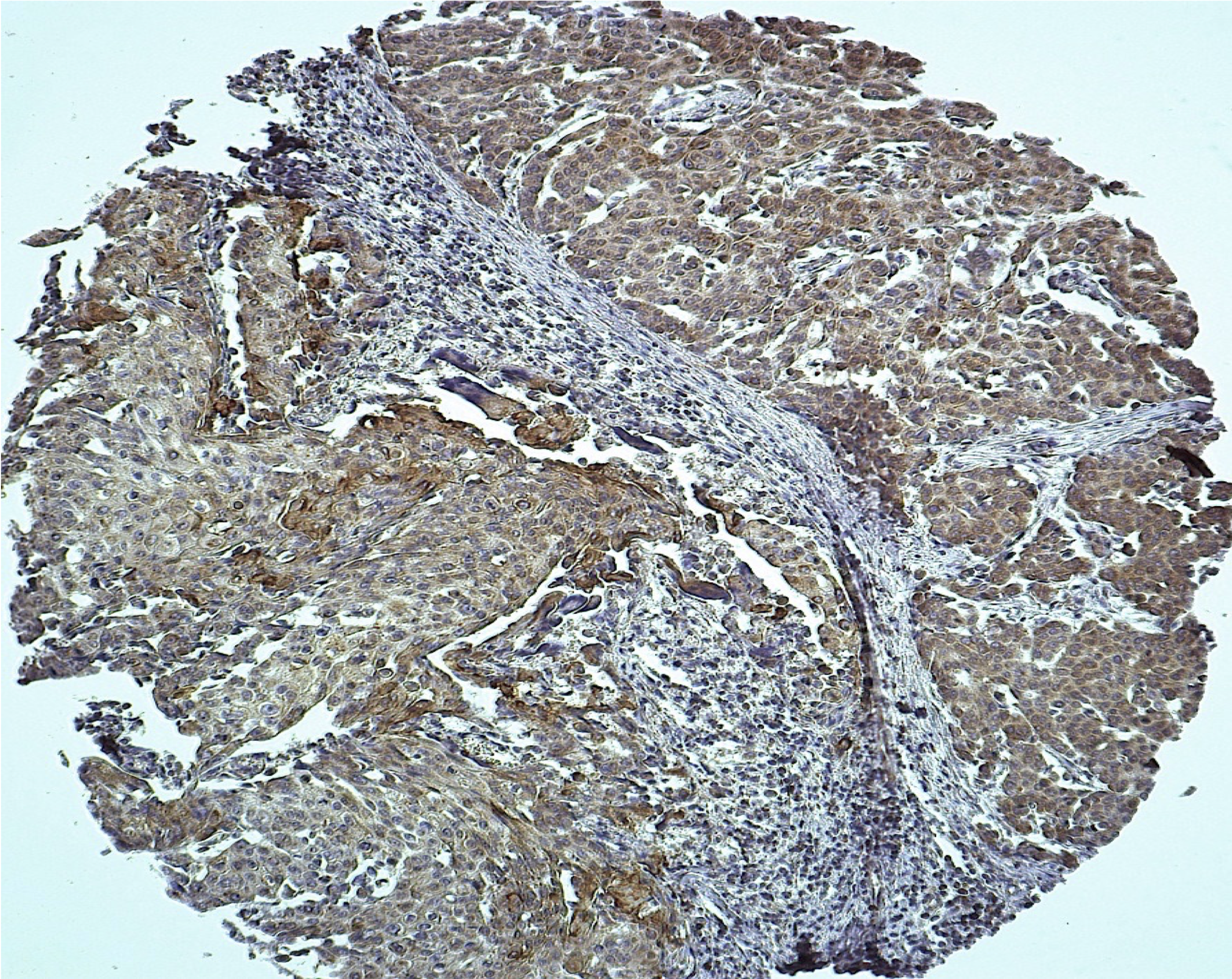

Supplement: S3 File — (ZIP) [file pone.0349359.s003.zip › Figure 3A AKT2 SCC right 10x.tiff]

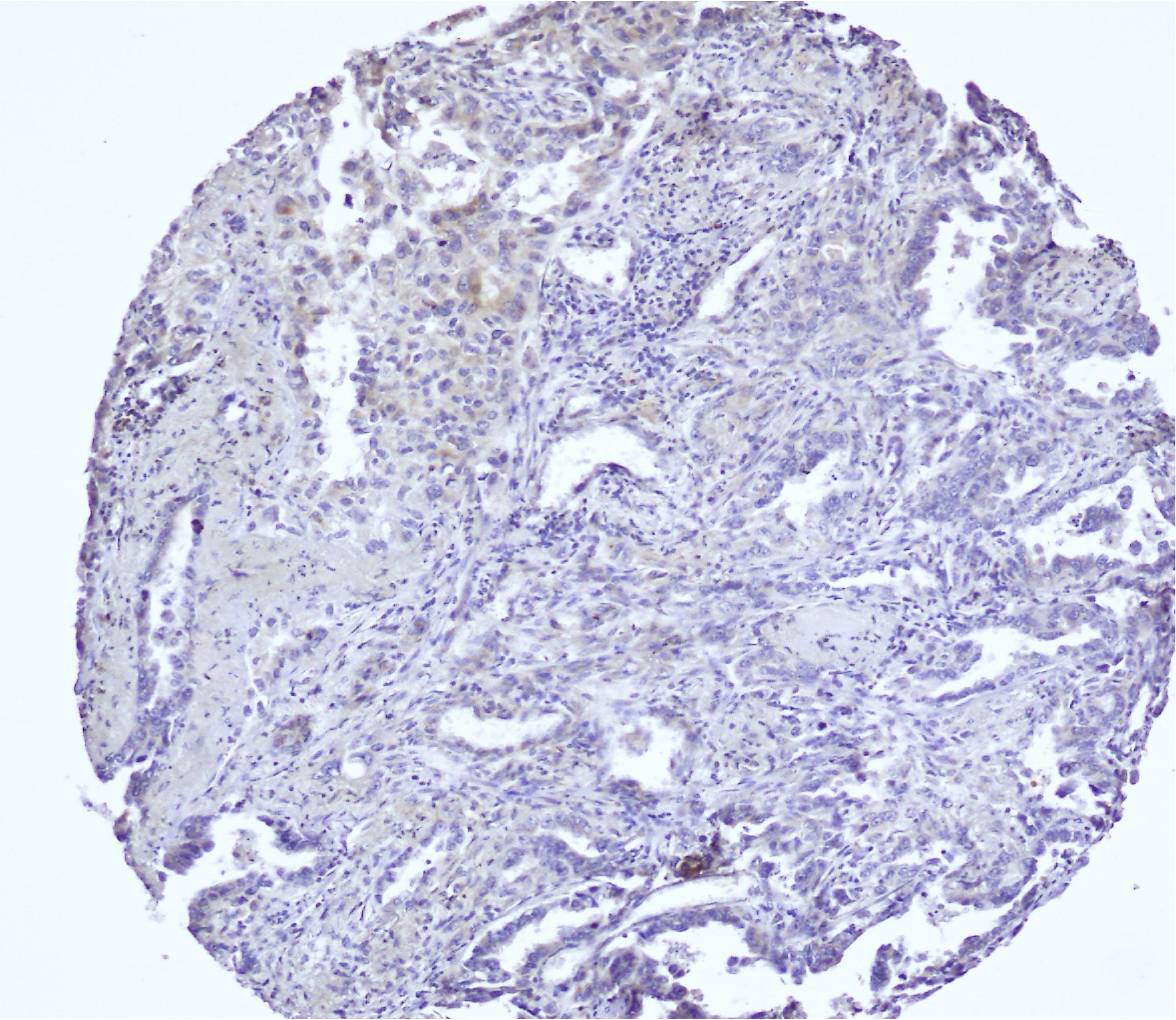

Supplement: S3 File — (ZIP) [file pone.0349359.s003.zip › Figure 3B AKT2 ADC left 10x.tiff]

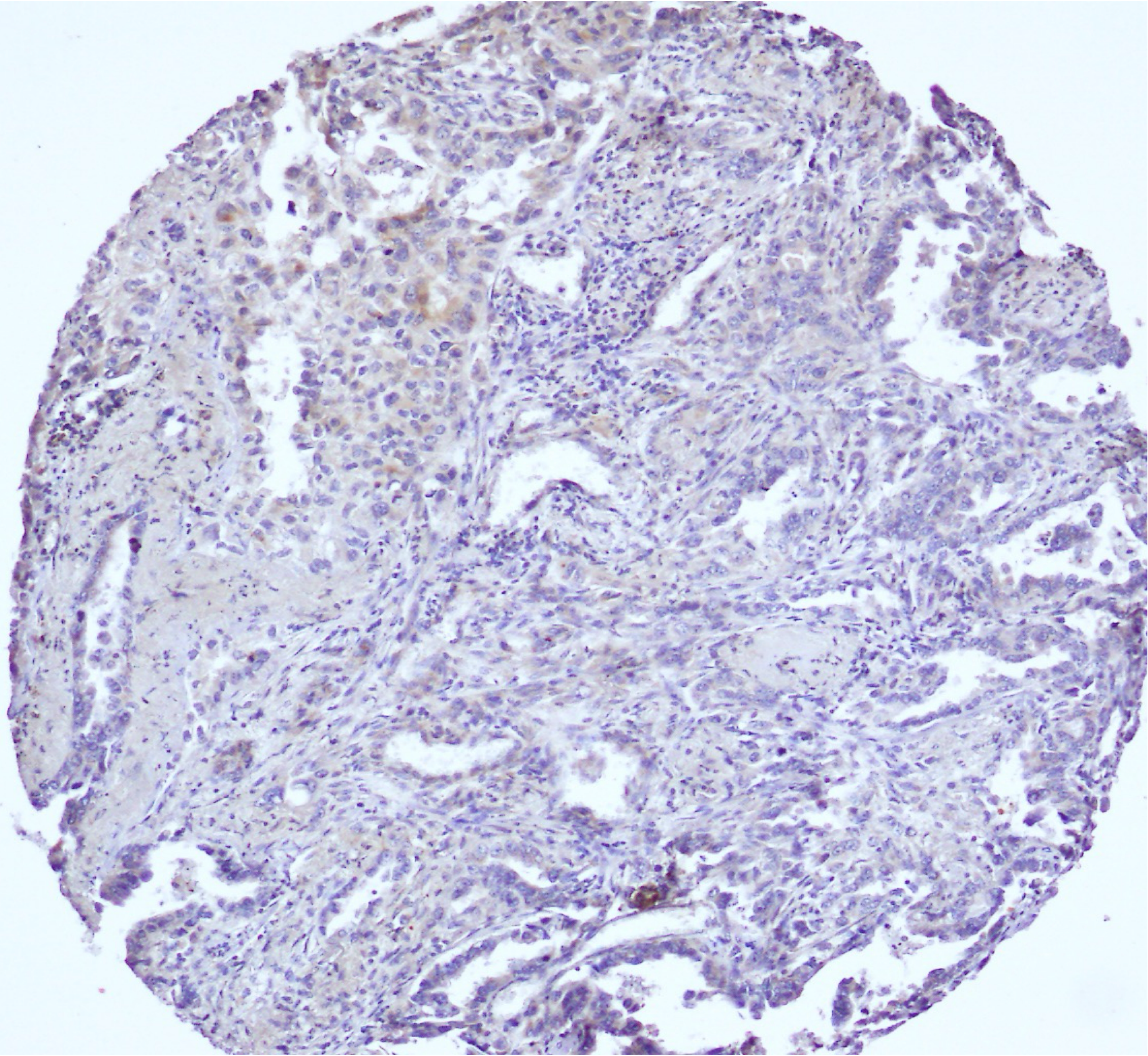

Supplement: S3 File — (ZIP) [file pone.0349359.s003.zip › Figure 3B AKT2 ADC left 10x.pdf]

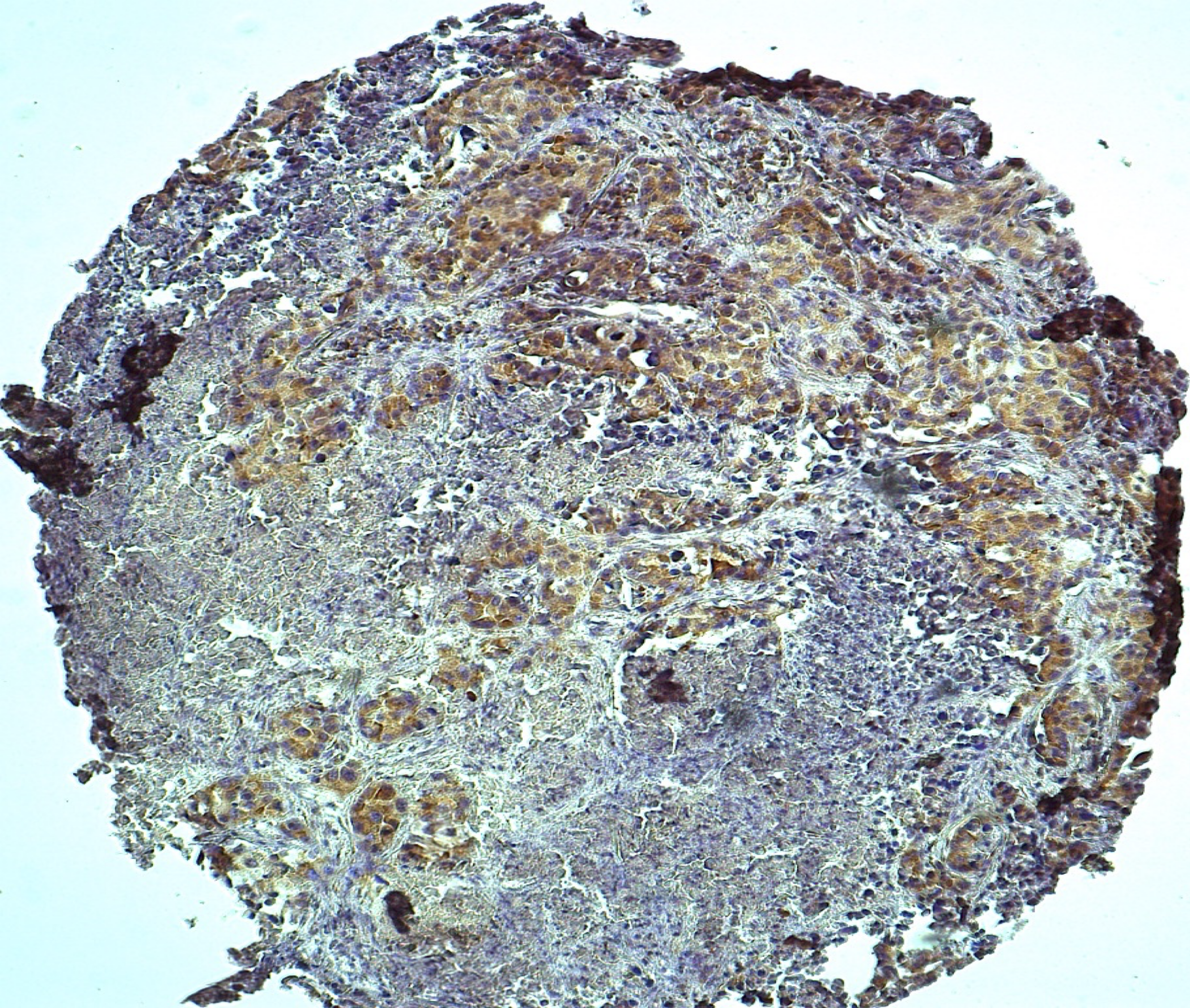

Supplement: S3 File — (ZIP) [file pone.0349359.s003.zip › Figure 3B AKT2 ADC right 10x.tiff]

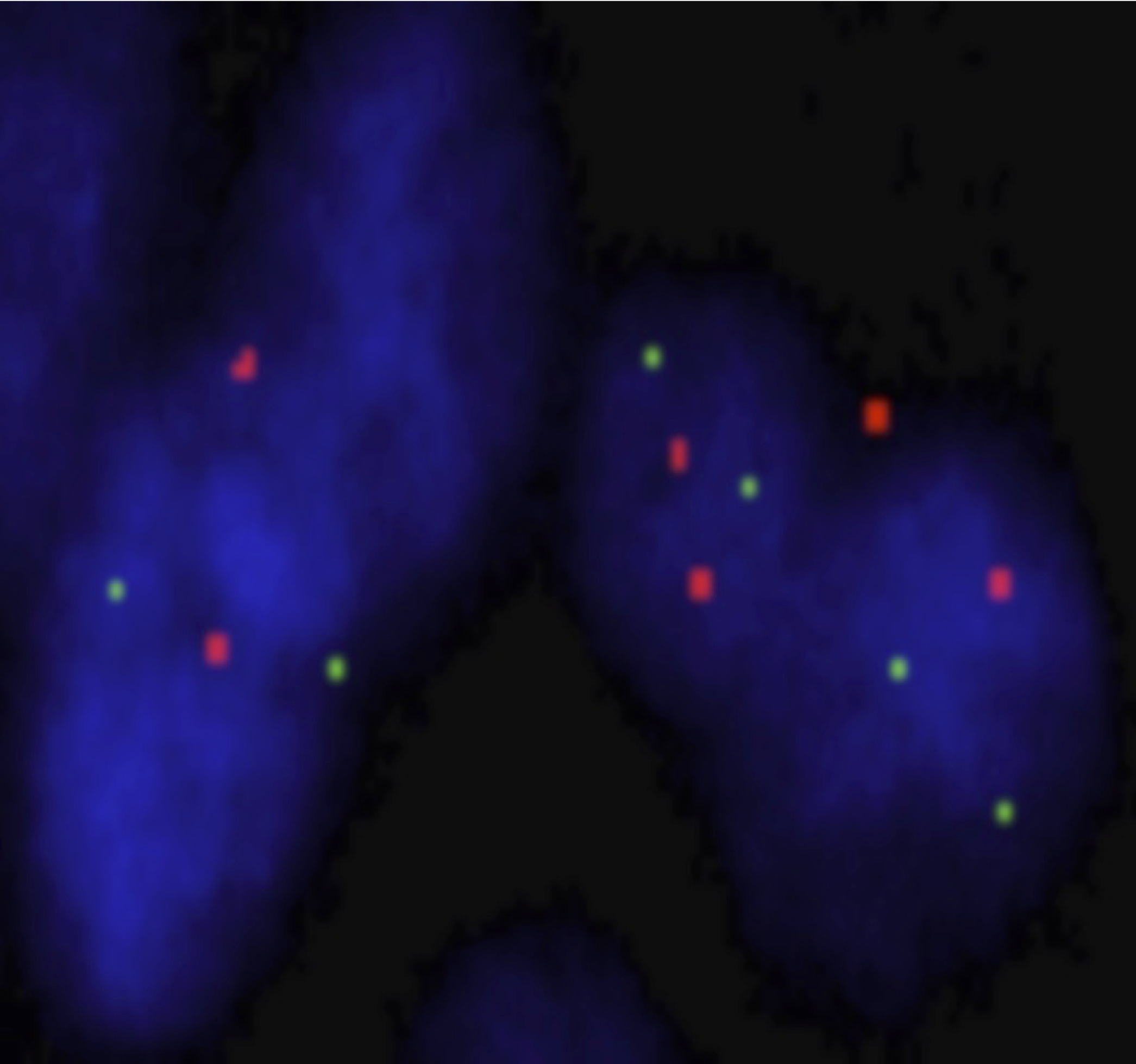

Supplement: S3 File — (ZIP) [file pone.0349359.s003.zip › Figure 3C AKT2 diploid left.tiff]

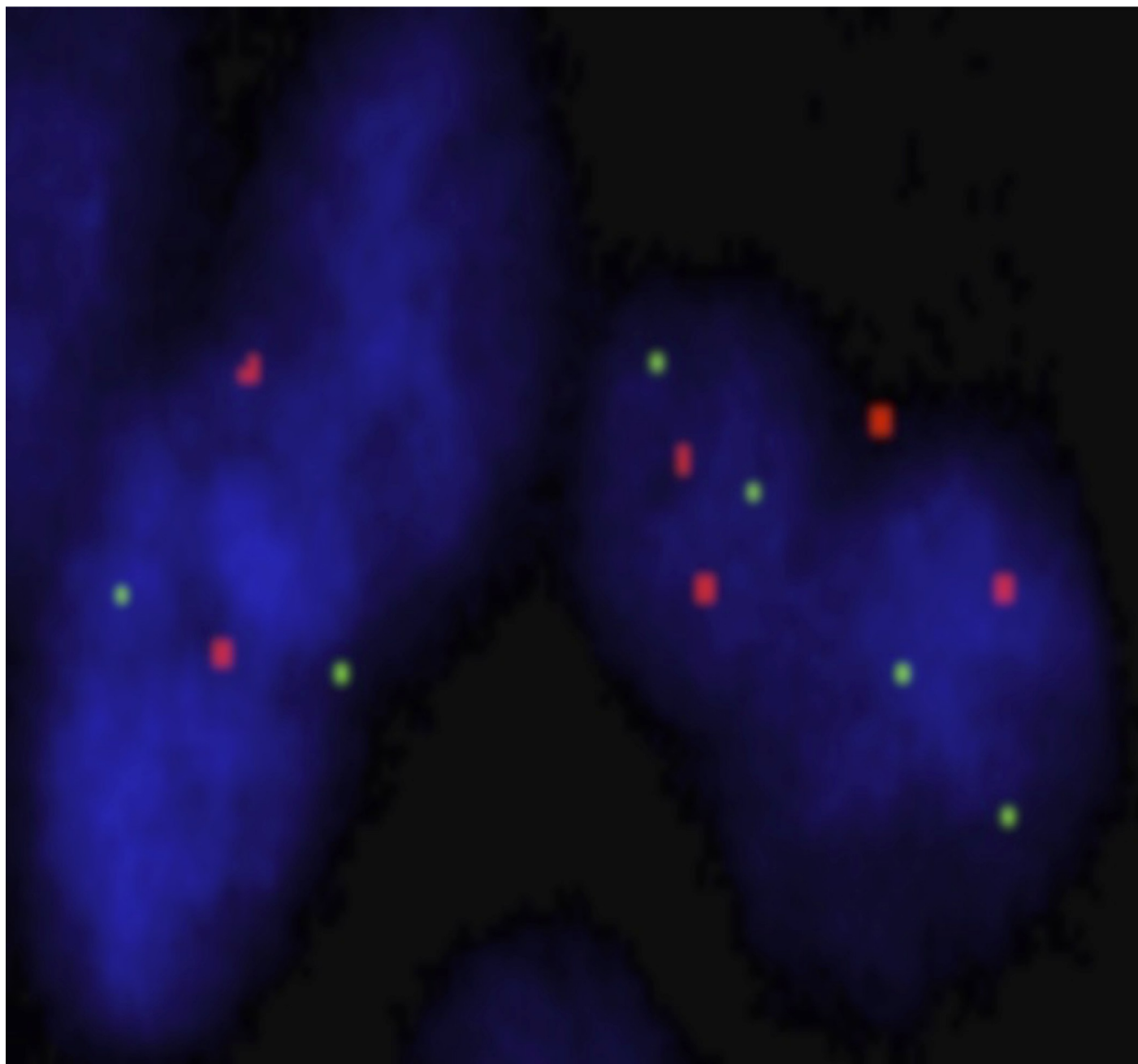

Supplement: S3 File — (ZIP) [file pone.0349359.s003.zip › Figure 3C AKT2 diploid left.pdf]

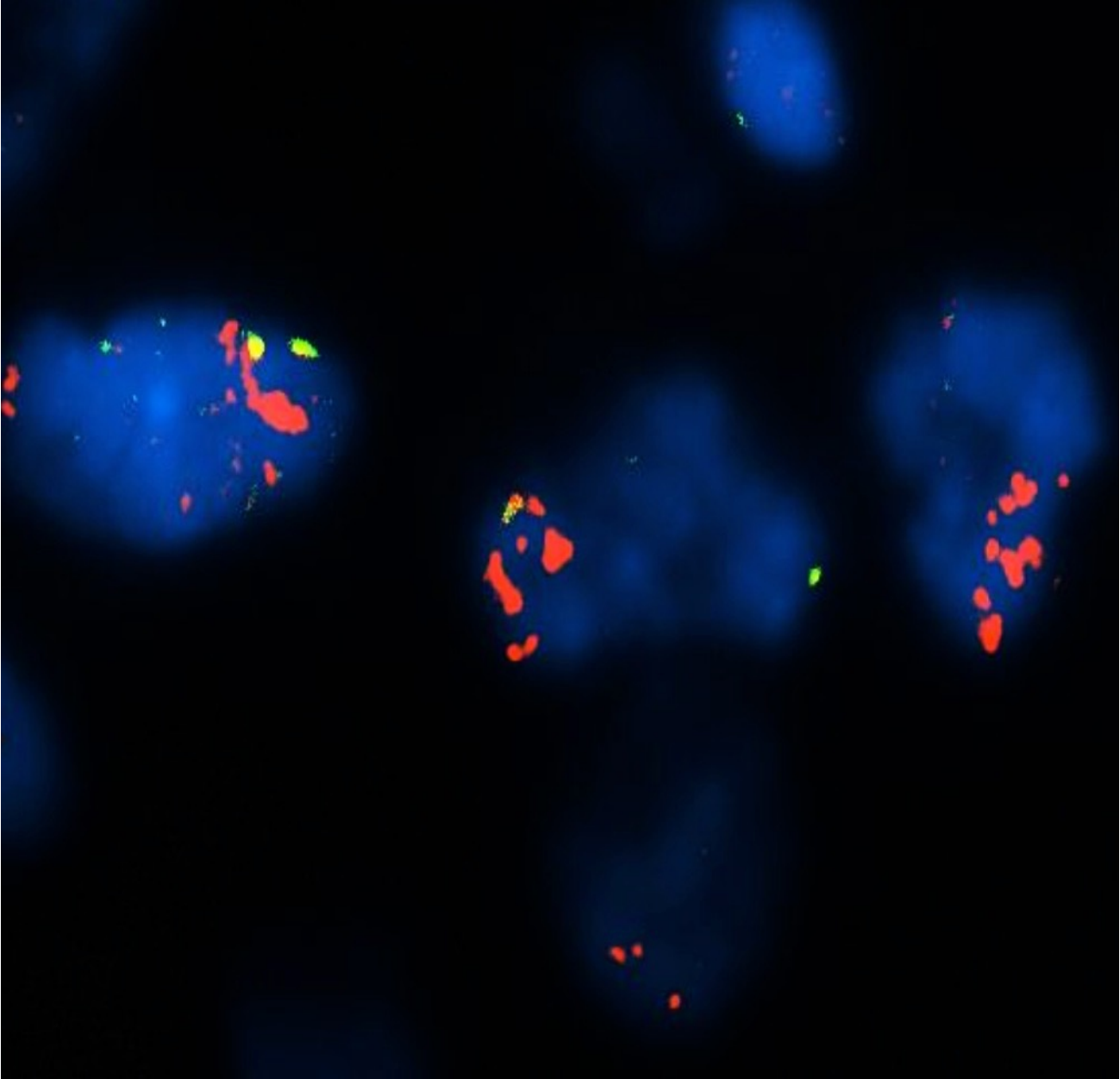

Supplement: S3 File — (ZIP) [file pone.0349359.s003.zip › Figure 3C AKT2 gene amplification right.pdf]

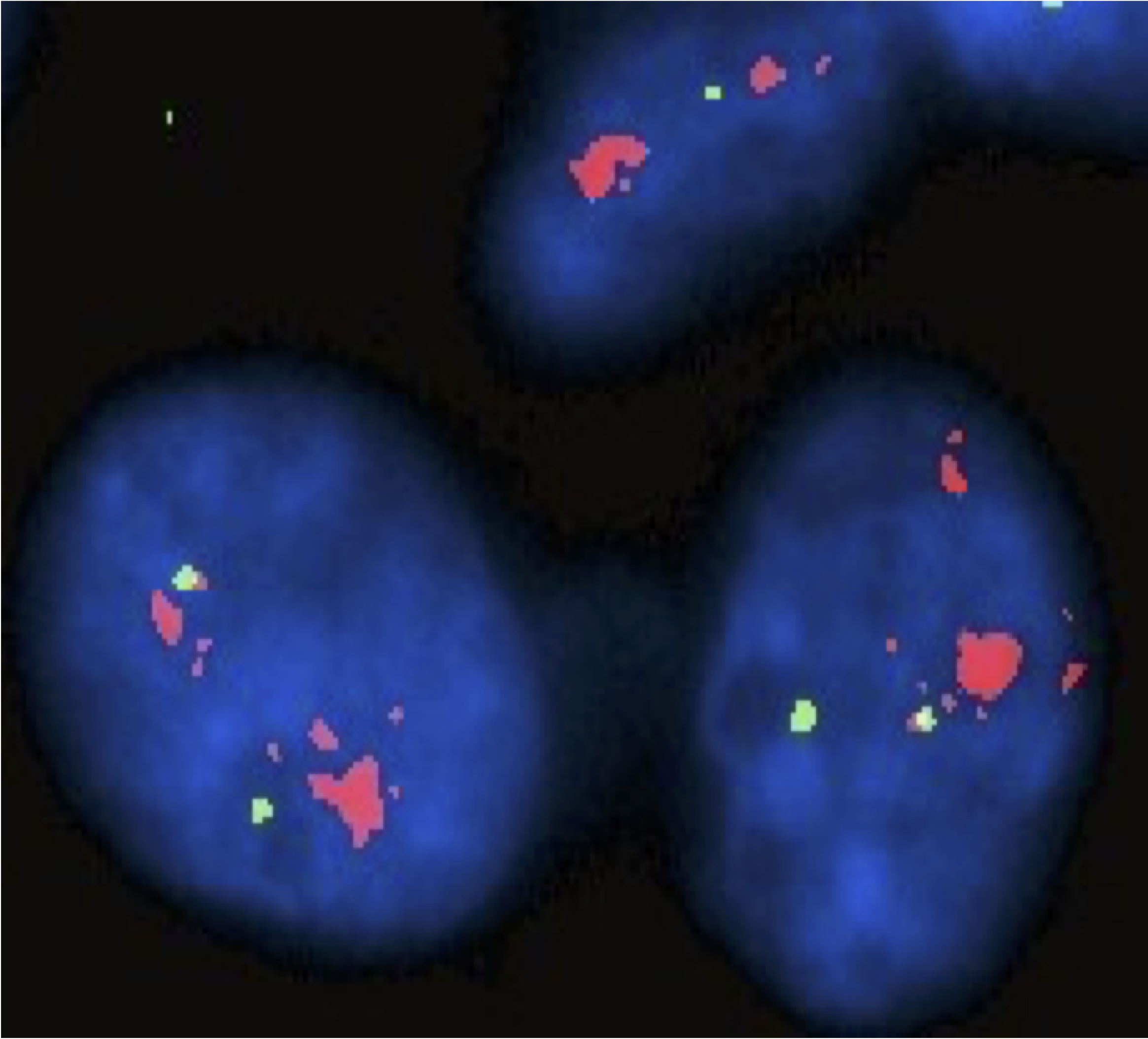

Supplement: S4 File — (ZIP) [file pone.0349359.s004.zip › Figure 4C PI3KCA gene amplification right .tiff]

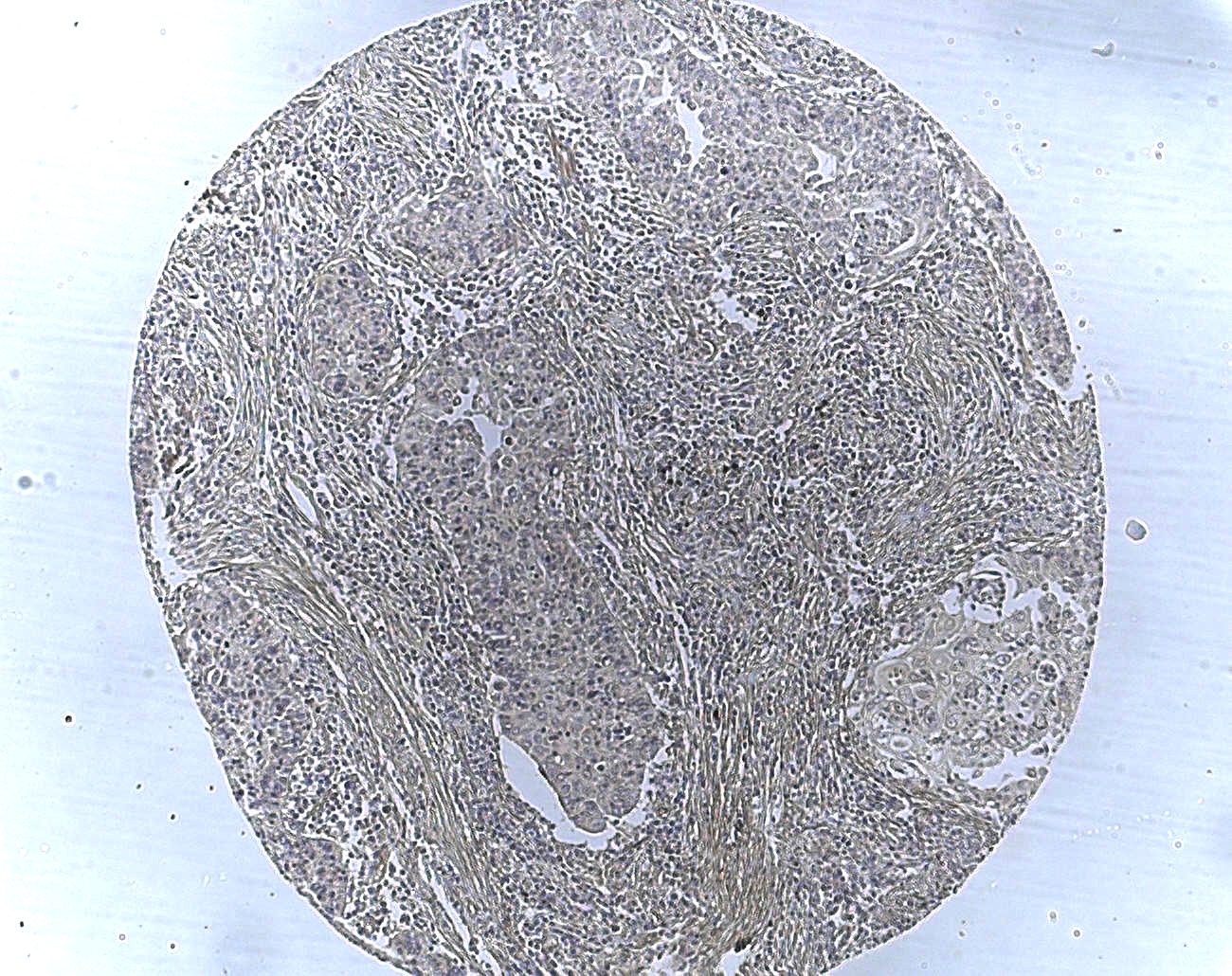

Supplement: S4 File — (ZIP) [file pone.0349359.s004.zip › Figure 4A SCC PI3KCA left 10x.jpg]

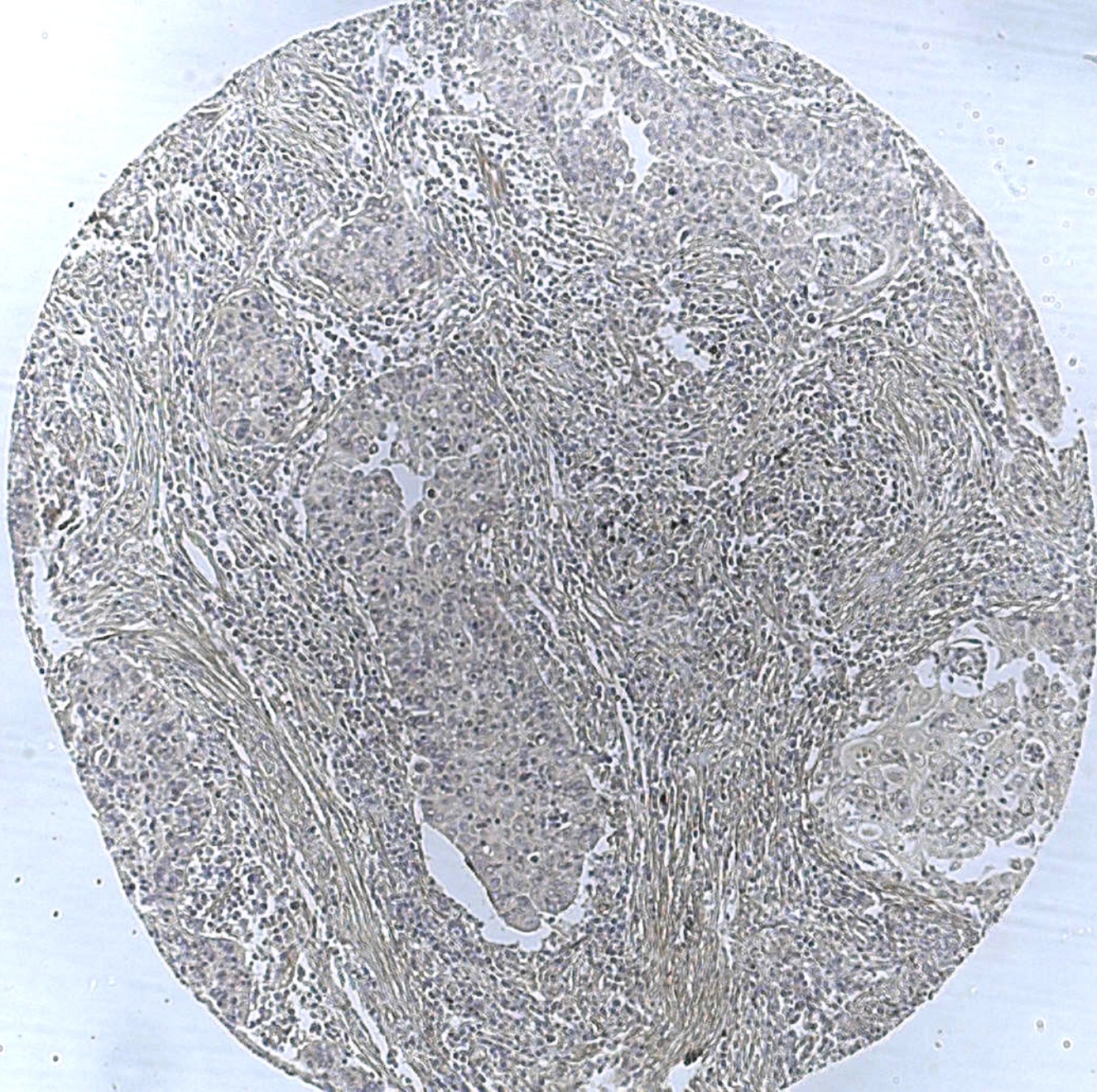

Supplement: S4 File — (ZIP) [file pone.0349359.s004.zip › Figure 4A SCC PI3KCA left 10x.pdf]

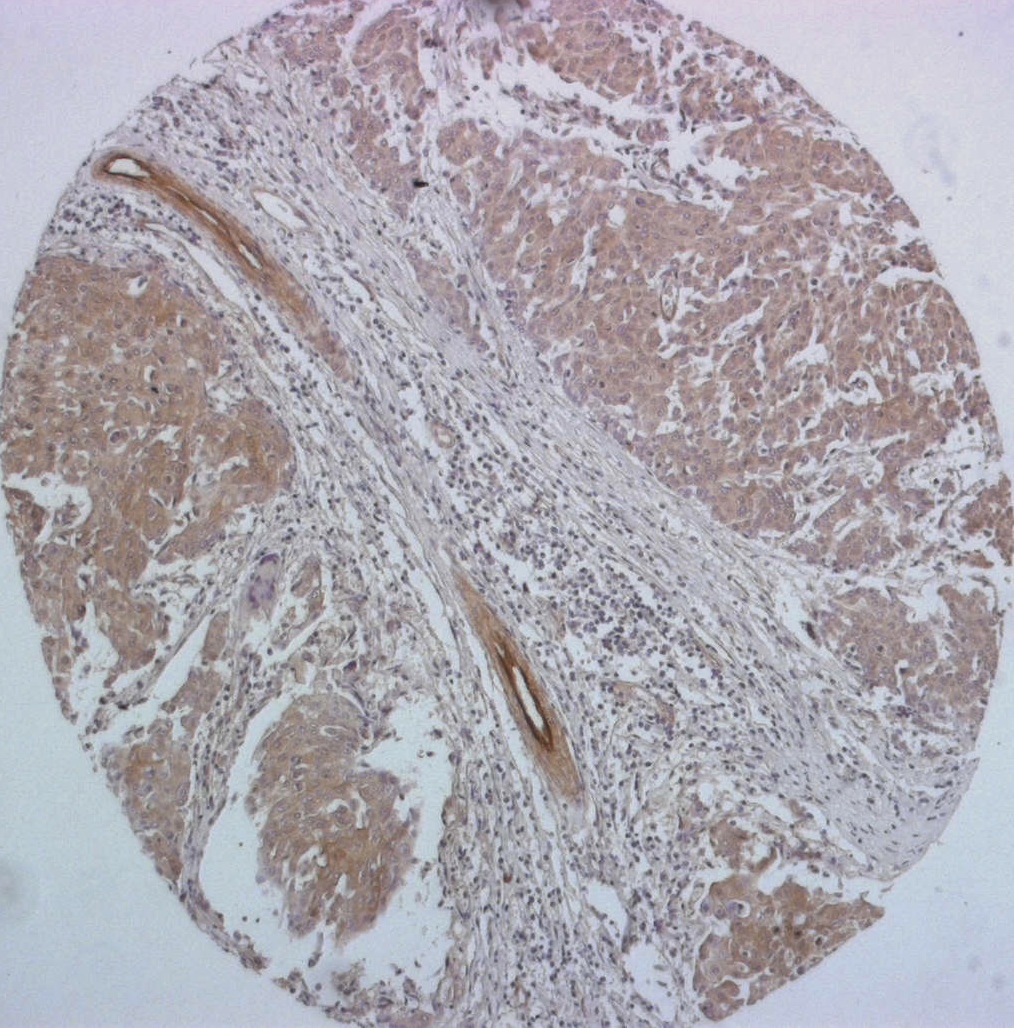

Supplement: S4 File — (ZIP) [file pone.0349359.s004.zip › Figure 4A SCC PI3KCA right 10x.jpg]

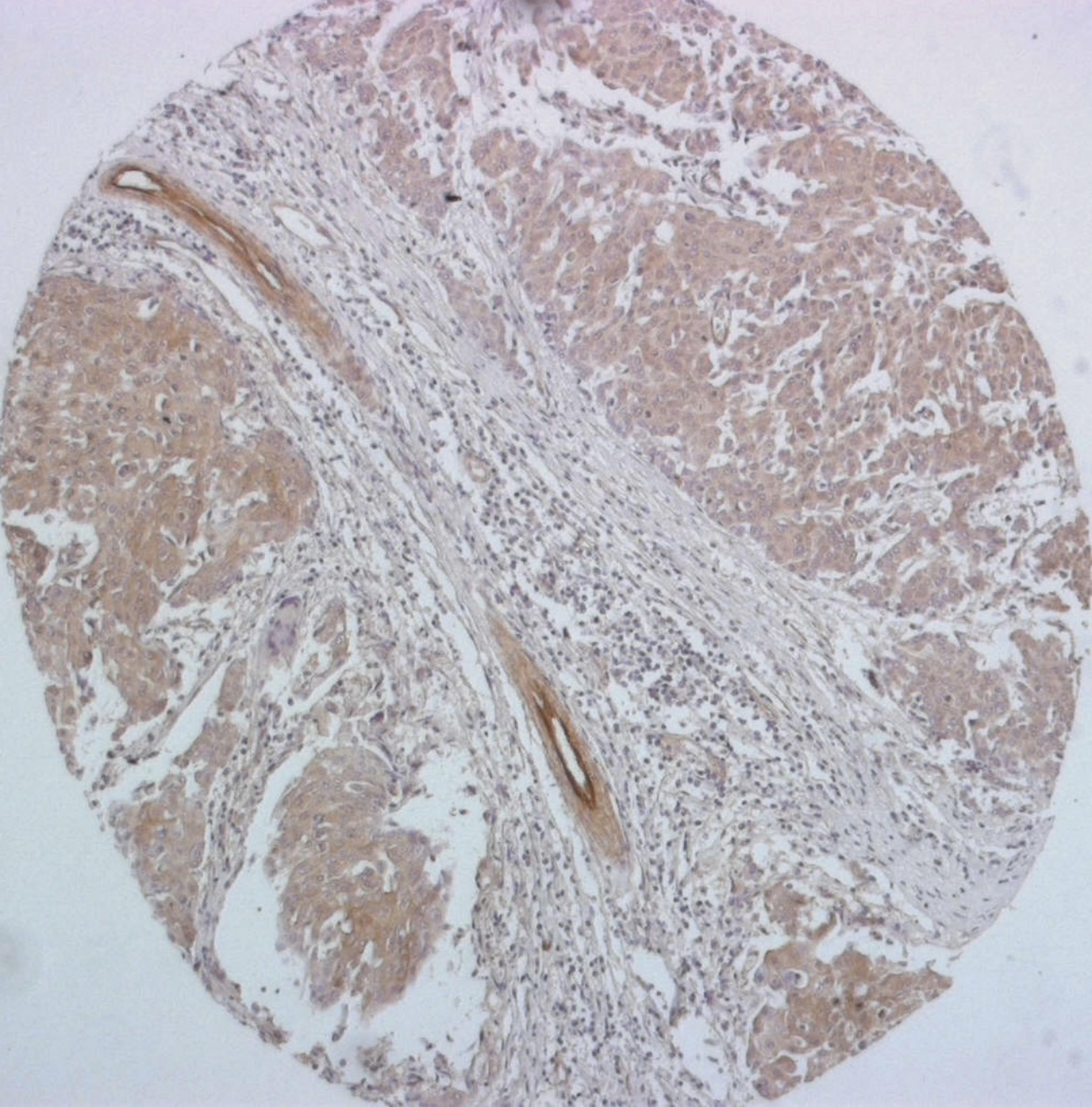

Supplement: S4 File — (ZIP) [file pone.0349359.s004.zip › Figure 4A SCC PI3KCA right 10x.pdf]

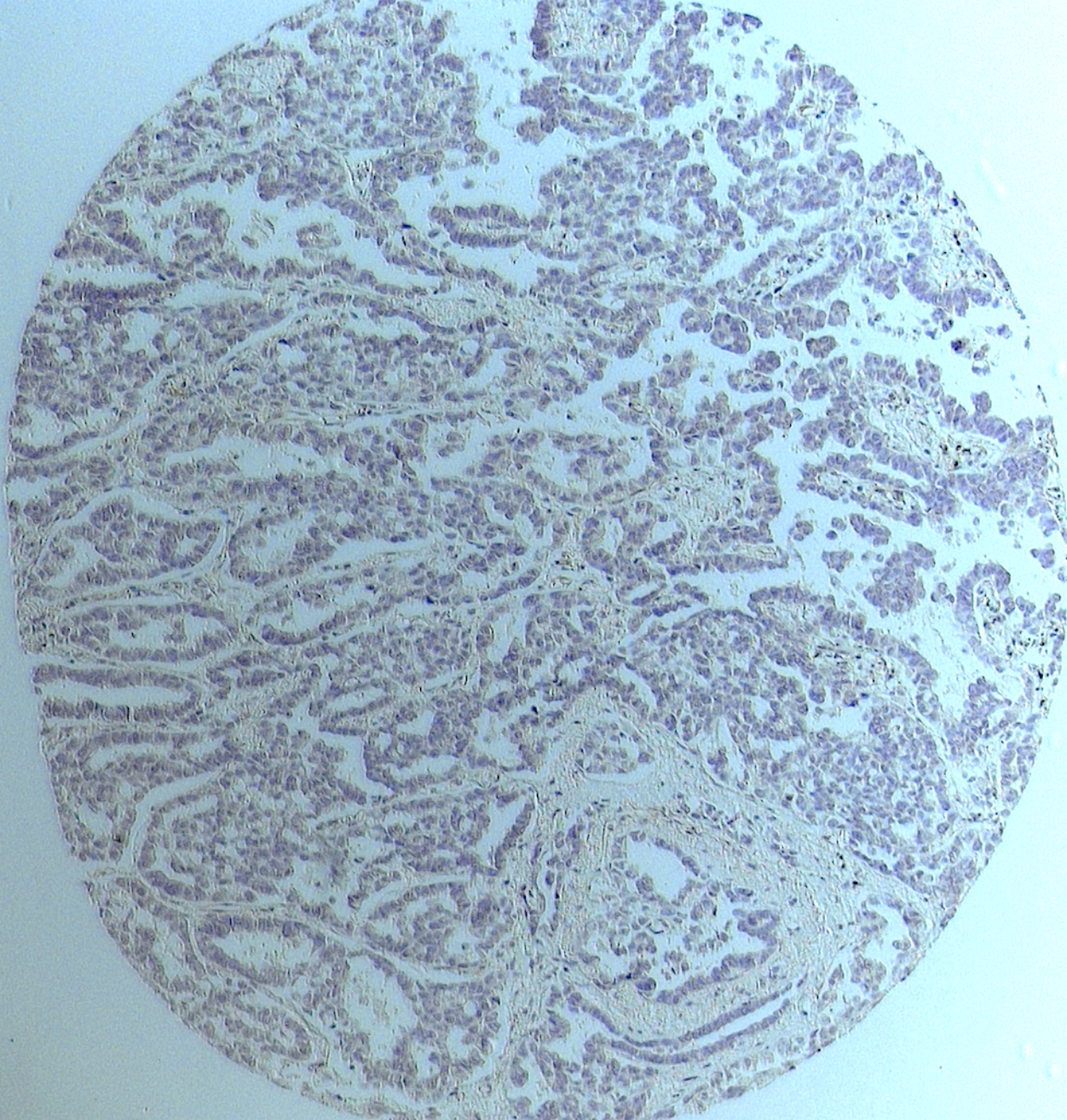

Supplement: S4 File — (ZIP) [file pone.0349359.s004.zip › Figure 4B ADC PI3KCA left 10x.pdf]

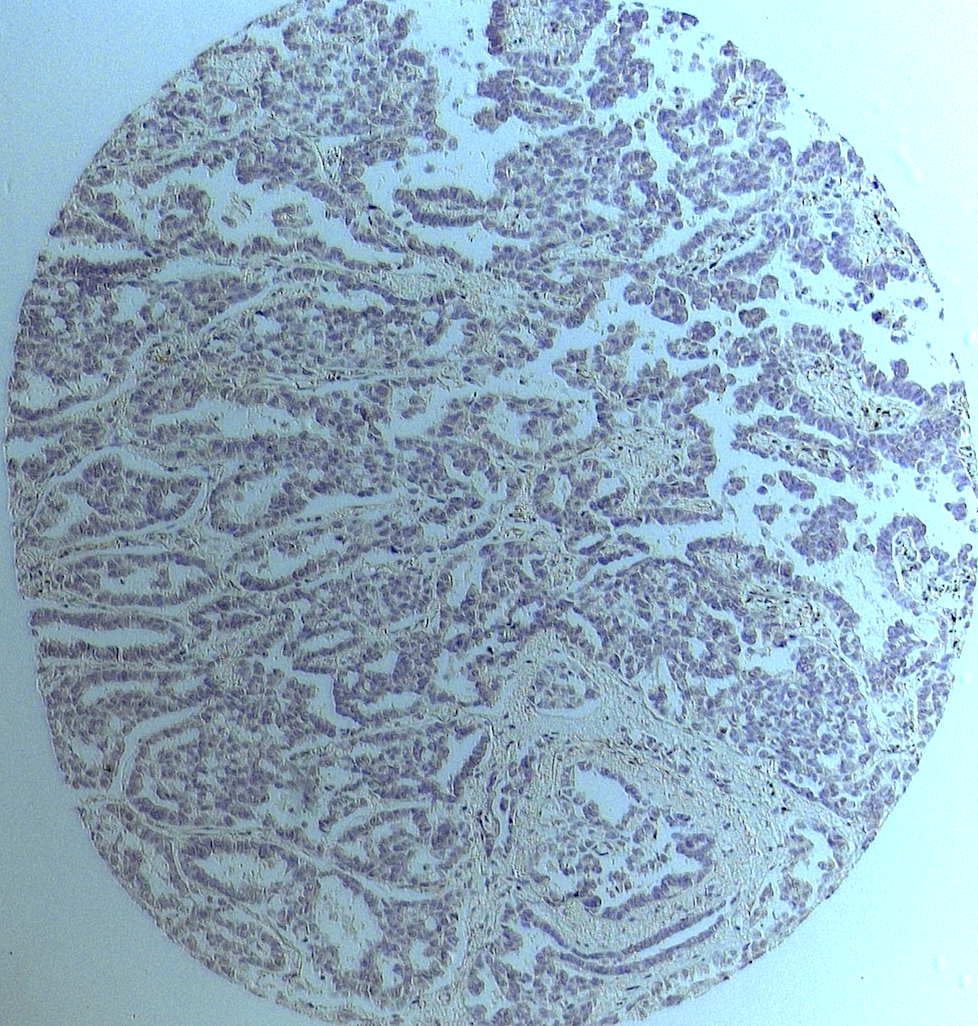

Supplement: S4 File — (ZIP) [file pone.0349359.s004.zip › Figure 4B ADC PI3KCA left 10x.tif]

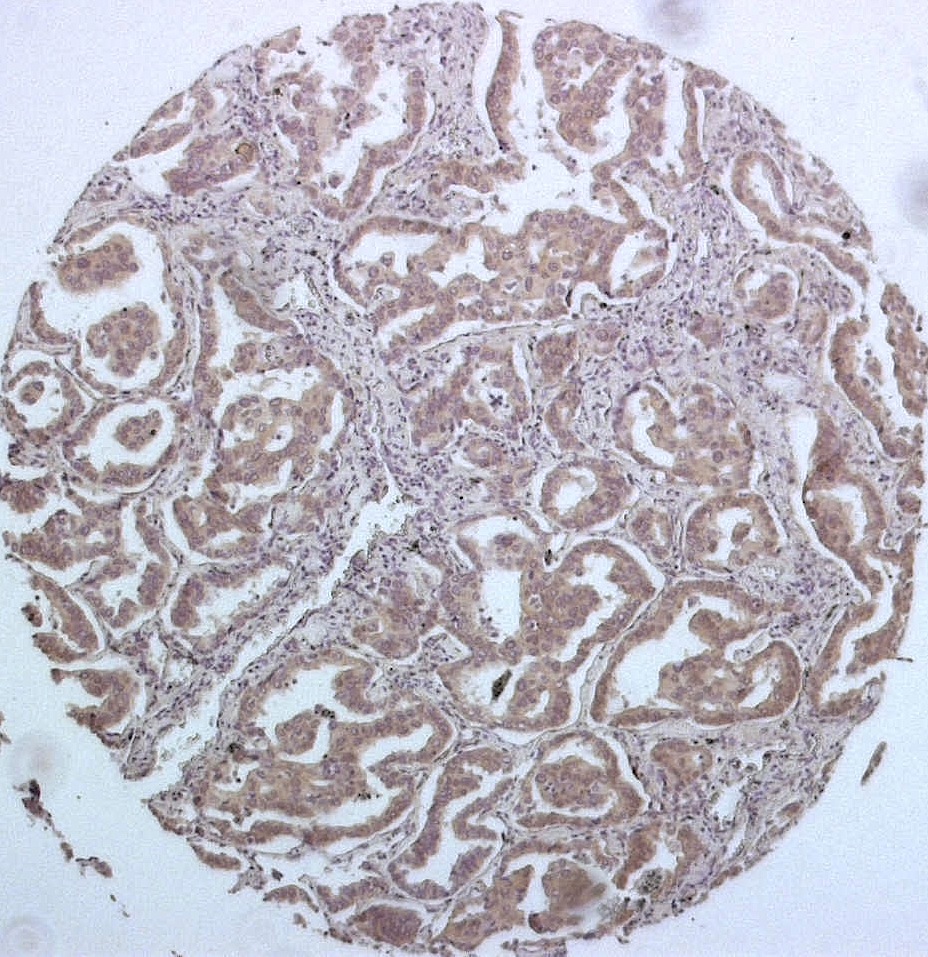

Supplement: S4 File — (ZIP) [file pone.0349359.s004.zip › Figure 4B ADC PI3KCA right 10x.jpg]

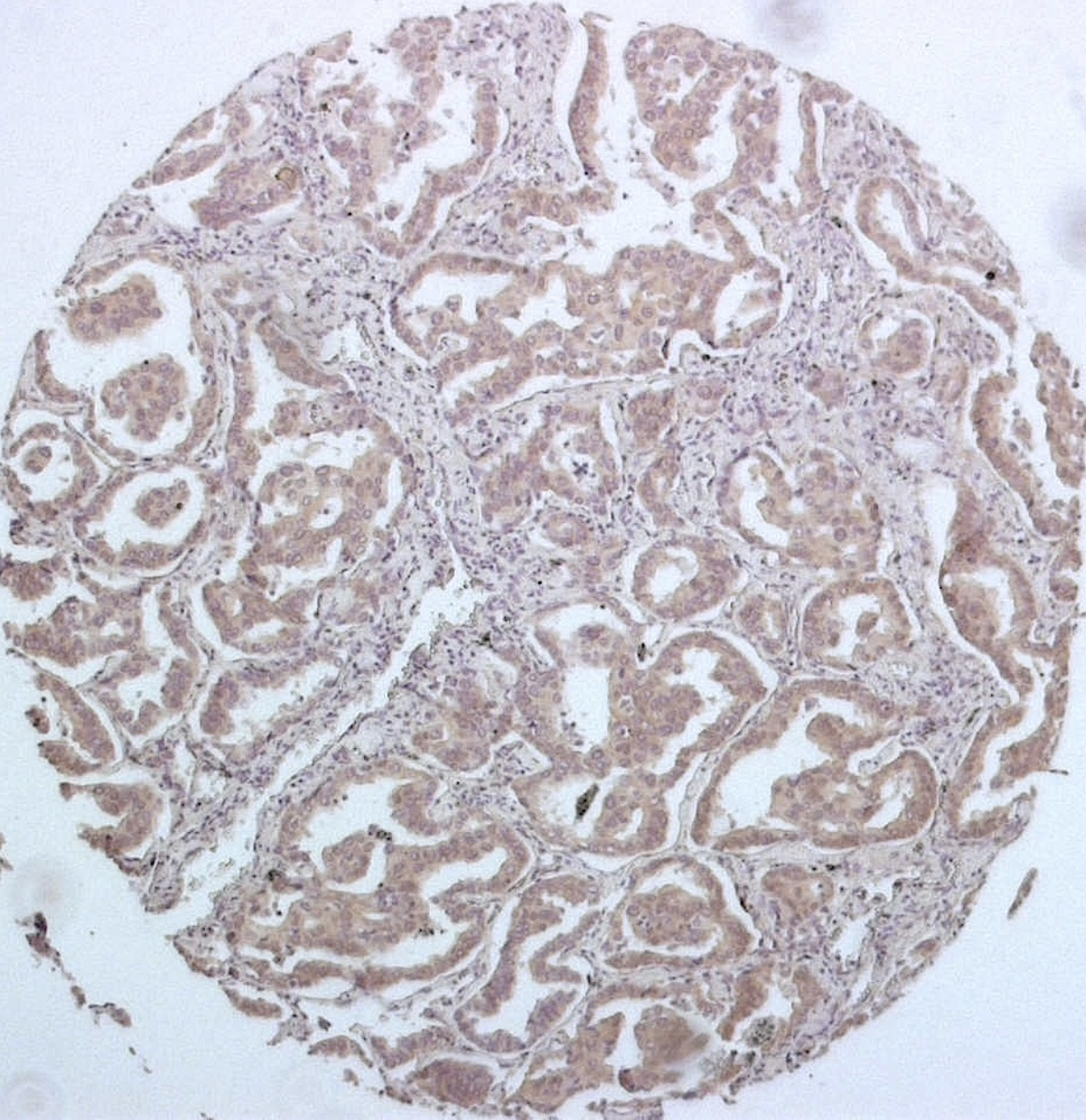

Supplement: S4 File — (ZIP) [file pone.0349359.s004.zip › Figure 4B ADC PI3KCA right 10x.pdf]

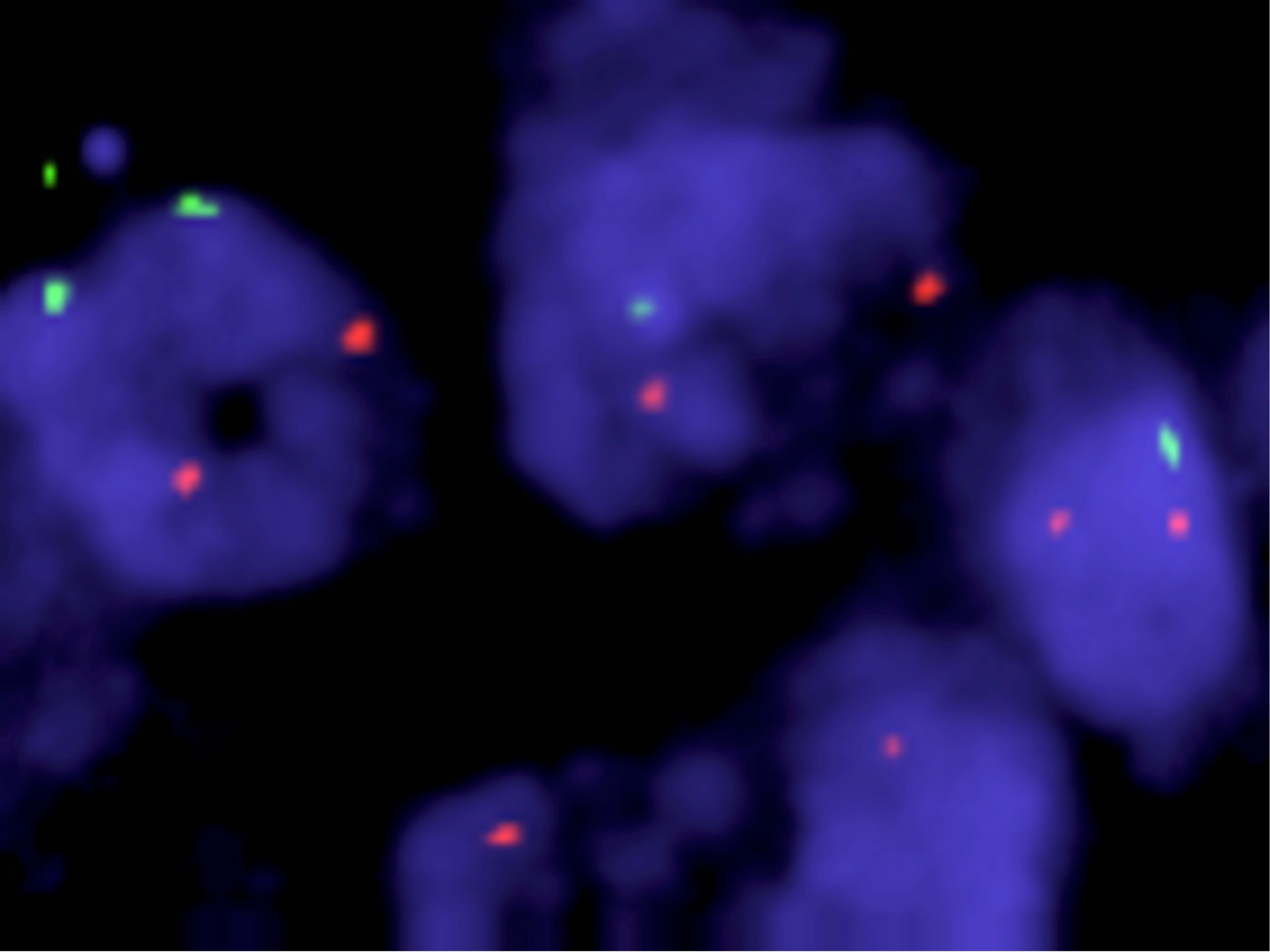

Supplement: S4 File — (ZIP) [file pone.0349359.s004.zip › Figure 4C PI3KCA diploid cells left.pdf]

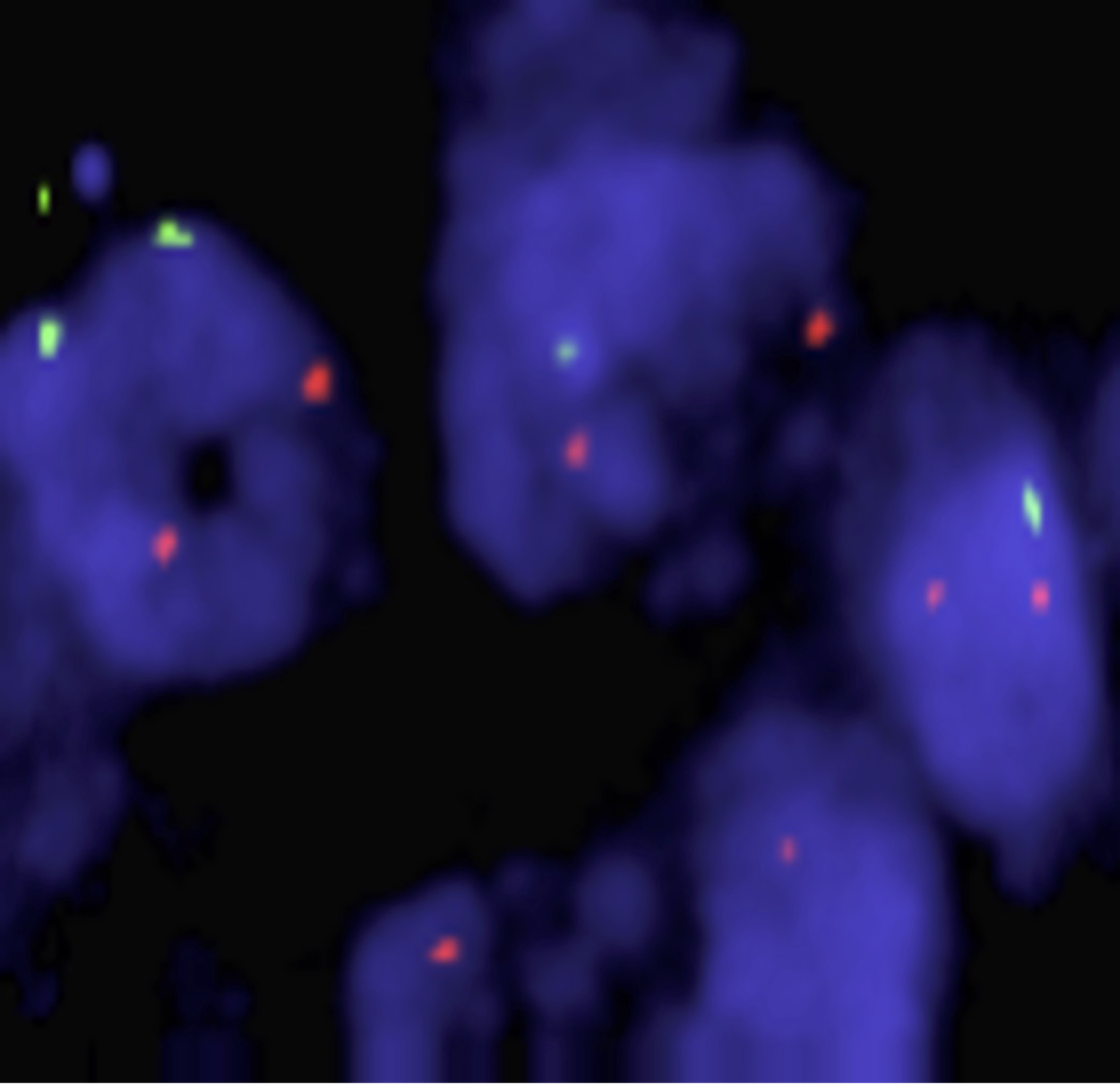

Supplement: S4 File — (ZIP) [file pone.0349359.s004.zip › Figure 4C PI3KCA diploid cells left.tiff]

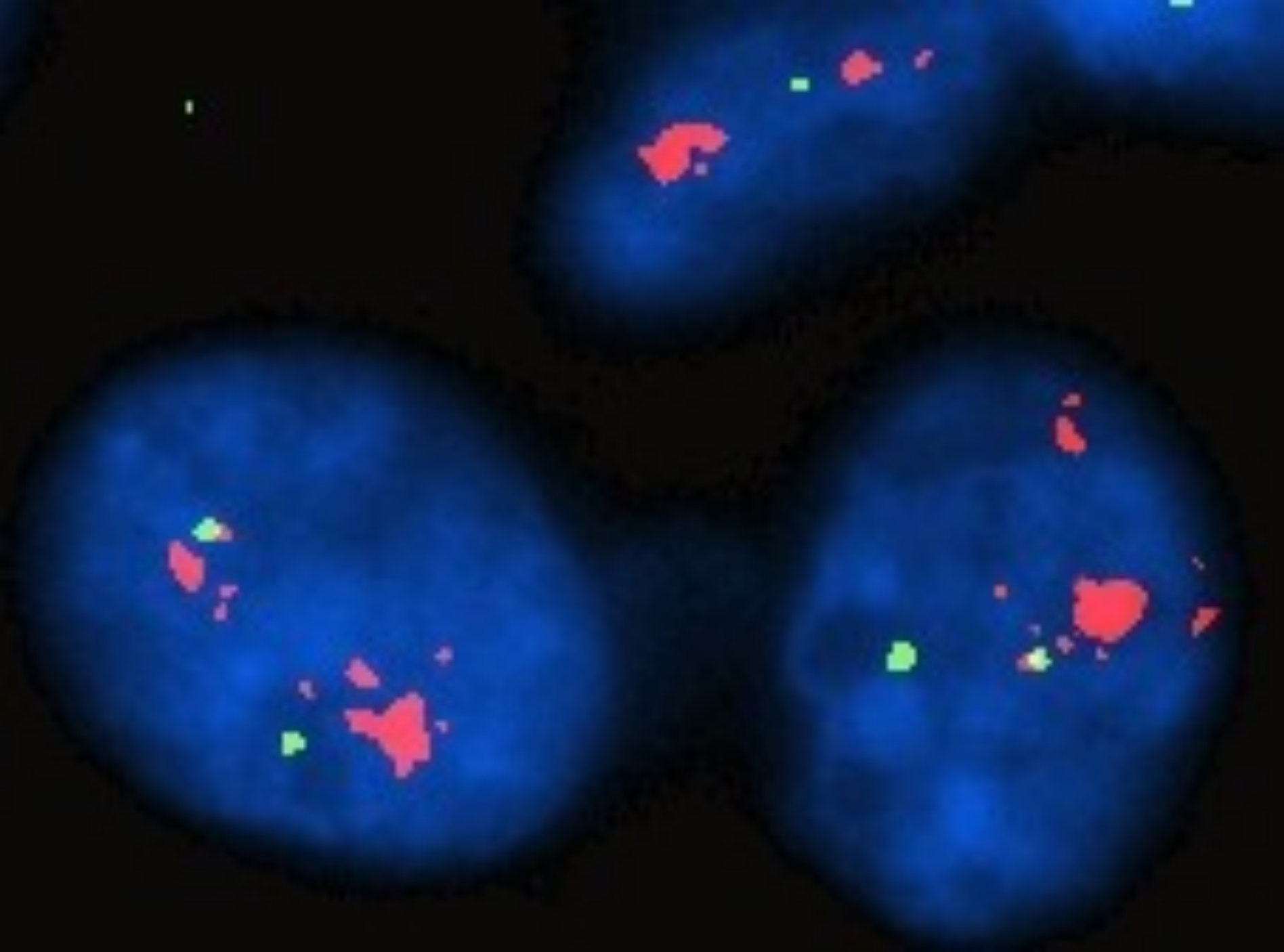

Supplement: S4 File — (ZIP) [file pone.0349359.s004.zip › Figure 4C PI3KCA gene amplification right .pdf]

# Melting Peaks

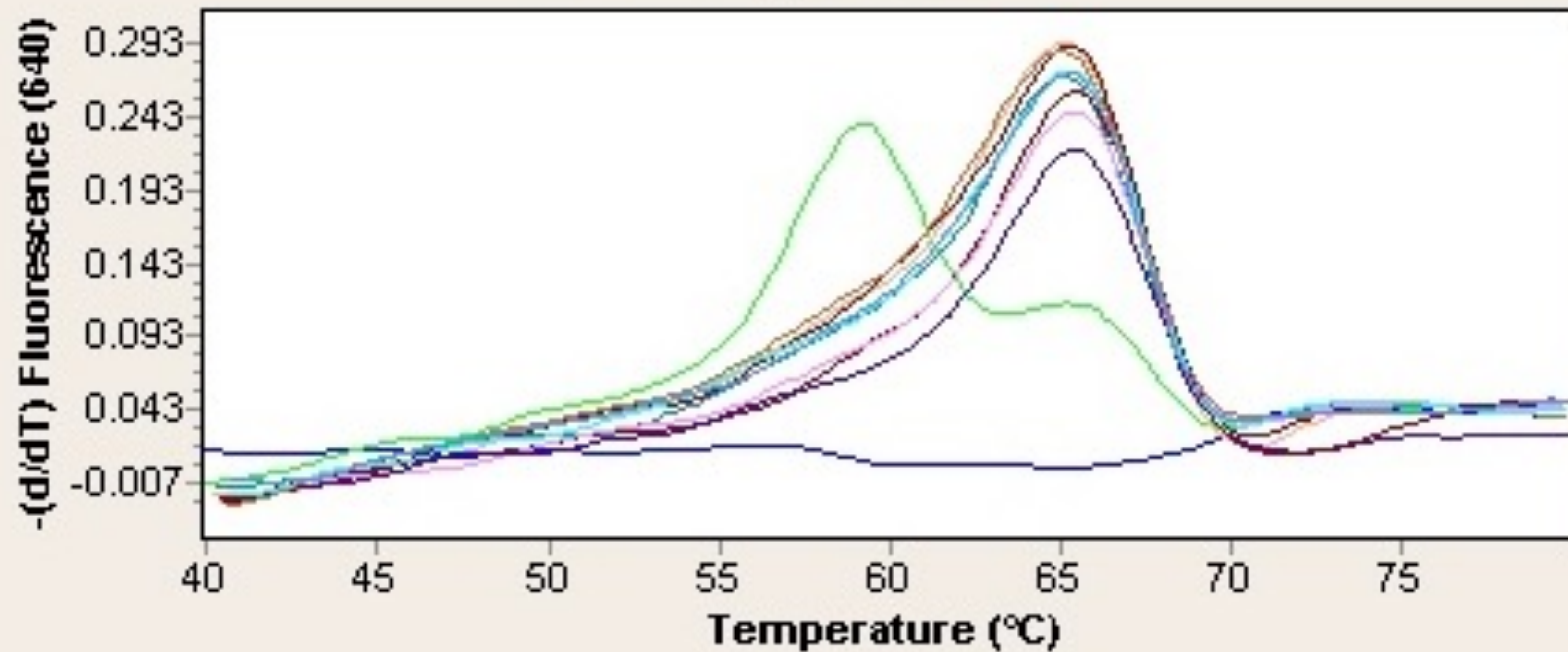

Supplement: S5 File — The electropherograms were derived from an ABIPRISM 3100 sequencer. The melting curve for PI3KCA mutations are images taken from a Roche Lightcycler machine. (ZIP) [file pone.0349359.s005.zip › Figure 5A PI3KCA exon 9.pdf]

# Melting Peaks

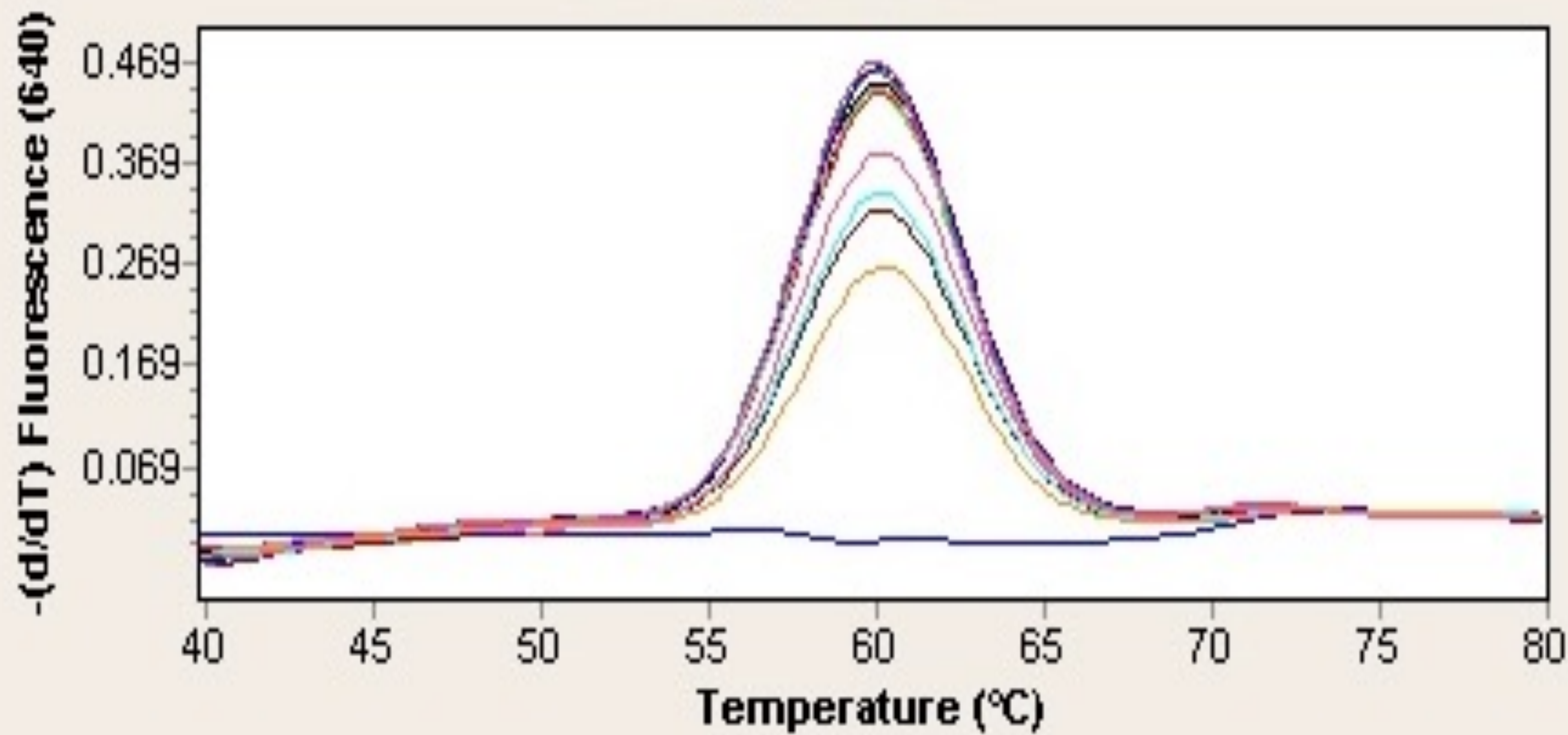

Supplement: S5 File — The electropherograms were derived from an ABIPRISM 3100 sequencer. The melting curve for PI3KCA mutations are images taken from a Roche Lightcycler machine. (ZIP) [file pone.0349359.s005.zip › Figure 5A PI3KCA exon 20.pdf]

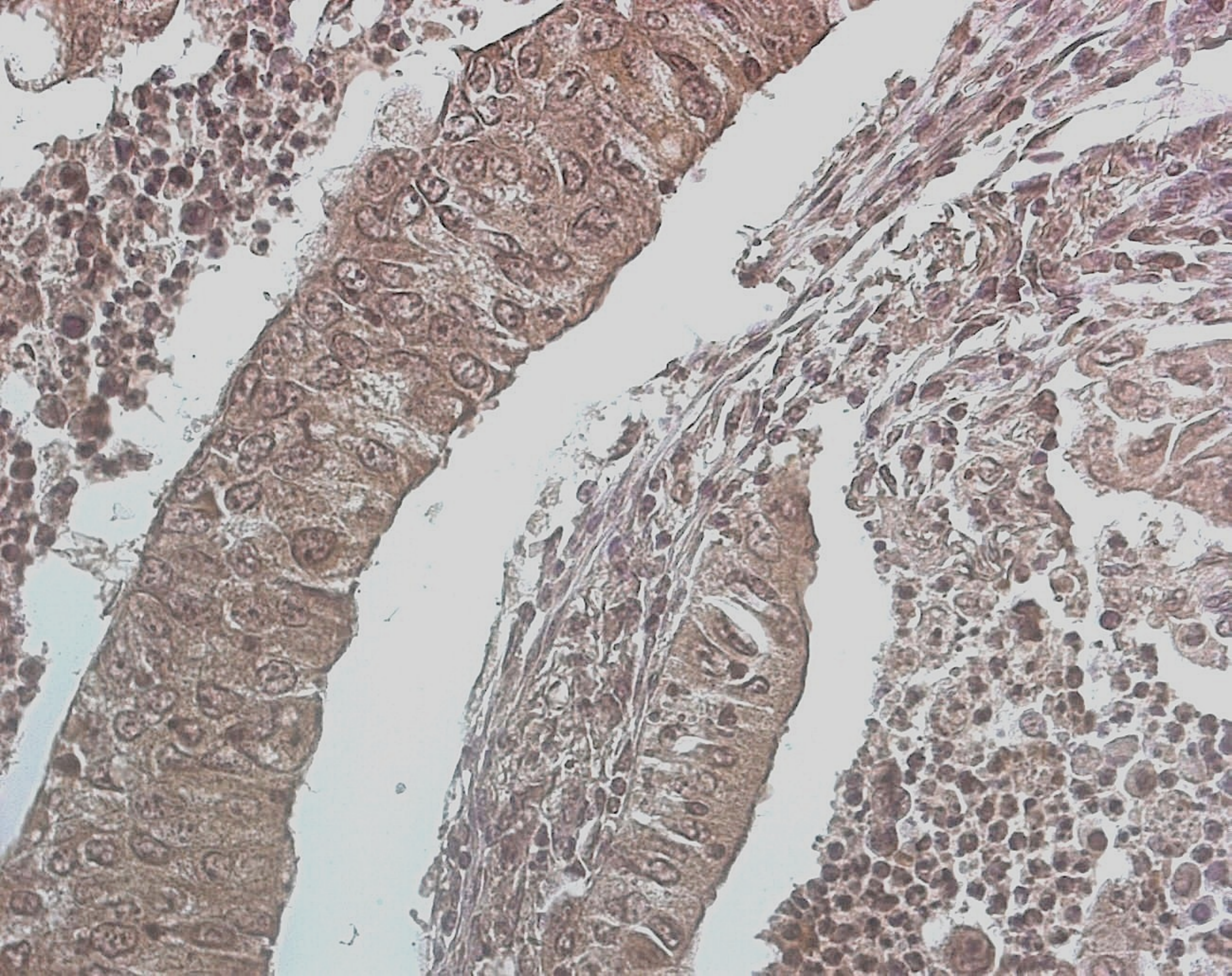

Supplement: S5 File — The electropherograms were derived from an ABIPRISM 3100 sequencer. The melting curve for PI3KCA mutations are images taken from a Roche Lightcycler machine. (ZIP) [file pone.0349359.s005.zip › Figure 5D pAKT ADC-30 40x.pdf]

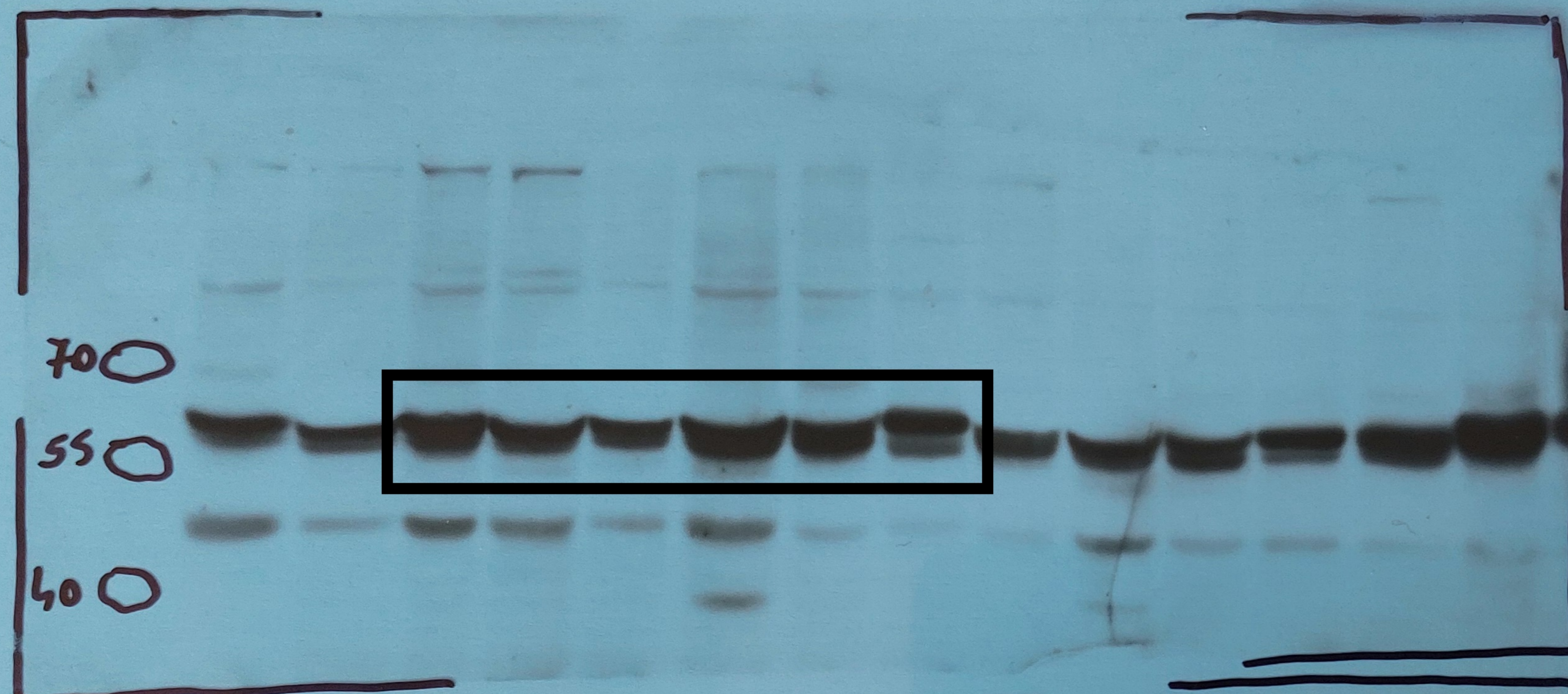

70 ○

55 ○

40 ○

dA KT  
(A 255)

UPSTATE

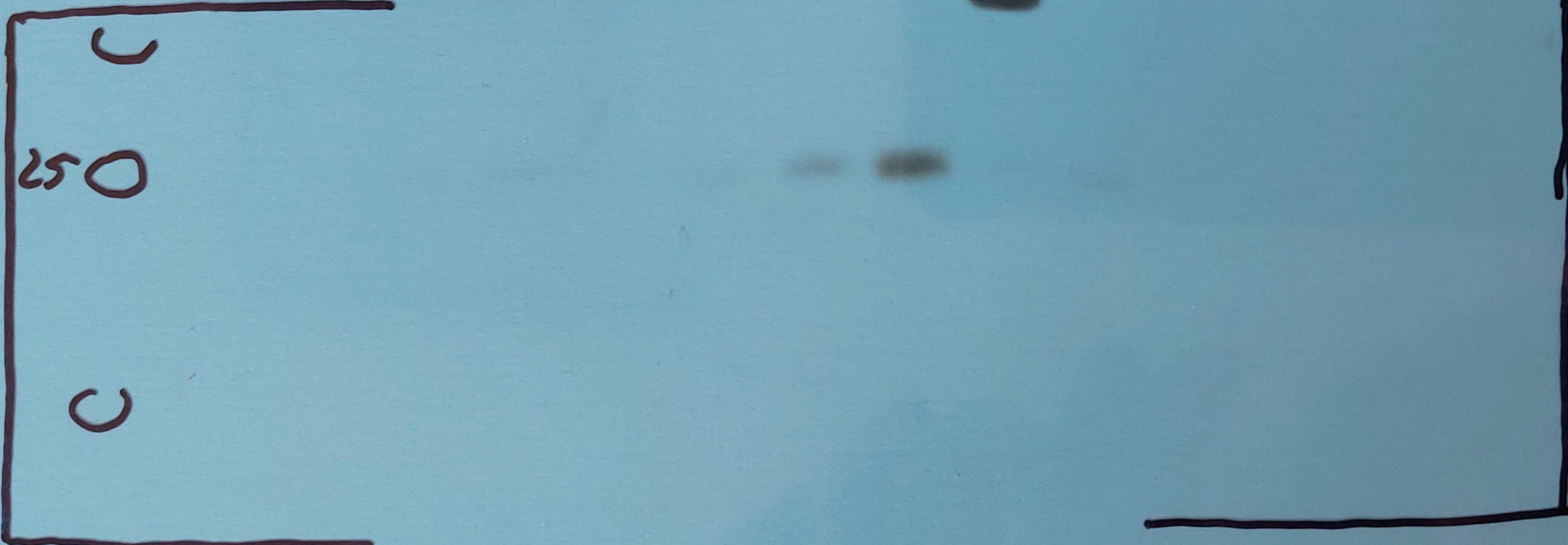

250 ○

2 pcr Ras.

Supplement: S6 File — (ZIP) [file pone.0349359.s006.zip › Figure 6A AKT blot.pdf]

αPAKT

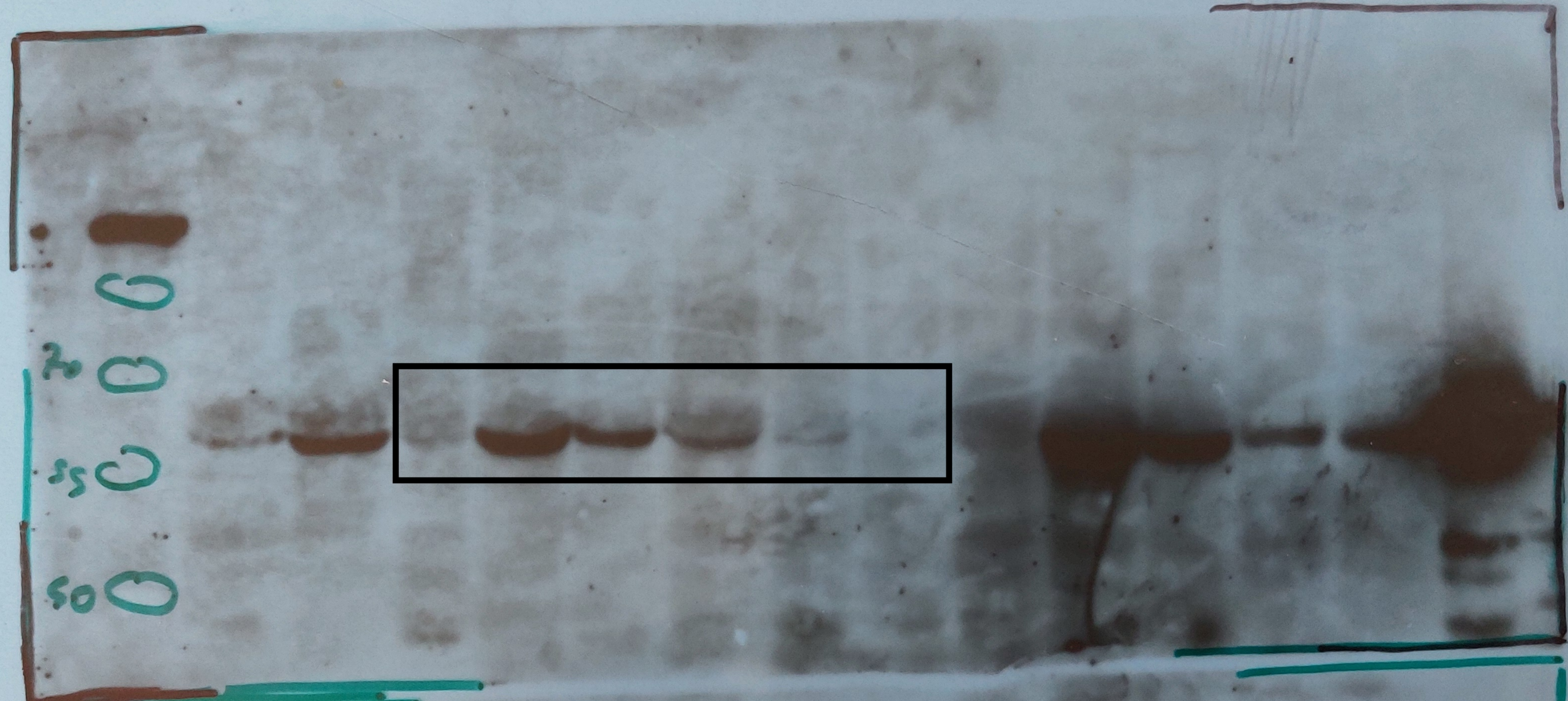

αp27<sup>h</sup>

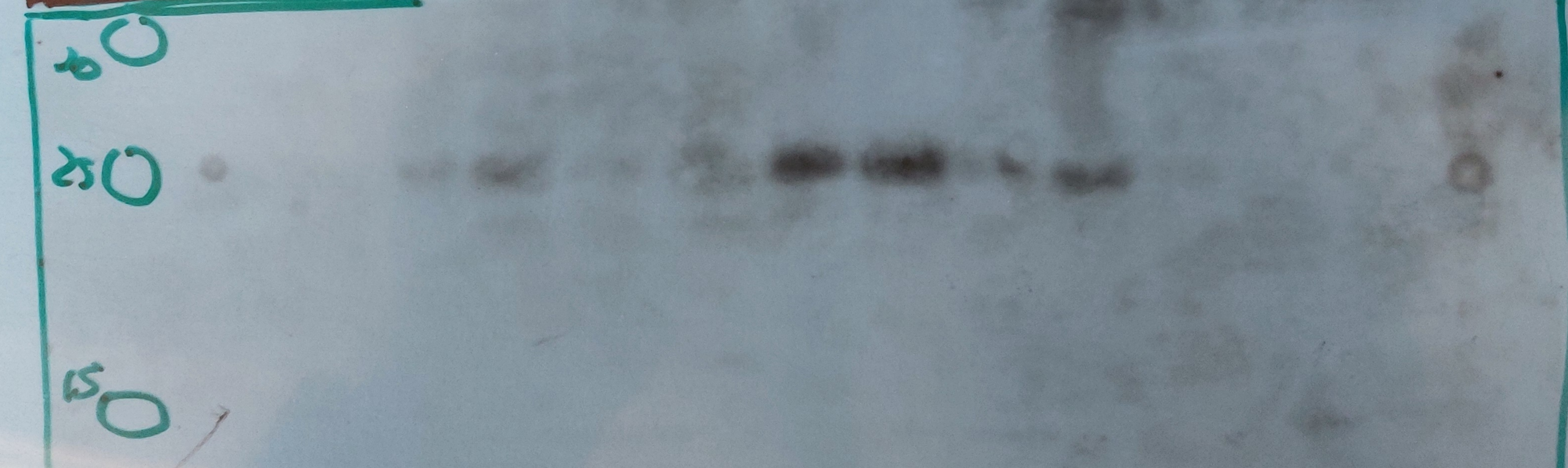

21/12/07

1h

Supplement: S6 File — (ZIP) [file pone.0349359.s006.zip › Figure 6A p-AKT blot.pdf]

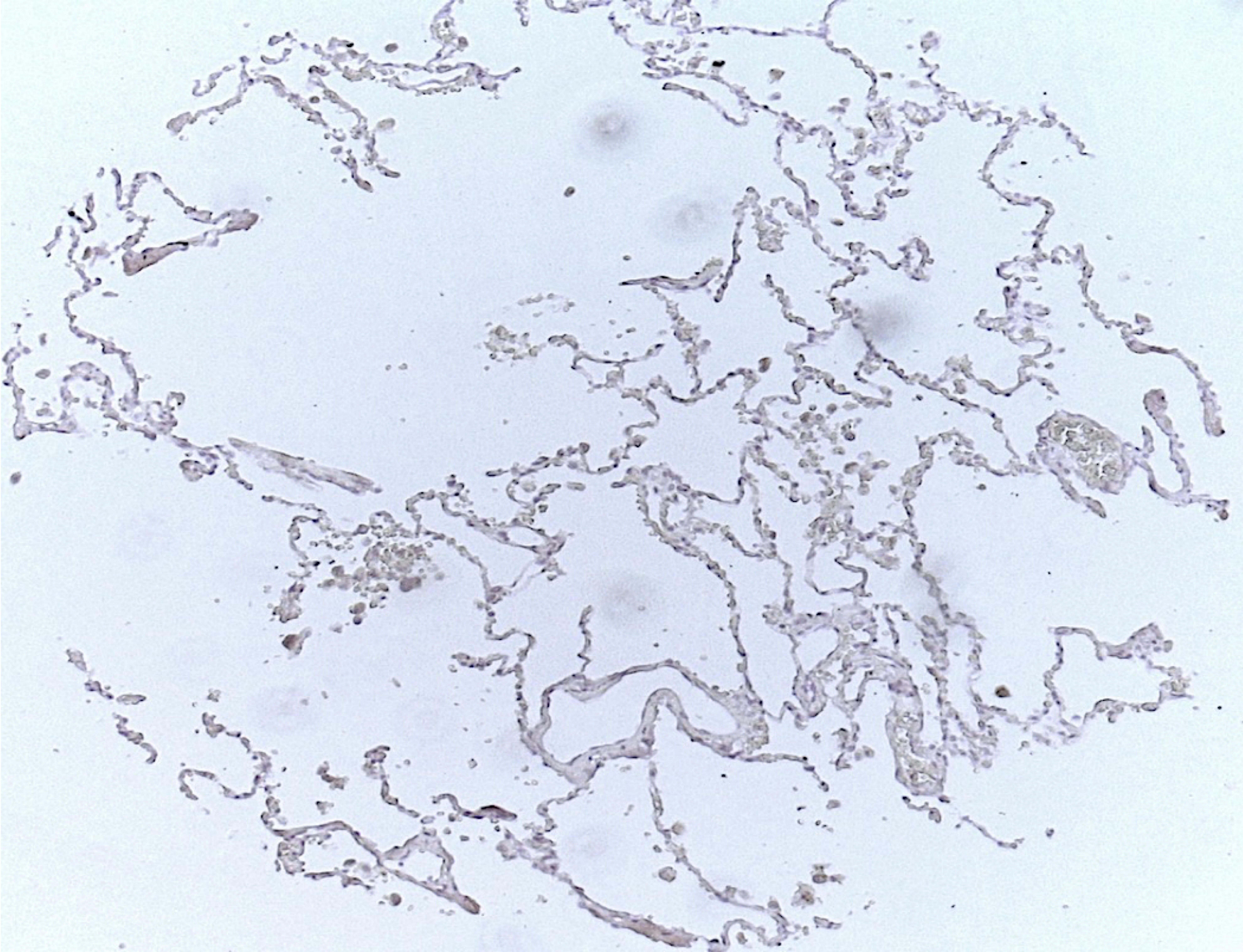

Supplement: S7 File — (ZIP) [file pone.0349359.s007.zip › Figure S1B PI3KCA normal 10x.pdf]

T. ESPOSIZIONE

5 min

28.09.11

┌

T

┐

polter  
unipice  
int. ANT

β. ACTIN  
HOUSE

0.50

0.50

1.00

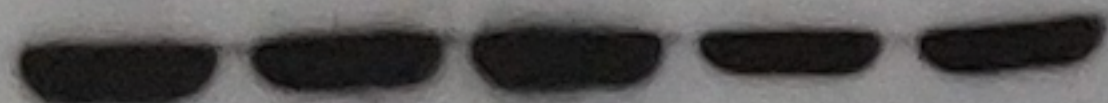

Supplement: S7 File — (ZIP) [file pone.0349359.s007.zip › Figure S1A_left_Actin blot.pdf]

$\alpha$  Akt2

H460 SCR

" Akt1 NI

" Akt2

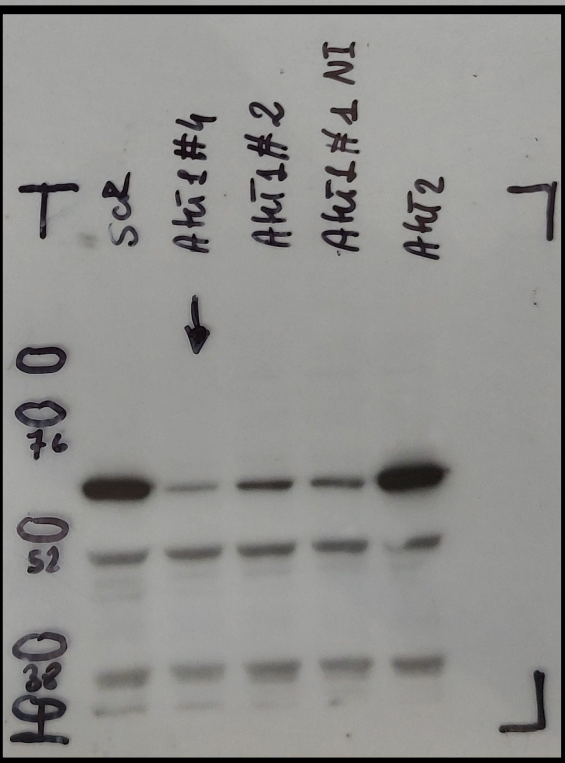

$\alpha$  Akt1

$\alpha$  GSK  
3 $\alpha$ /B

Supplement: S7 File — (ZIP) [file pone.0349359.s007.zip › Figure S1A_left_Akt1 blot .pdf]

T. ESPOSIZIONE

5 min

28.09.11

┌

T

┐

0303

52

1030

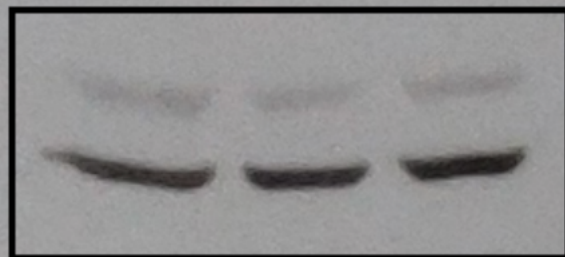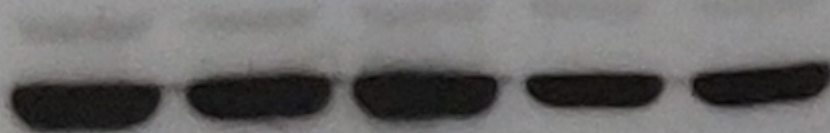

polk  
unipice  
int. ANT  
β. ACTIN  
MOUSE

Supplement: S7 File — (ZIP) [file pone.0349359.s007.zip › Figure S1A_right_Actin blot.pdf]

$\alpha$  Akt2

+

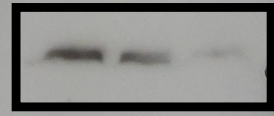

1000  
700  
500

+

H460 SCR  
" Akt1 NI  
" Akt2

1000  
700  
500

+

+

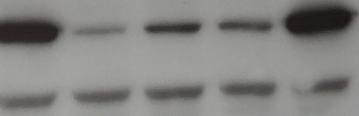

1000  
700  
500

+

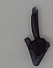

SCR  
Akt1 #4  
Akt1 #2  
Akt1 #1 NI  
Akt2

+



1000  
700  
500

+

$\alpha$  Akt1

$\alpha$  GSK  
3 $\alpha$ /B

+

+

310

310

52

Supplement: S7 File — (ZIP) [file pone.0349359.s007.zip › Figure S1A_right_AKT2 blot.pdf]

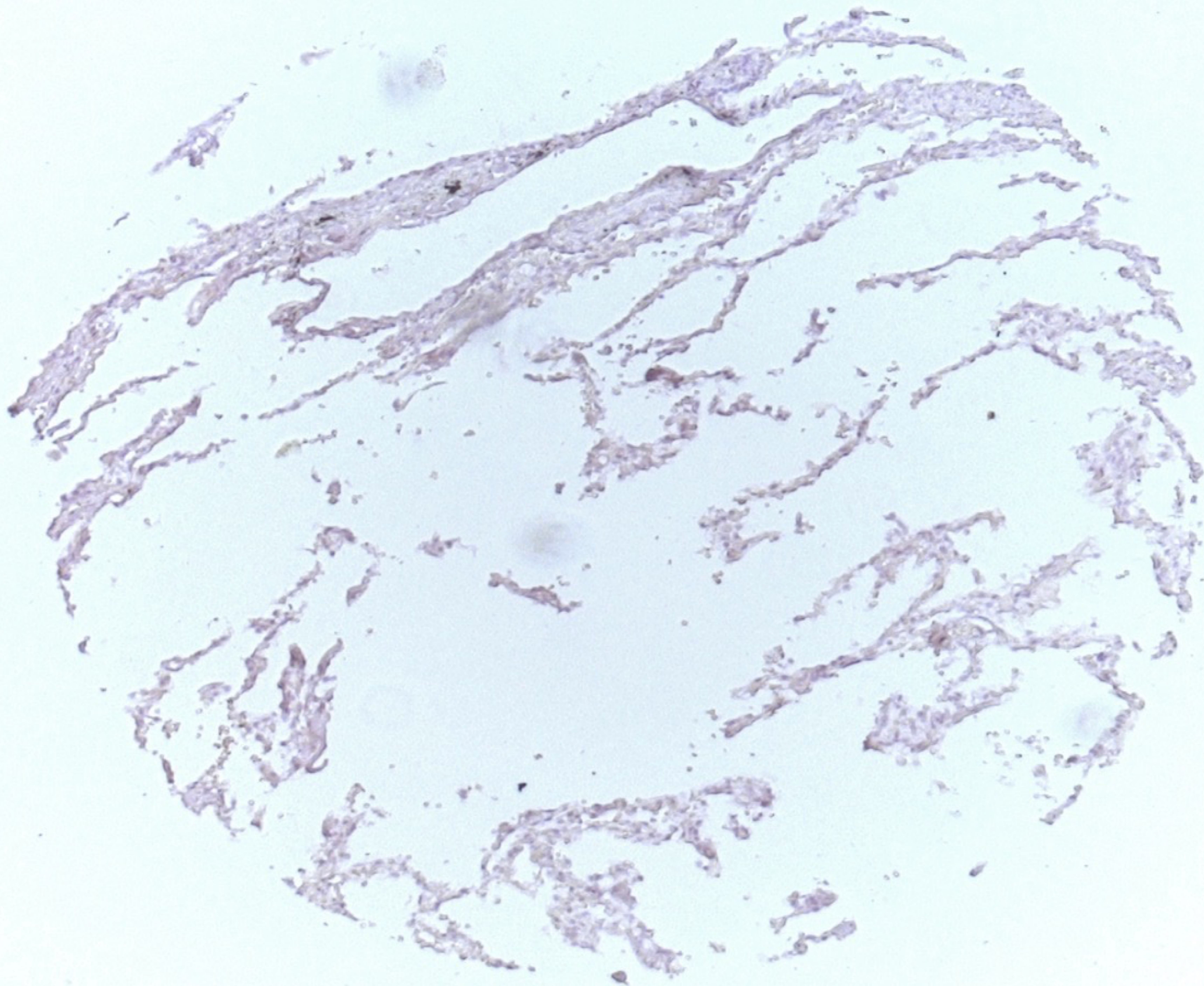

Supplement: S7 File — (ZIP) [file pone.0349359.s007.zip › Figure S1B AKT1 normal 10x.pdf]

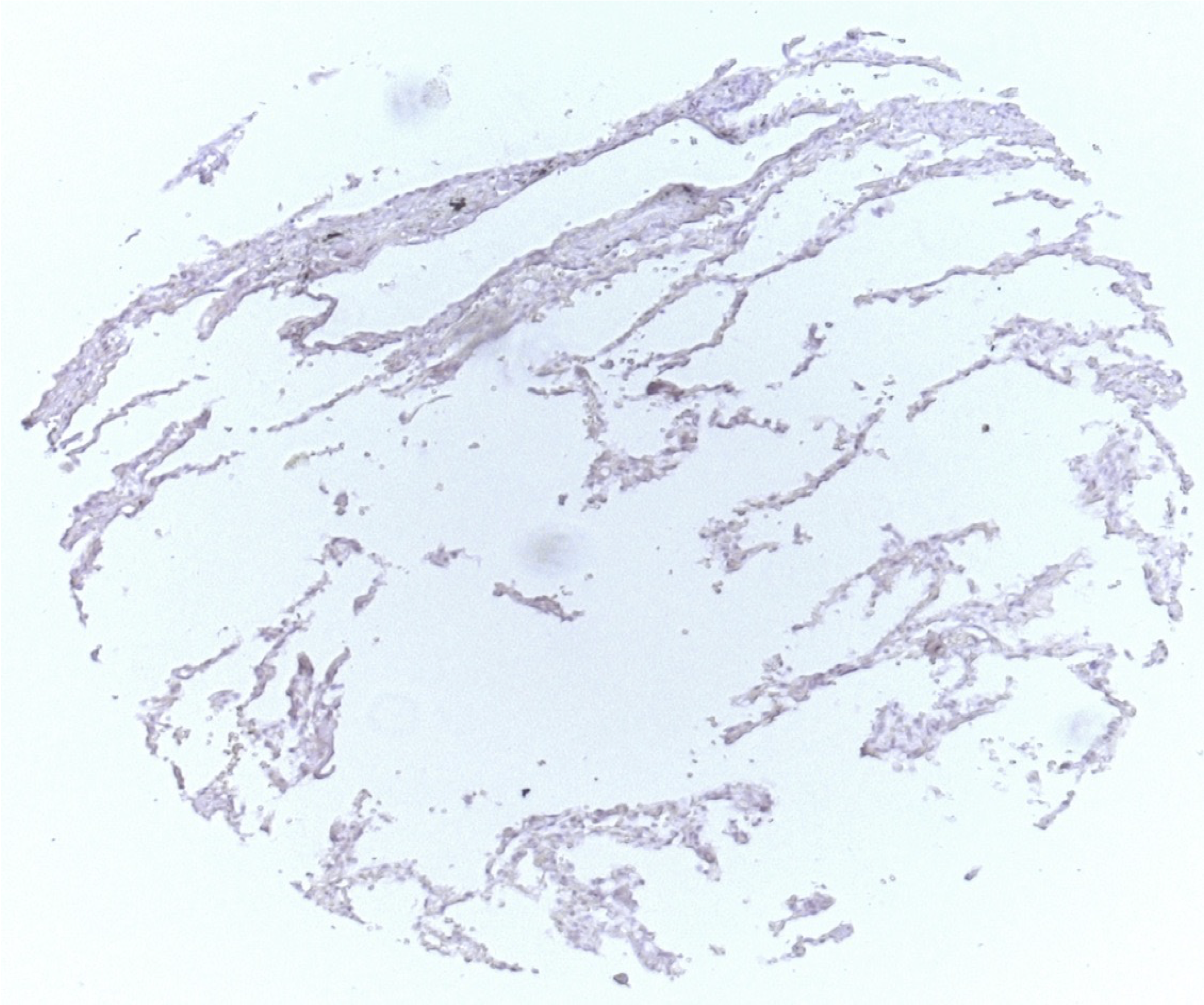

Supplement: S7 File — (ZIP) [file pone.0349359.s007.zip › Figure S1B AKT1 normal 10x.tiff]

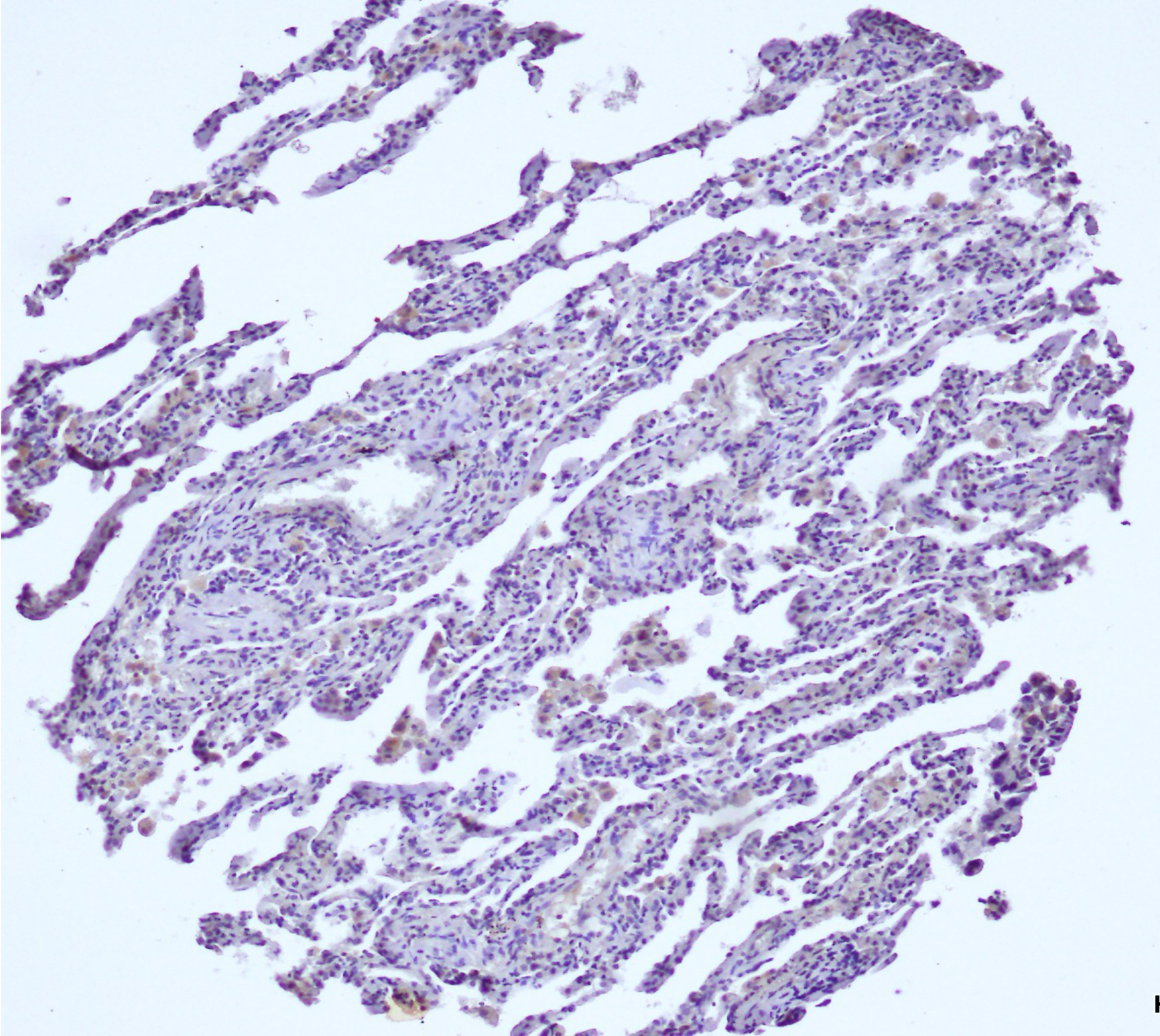

Supplement: S7 File — (ZIP) [file pone.0349359.s007.zip › Figure S1B AKT2 normal 10x.pdf]

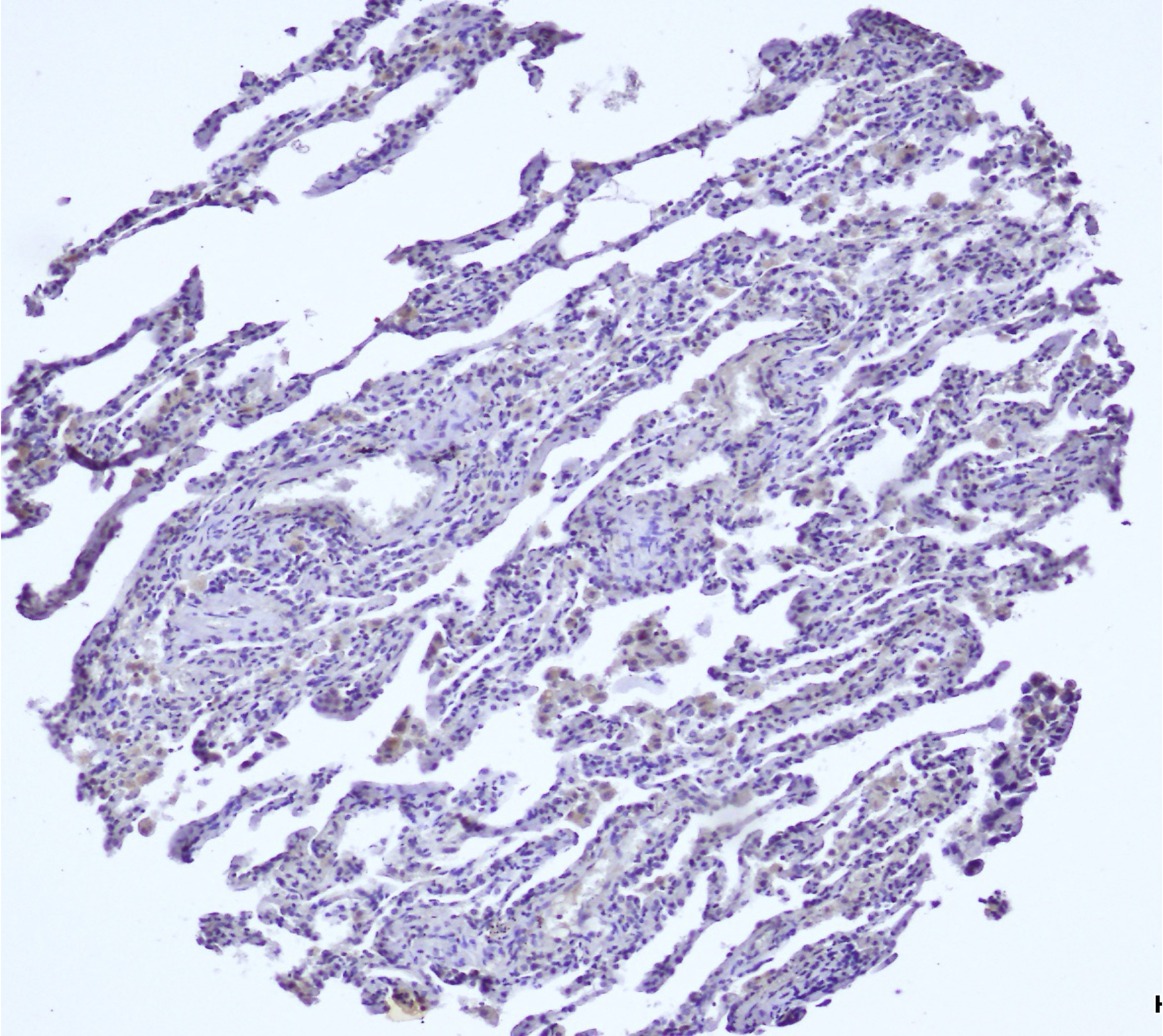

Supplement: S7 File — (ZIP) [file pone.0349359.s007.zip › Figure S1B AKT2 normal 10x.tiff]

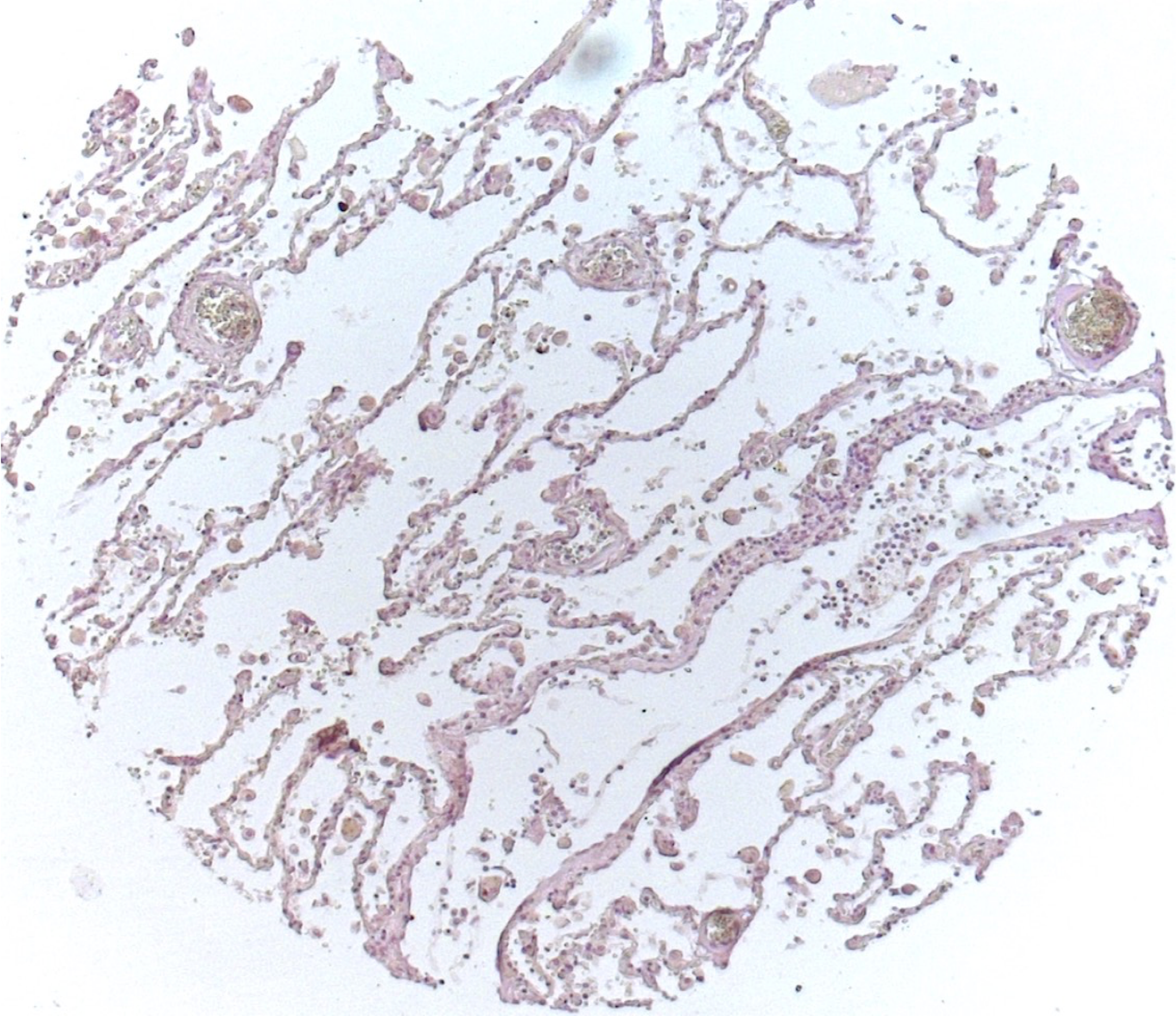

Supplement: S7 File — (ZIP) [file pone.0349359.s007.zip › Figure S1B pAKT normal 10x.pdf]

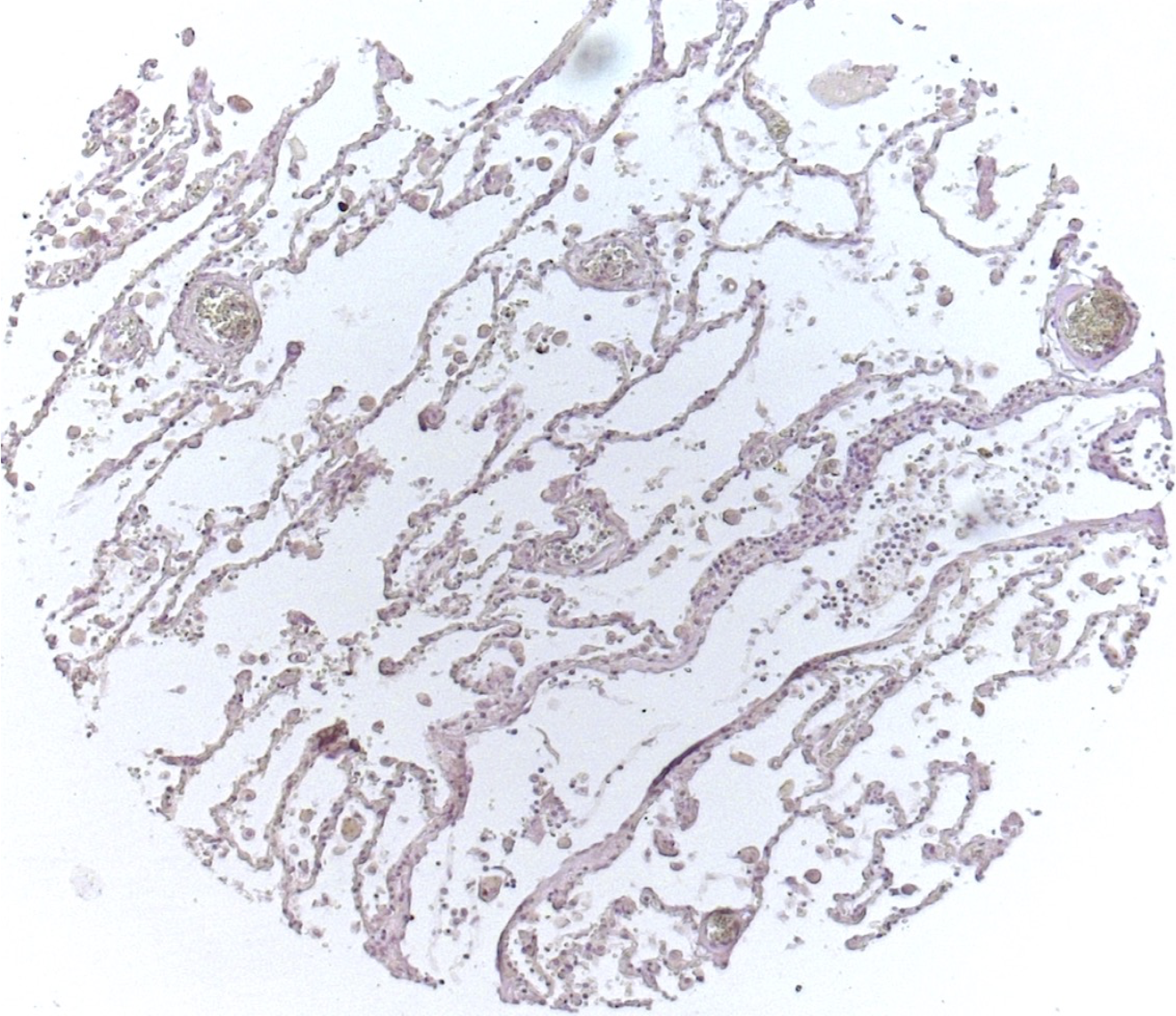

Supplement: S7 File — (ZIP) [file pone.0349359.s007.zip › Figure S1B pAKT normal 10x.tiff]

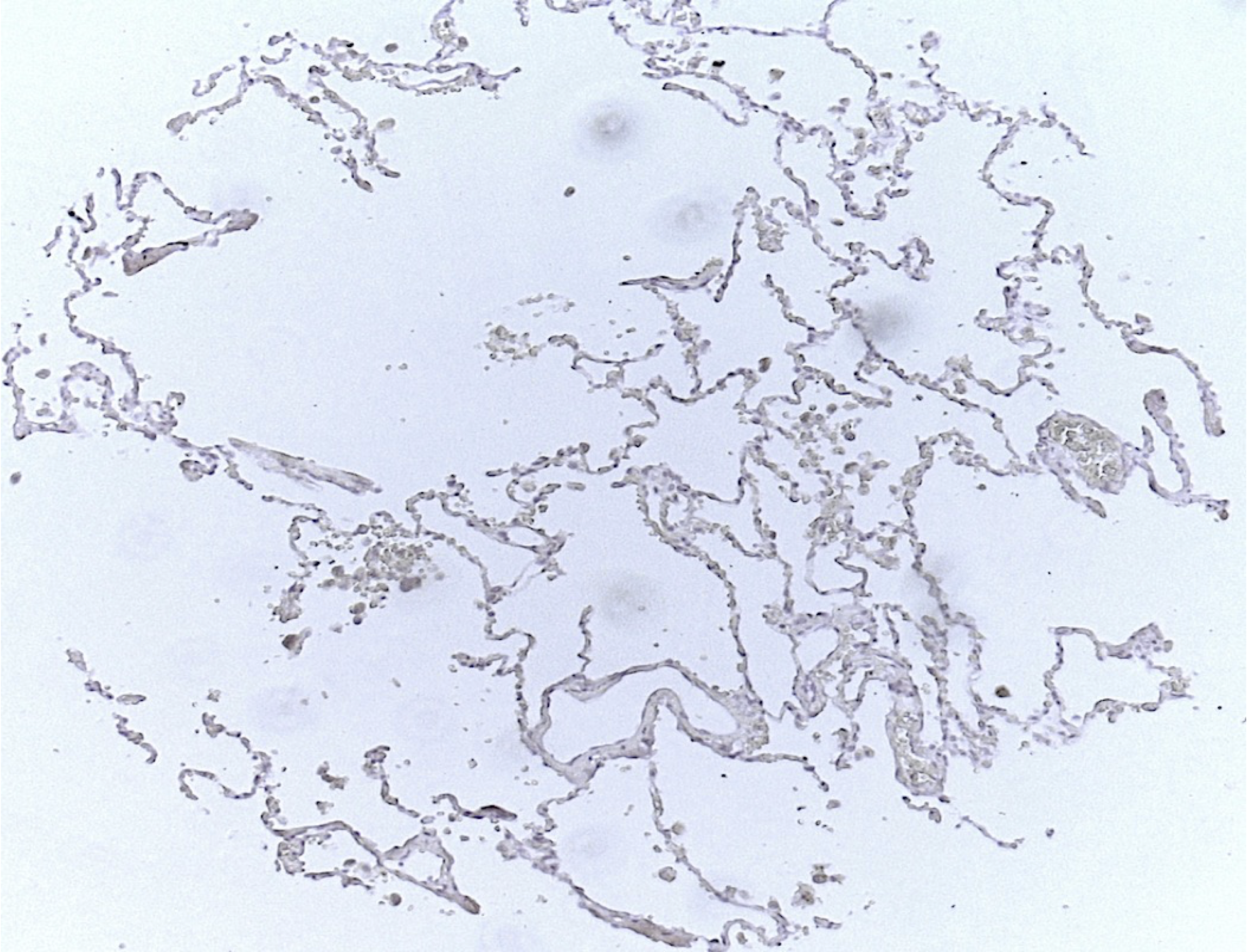

Supplement: S7 File — (ZIP) [file pone.0349359.s007.zip › Figure S1B PI3KCA normal 10x .tiff]

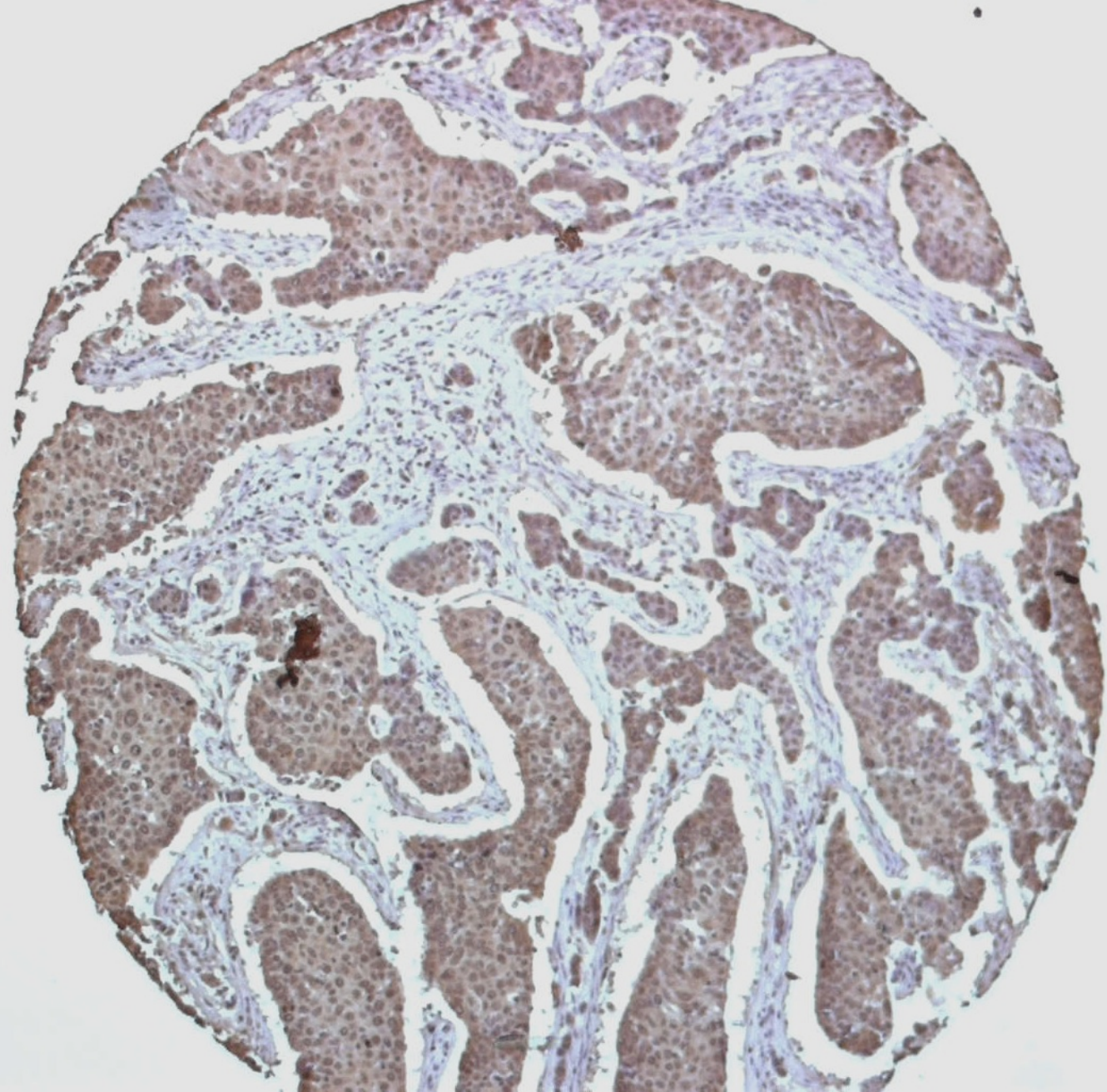

Supplement: S8 File — (ZIP) [file pone.0349359.s008.zip › Figure S2A AKT1 SCC right 10x.pdf]

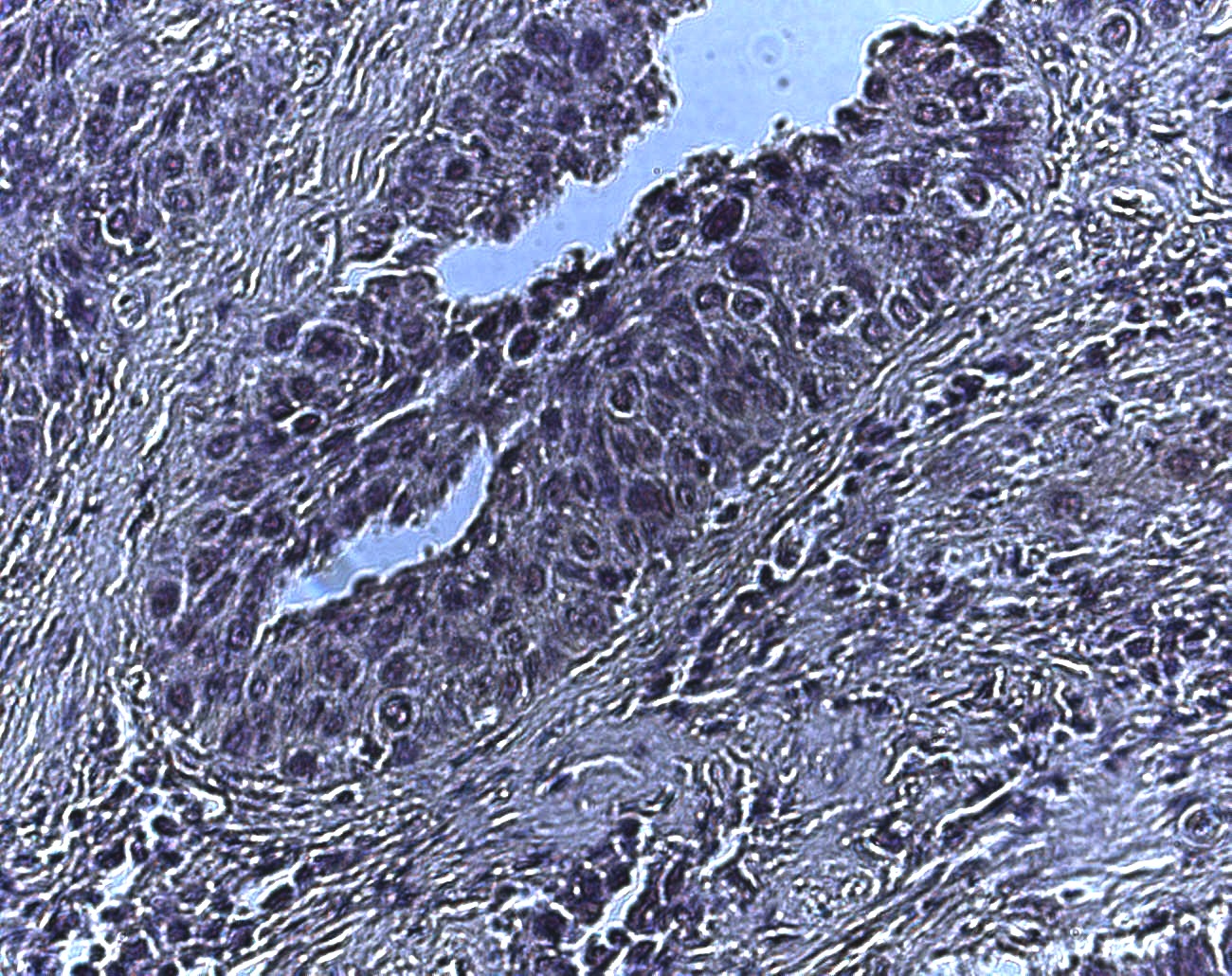

Supplement: S8 File — (ZIP) [file pone.0349359.s008.zip › Figure S2A AKT1 SCC left 40x.jpg]

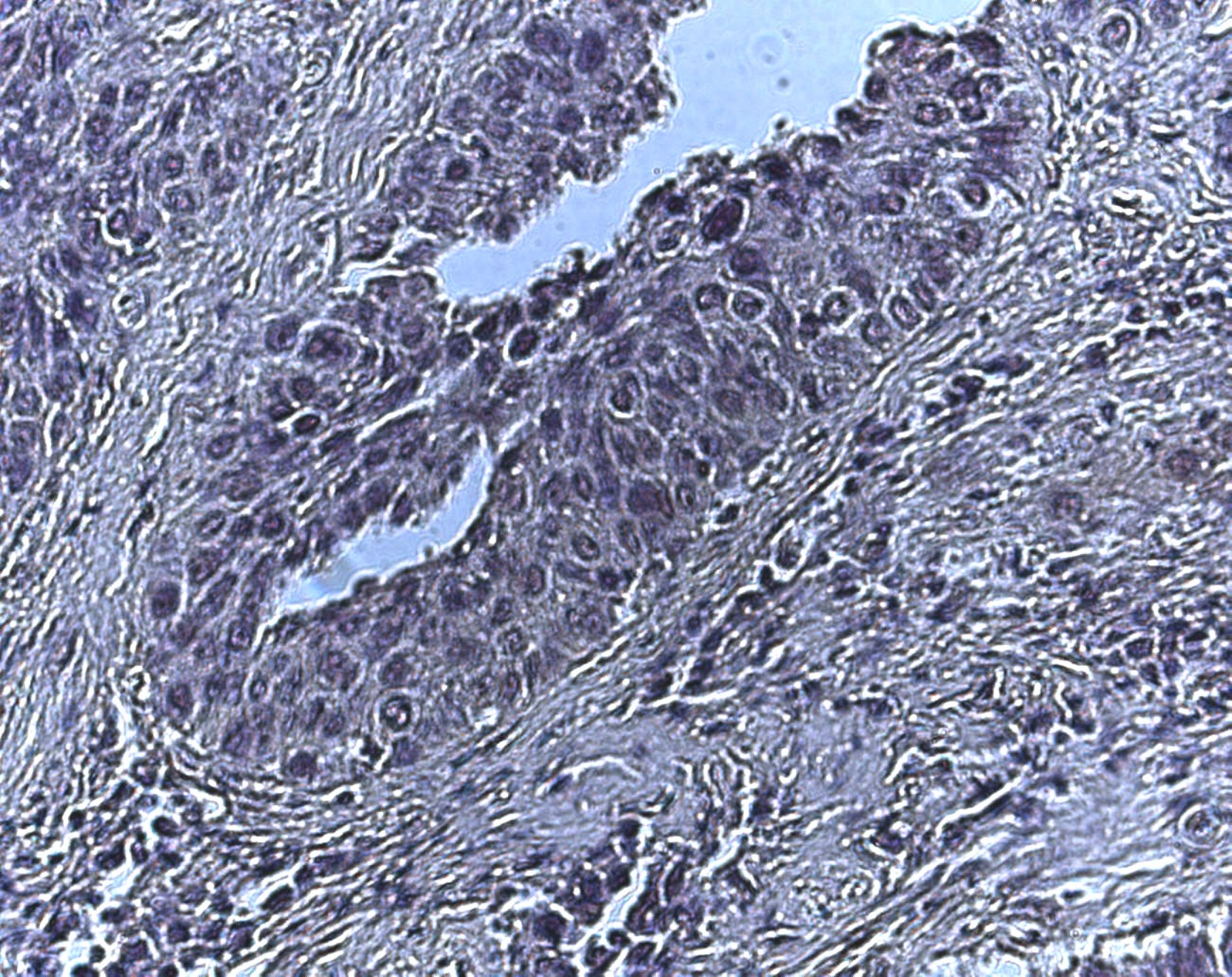

Supplement: S8 File — (ZIP) [file pone.0349359.s008.zip › Figure S2A AKT1 SCC left 40x.pdf]

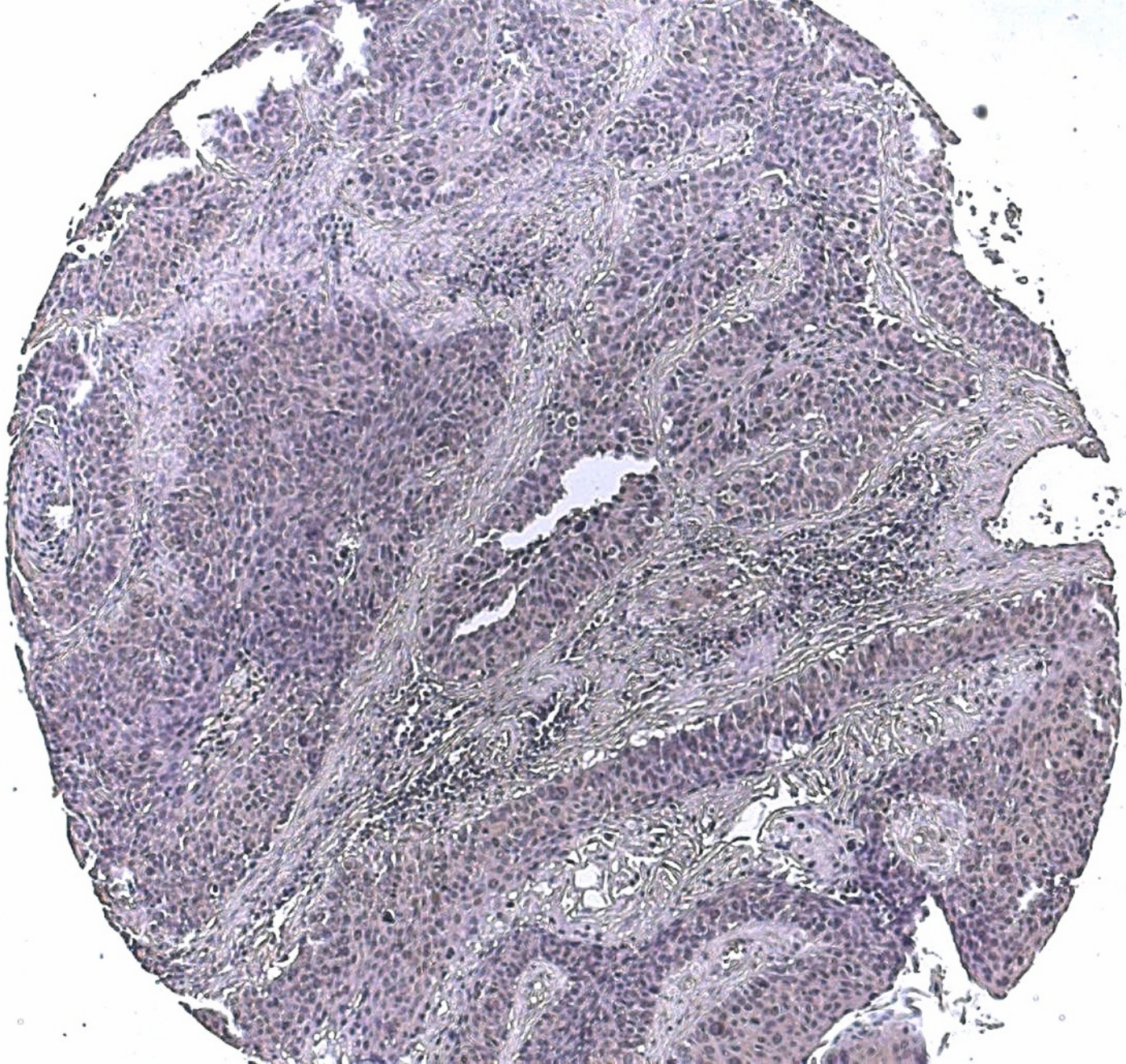

Supplement: S8 File — (ZIP) [file pone.0349359.s008.zip › Figure S2A AKT1 SCC left 10x.pdf]

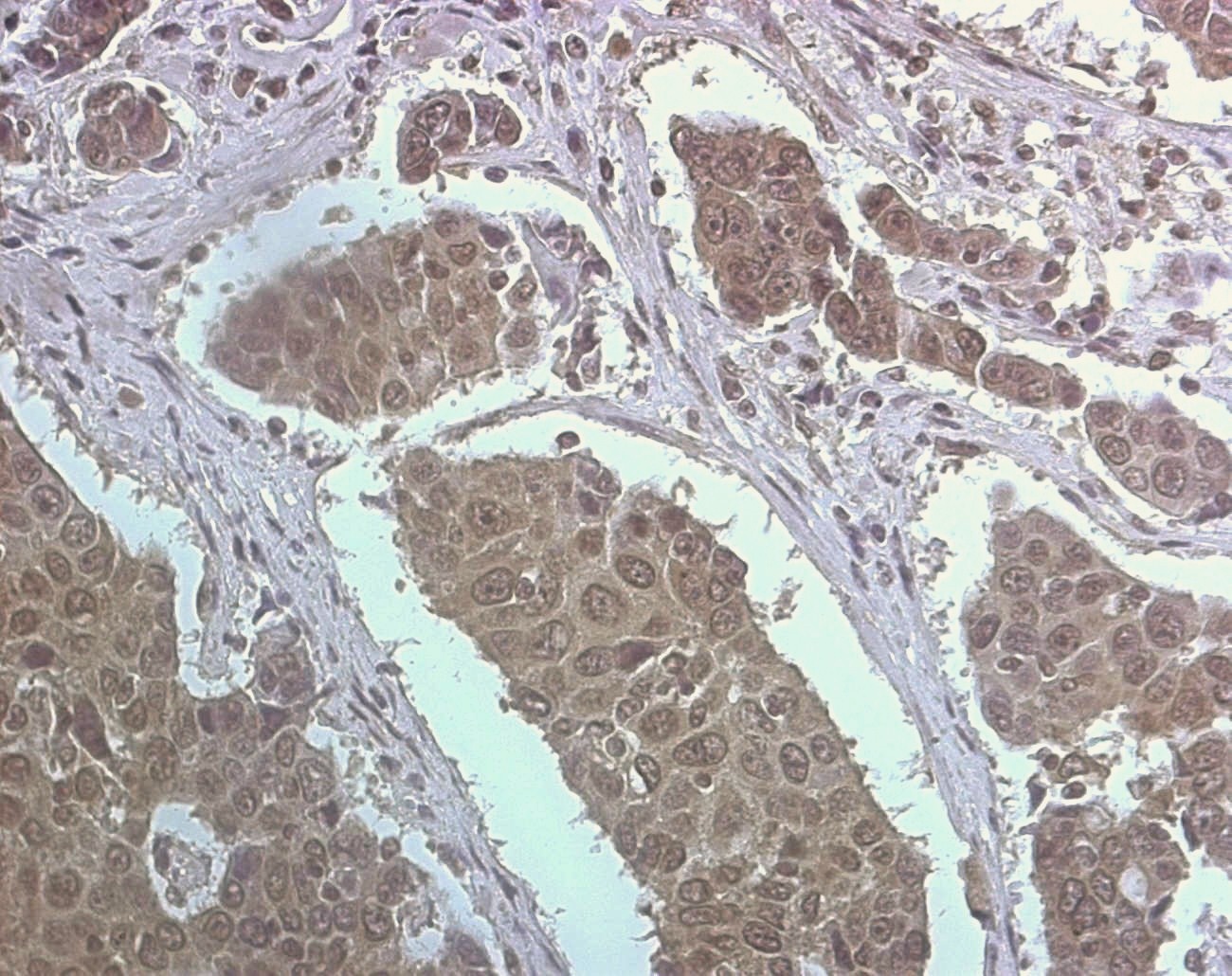

Supplement: S8 File — (ZIP) [file pone.0349359.s008.zip › Figure S2A AKT1 SCC right 40x.jpg]

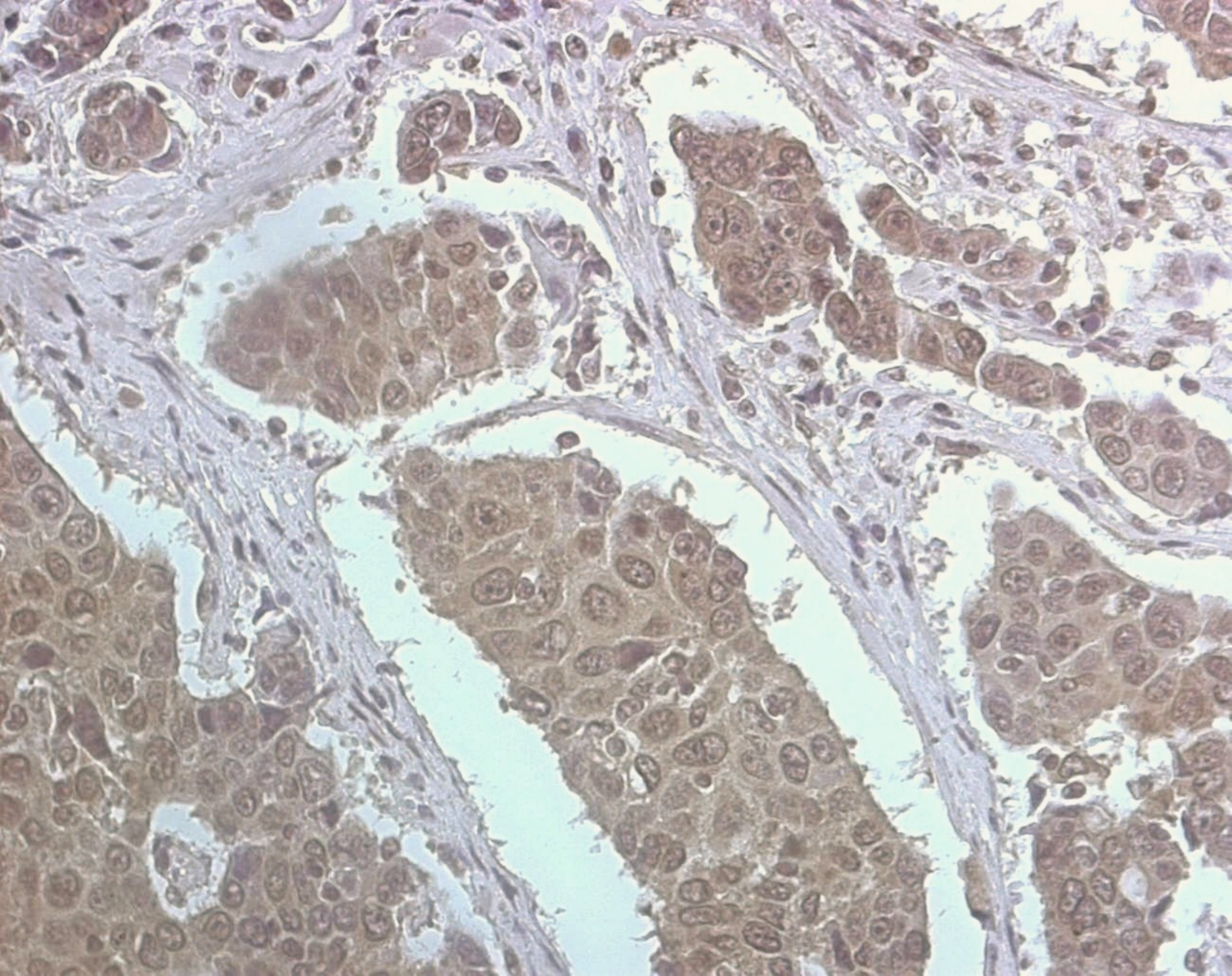

Supplement: S8 File — (ZIP) [file pone.0349359.s008.zip › Figure S2A AKT1 SCC right 40x.pdf]

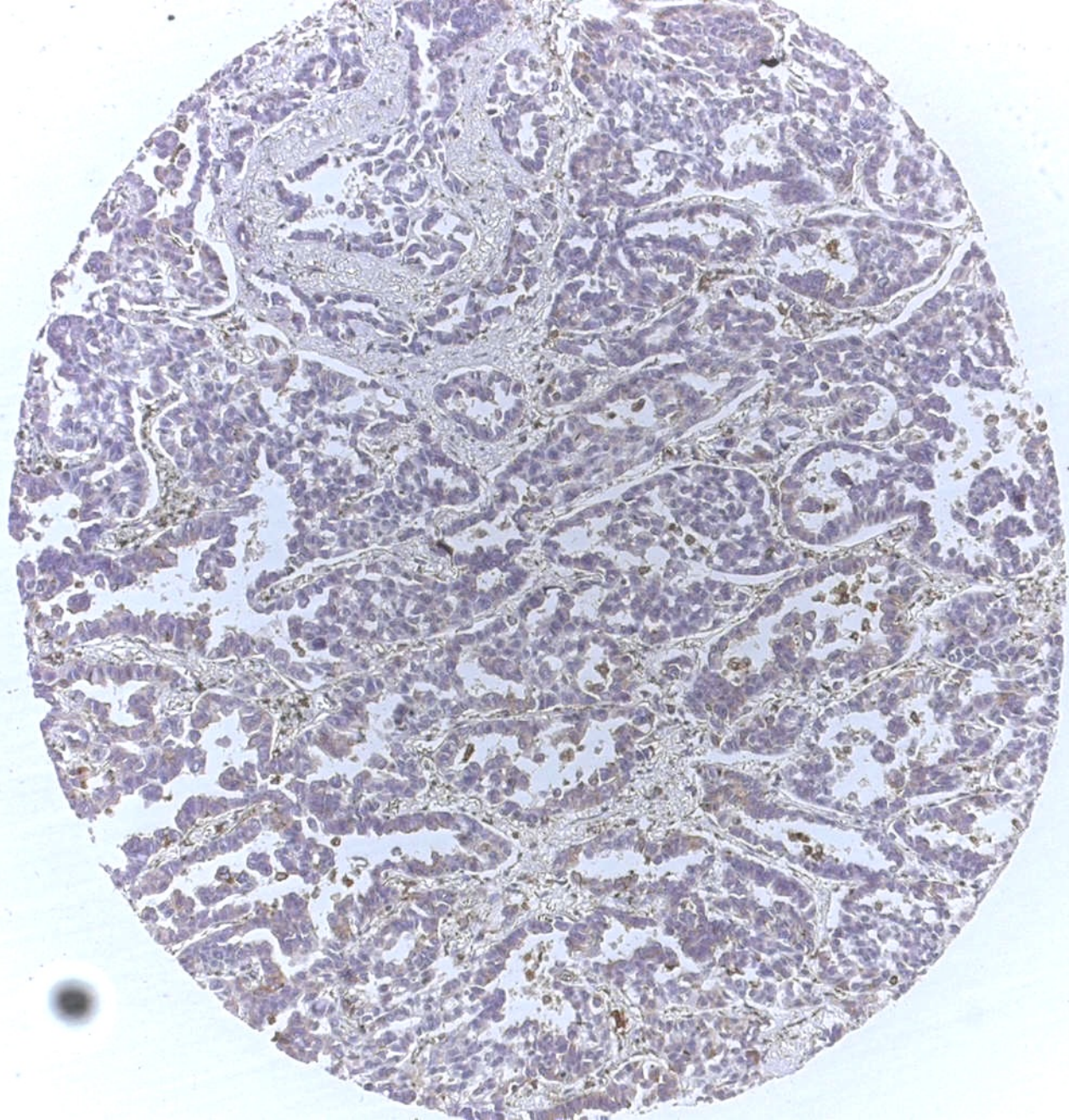

Supplement: S8 File — (ZIP) [file pone.0349359.s008.zip › Figure S2B AKT1 ADC left 10x.pdf]

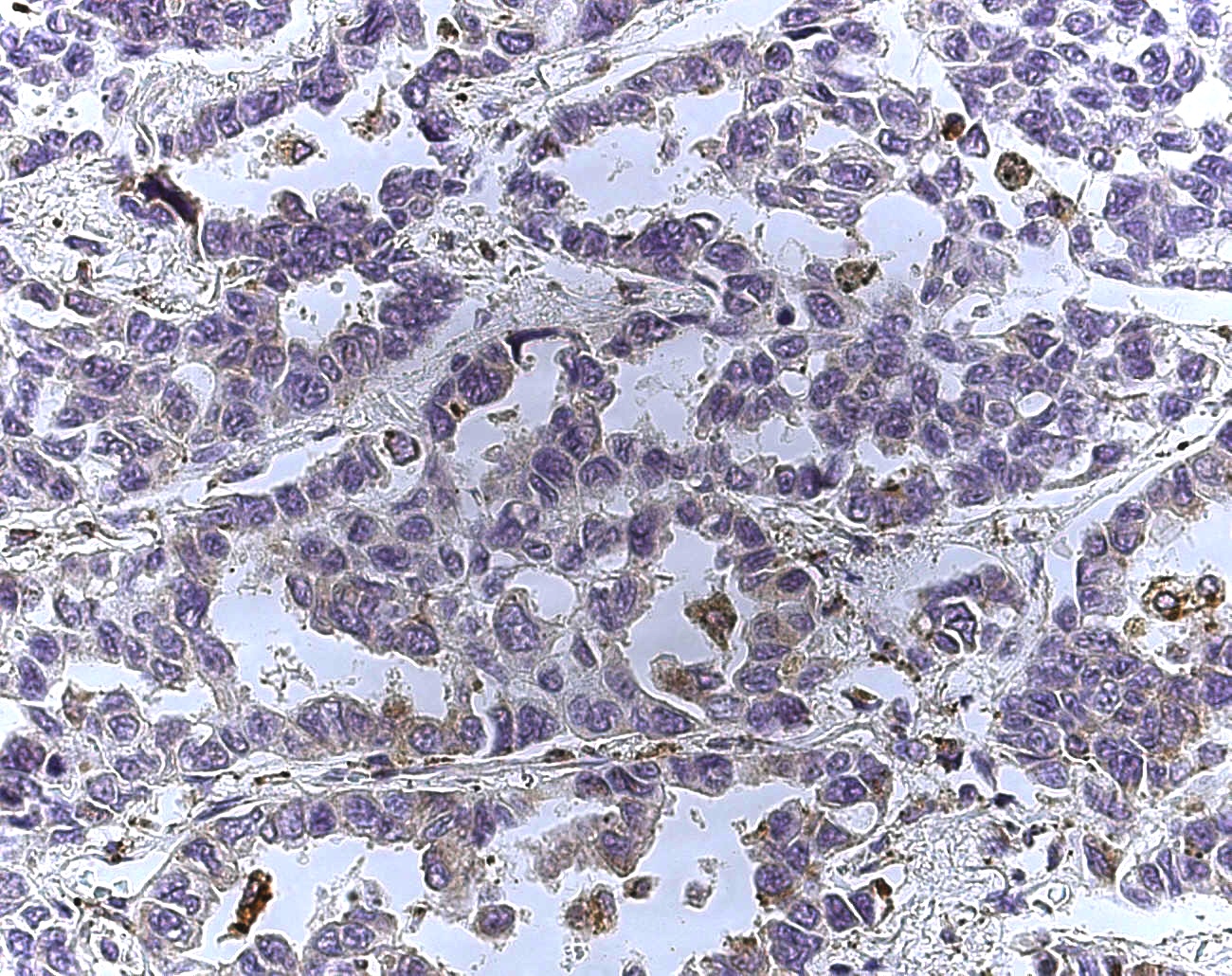

Supplement: S8 File — (ZIP) [file pone.0349359.s008.zip › Figure S2B AKT1 ADC left40x.jpg]

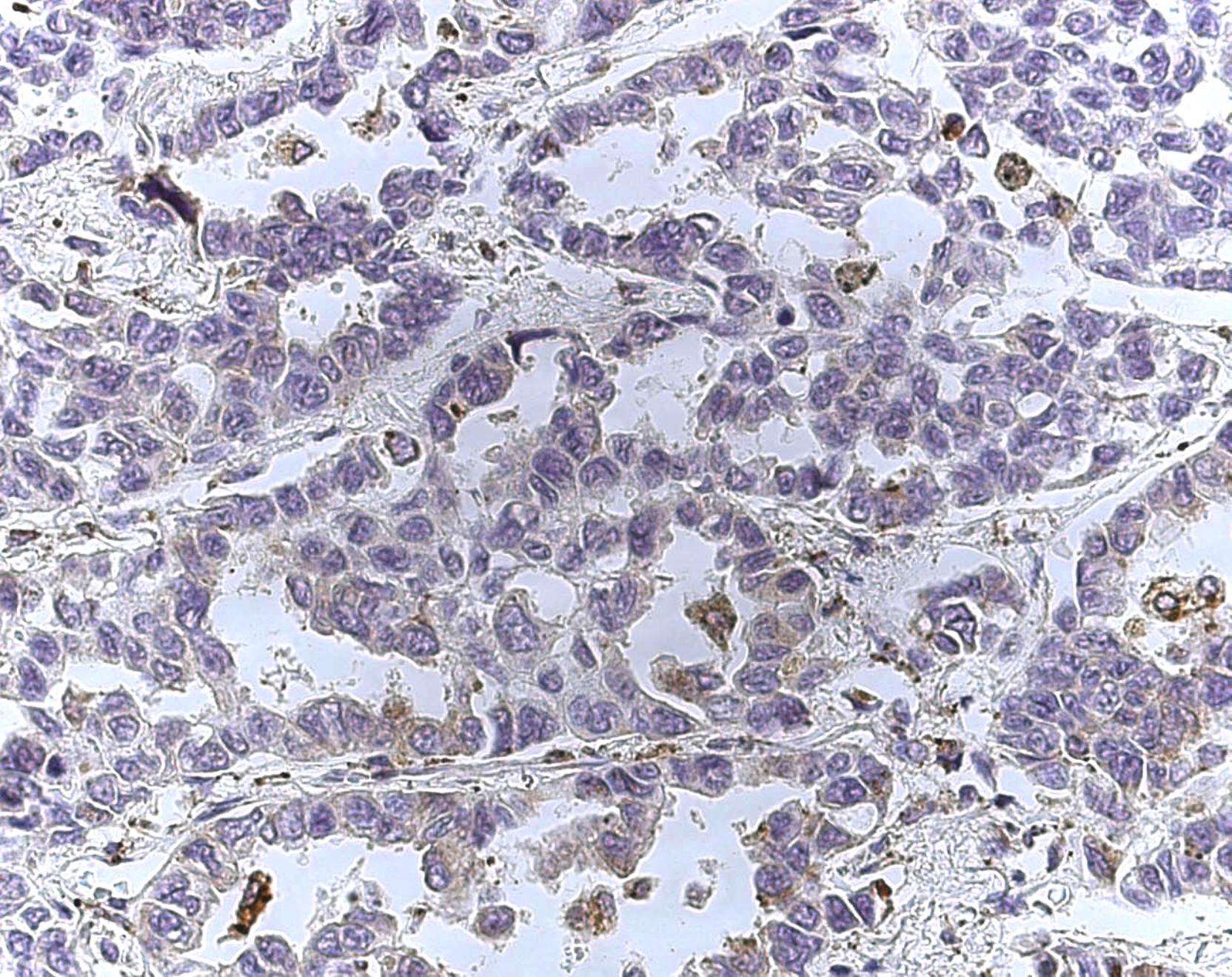

Supplement: S8 File — (ZIP) [file pone.0349359.s008.zip › Figure S2B AKT1 ADC left40x.pdf]

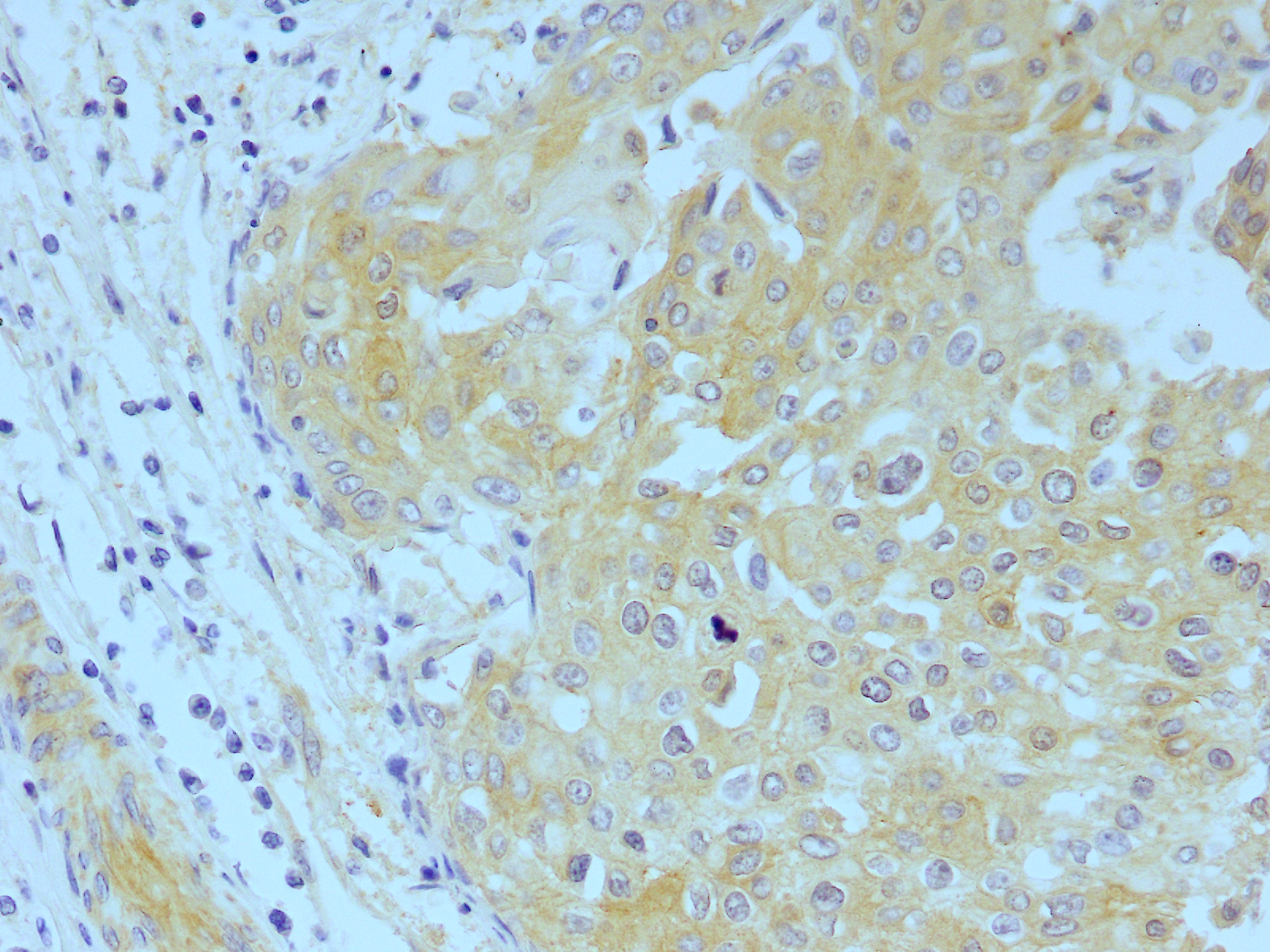

Supplement: S8 File — (ZIP) [file pone.0349359.s008.zip › FigureS2A AKT1 (++) SCC 40x.pdf]

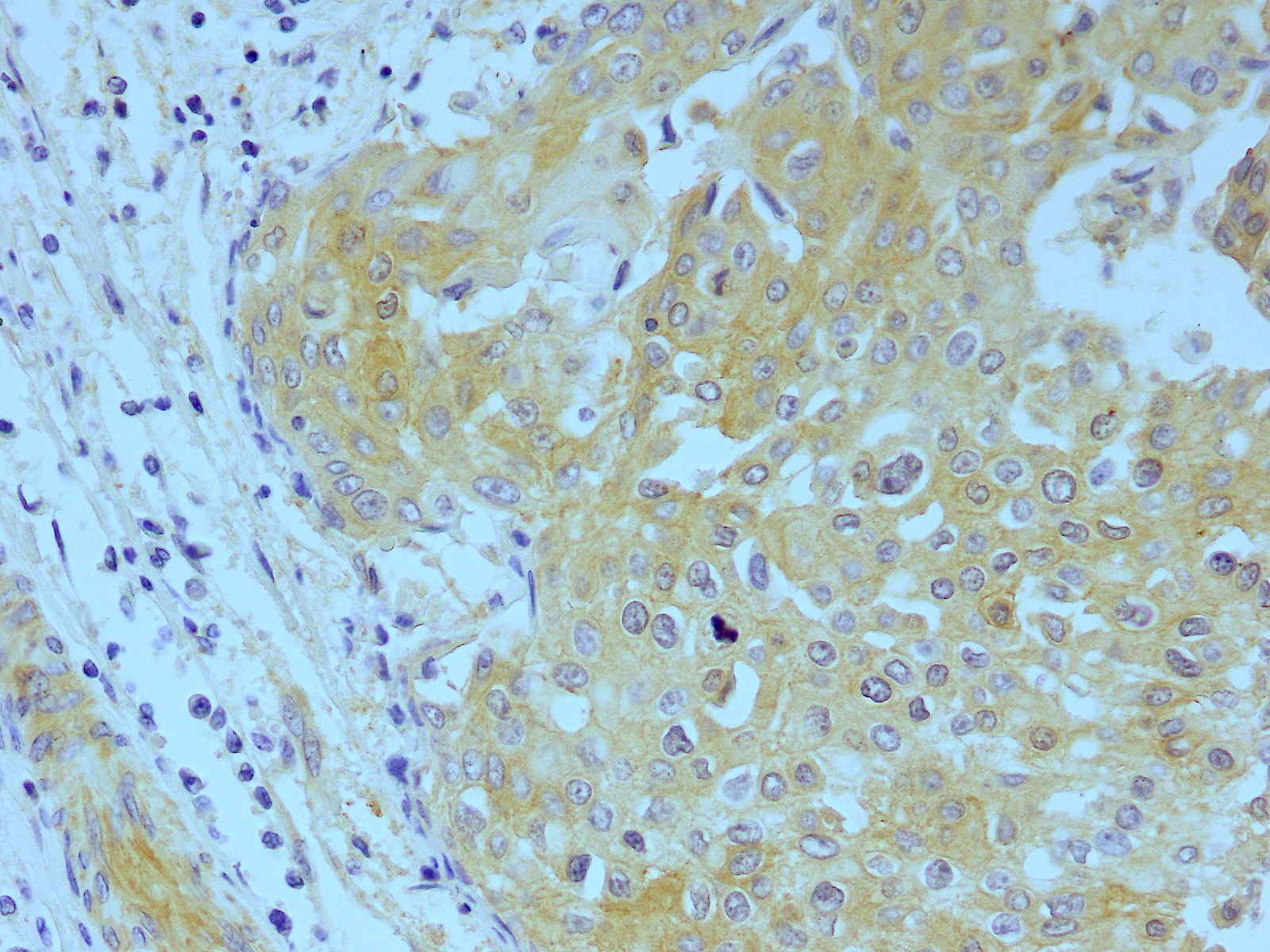

Supplement: S8 File — (ZIP) [file pone.0349359.s008.zip › FigureS2A AKT1 (++) SCC 40x.TIF]

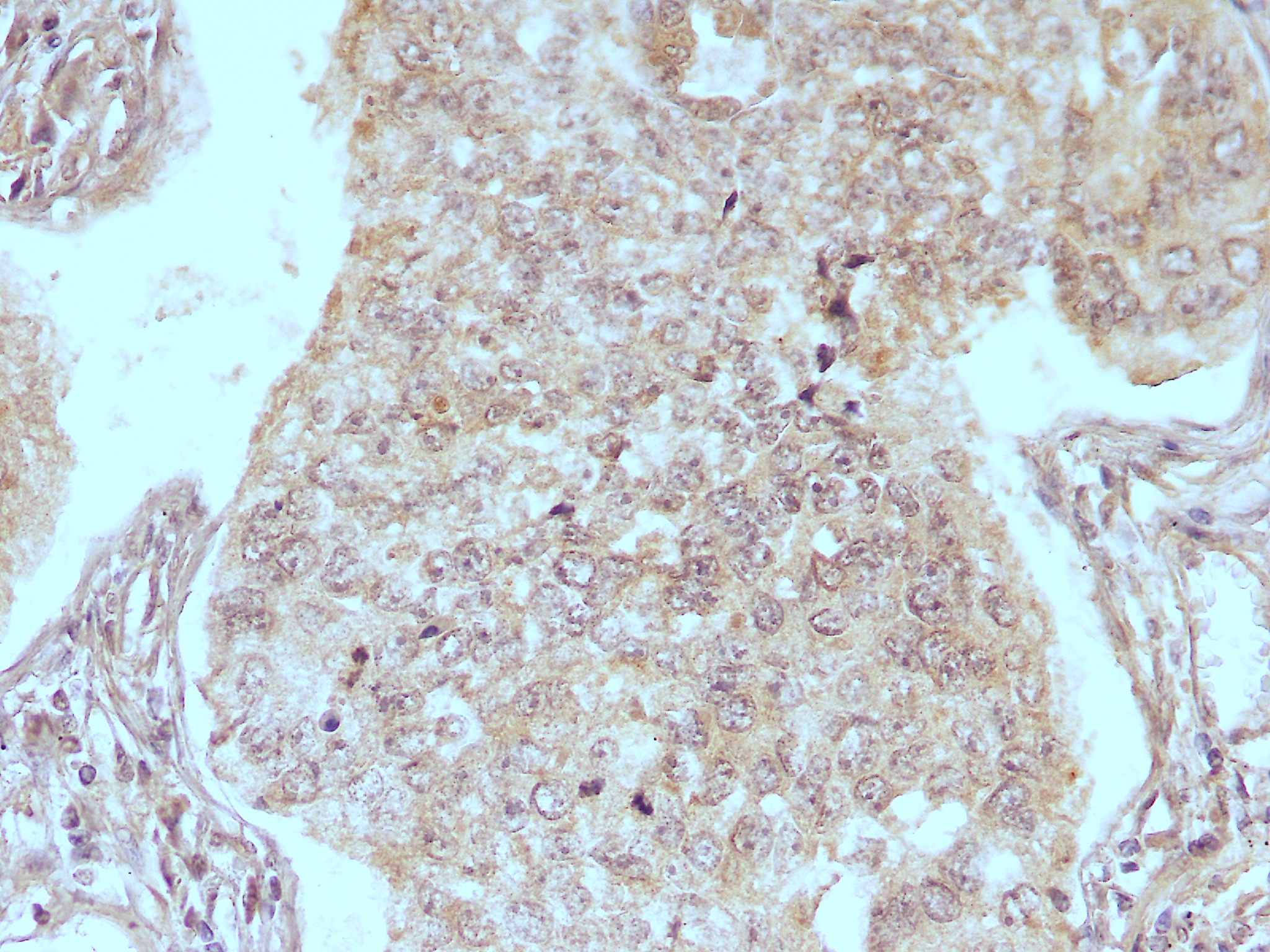

Supplement: S8 File — (ZIP) [file pone.0349359.s008.zip › FigureS2A AKT1(+) SCC 40x.pdf]

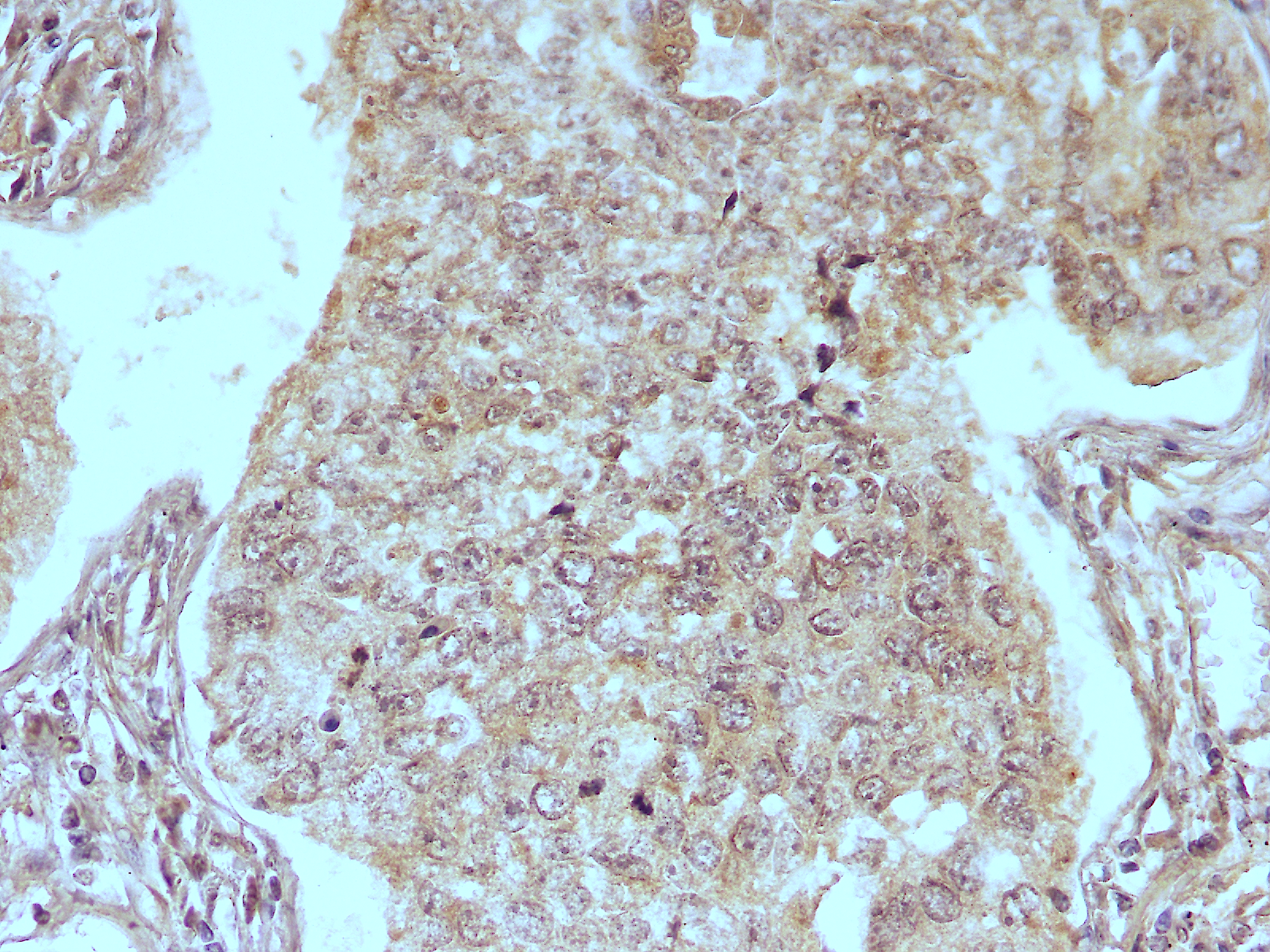

Supplement: S8 File — (ZIP) [file pone.0349359.s008.zip › FigureS2A AKT1(+) SCC 40x.TIF]

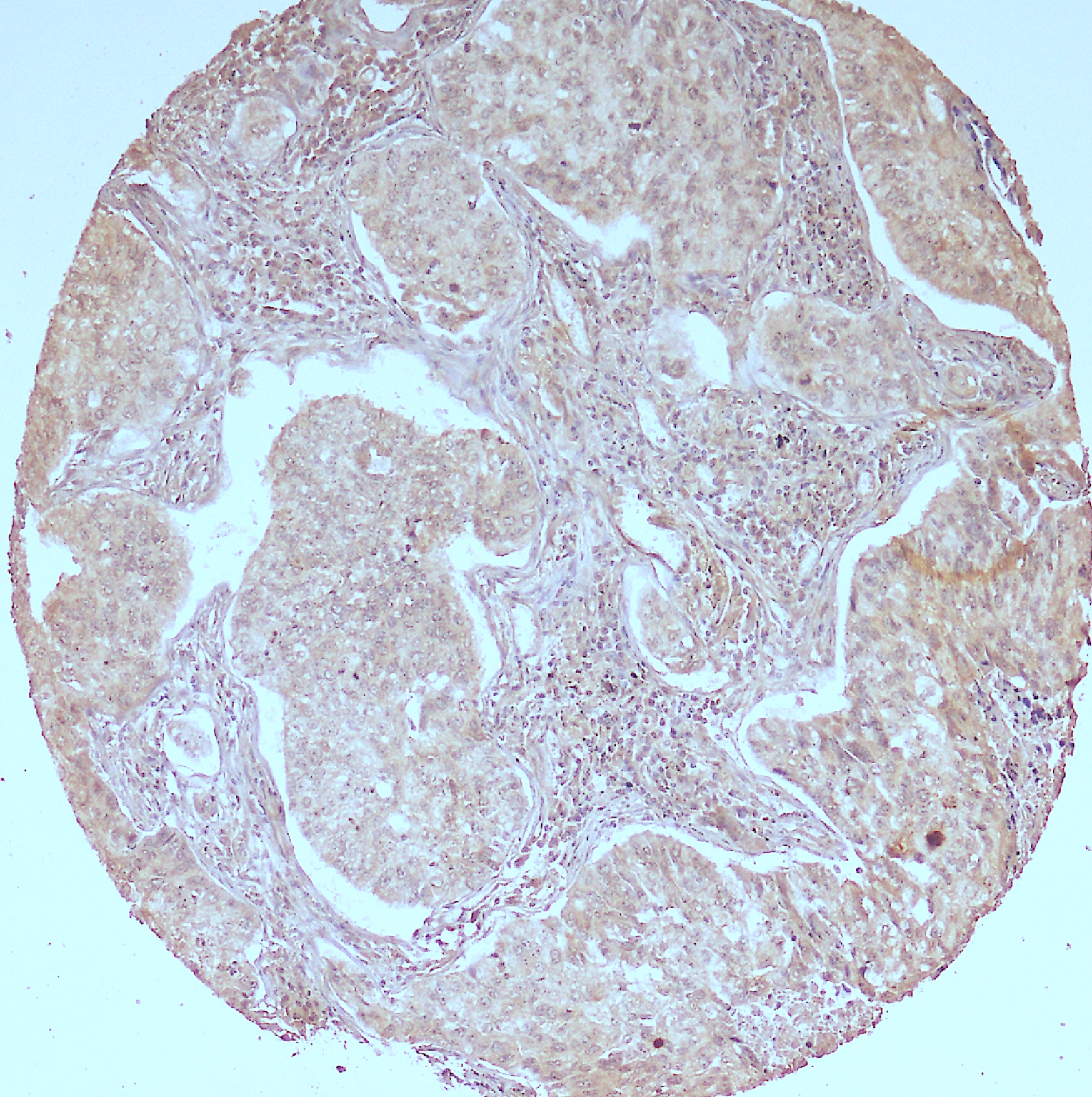

Supplement: S8 File — (ZIP) [file pone.0349359.s008.zip › FigureS2A AKT1(+)SCC 10x.pdf]

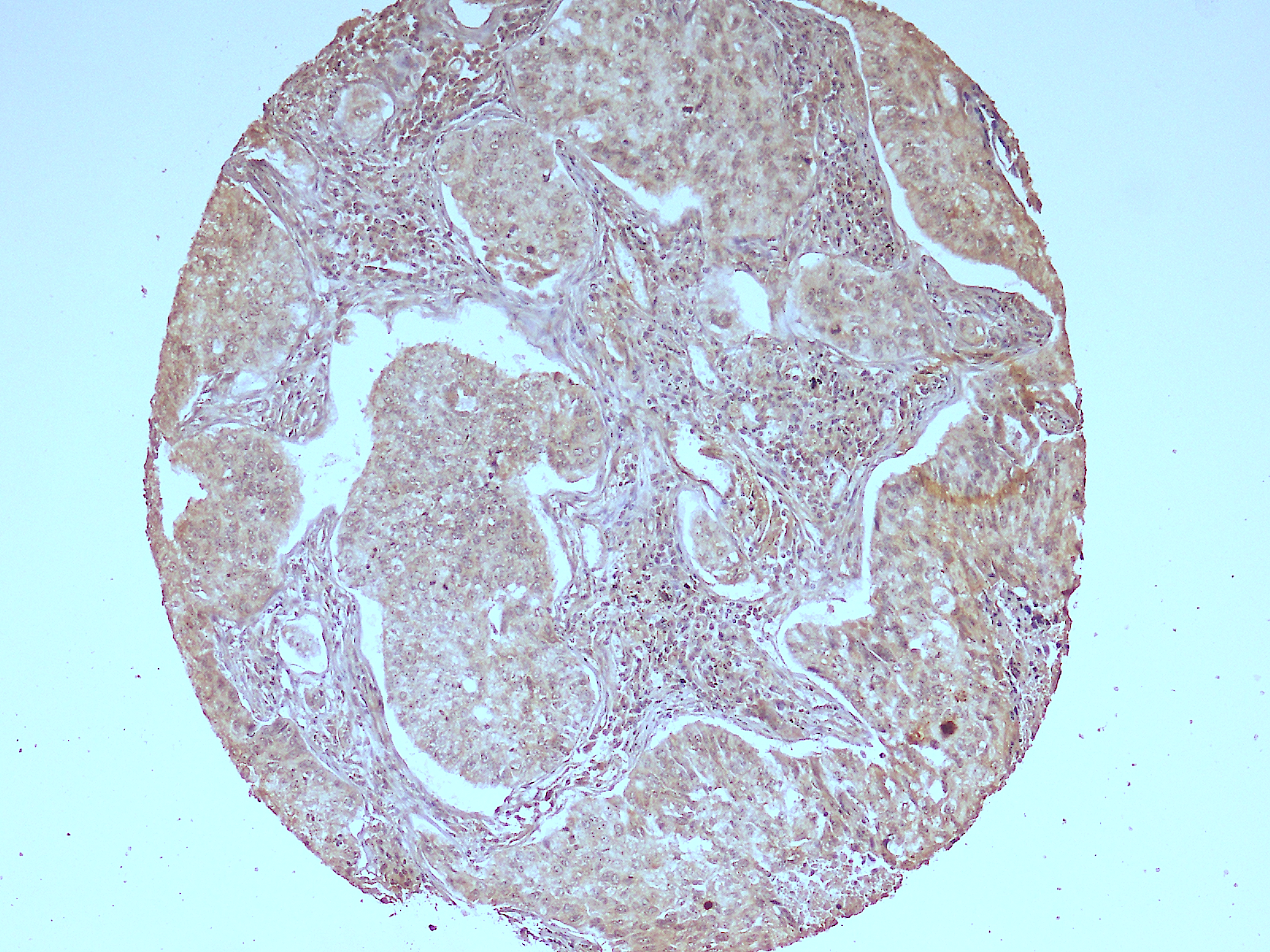

Supplement: S8 File — (ZIP) [file pone.0349359.s008.zip › FigureS2A AKT1(+)SCC 10x.TIF]

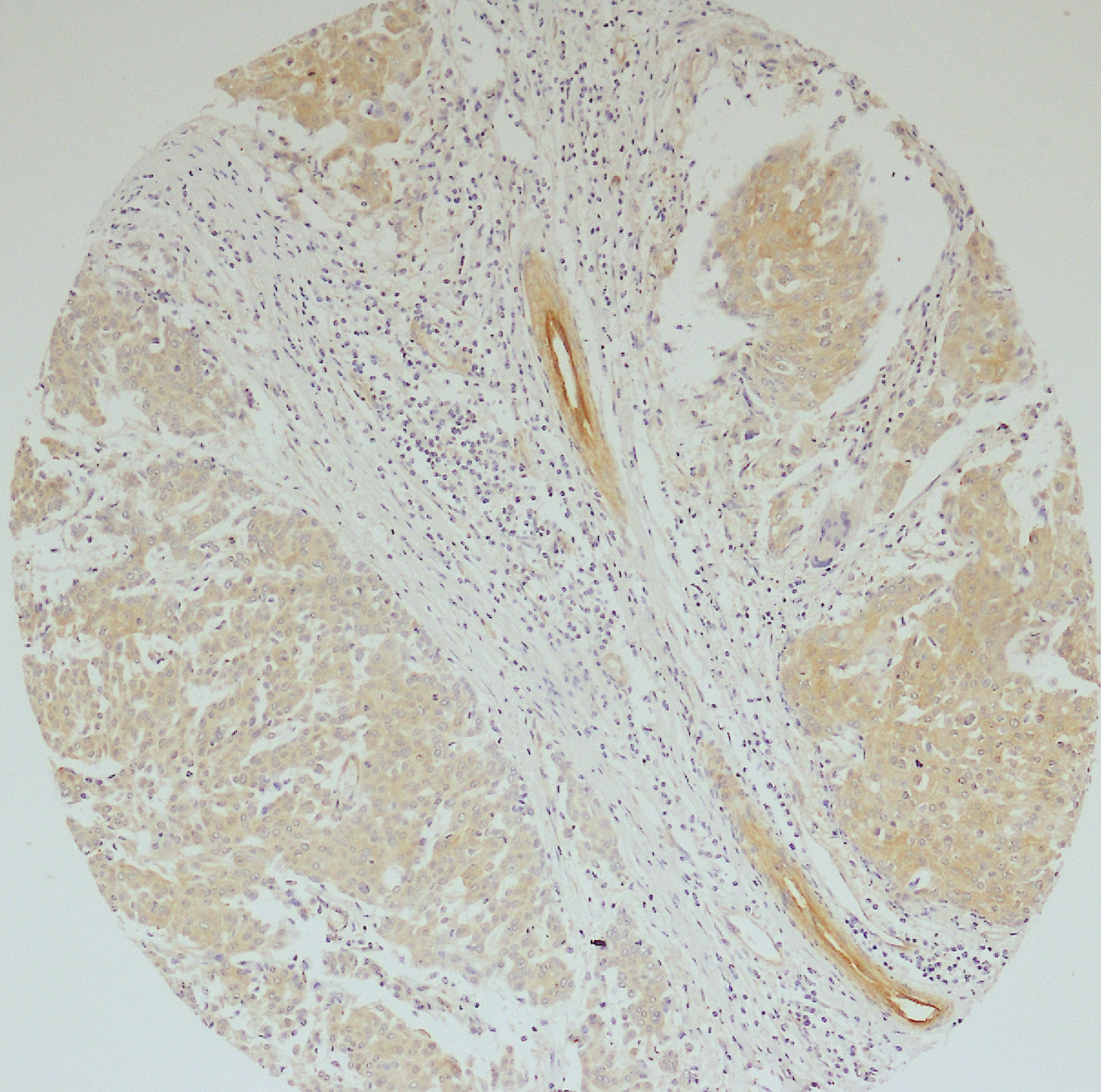

Supplement: S8 File — (ZIP) [file pone.0349359.s008.zip › FigureS2A AKT1(++) SCC 10x.pdf]

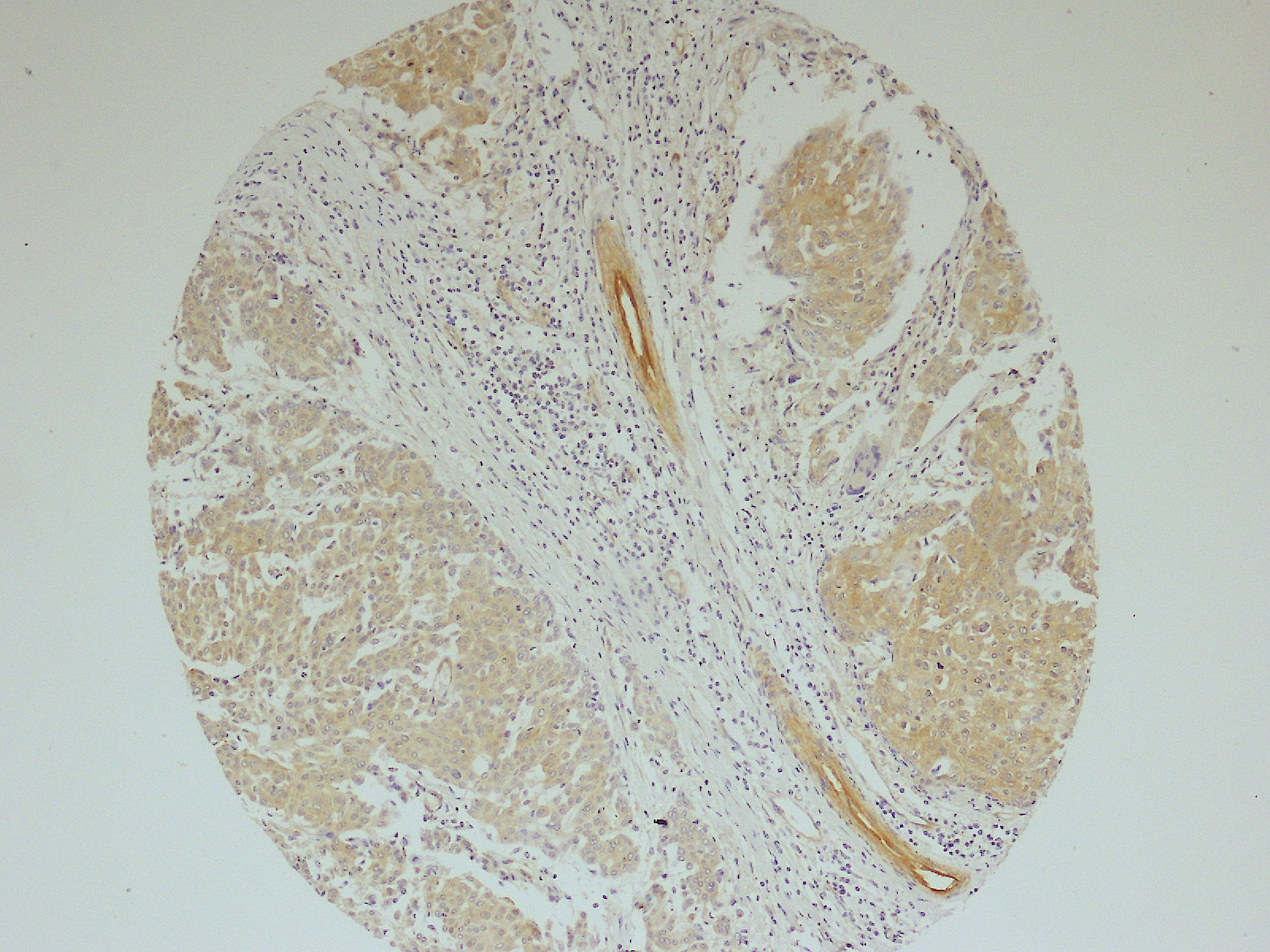

Supplement: S8 File — (ZIP) [file pone.0349359.s008.zip › FigureS2A AKT1(++) SCC 10x.TIF]

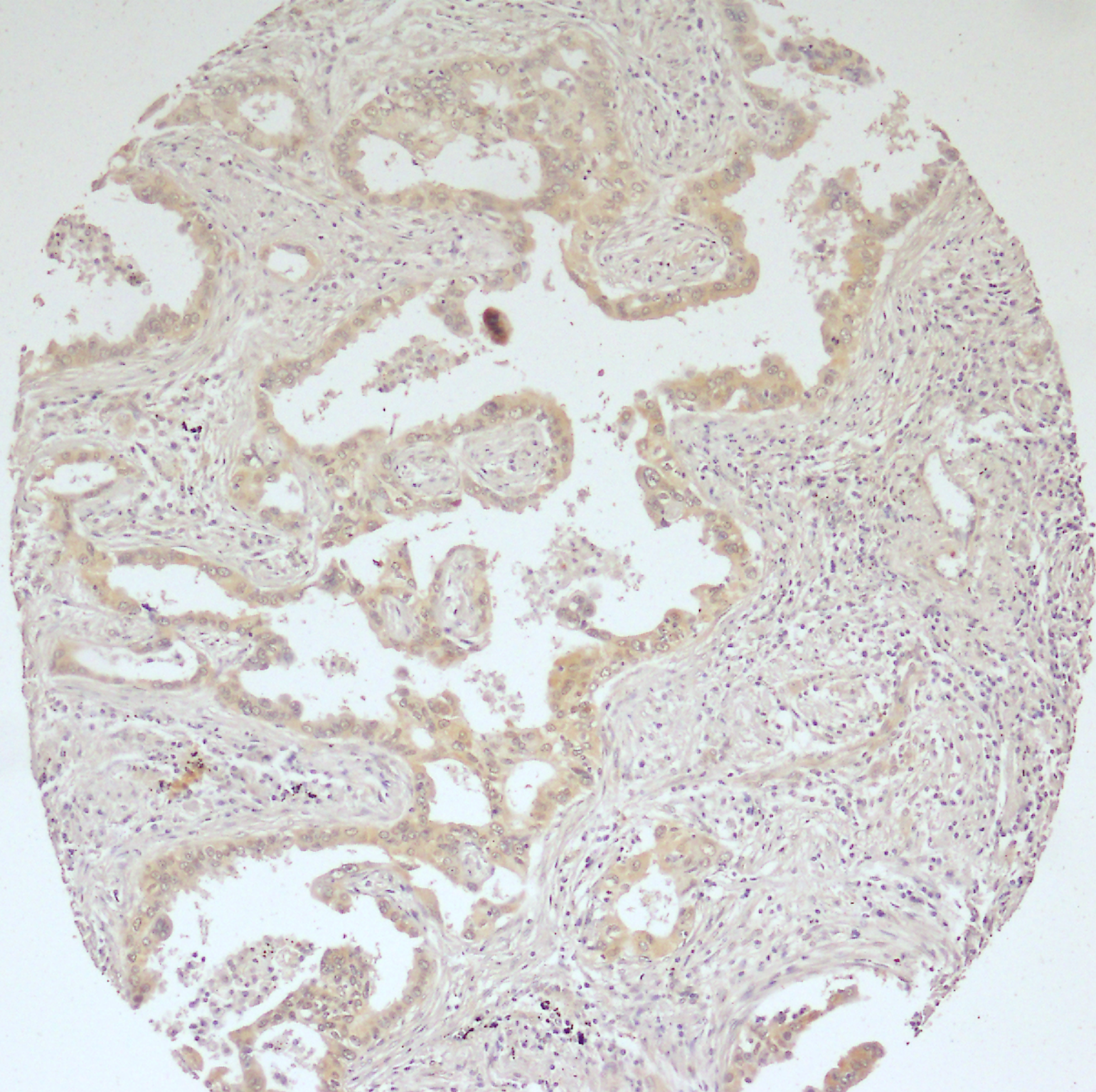

Supplement: S8 File — (ZIP) [file pone.0349359.s008.zip › FigureS2B AKT1 (+) ADC 10x.pdf]

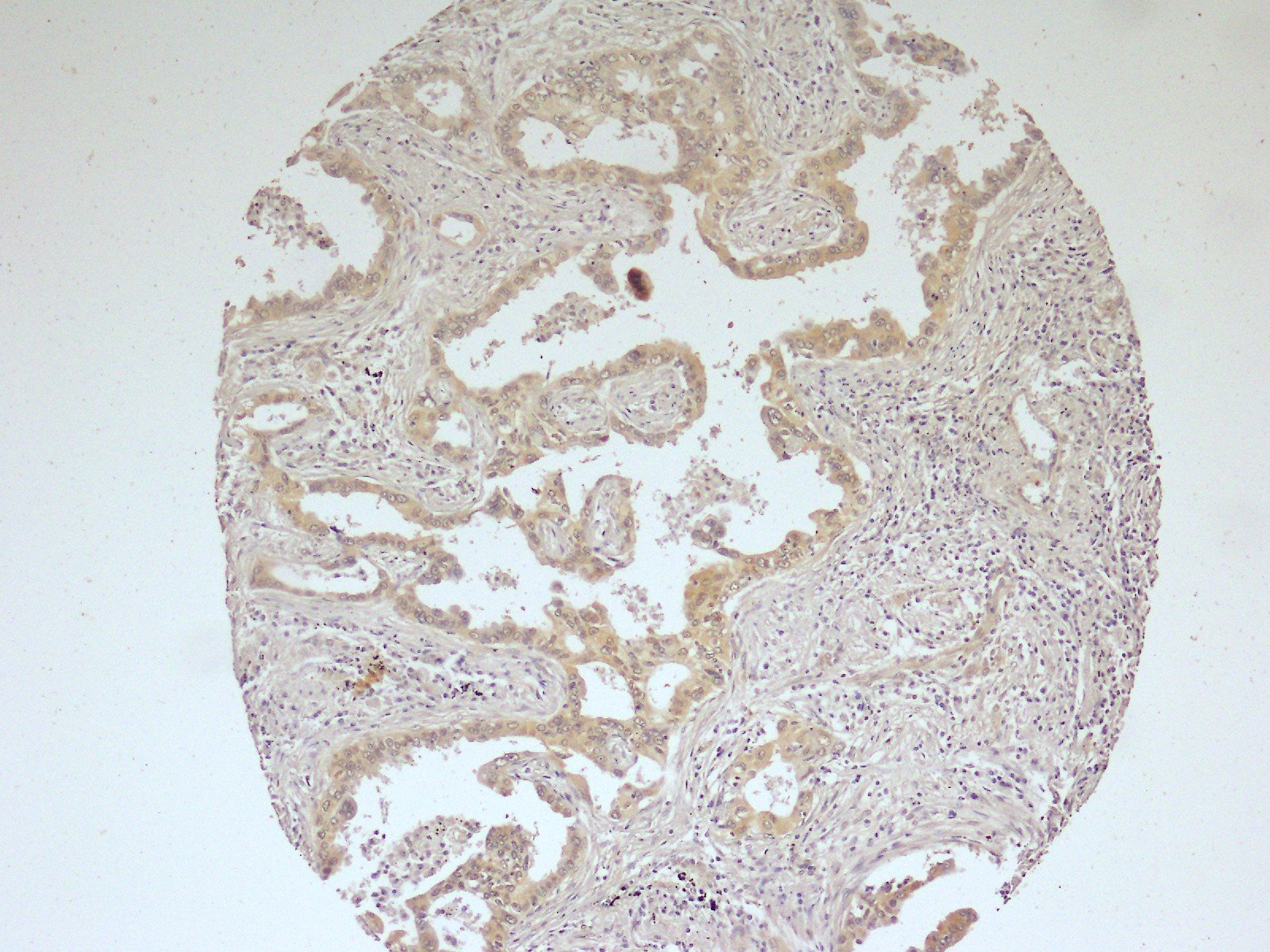

Supplement: S8 File — (ZIP) [file pone.0349359.s008.zip › FigureS2B AKT1 (+) ADC 10x.TIF]

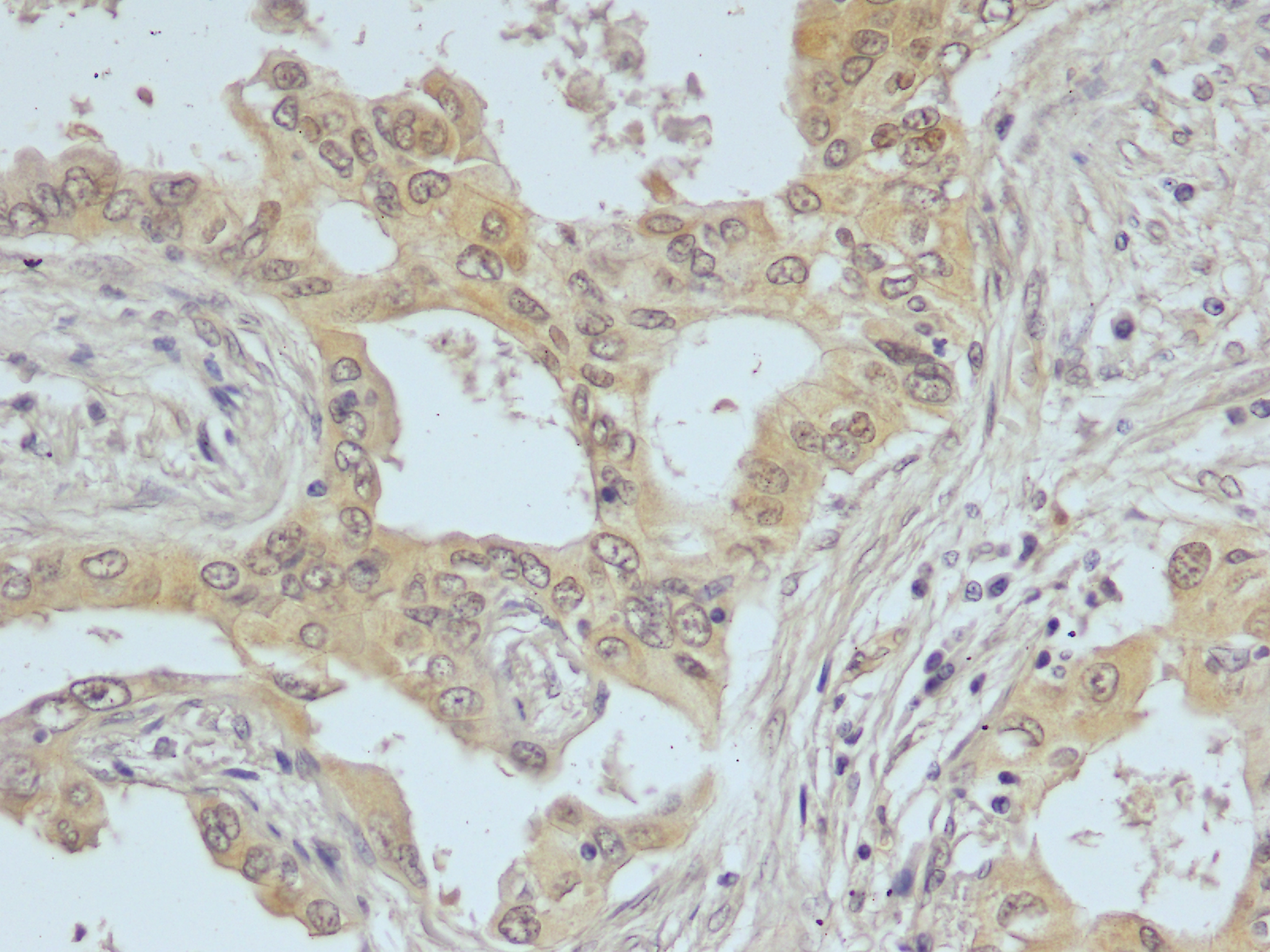

Supplement: S8 File — (ZIP) [file pone.0349359.s008.zip › FigureS2B AKT1 (+) ADC 40x.pdf]

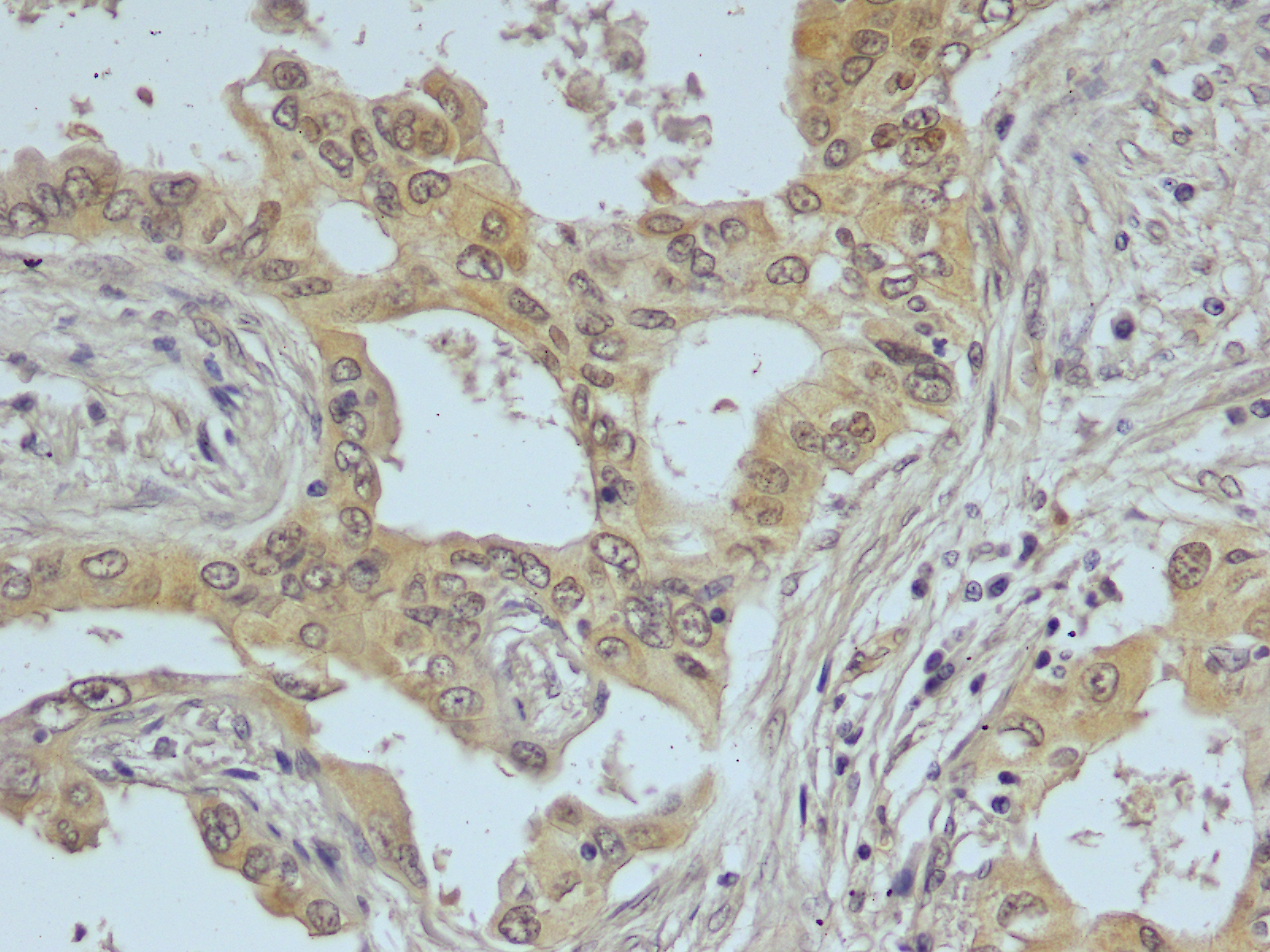

Supplement: S8 File — (ZIP) [file pone.0349359.s008.zip › FigureS2B AKT1 (+) ADC 40x.TIF]

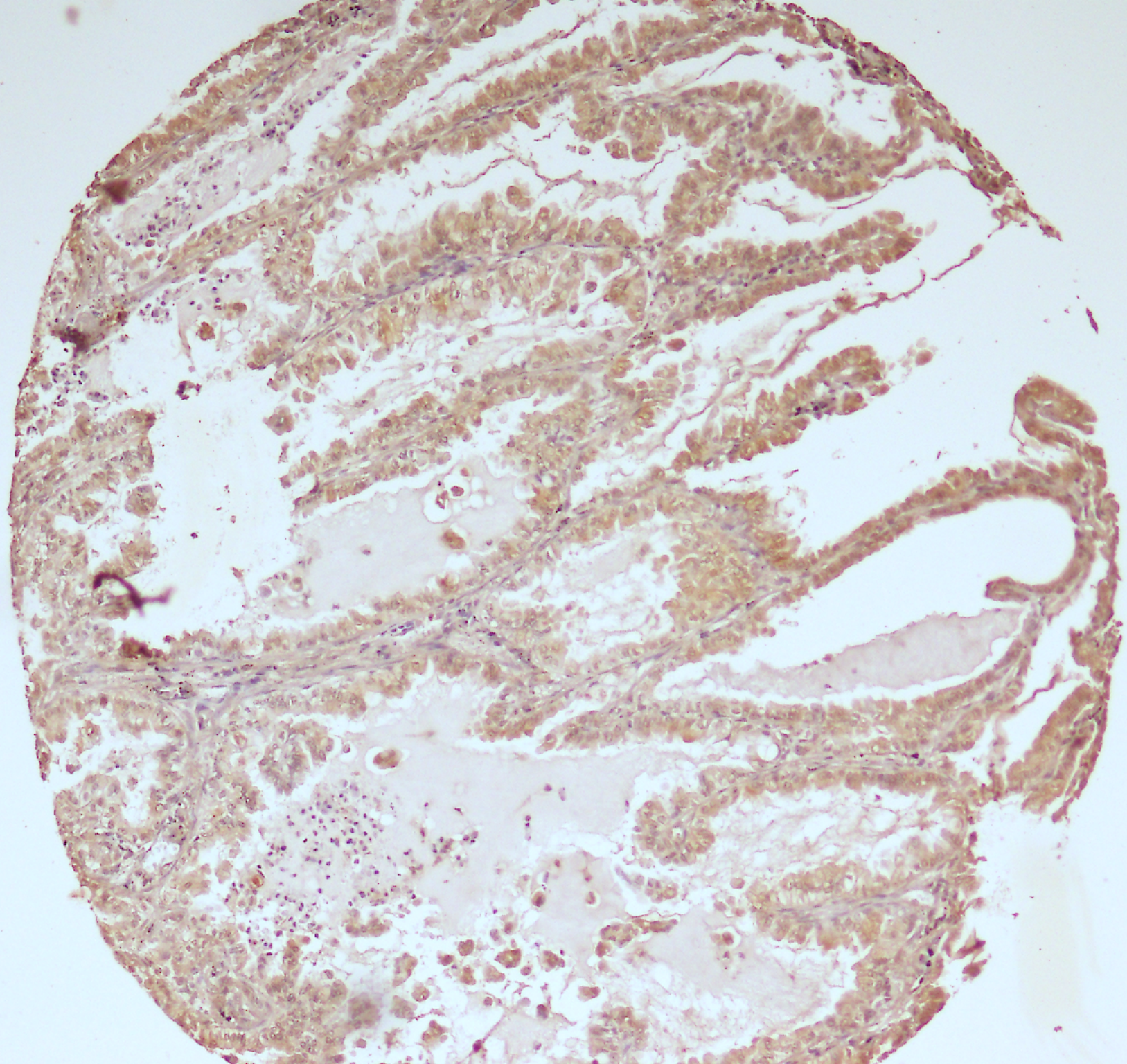

Supplement: S8 File — (ZIP) [file pone.0349359.s008.zip › FigureS2B AKT1 (++) ADC 10x.pdf]

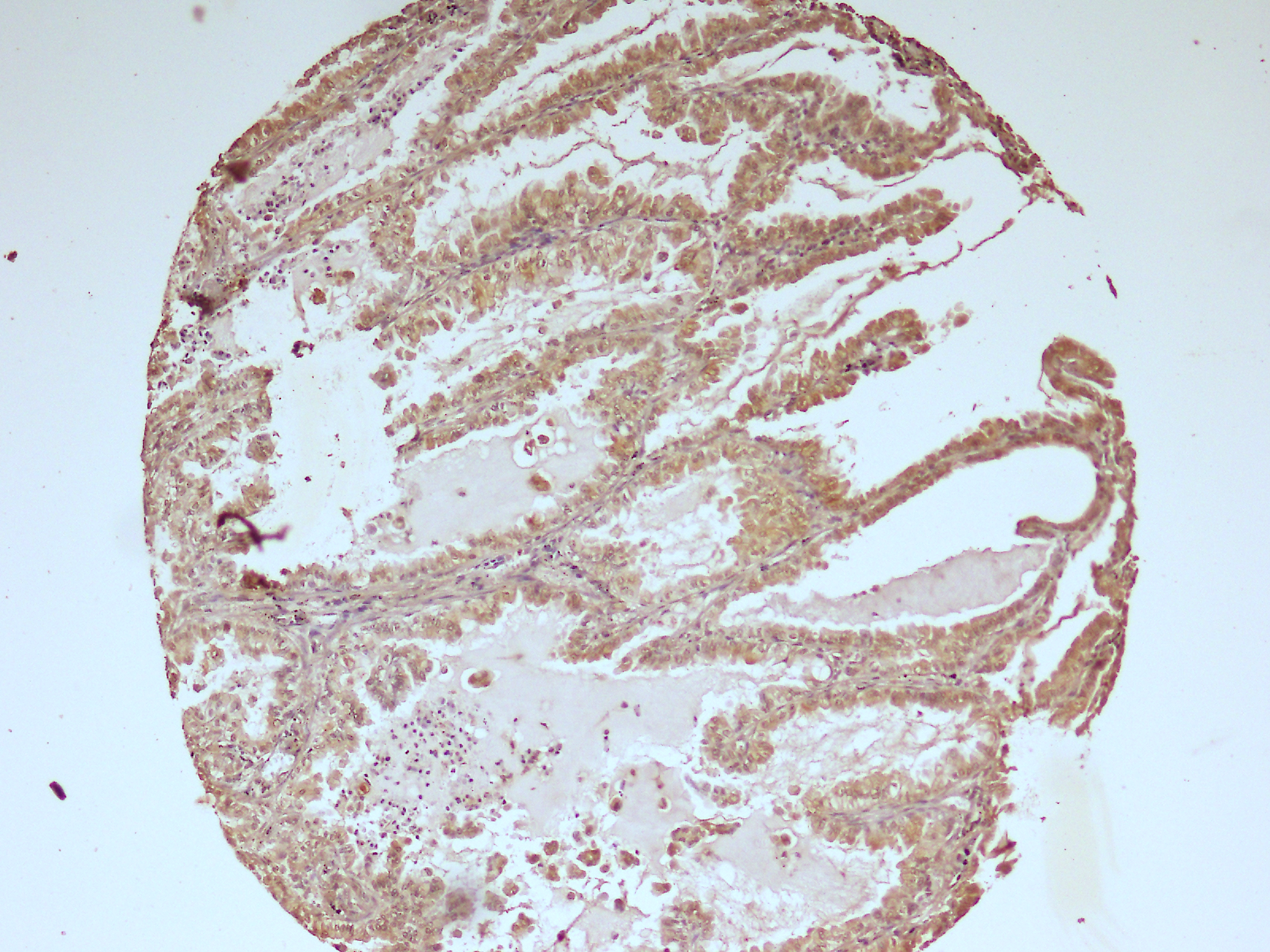

Supplement: S8 File — (ZIP) [file pone.0349359.s008.zip › FigureS2B AKT1 (++) ADC 10x.TIF]

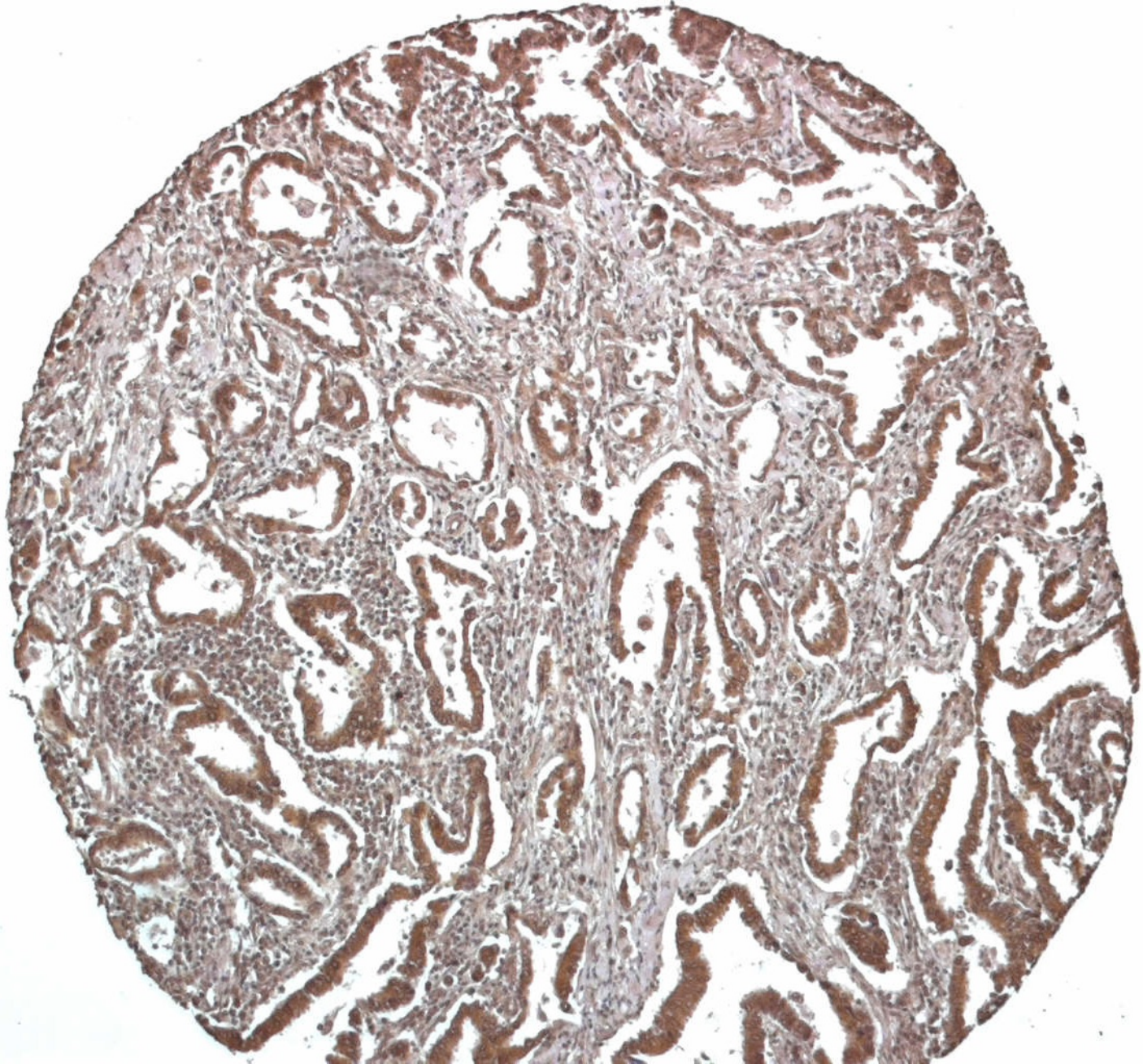

Supplement: S8 File — (ZIP) [file pone.0349359.s008.zip › FigureS2B AKT1 ADC right 10x.pdf]

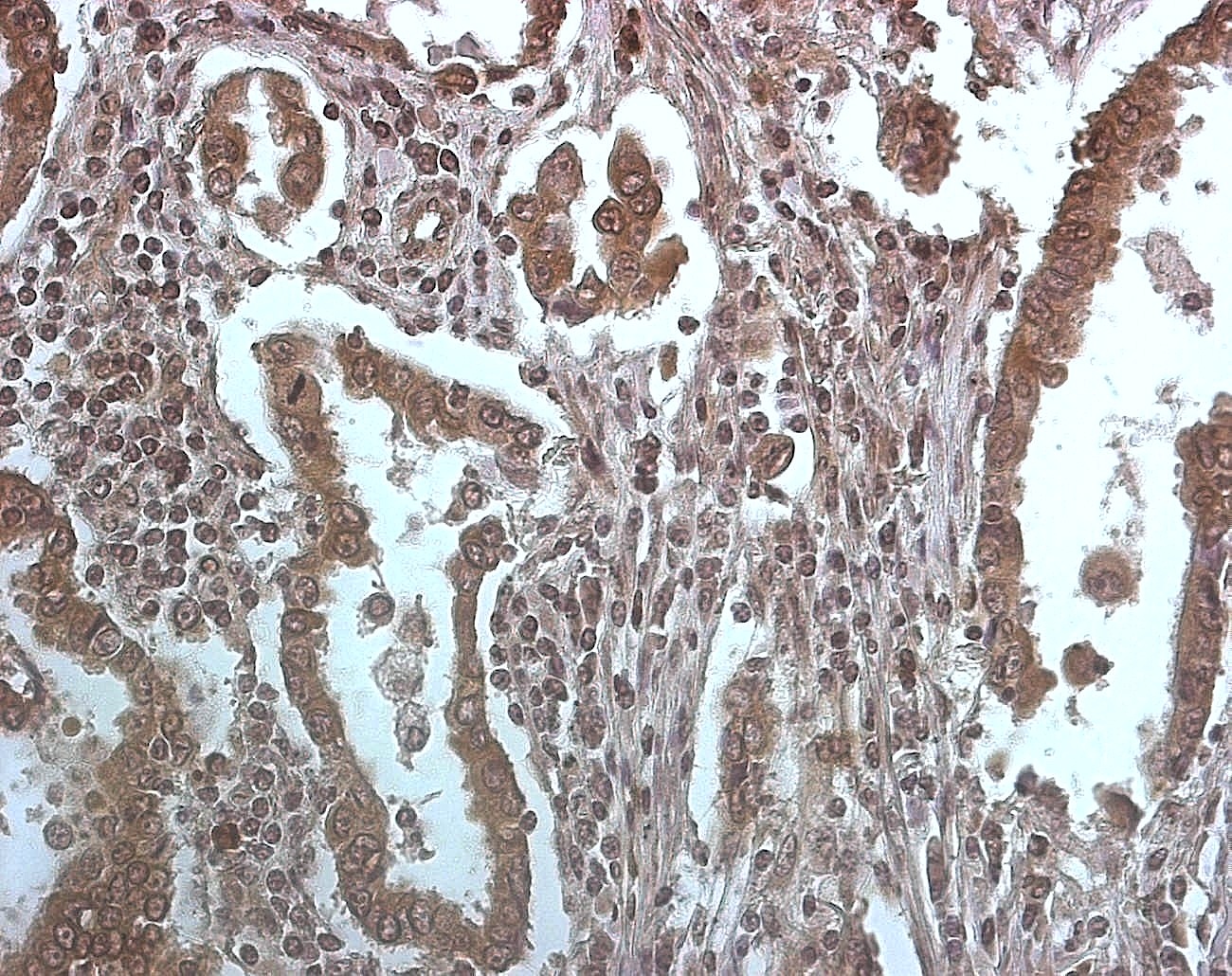

Supplement: S8 File — (ZIP) [file pone.0349359.s008.zip › FigureS2B AKT1 ADC right 40x.jpg]

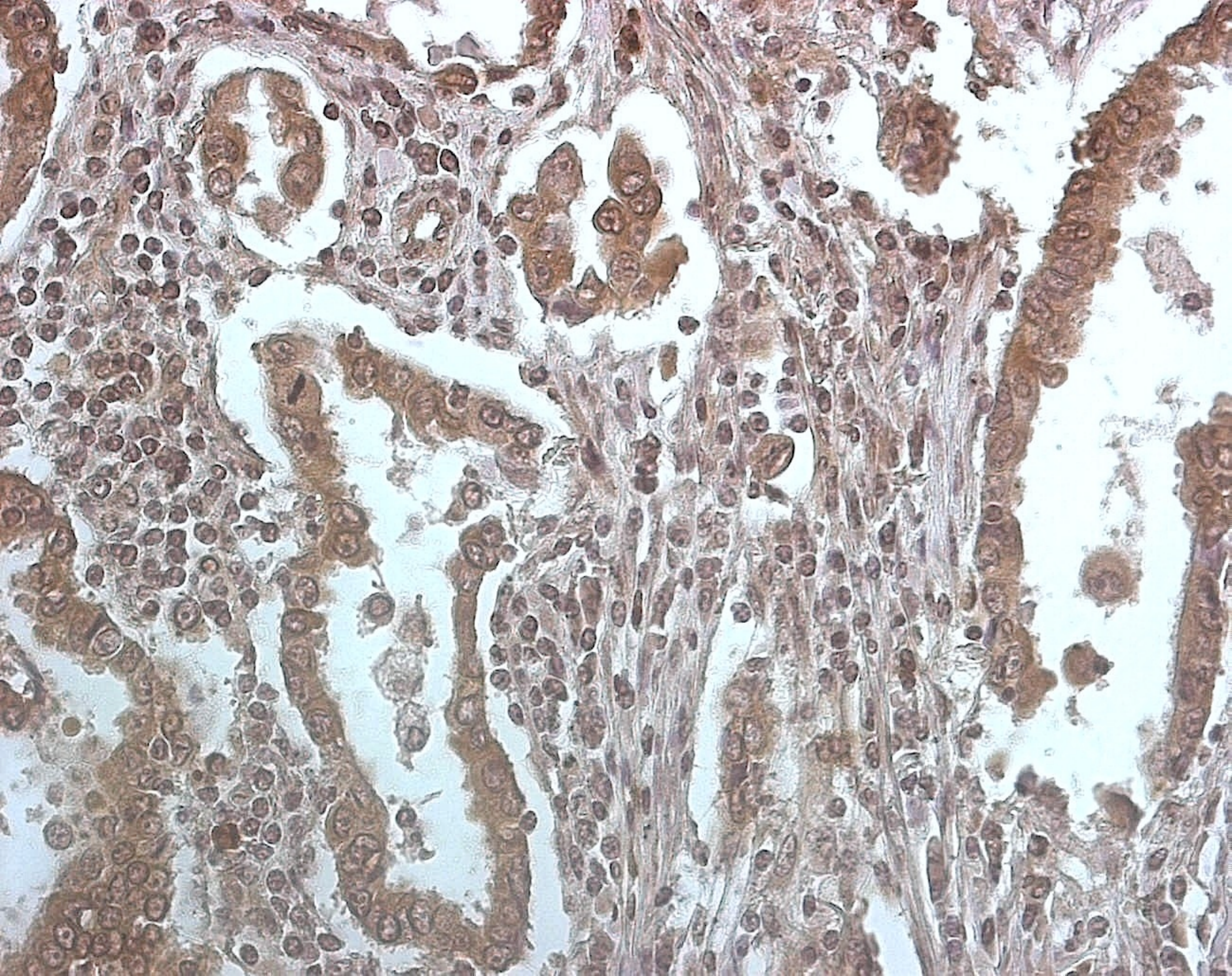

Supplement: S8 File — (ZIP) [file pone.0349359.s008.zip › FigureS2B AKT1 ADC right 40x.pdf]

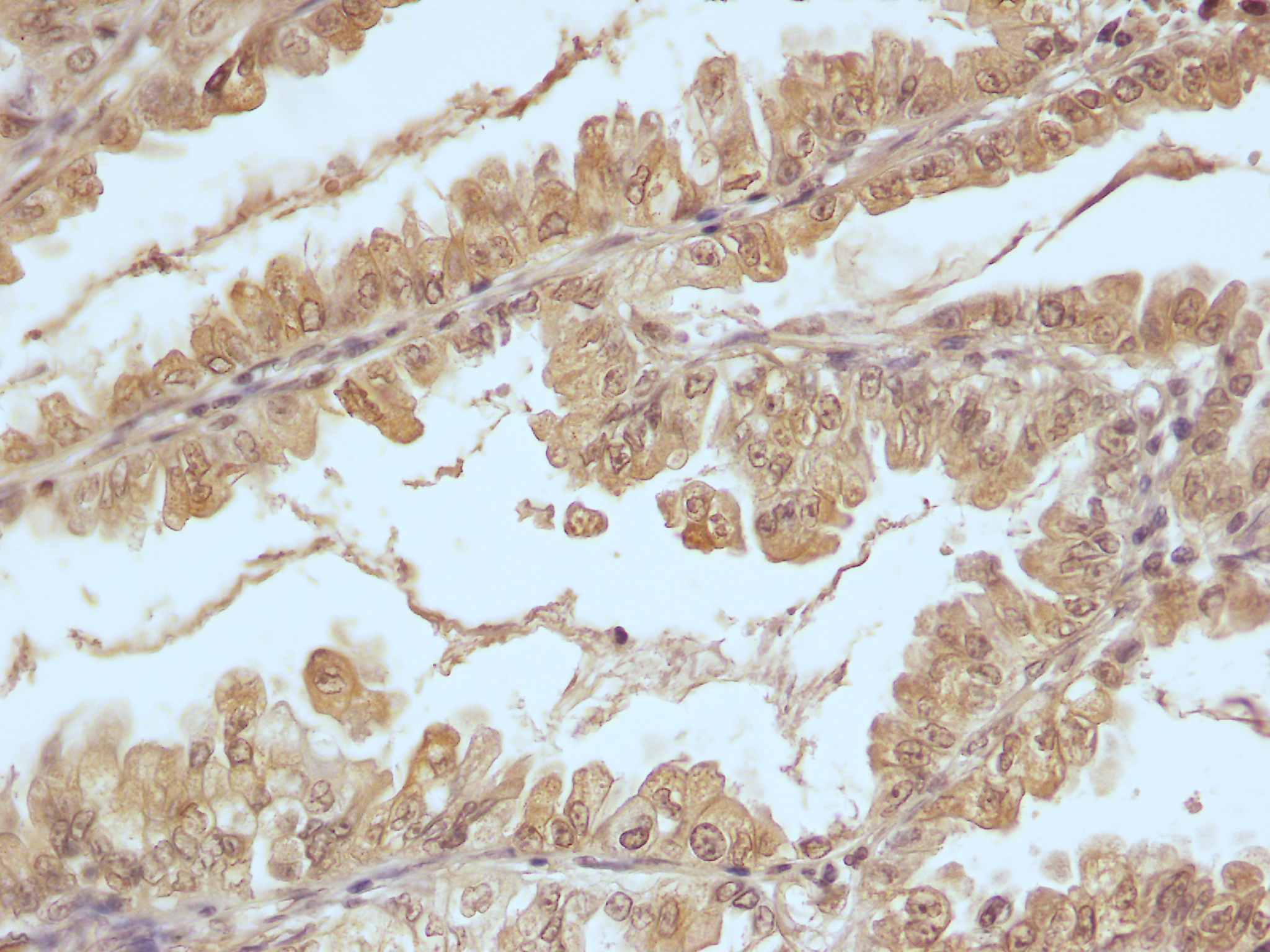

Supplement: S8 File — (ZIP) [file pone.0349359.s008.zip › FigureS2B AKT1(++) ADC 40x.pdf]

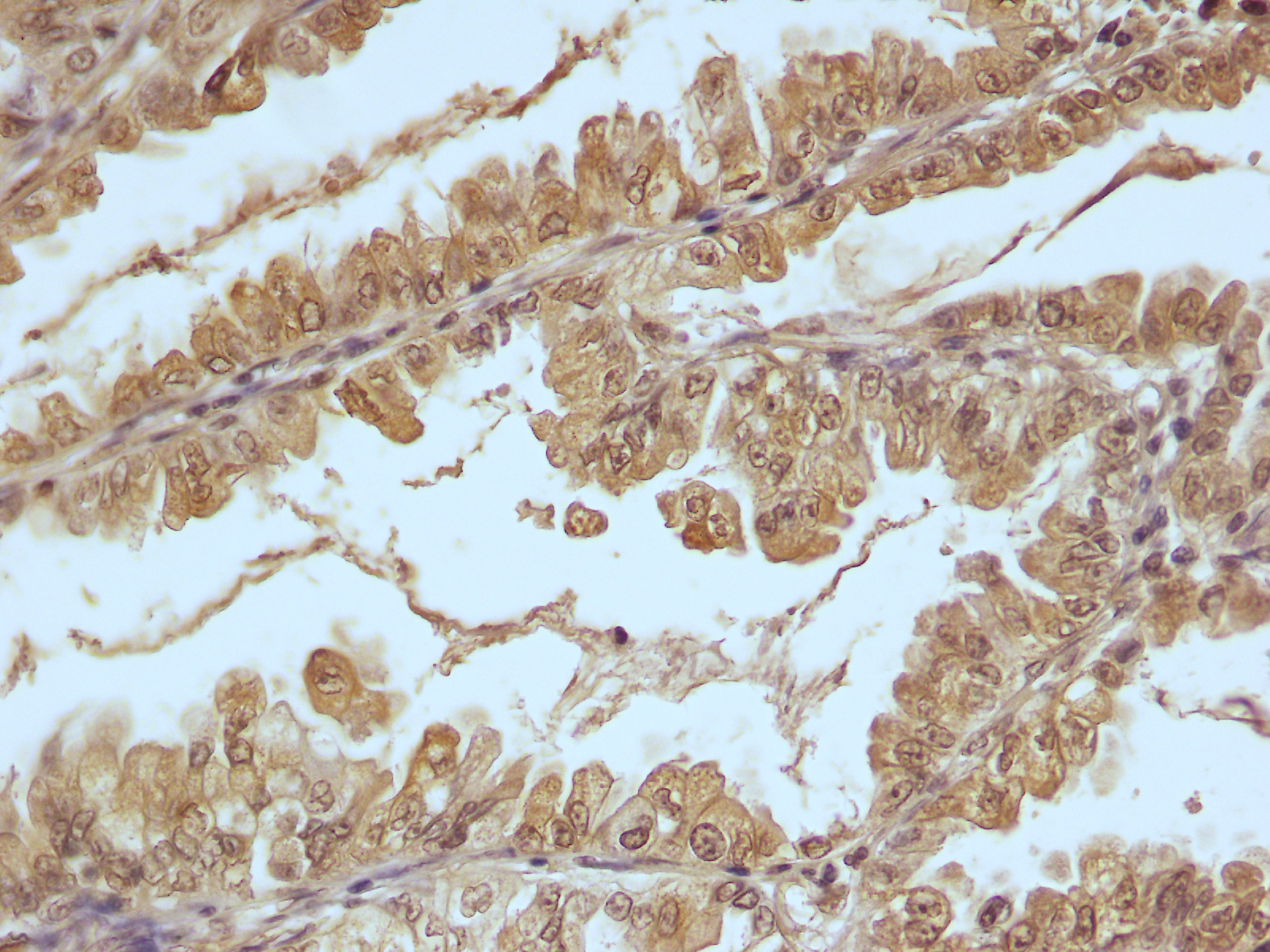

Supplement: S8 File — (ZIP) [file pone.0349359.s008.zip › FigureS2B AKT1(++) ADC 40x.TIF]

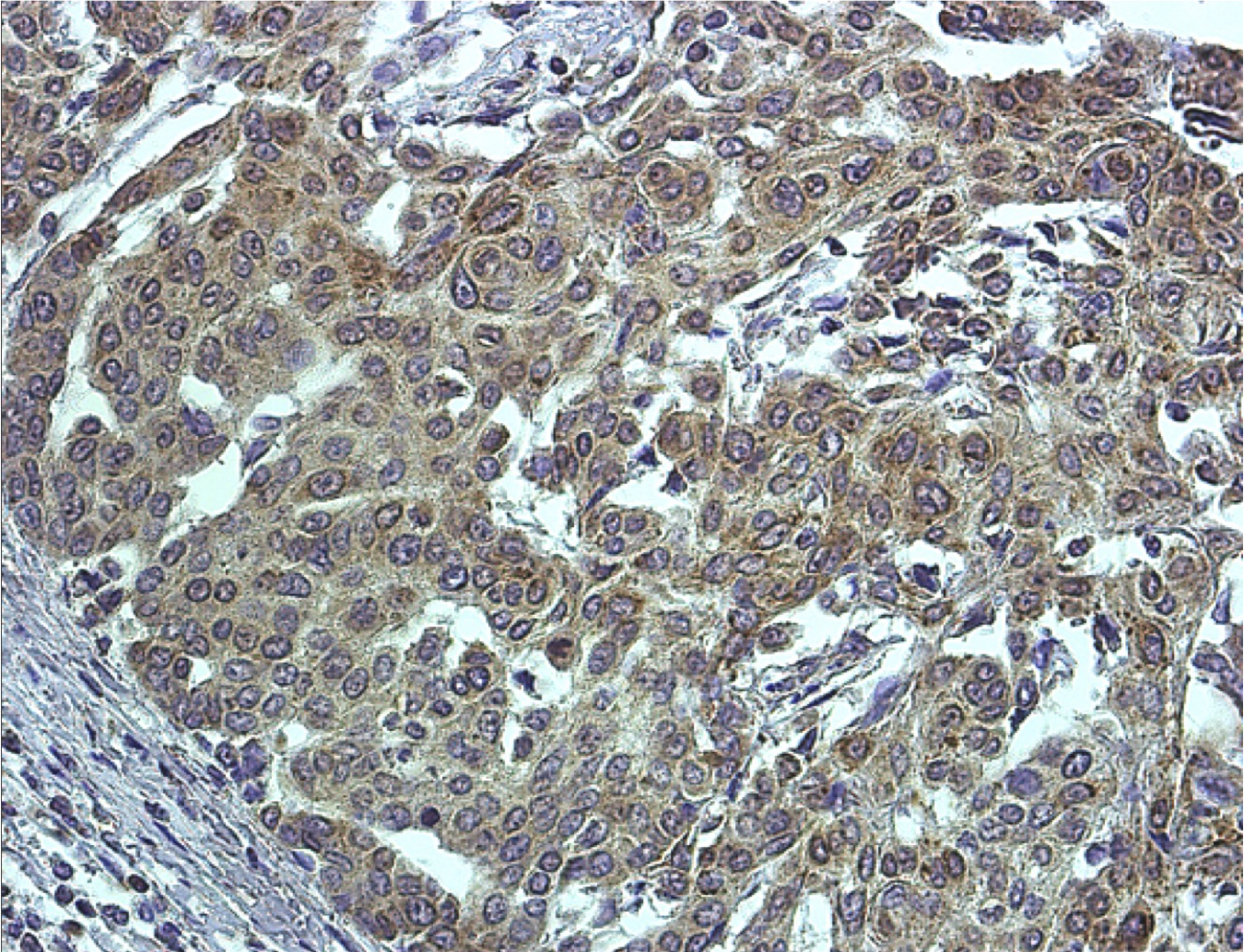

Supplement: S9 File — (ZIP) [file pone.0349359.s009.zip › Figure S3A AKT2 right 40x.tiff]

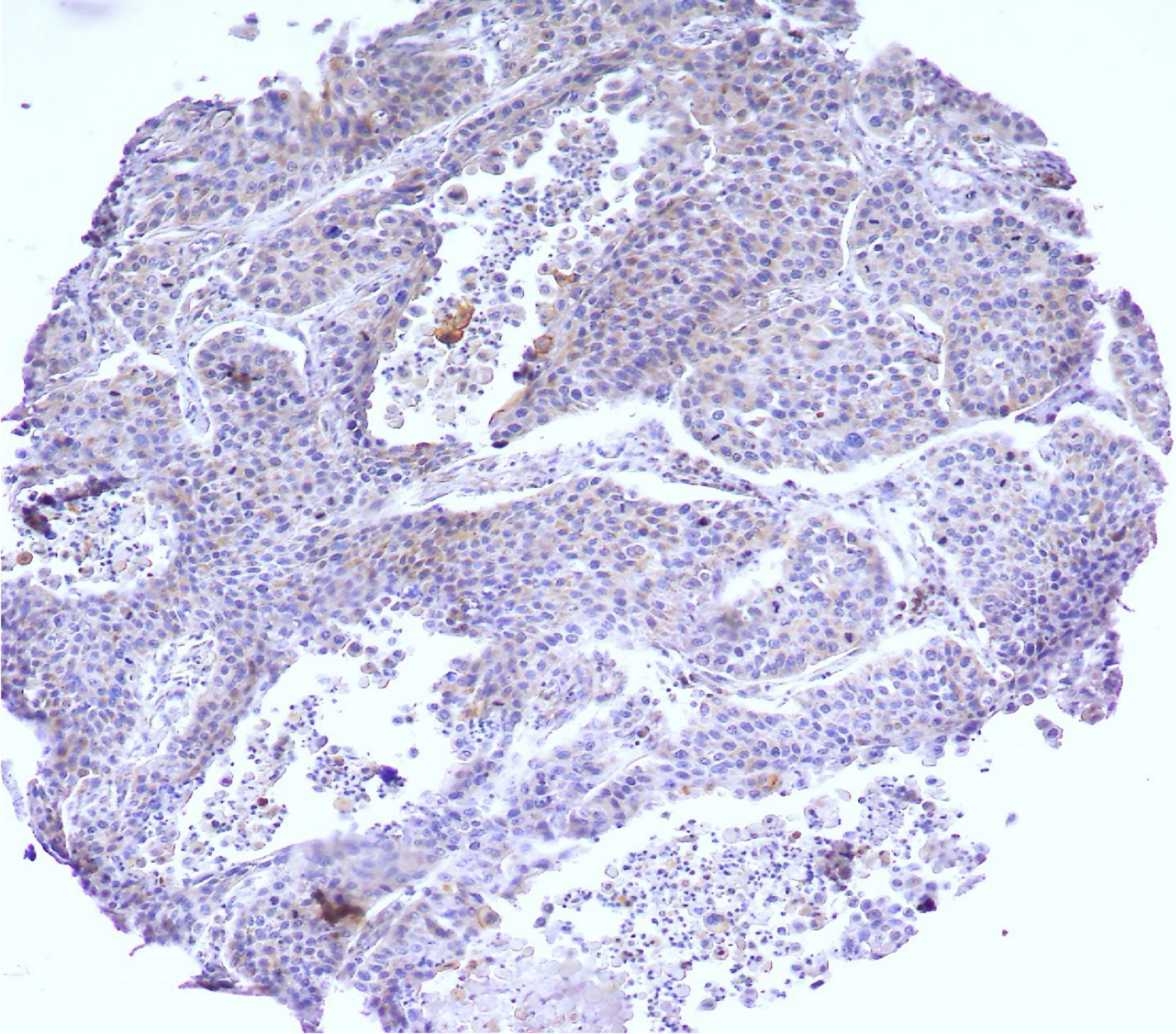

Supplement: S9 File — (ZIP) [file pone.0349359.s009.zip › Figure S3A AKT2 (+) 10x.pdf]

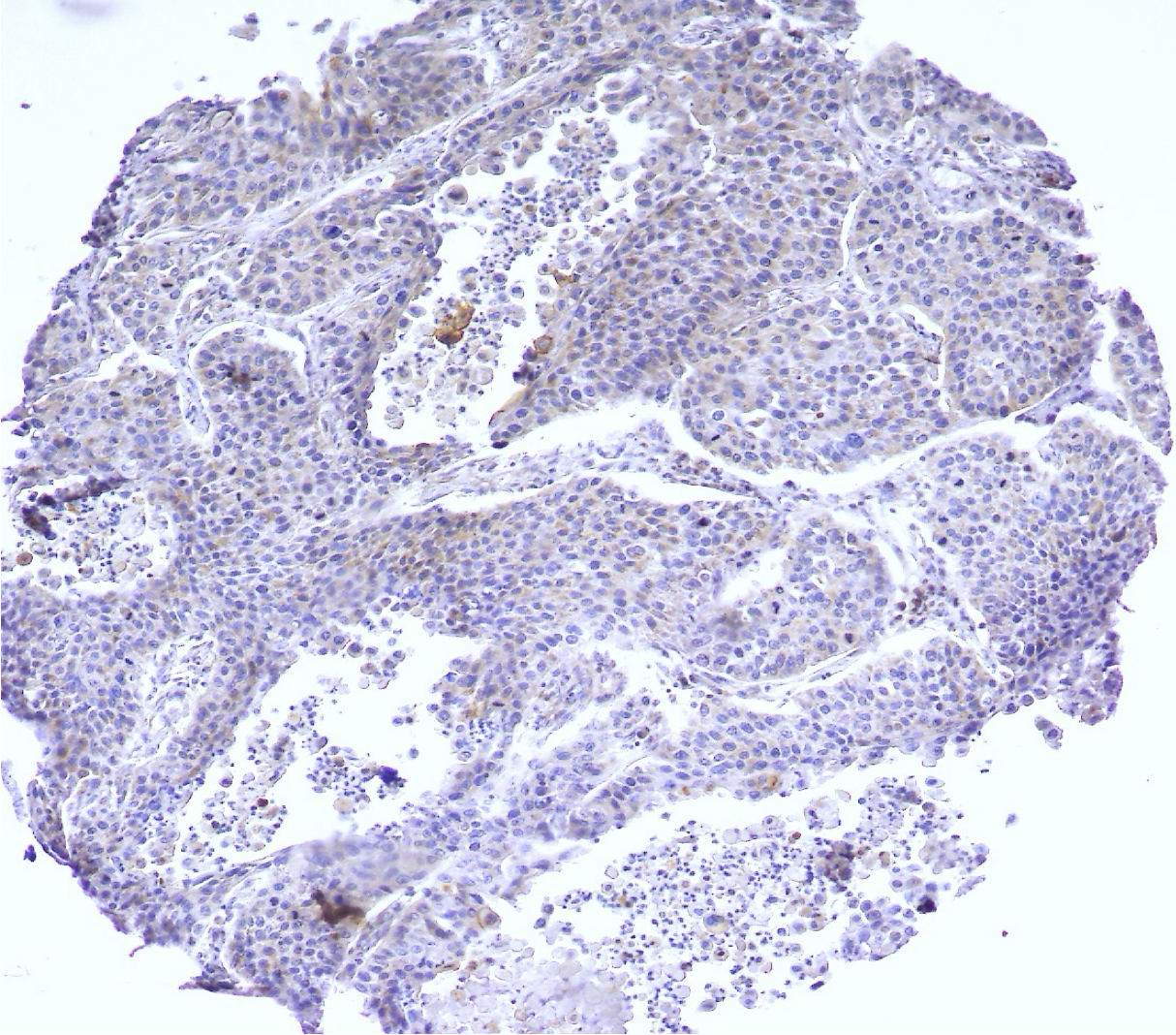

Supplement: S9 File — (ZIP) [file pone.0349359.s009.zip › Figure S3A AKT2 (+) 10x.tiff]

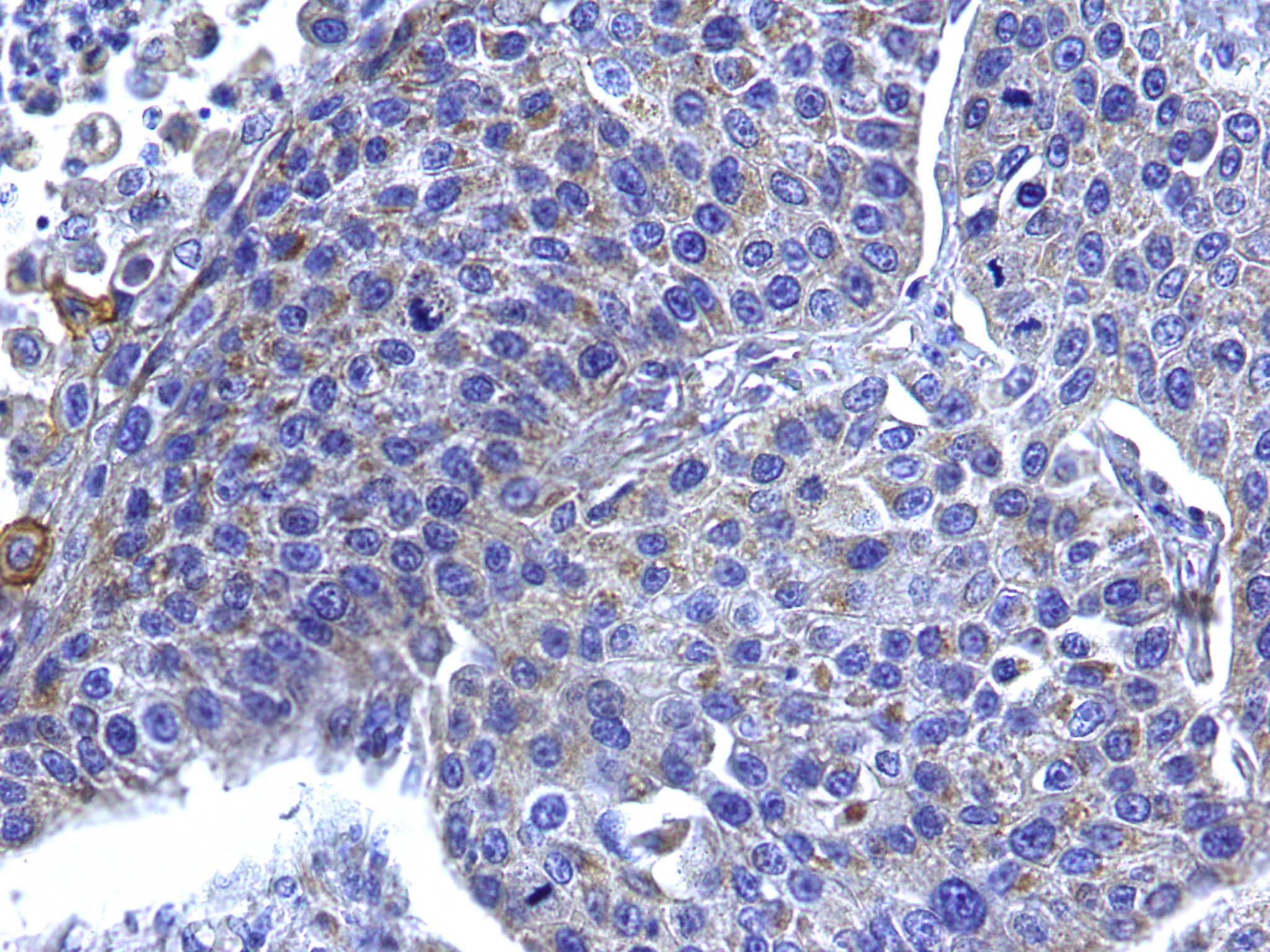

Supplement: S9 File — (ZIP) [file pone.0349359.s009.zip › Figure S3A AKT2 (+) 40x.pdf]
