# Supplementary material for: Expression of Concern: Signaling Networks Associated with AKT Activation in Non-Small Cell Lung Cancer (NSCLC): New Insights on the Role of Phosphatydil-Inositol-3 kinase
Source: PLoS One. 2026 May 14;21(5):e0349359. doi: 10.1371/journal.pone.0349359 (PMC13175380; doi:10.1371/journal.pone.0349359)
Supplement: S2 File — (ZIP) [file pone.0349359.s002.zip › Figure 2 LIST OF CONTENTS .docx]

Figure 2C AKT1 right.pdf

Figure 2A AKT1 SCC left 10x.pdf

Figure 2A AKT1 SCC right 10x.pdf

Figure 2B AKT1 ADC left 10x.pdf

Figure 2B AKT1 ADC right 10x.pdf

Figure 2C AKT1 left.pdf

Figure 2C AKT1 left.tiff

Figure 2C AKT1 right .tiff

FIGURES FOR SUBMISSION.ppt
