# Supplementary material for: Expression of Concern: Signaling Networks Associated with AKT Activation in Non-Small Cell Lung Cancer (NSCLC): New Insights on the Role of Phosphatydil-Inositol-3 kinase
Source: PLoS One. 2026 May 14;21(5):e0349359. doi: 10.1371/journal.pone.0349359 (PMC13175380; doi:10.1371/journal.pone.0349359)
Supplement: S3 File — (ZIP) [file pone.0349359.s003.zip › Figure 3 list of contents.docx]

Figure 3A AKT2 SCC left 10x.pdf

Figure 3A AKT2 SCC left 10x.tiff

Figure 3A AKT2 SCC right 10x.pdf

Figure 3A AKT2 SCC right 10x.tiff

Figure 3B AKT2 ADC left 10x.tiff

Figure 3B AKT2 ADC left 10x.pdf

Figure 3B AKT2 ADC right 10x.tiff

Figure 3C AKT2 diploid left.tiff

Figure 3C AKT2 diploid left.pdf

Figure 3C AKT2 gene amplification right.jpg

Figure 3C AKT2 gene amplification right.pdf

FIGURES FOR SUBMISSION.ppt
