# Supplementary material for: Expression of Concern: Signaling Networks Associated with AKT Activation in Non-Small Cell Lung Cancer (NSCLC): New Insights on the Role of Phosphatydil-Inositol-3 kinase
Source: PLoS One. 2026 May 14;21(5):e0349359. doi: 10.1371/journal.pone.0349359 (PMC13175380; doi:10.1371/journal.pone.0349359)
Supplement: S4 File — (ZIP) [file pone.0349359.s004.zip › Figure 4 list of contents.docx]

Figure 4A SCC PI3KCA left 10x.pdf

Figure 4A SCC PI3KCA left 10x.jpg

Figure 4A SCC PI3KCA right 10x.jpg

Figure 4A SCC PI3KCA right 10x.pdf

Figure 4B ADC PI3KCA left 10x.pdf

Figure 4B ADC PI3KCA left 10x.tif

Figure 4B ADC PI3KCA right 10x.pdf

Figure 4B ADC PI3KCA right 10x.jpg

Figure 4C PI3KCA diploid cells left.pdf

Figure 4C PI3KCA diploid cells left.tiff

Figure 4C PI3KCA gene amplification right.pdf

Figure 4C PI3KCA gene amplification right. tiff

FIGURES FOR SUBMISSION.ppt
