# Supplementary material for: Expression of Concern: Signaling Networks Associated with AKT Activation in Non-Small Cell Lung Cancer (NSCLC): New Insights on the Role of Phosphatydil-Inositol-3 kinase
Source: PLoS One. 2026 May 14;21(5):e0349359. doi: 10.1371/journal.pone.0349359 (PMC13175380; doi:10.1371/journal.pone.0349359)
Supplement: S5 File — The electropherograms were derived from an ABIPRISM 3100 sequencer. The melting curve for PI3KCA mutations are images taken from a Roche Lightcycler machine. (ZIP) [file pone.0349359.s005.zip › Figure 5 list of contents.docx]

Figure 5A PI3KCA exon 9.pdf

Figure 5A PI3KCA exon 20.pdf

Figure 5B_SCC6-PI3K_ E545K Forward.abi

Figure 5C_ADC4_G12A_KRAS Reverse.ab1

Figure 5C_ADC7-WT_KRAS_Reverse.ab1

Figure 5C_ADC25_G12C_KRAS_Reverse.ab1

Figure 5D pAKT ADC-30 40x.pdf

Figure 5D pAKT ADC-30 40x ad.jpg

Figure5C_ADC14_G13C_KRAS_Reverse.ab1

Figure5C_ADC30_G12V_KRAS_Reverse.ab1
