# Supplementary material for: Expression of Concern: Signaling Networks Associated with AKT Activation in Non-Small Cell Lung Cancer (NSCLC): New Insights on the Role of Phosphatydil-Inositol-3 kinase
Source: PLoS One. 2026 May 14;21(5):e0349359. doi: 10.1371/journal.pone.0349359 (PMC13175380; doi:10.1371/journal.pone.0349359)
Supplement: S6 File — (ZIP) [file pone.0349359.s006.zip › Figure 6 list of contents.docx]

Figure 6A AKT blot.pdf

Figure 6A p-AKT blot.pdf

Figure 6C complete file _see sheet shPI3K.xls

Figure 6C.xlsx

Figure 6D complete file_see sheet AKT2.xls

Figure 6D.xlsx

FIGURES FOR SUBMISSION.ppt
