# Supplementary material for: Expression of Concern: Signaling Networks Associated with AKT Activation in Non-Small Cell Lung Cancer (NSCLC): New Insights on the Role of Phosphatydil-Inositol-3 kinase
Source: PLoS One. 2026 May 14;21(5):e0349359. doi: 10.1371/journal.pone.0349359 (PMC13175380; doi:10.1371/journal.pone.0349359)
Supplement: S7 File — (ZIP) [file pone.0349359.s007.zip › Figure S1 list of contents.docx]

Figure S1A_left_Actin blot.pdf

Figure S1A_left_Akt1 blot .pdf

Figure S1A_right_Actin blot.pdf

Figure S1A_right_AKT2 blot.pdf

Figure S1B AKT1 normal 10x.pdf

Figure S1B AKT1 normal 10x.tiff

Figure S1B AKT2 normal 10x.pdf

Figure S1B AKT2 normal 10x.tiff

Figure S1B pAKT normal 10x.pdf

Figure S1B pAKT normal 10x.tiff

Figure S1B PI3KCA normal 10x.pdf

Figure S1B PI3KCA normal 10x.tiff

SUPPORTING FIGURES FOR SUBMISSION.ppt
