# Supplementary material for: Expression of Concern: Signaling Networks Associated with AKT Activation in Non-Small Cell Lung Cancer (NSCLC): New Insights on the Role of Phosphatydil-Inositol-3 kinase
Source: PLoS One. 2026 May 14;21(5):e0349359. doi: 10.1371/journal.pone.0349359 (PMC13175380; doi:10.1371/journal.pone.0349359)
Supplement: S8 File — (ZIP) [file pone.0349359.s008.zip › Figure S2 list of contents.docx]

Figure S2A AKT1 SCC right 10x

Figure S2A AKT1 SCC right 10x.jpg

Figure S2A AKT1 SCC left 40x

Figure S2A AKT1 SCC left 40x.jpg

Figure S2A AKT1 SCC left 10x

Figure S2A AKT1 SCC left 10x.jpg

Figure S2A AKT1 SCC right 40x

Figure S2A AKT1 SCC right 40x.jpg

Figure S2B AKT1 ADC left 10x

Figure S2B AKT1 ADC left 10x.jpg

Figure S2B AKT1 ADC left40x

Figure S2B AKT1 ADC left40x.jpg

FigureS2A AKT1 (++) SCC 40x

FigureS2A AKT1 (++) SCC 40x.TIF

FigureS2A AKT1(+) SCC 40x

FigureS2A AKT1(+) SCC 40x.TIF

FigureS2A AKT1(+)SCC 10x

FigureS2A AKT1(+)SCC 10x.TIF

FigureS2A AKT1(++) SCC 10x

FigureS2A AKT1(++) SCC 10x.TIF

FigureS2B AKT1 (+) ADC 10x

FigureS2B AKT1 (+) ADC 10x.TIF

FigureS2B AKT1 (+) ADC 40x

FigureS2B AKT1 (+) ADC 40x.TIF

FigureS2B AKT1 (++) ADC 10x

FigureS2B AKT1 (++) ADC 10x.TIF

FigureS2B AKT1 ADC right 10x

FigureS2B AKT1 ADC right 10x.jpg

FigureS2B AKT1 ADC right 40x

FigureS2B AKT1 ADC right 40x.jpg

FigureS2B AKT1(++) ADC 40x

FigureS2B AKT1(++) ADC 40x.TIF

SUPPORTING FIGURES FOR SUBMISSION.ppt
