# Supplementary material for: Expression of Concern: Signaling Networks Associated with AKT Activation in Non-Small Cell Lung Cancer (NSCLC): New Insights on the Role of Phosphatydil-Inositol-3 kinase
Source: PLoS One. 2026 May 14;21(5):e0349359. doi: 10.1371/journal.pone.0349359 (PMC13175380; doi:10.1371/journal.pone.0349359)
Supplement: S9 File — (ZIP) [file pone.0349359.s009.zip › Figure S3 list of contents.docx]

Figure S3A AKT2 (+) 10x.pdf

Figure S3A AKT2 (+) 40x.pdf

Figure S3A AKT2 (+) 40x.TIF

Figure S3A AKT2 (++) 10x.pdf

Figure S3A AKT2 (++) 10x.TIF

Figure S3A AKT2 (++) 40x.pdf

Figure S3A AKT2 (++) 40x.TIF

Figure S3A AKT2 left 10x.pdf

Figure S3A AKT2 left 10x.tiff

Figure S3A AKT2 left 40x.pdf

Figure S3A AKT2 left 40x.tiff

Figure S3A AKT2 right 10x.pdf

Figure S3A AKT2 right 10x.tiff

Figure S3A AKT2 right 40x.pdf

Figure S3A AKT2 right 40x.tiff

Figure S3A AKT2(+) 10x.tiff

Figure S3B AKT2 (+) 10x.tiff

Figure S3B AKT2 (+) 10x.TIF

Figure S3B AKT2 (+) 40x

Figure S3B AKT2 (+) 40x.TIF

Figure S3B AKT2 (++) 10x.pdf

Figure S3B AKT2 (++) 10x.tiff

Figure S3B AKT2 (++) 10x.TIF

Figure S3B AKT2 (++) 40x.pdf

Figure S3B AKT2 (++) 40x.TIF

Figure S3B AKT2 left 10x.pdf

Figure S3B AKT2 left 10x.tiff

Figure S3B AKT2 left 40x.pdf

Figure S3B AKT2 left 40x.tiff

Figure S3B AKT2 right 10x.pdf

Figure S3B AKT2 right 10x.tiff

Figure S3B AKT2 right 40x.pdf

Figure S3B AKT2 right 40x.tiff

SUPPORTING FIGURES FOR SUBMISSION.ppt
