# Supplementary figures and images for: Expression of Concern: Signaling Networks Associated with AKT Activation in Non-Small Cell Lung Cancer (NSCLC): New Insights on the Role of Phosphatydil-Inositol-3 kinase (part 2 of 2)
Source: PLoS One. 2026 May 14;21(5):e0349359. doi: 10.1371/journal.pone.0349359 (PMC13175380; doi:10.1371/journal.pone.0349359)

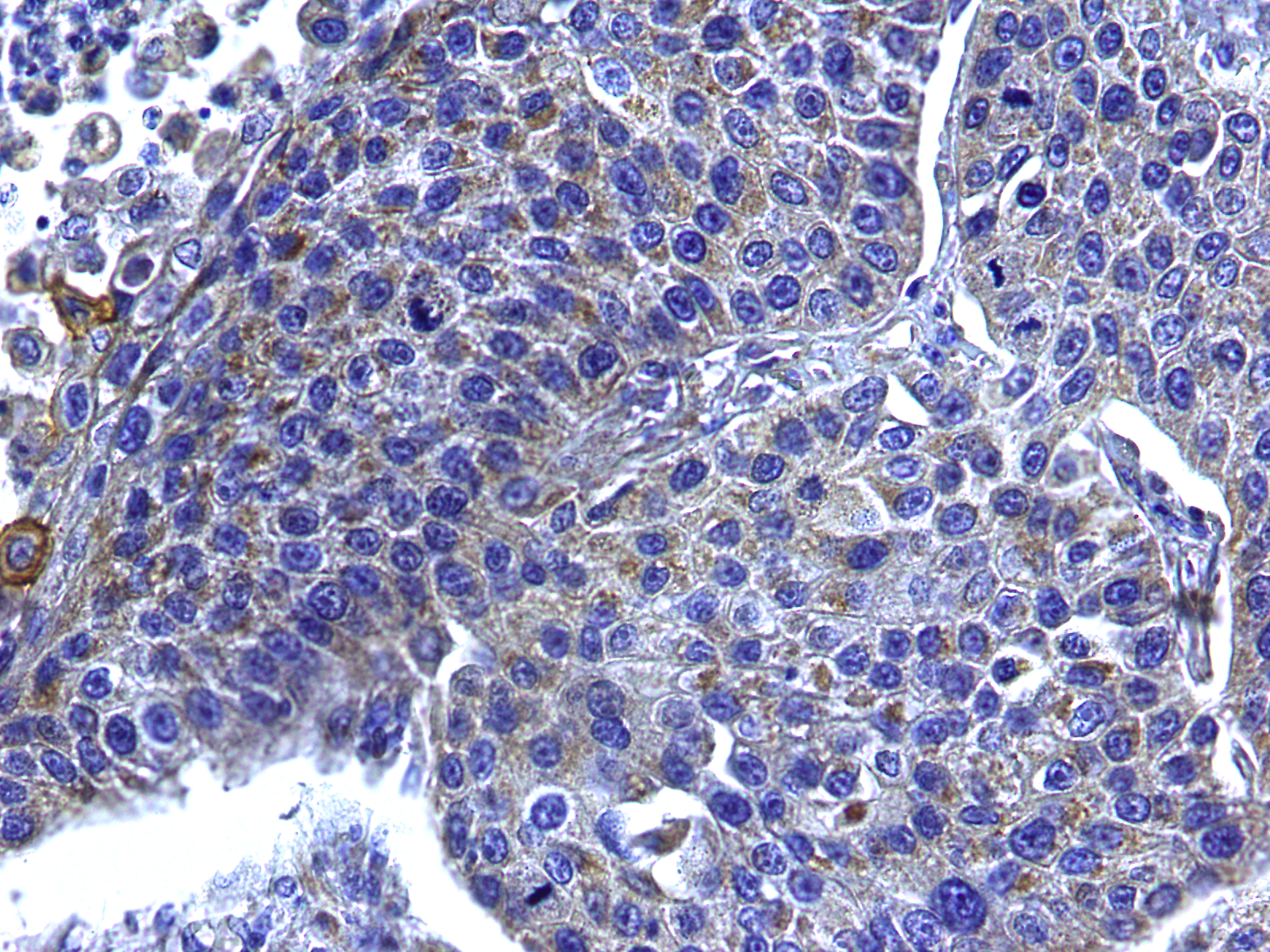

Supplement: S9 File — (ZIP) [file pone.0349359.s009.zip › Figure S3A AKT2 (+) 40x.TIF]

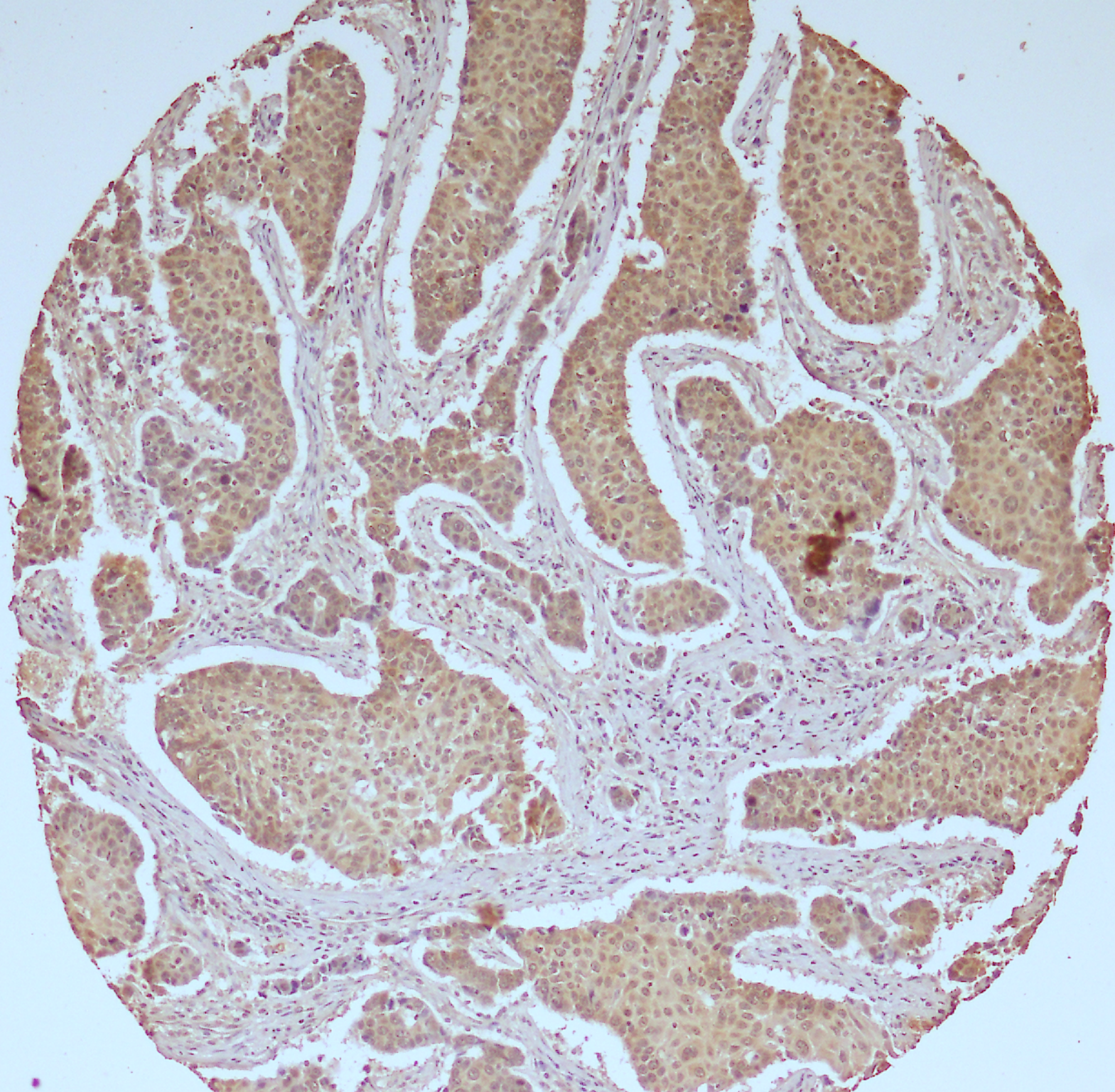

Supplement: S9 File — (ZIP) [file pone.0349359.s009.zip › Figure S3A AKT2 (++) 10x.pdf]

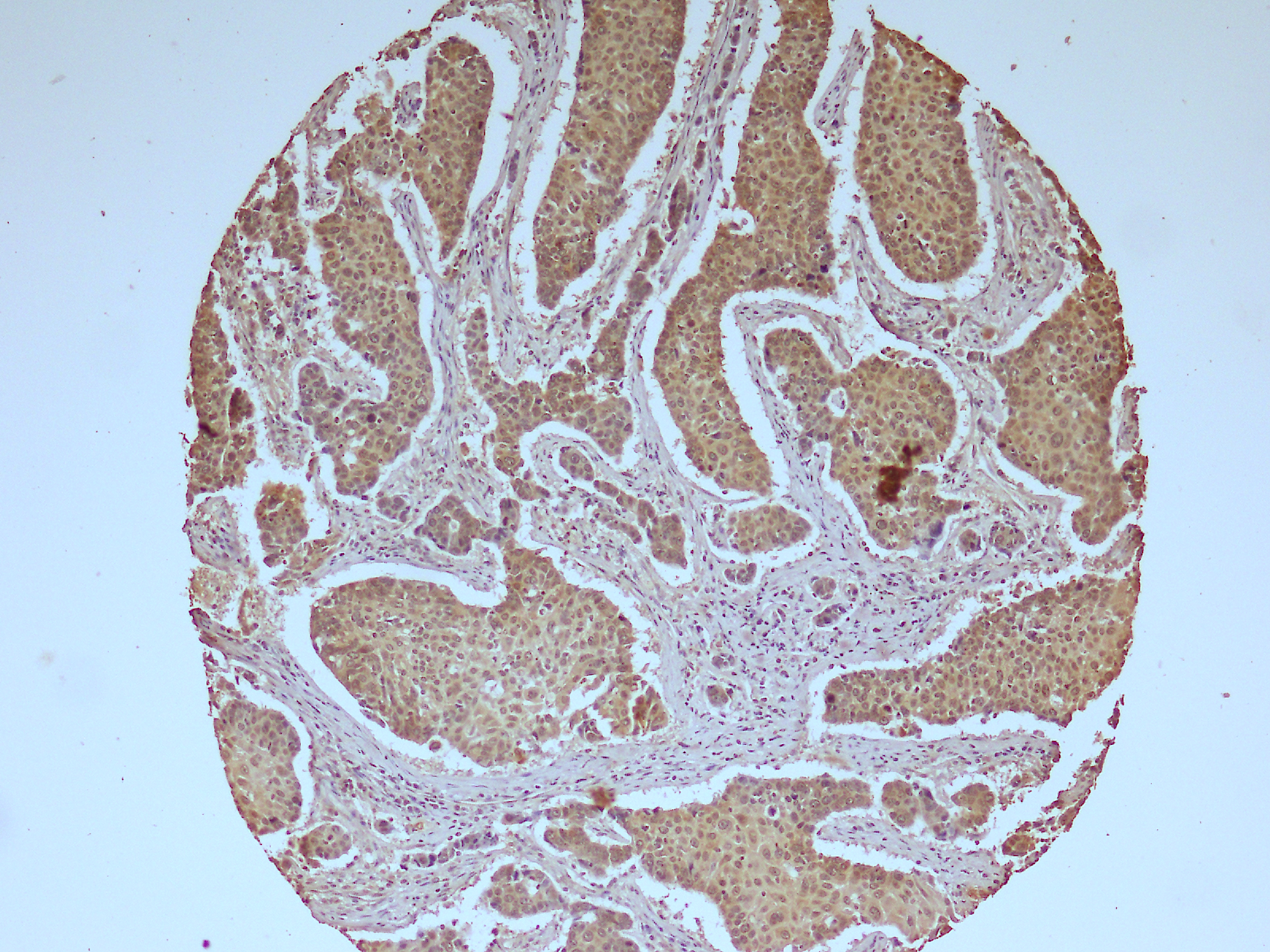

Supplement: S9 File — (ZIP) [file pone.0349359.s009.zip › Figure S3A AKT2 (++) 10x.TIF]

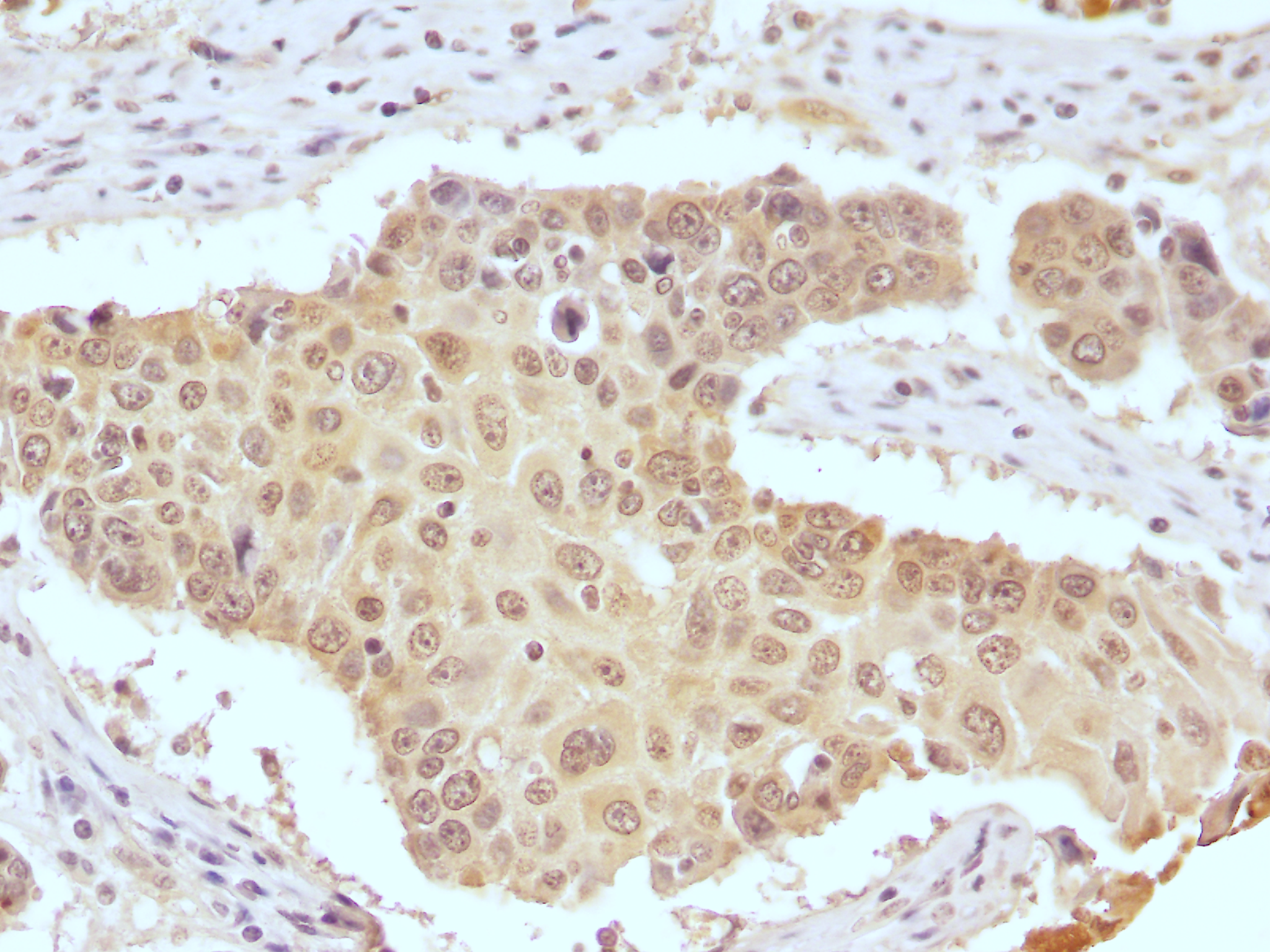

Supplement: S9 File — (ZIP) [file pone.0349359.s009.zip › Figure S3A AKT2 (++) 40x.pdf]

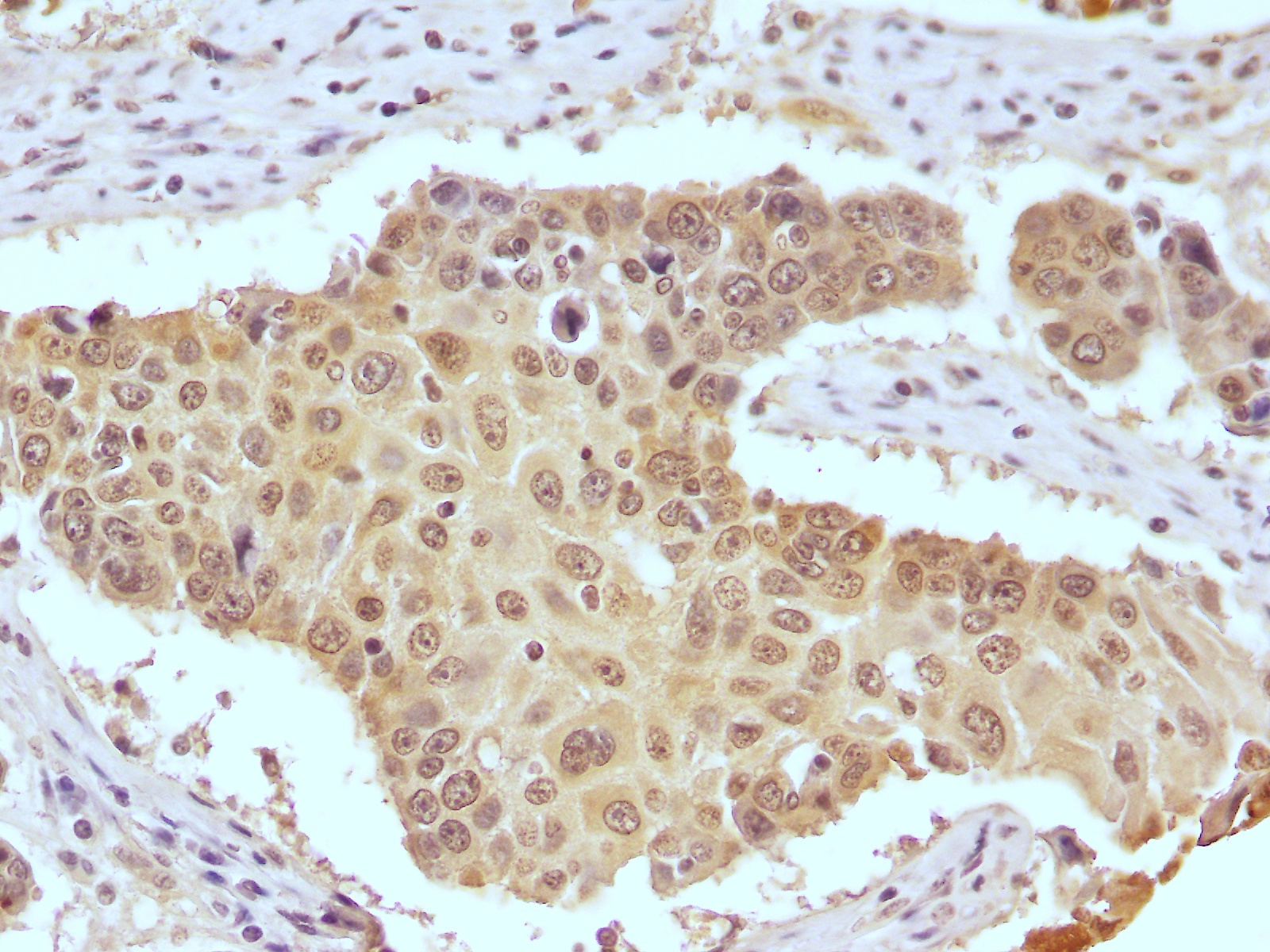

Supplement: S9 File — (ZIP) [file pone.0349359.s009.zip › Figure S3A AKT2 (++) 40x.TIF]

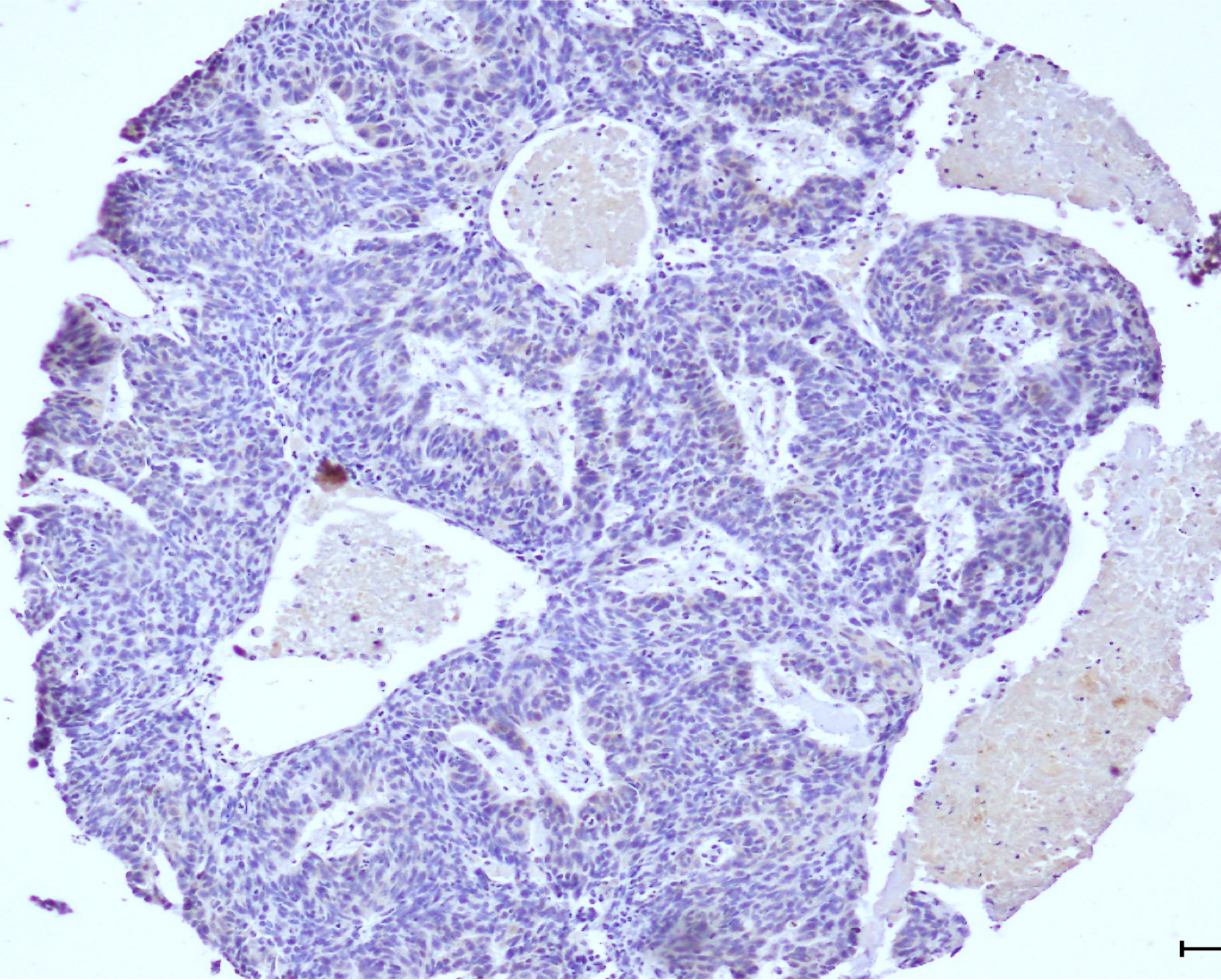

Supplement: S9 File — (ZIP) [file pone.0349359.s009.zip › Figure S3A AKT2 left 10x.pdf]

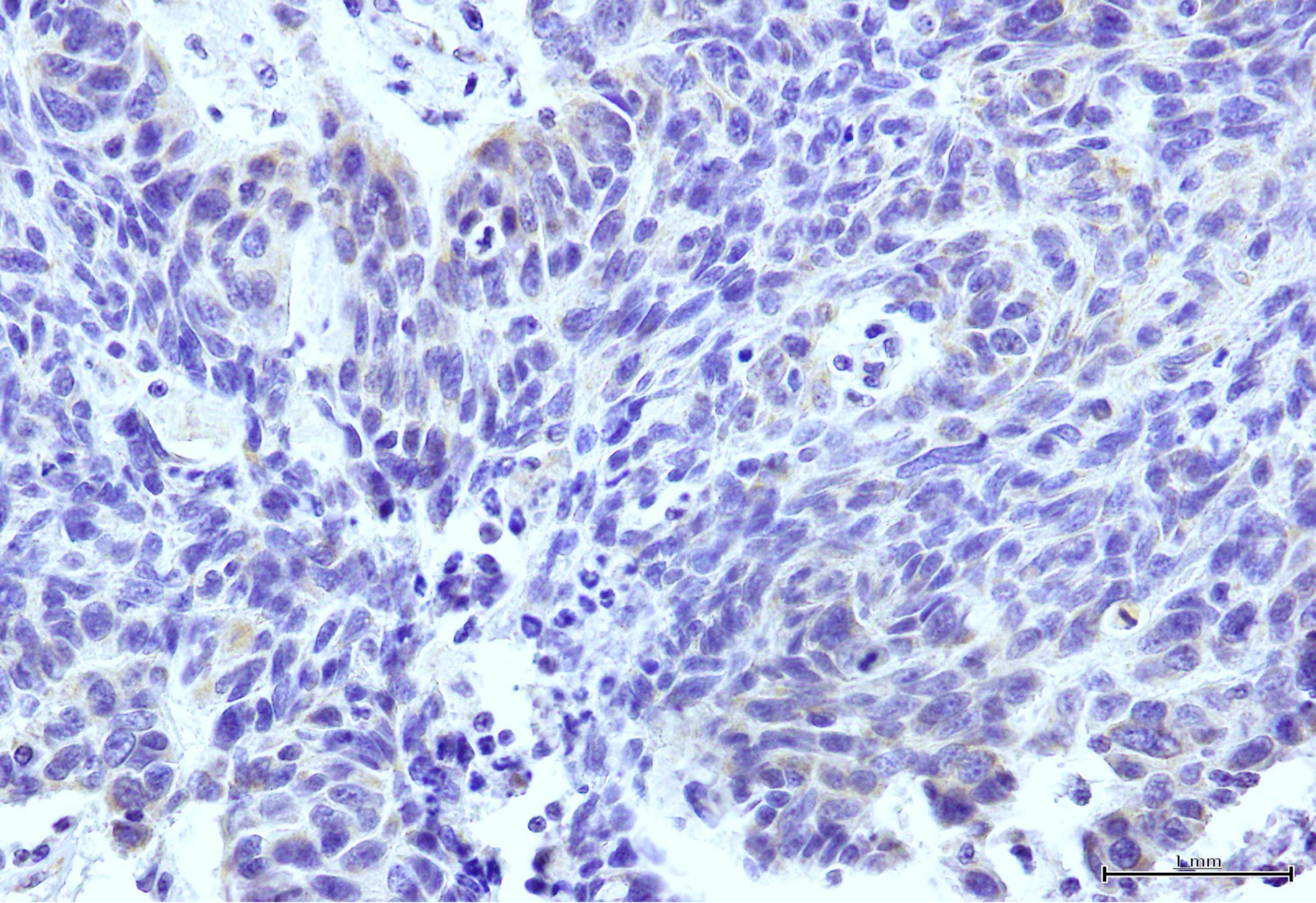

Supplement: S9 File — (ZIP) [file pone.0349359.s009.zip › Figure S3A AKT2 left 40x.pdf]

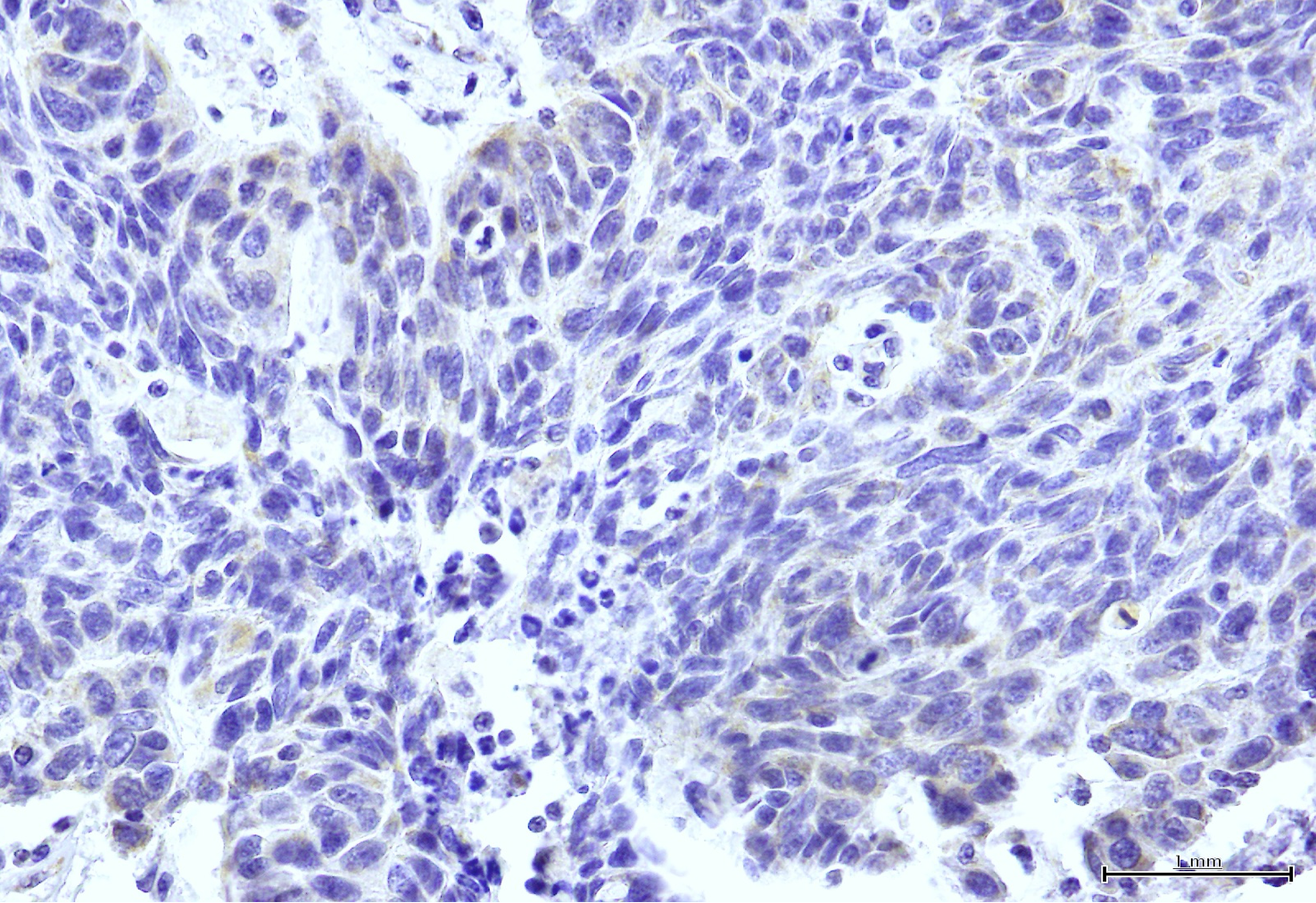

Supplement: S9 File — (ZIP) [file pone.0349359.s009.zip › Figure S3A AKT2 left 40x.tiff]

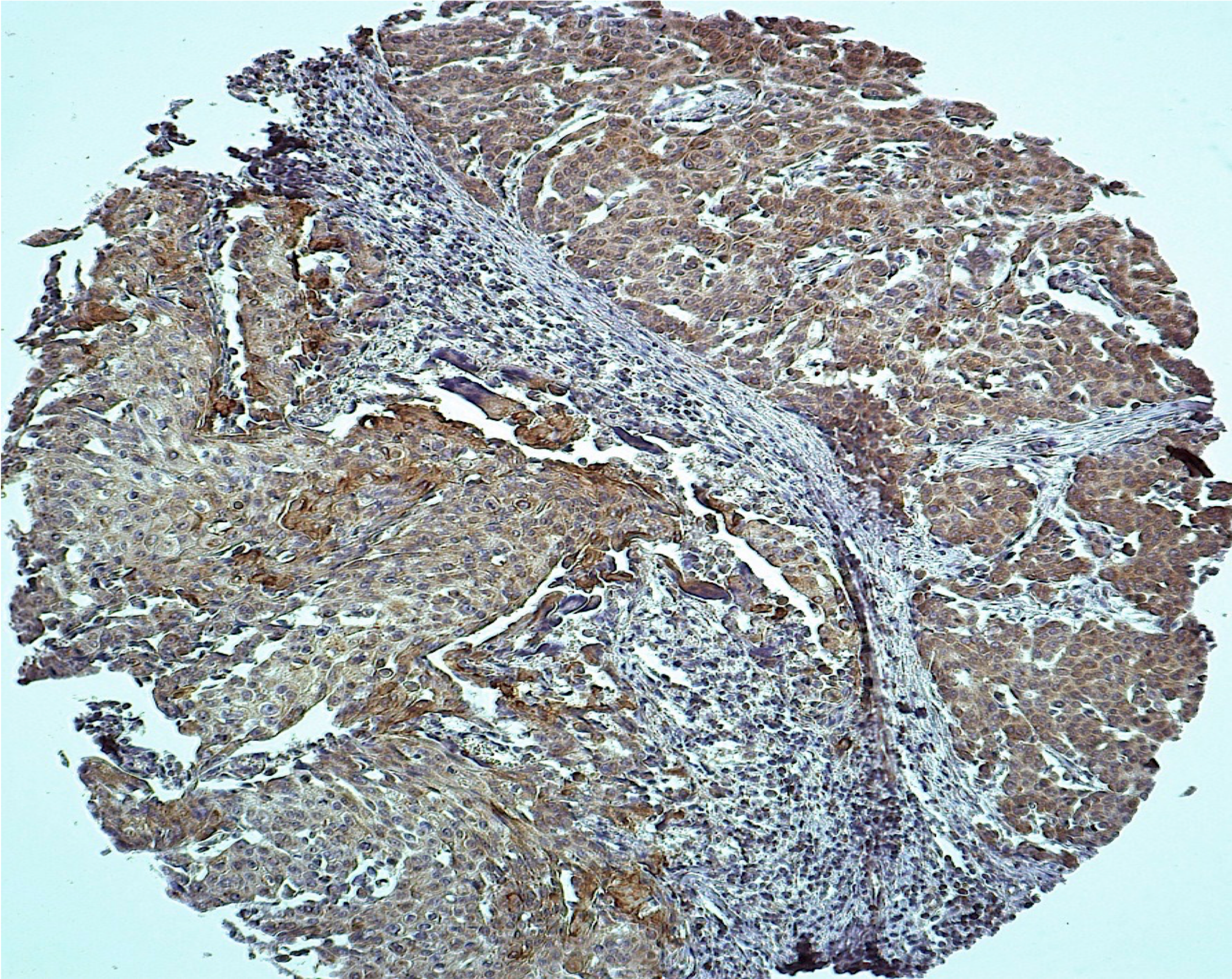

Supplement: S9 File — (ZIP) [file pone.0349359.s009.zip › Figure S3A AKT2 right 10x.pdf]

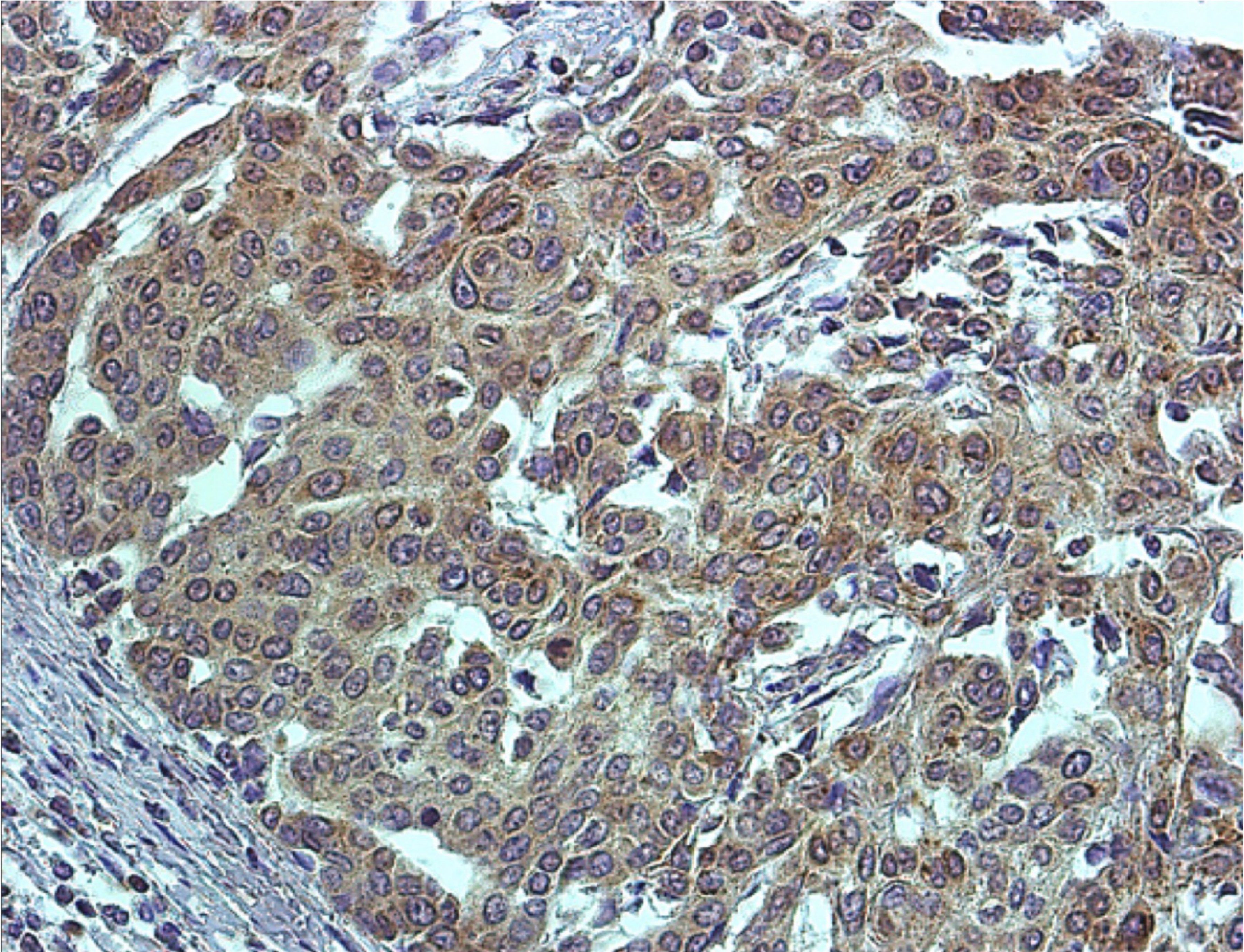

Supplement: S9 File — (ZIP) [file pone.0349359.s009.zip › Figure S3A AKT2 right 40x.pdf]

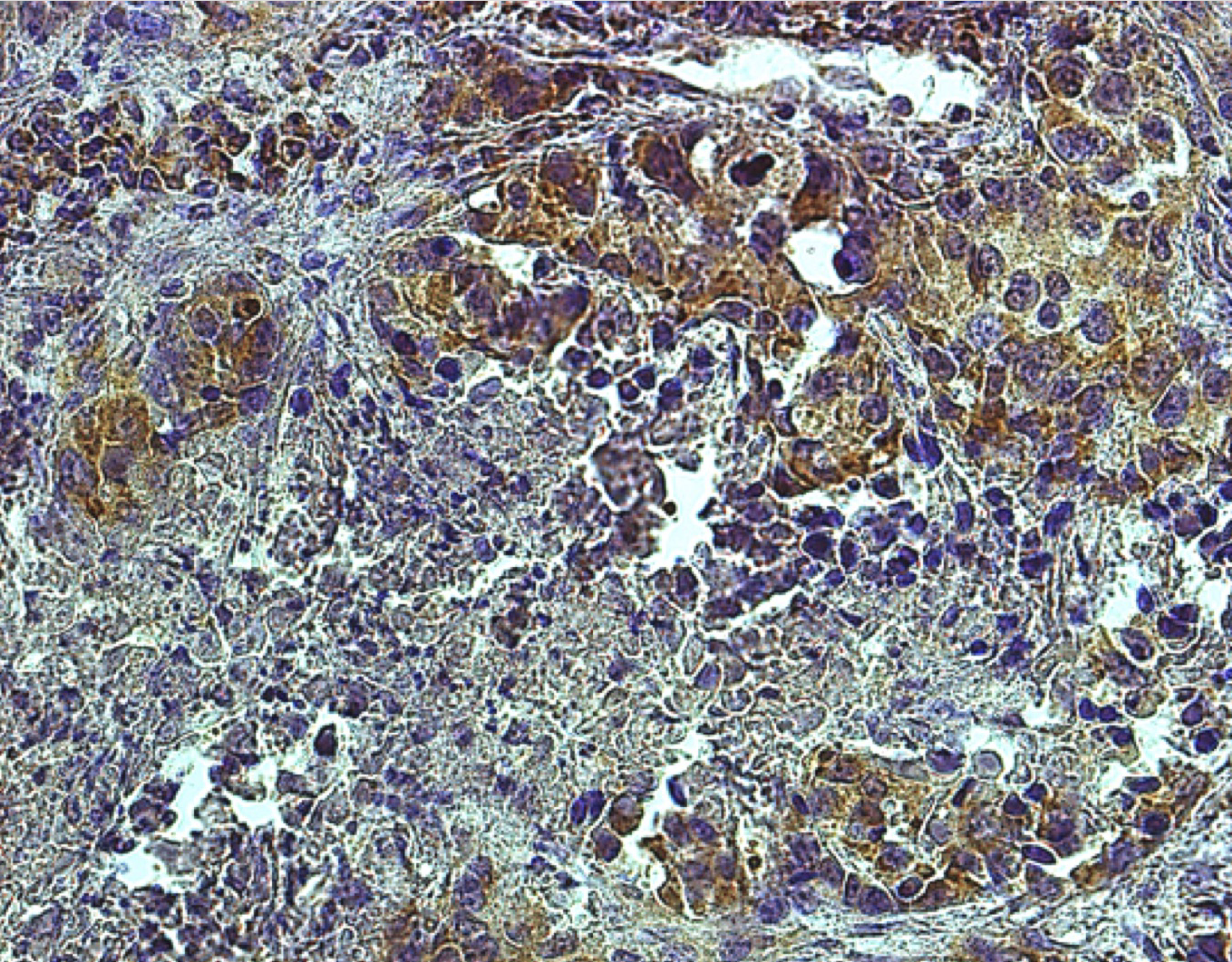

Supplement: S10 File — (ZIP) [file pone.0349359.s010.zip › Figure S3B AKT2 right 40x.tif]

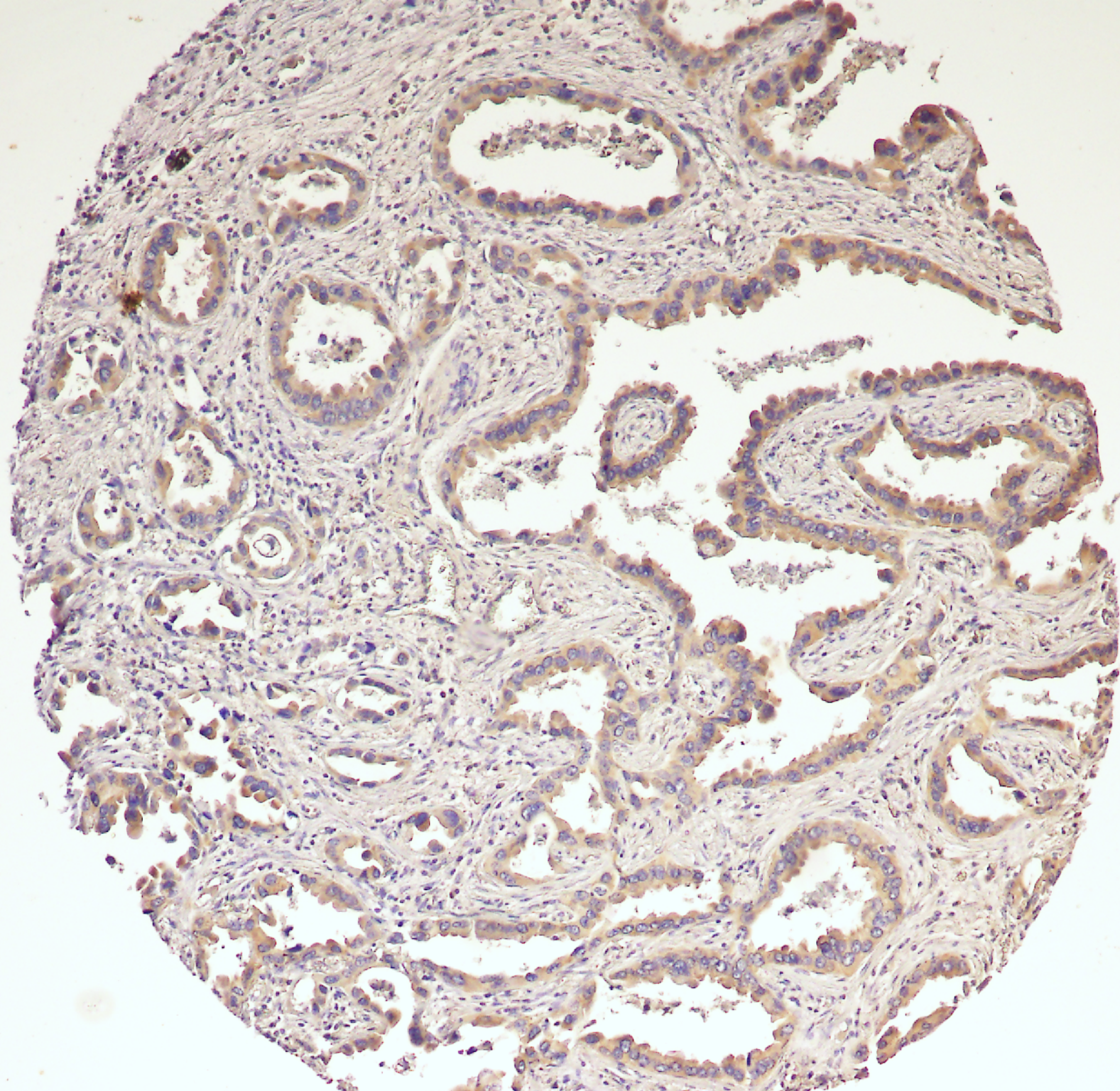

Supplement: S10 File — (ZIP) [file pone.0349359.s010.zip › Figure S3B AKT2 (+) 10x.pdf]

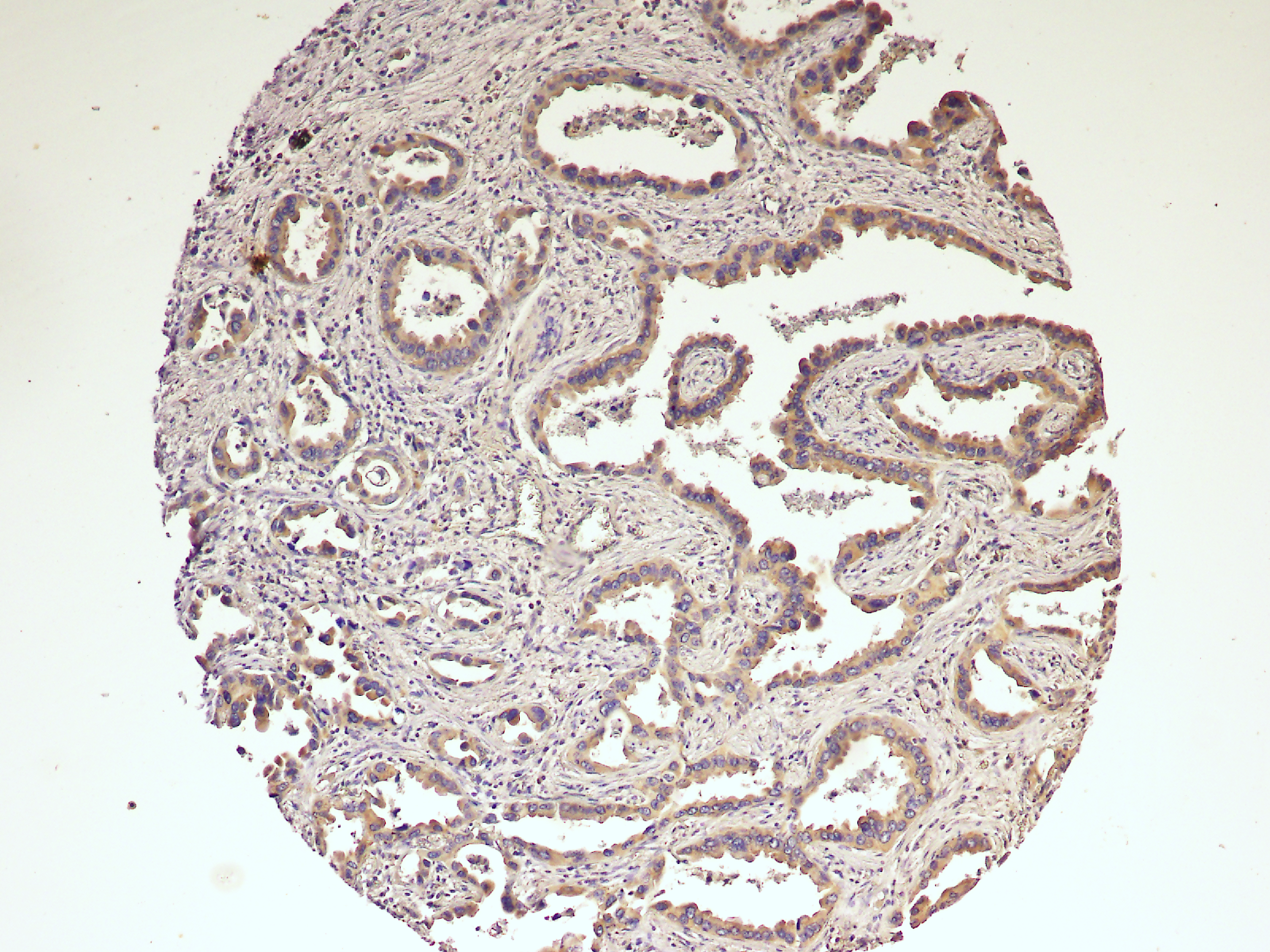

Supplement: S10 File — (ZIP) [file pone.0349359.s010.zip › Figure S3B AKT2 (+) 10x.TIF]

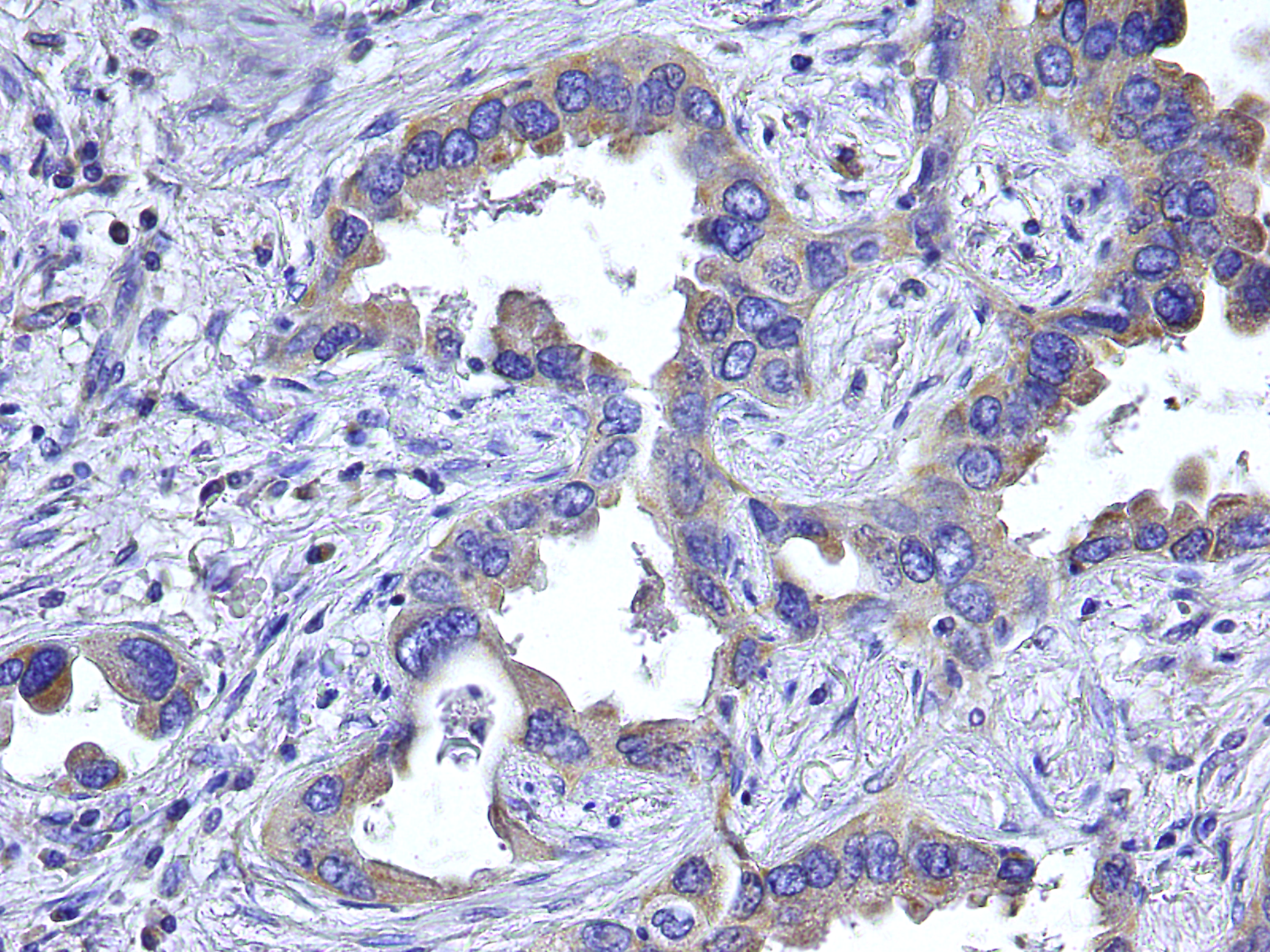

Supplement: S10 File — (ZIP) [file pone.0349359.s010.zip › Figure S3B AKT2 (+) 40x.pdf]

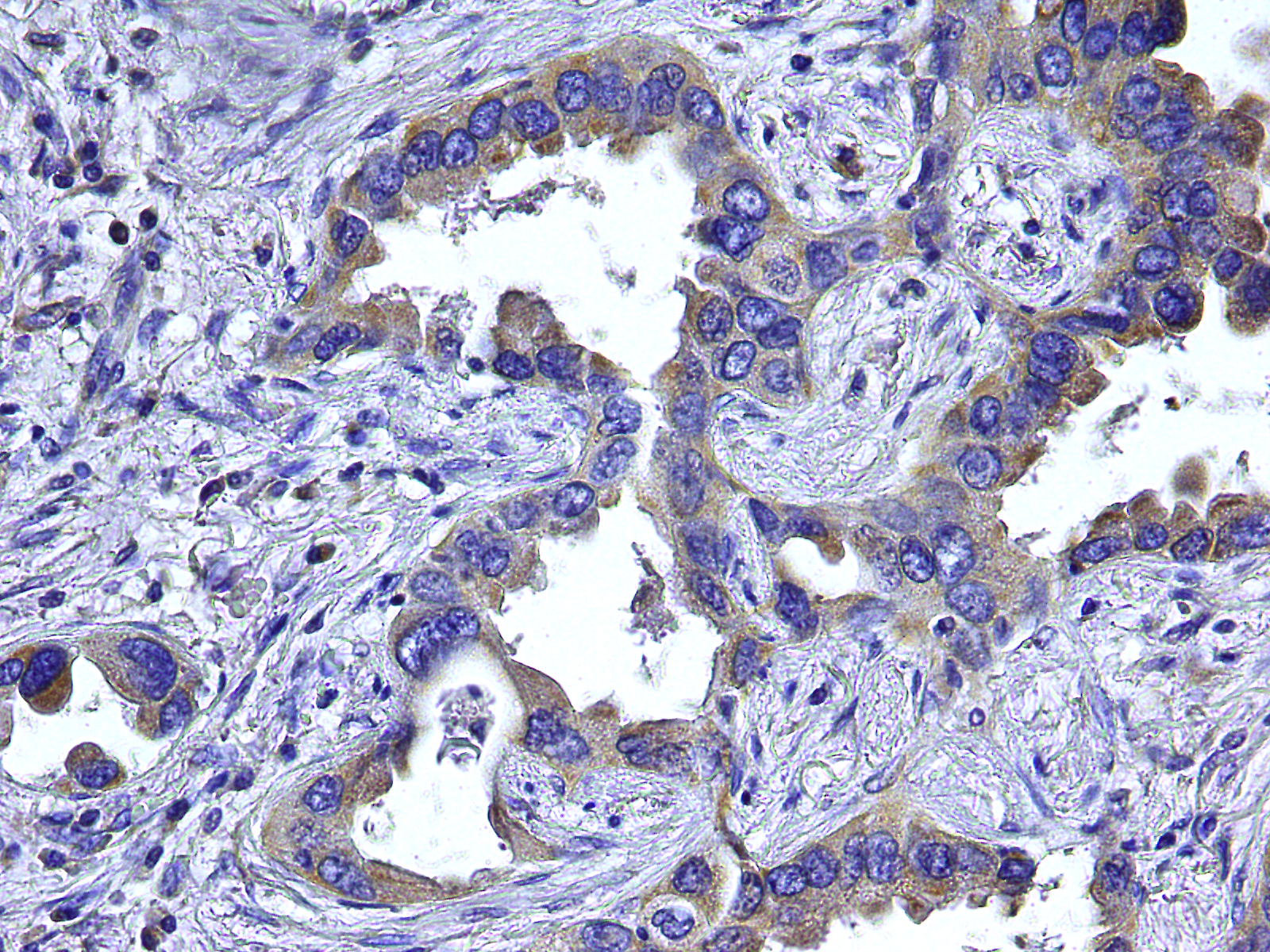

Supplement: S10 File — (ZIP) [file pone.0349359.s010.zip › Figure S3B AKT2 (+) 40x.TIF]

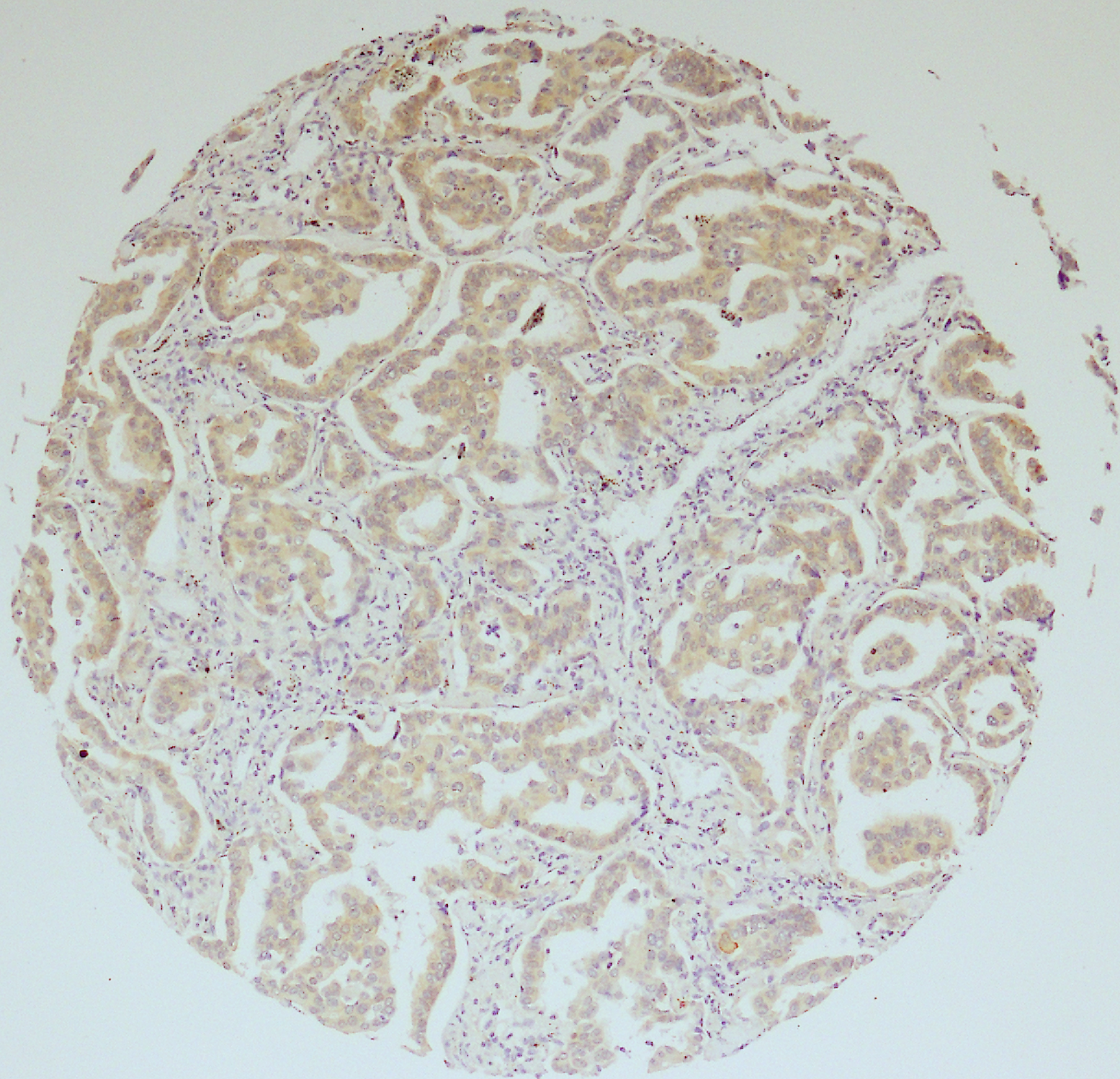

Supplement: S10 File — (ZIP) [file pone.0349359.s010.zip › Figure S3B AKT2 (++) 10x.pdf]

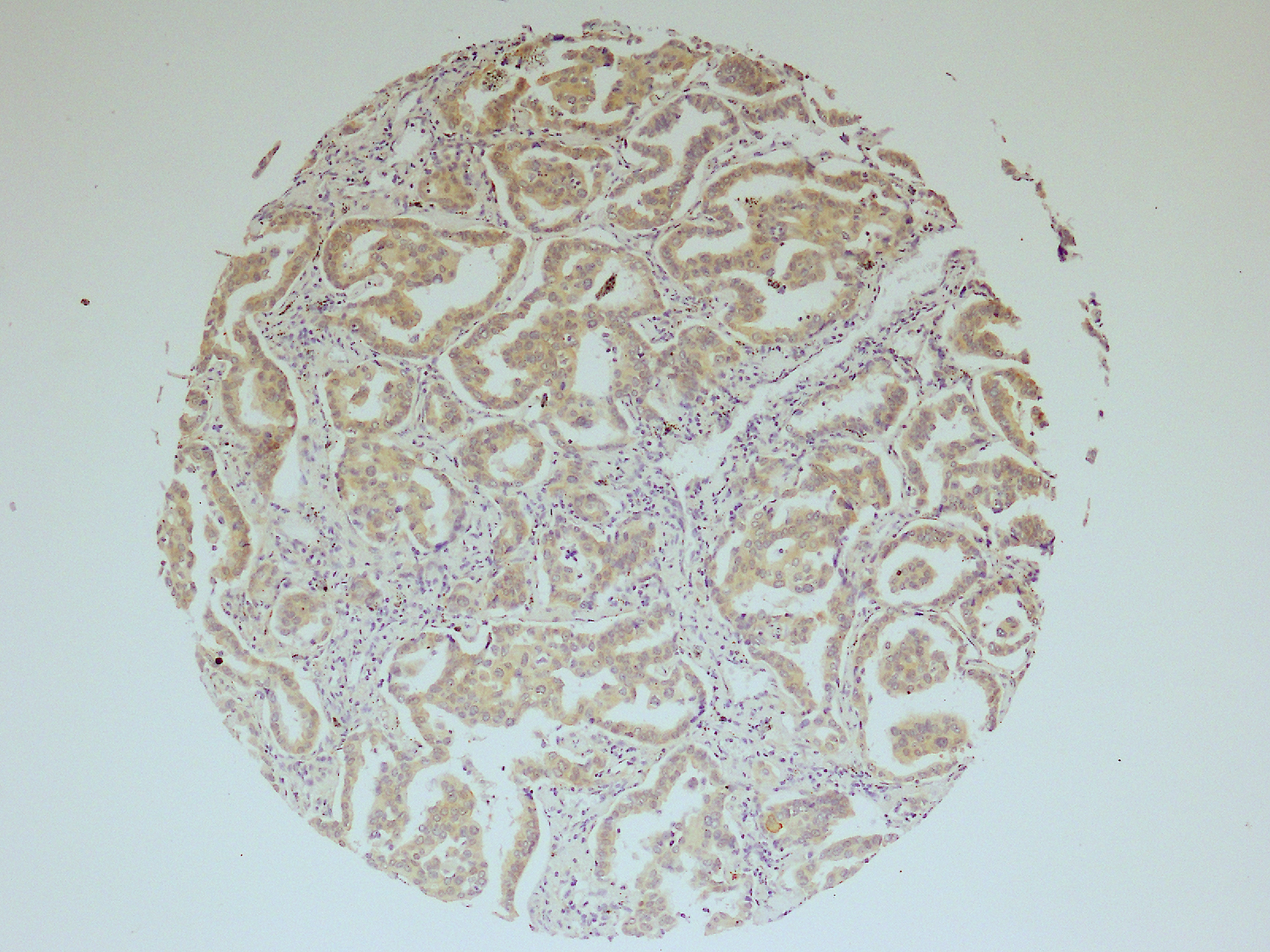

Supplement: S10 File — (ZIP) [file pone.0349359.s010.zip › Figure S3B AKT2 (++) 10x.TIF]

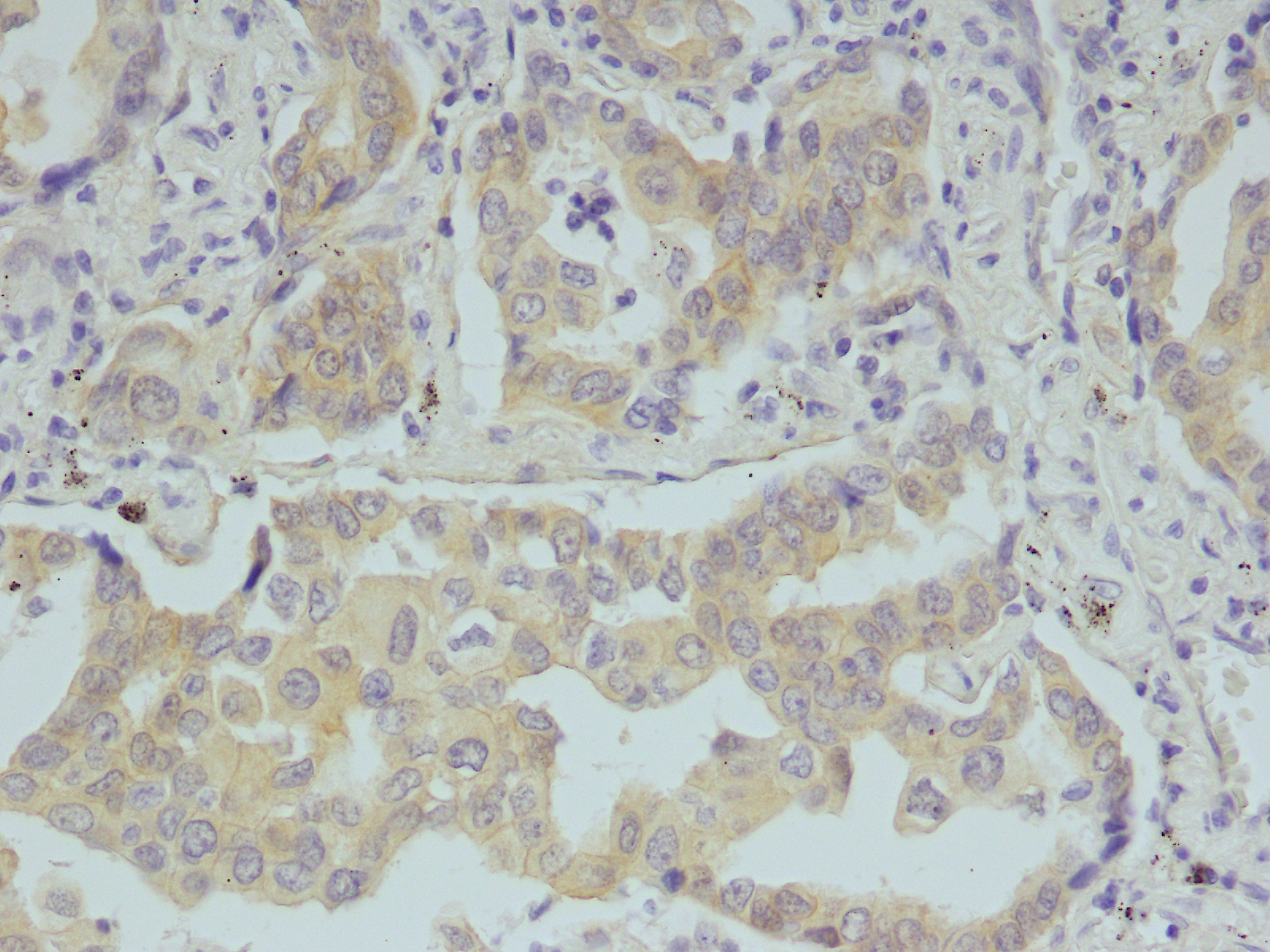

Supplement: S10 File — (ZIP) [file pone.0349359.s010.zip › Figure S3B AKT2 (++) 40x.pdf]

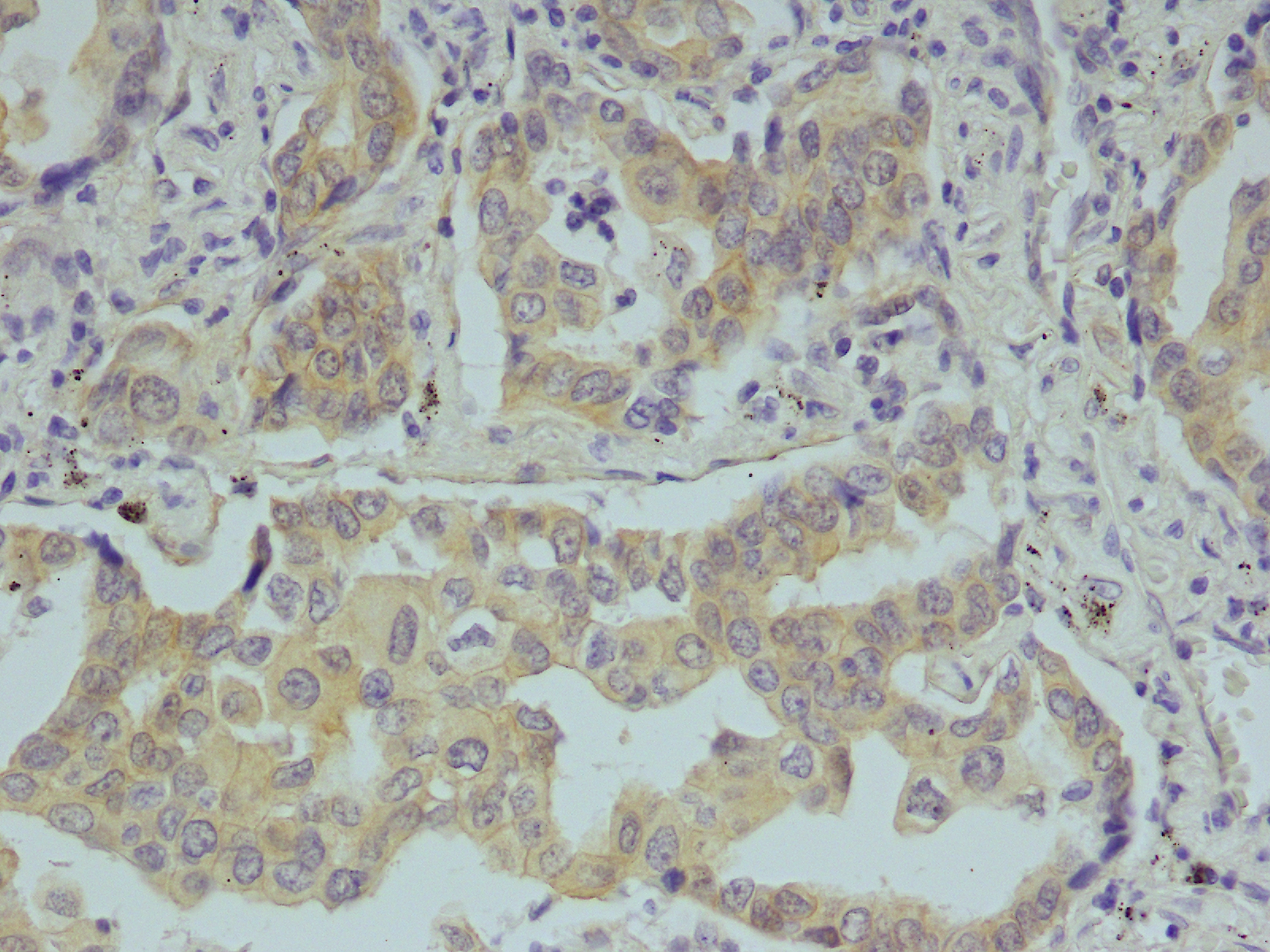

Supplement: S10 File — (ZIP) [file pone.0349359.s010.zip › Figure S3B AKT2 (++) 40x.TIF]

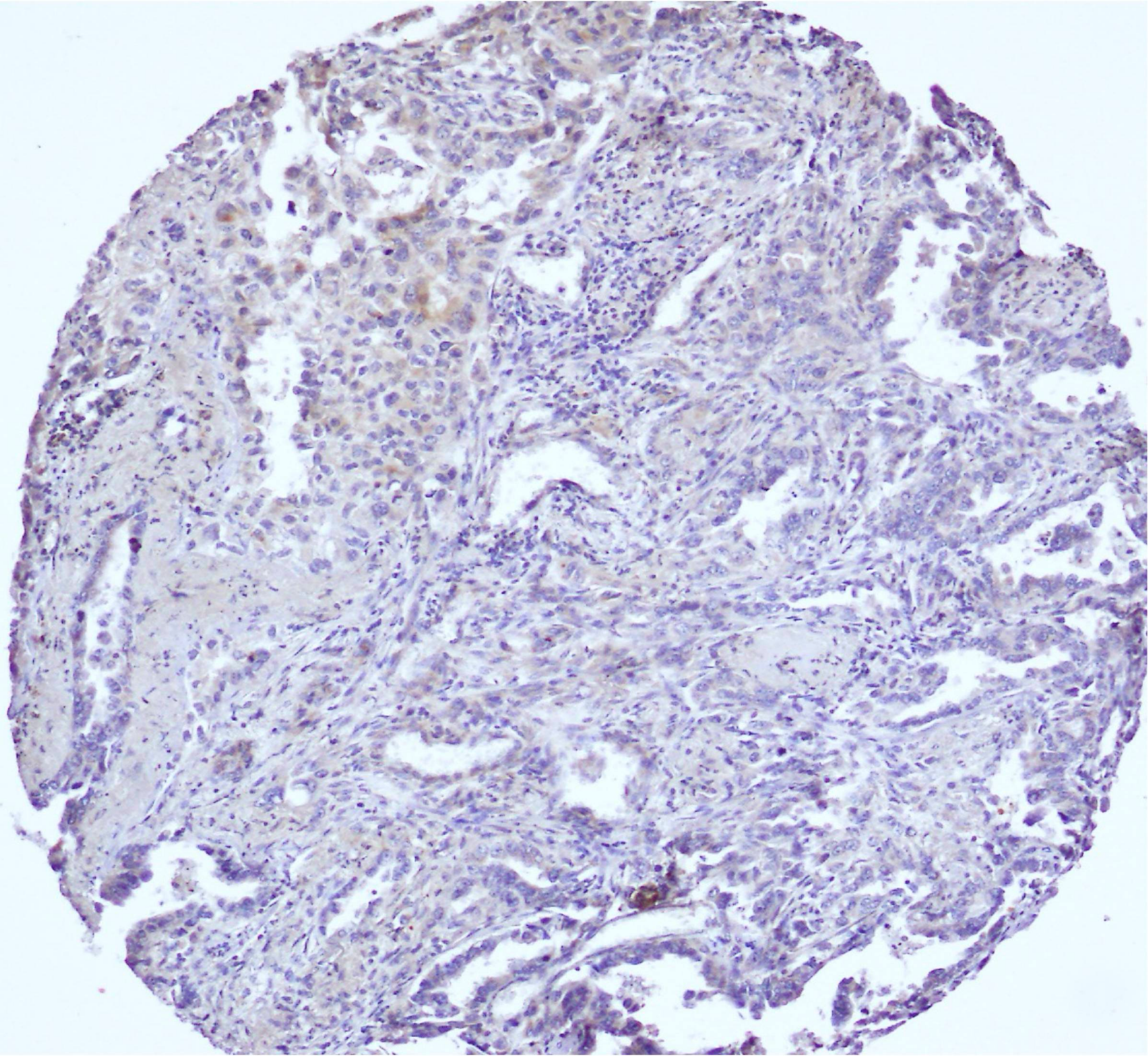

Supplement: S10 File — (ZIP) [file pone.0349359.s010.zip › Figure S3B AKT2 left 10x.pdf]

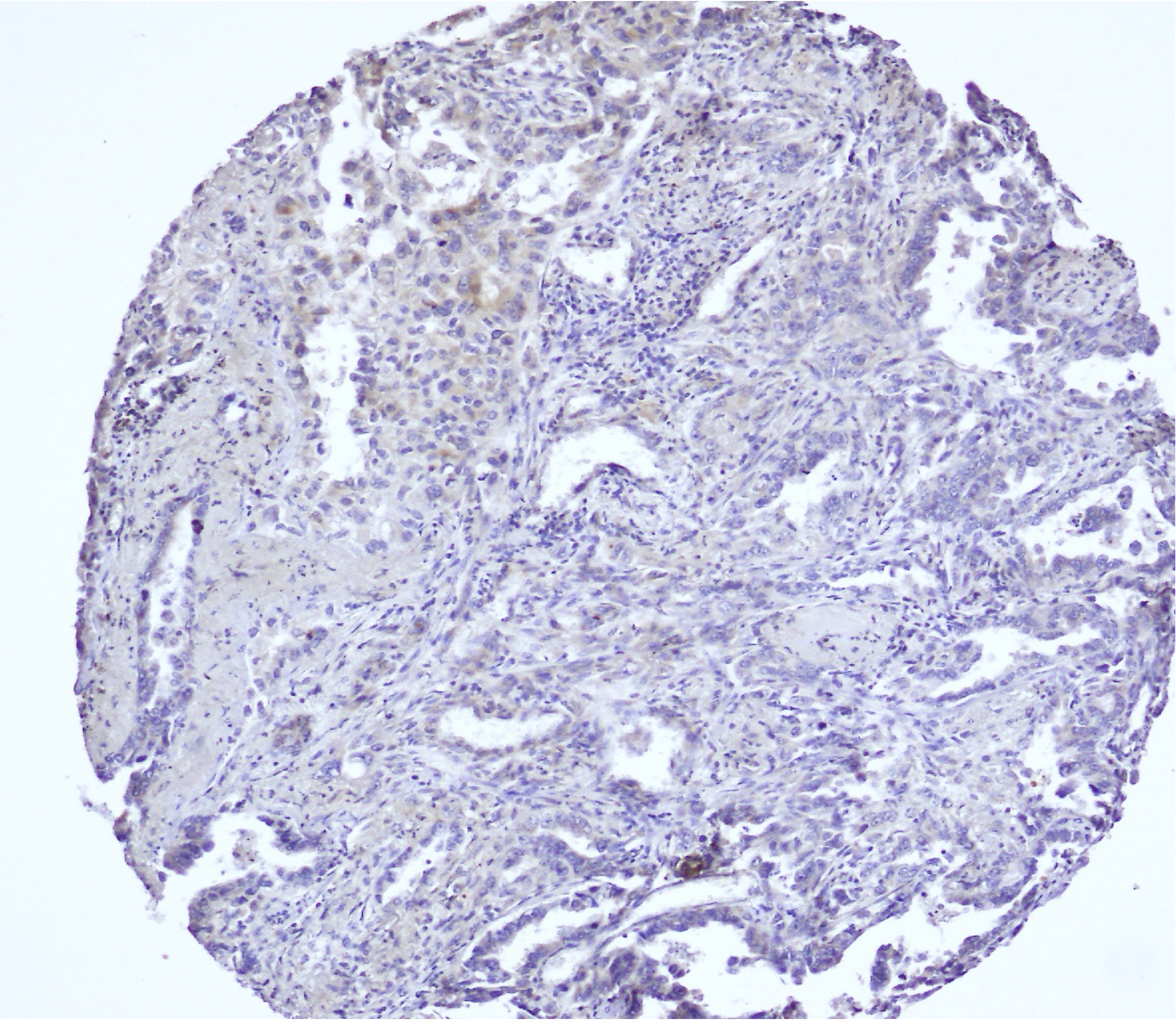

Supplement: S10 File — (ZIP) [file pone.0349359.s010.zip › Figure S3B AKT2 left 10x.tif]

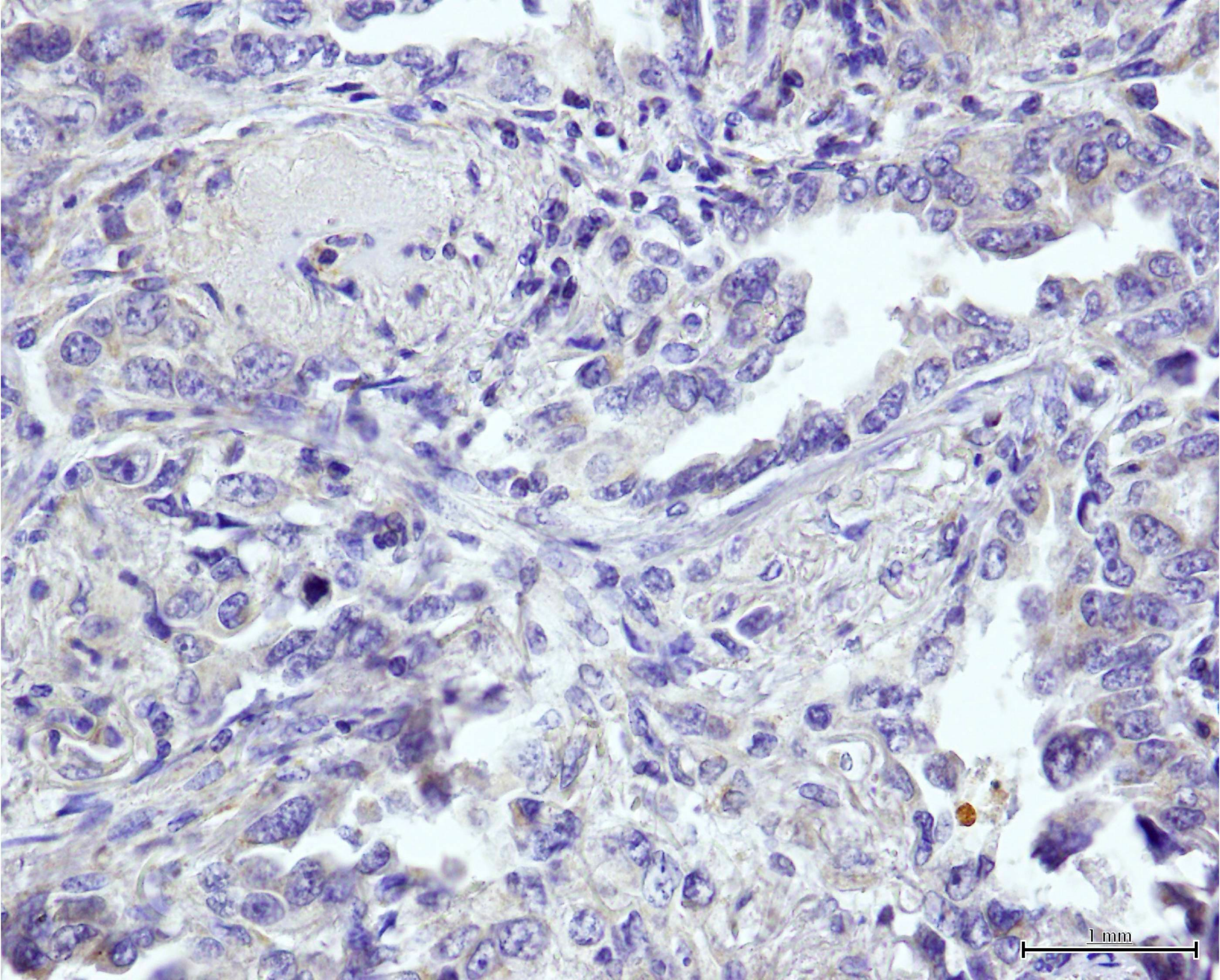

Supplement: S10 File — (ZIP) [file pone.0349359.s010.zip › Figure S3B AKT2 left 40x.pdf]

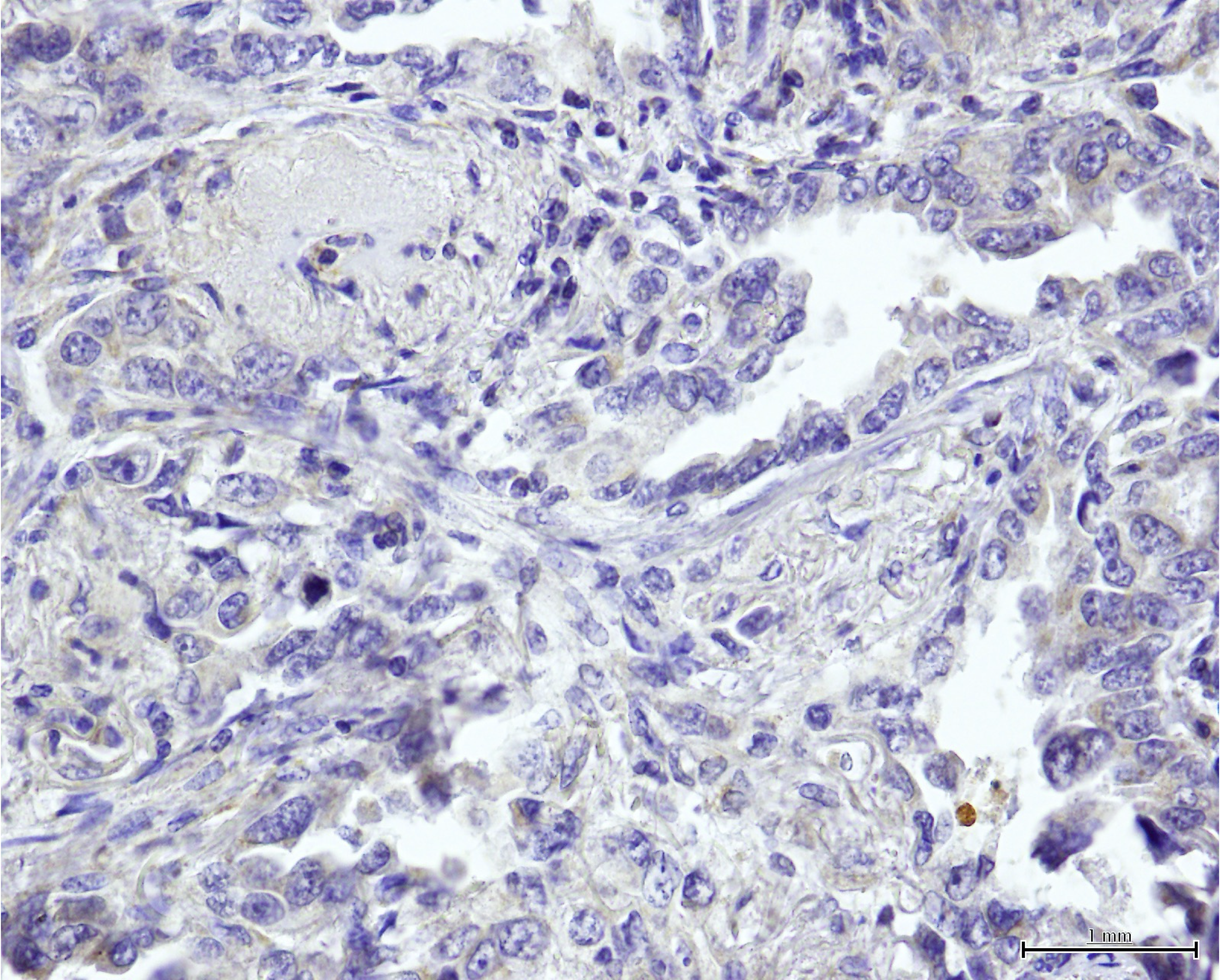

Supplement: S10 File — (ZIP) [file pone.0349359.s010.zip › Figure S3B AKT2 left 40x.tif]

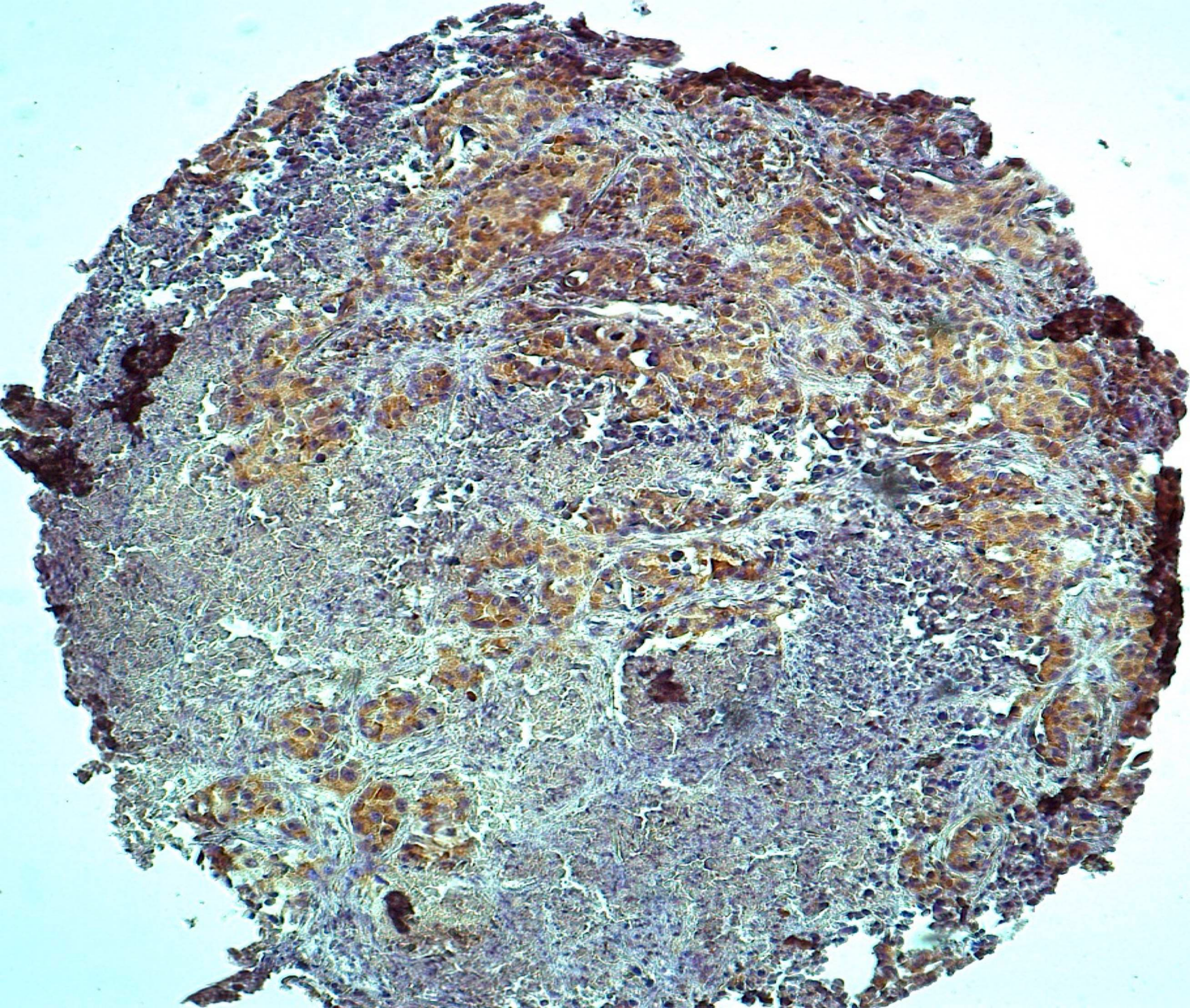

Supplement: S10 File — (ZIP) [file pone.0349359.s010.zip › Figure S3B AKT2 right 10x.pdf]

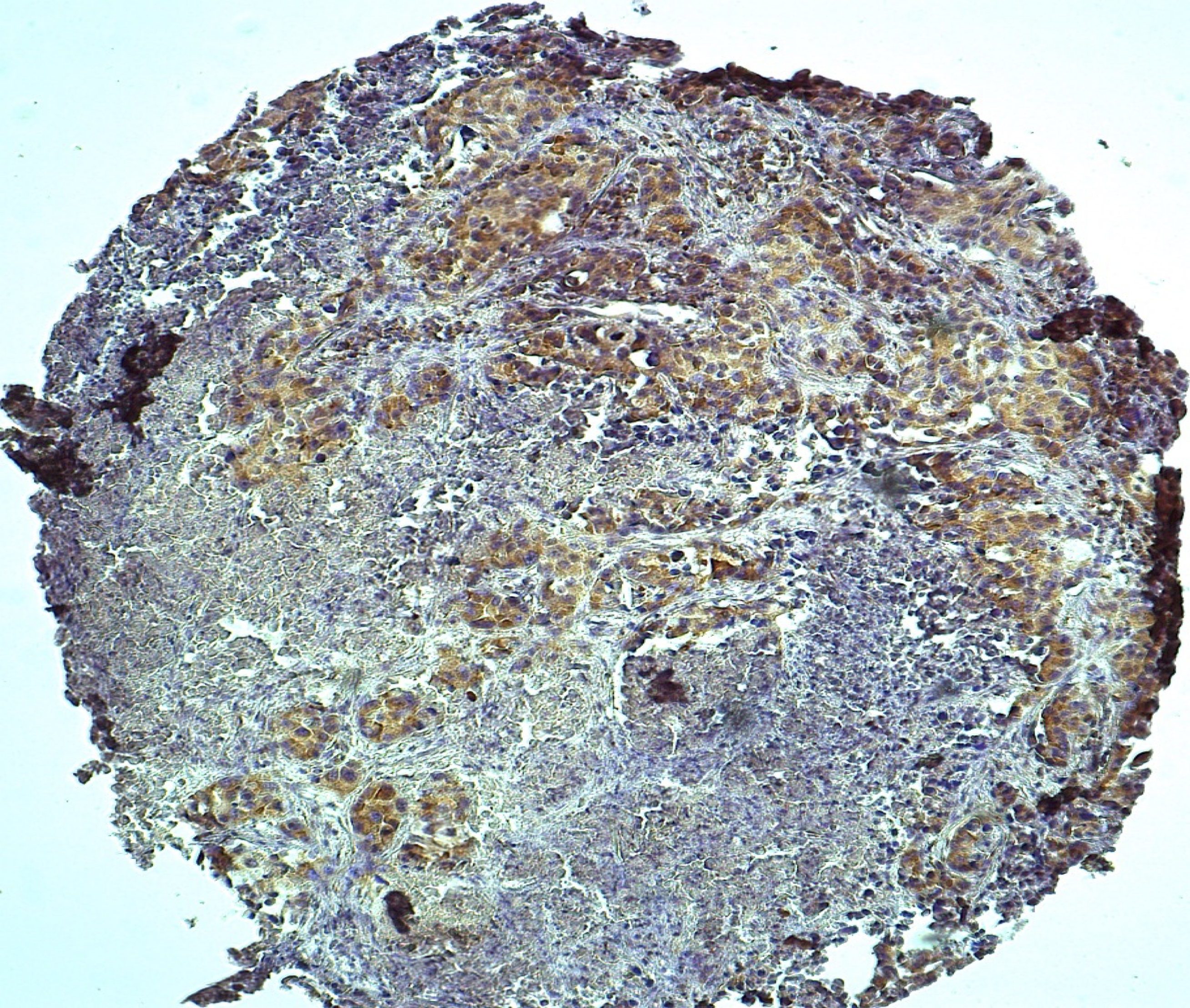

Supplement: S10 File — (ZIP) [file pone.0349359.s010.zip › Figure S3B AKT2 right 10x.tif]

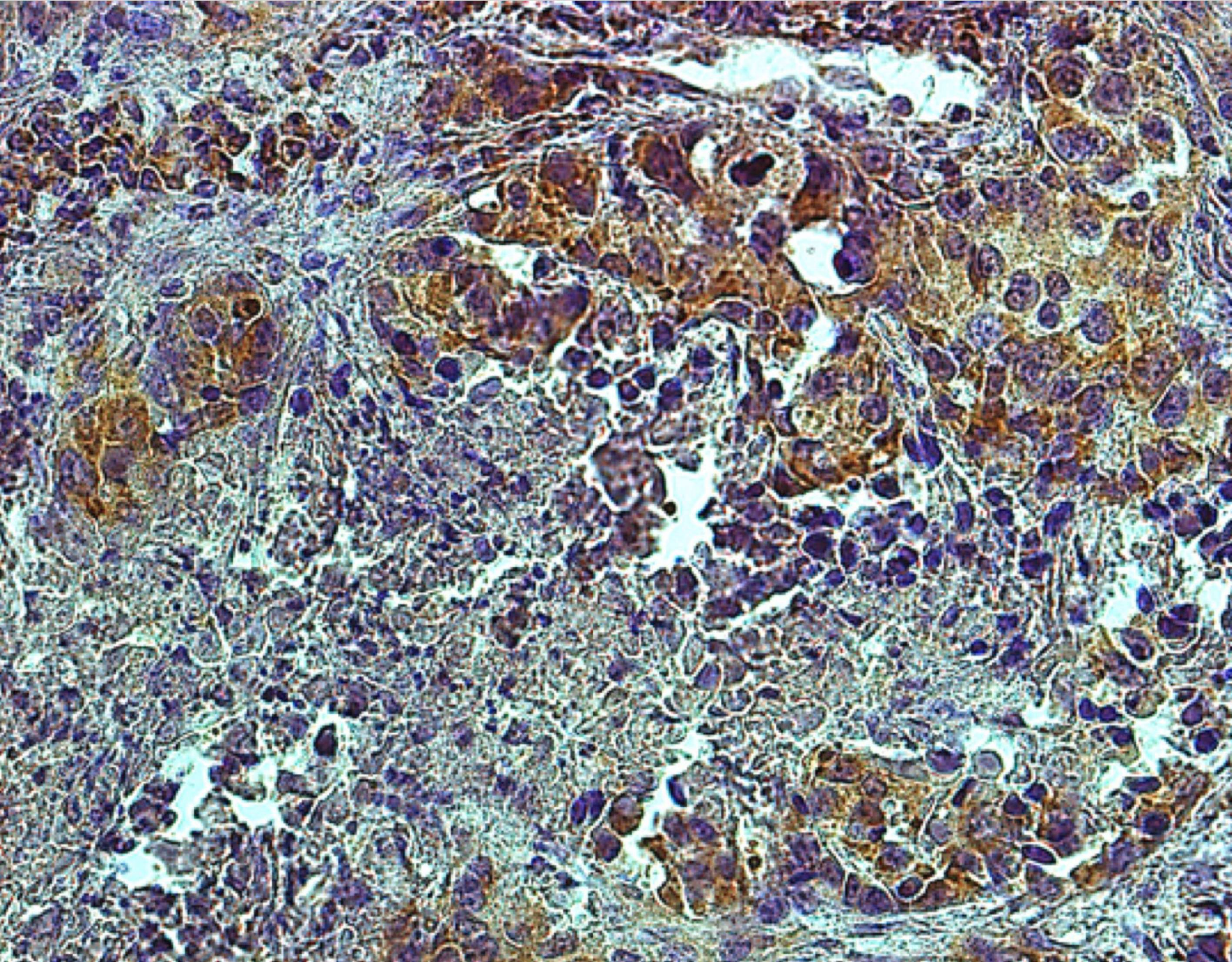

Supplement: S10 File — (ZIP) [file pone.0349359.s010.zip › Figure S3B AKT2 right 40x.pdf]

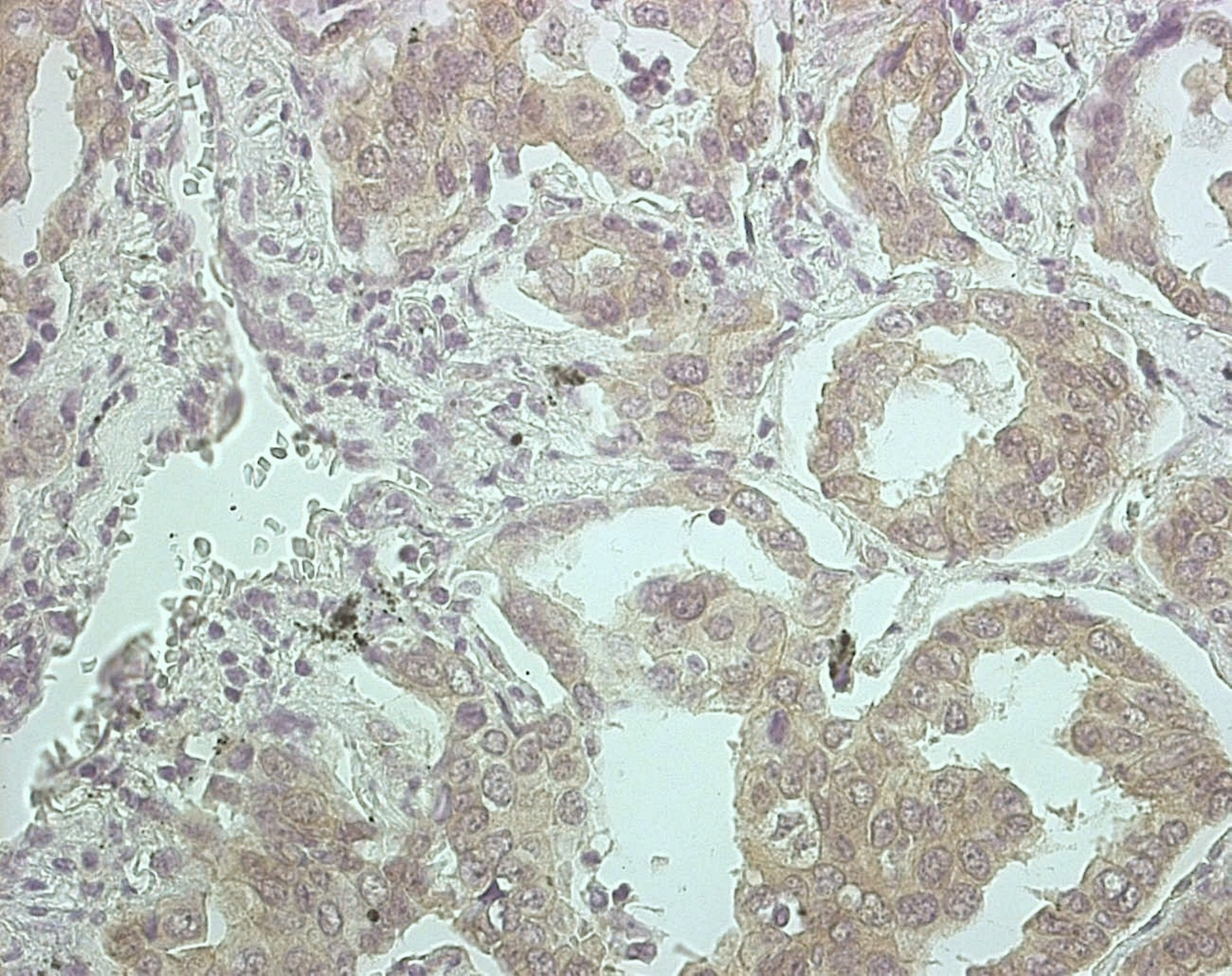

Supplement: S11 File — (ZIP) [file pone.0349359.s011.zip › Figure S4B PIK3CA right 40x.pdf]

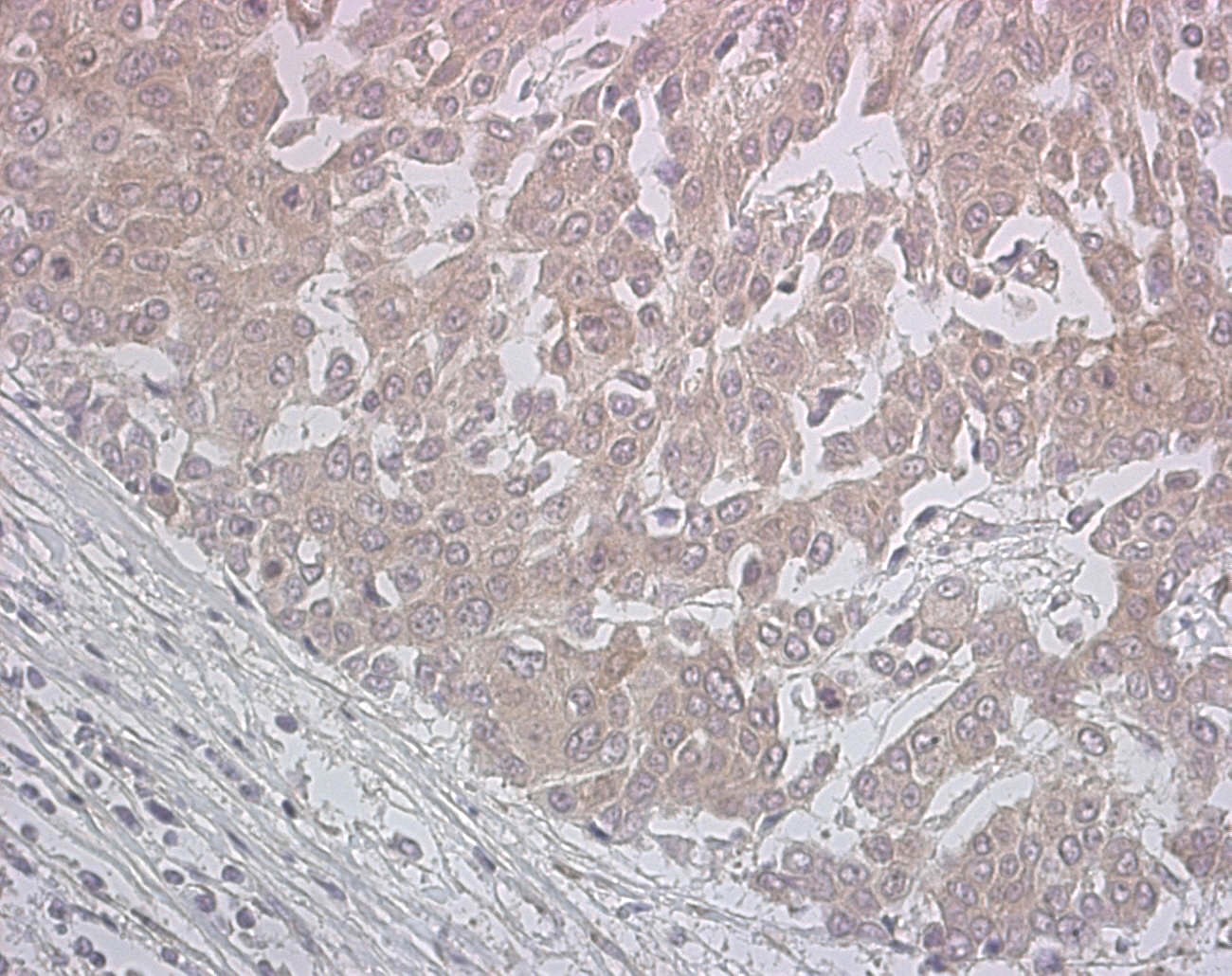

Supplement: S11 File — (ZIP) [file pone.0349359.s011.zip › Figure S4A PI3KCA right 40x.jpg]

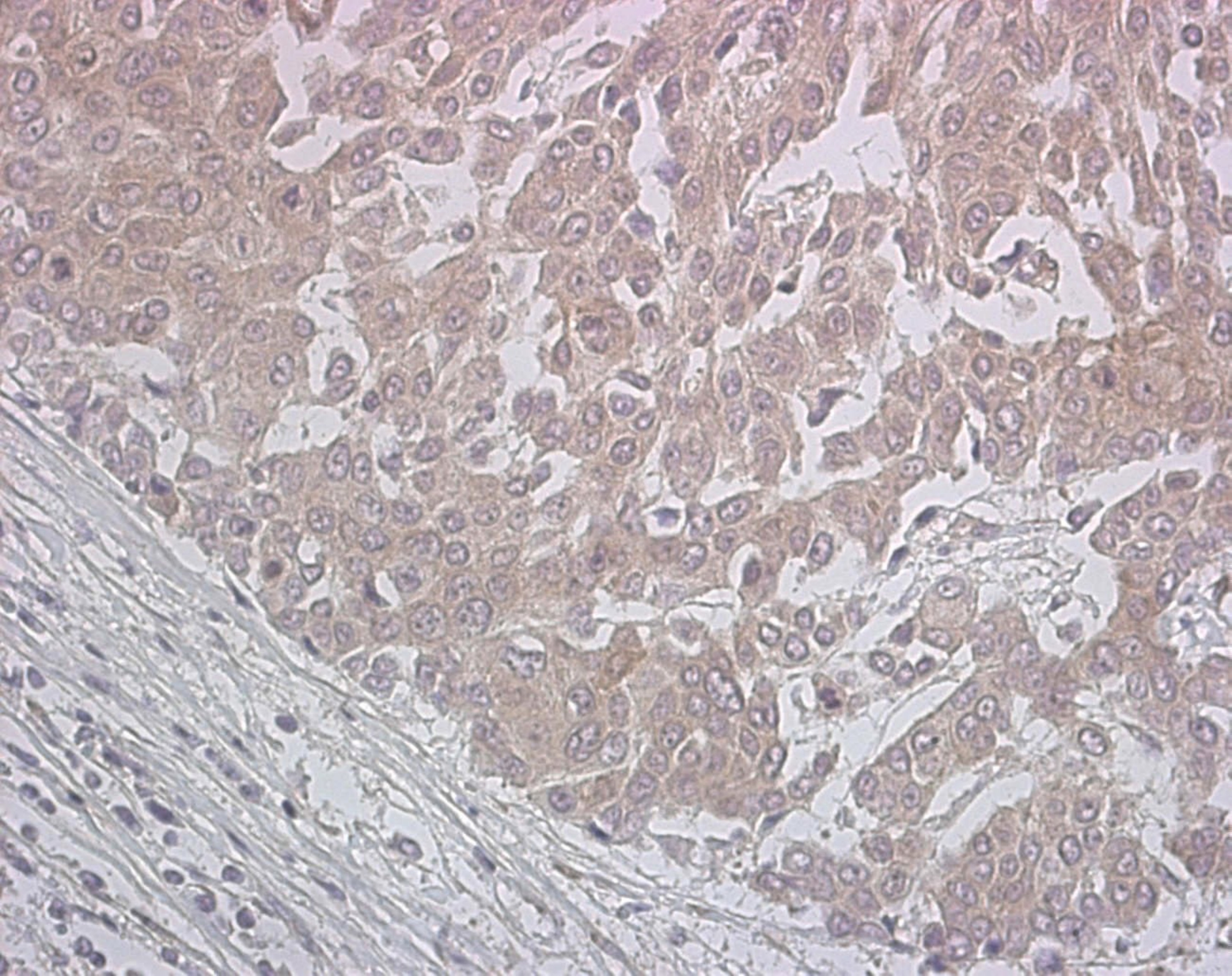

Supplement: S11 File — (ZIP) [file pone.0349359.s011.zip › Figure S4A PI3KCA right 40x.pdf]

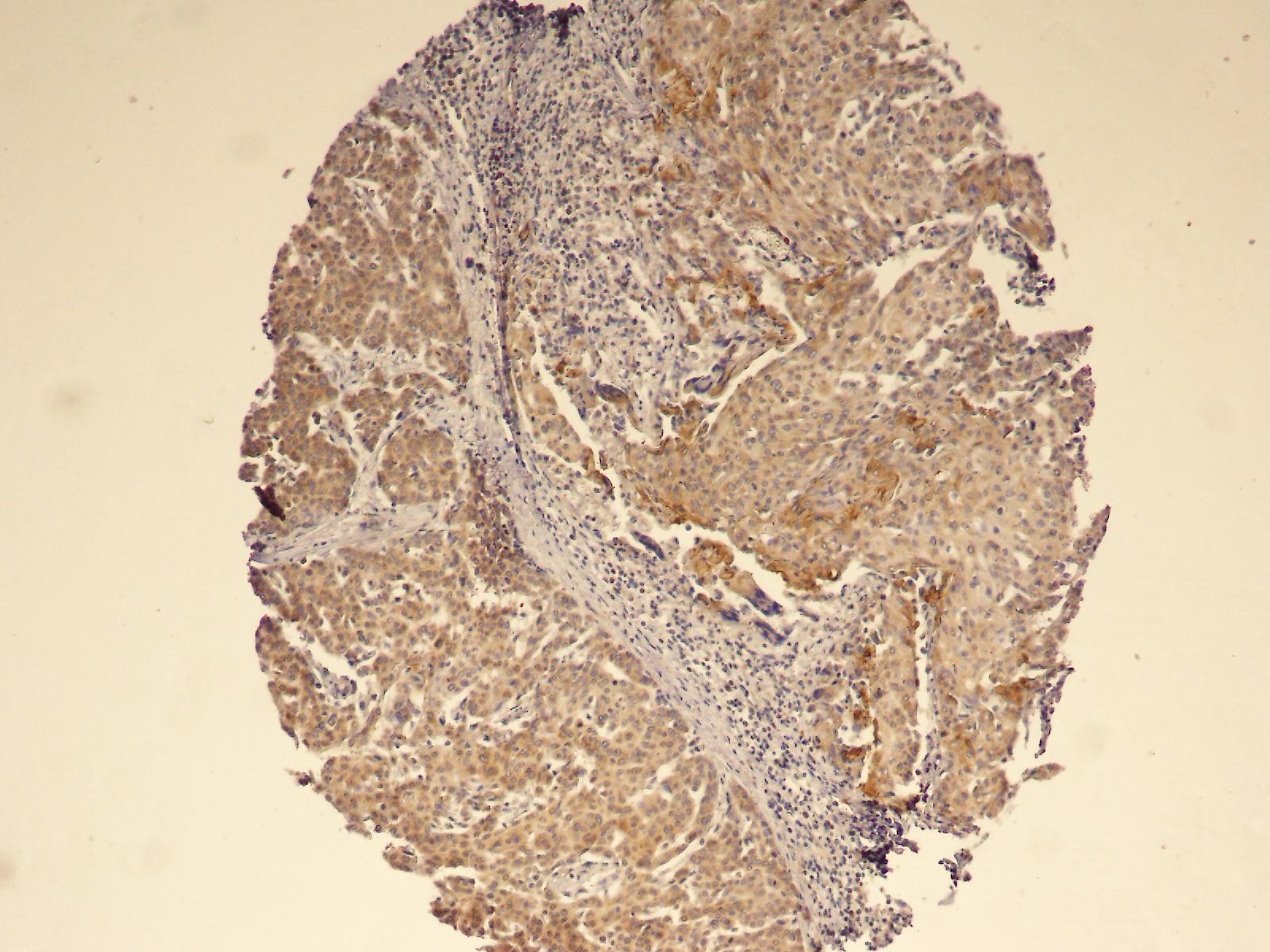

Supplement: S11 File — (ZIP) [file pone.0349359.s011.zip › Figure S4A PI3KCA(++) 10x.TIF]

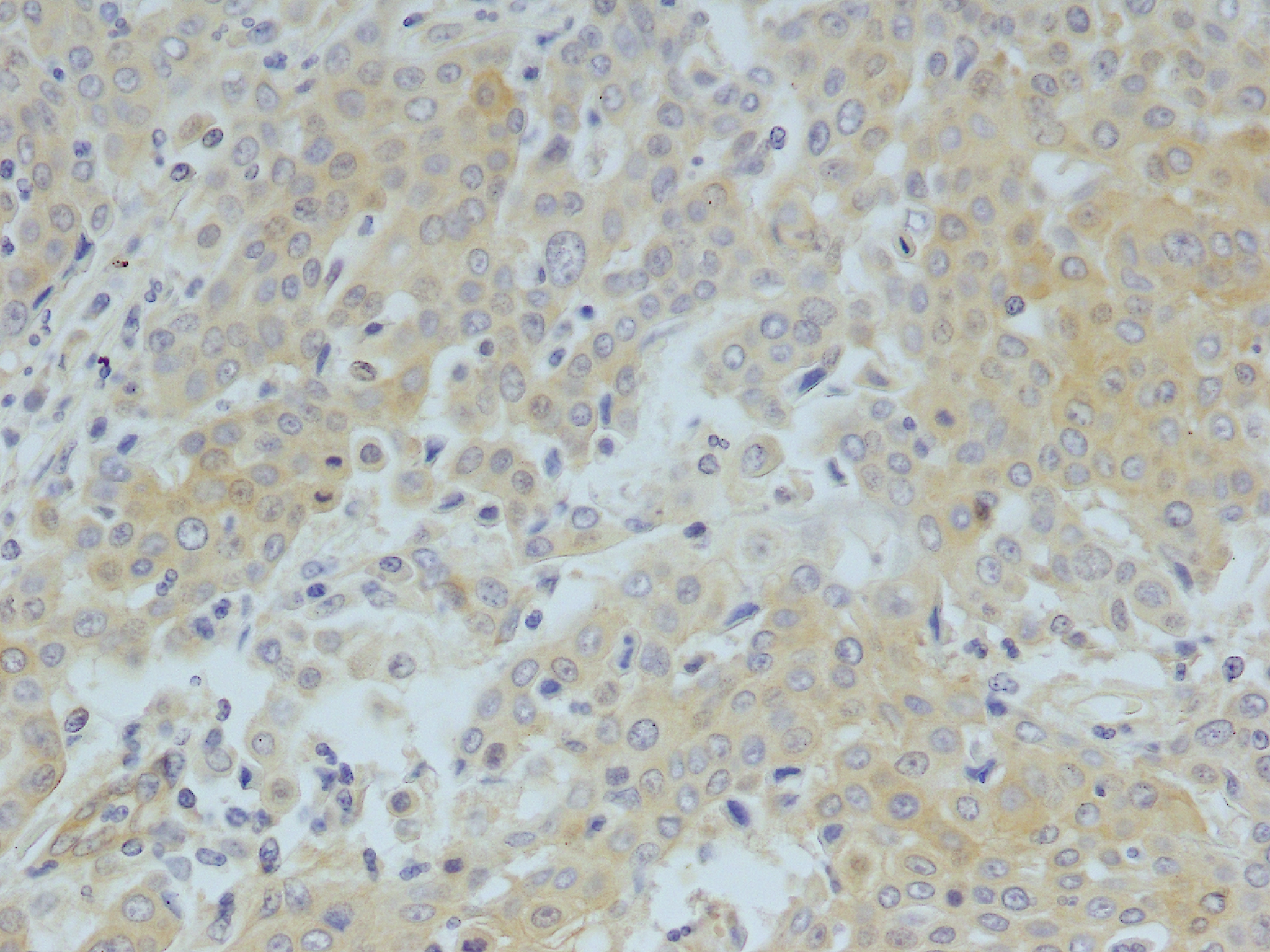

Supplement: S11 File — (ZIP) [file pone.0349359.s011.zip › Figure S4A PIK3CA (+) 40x.pdf]

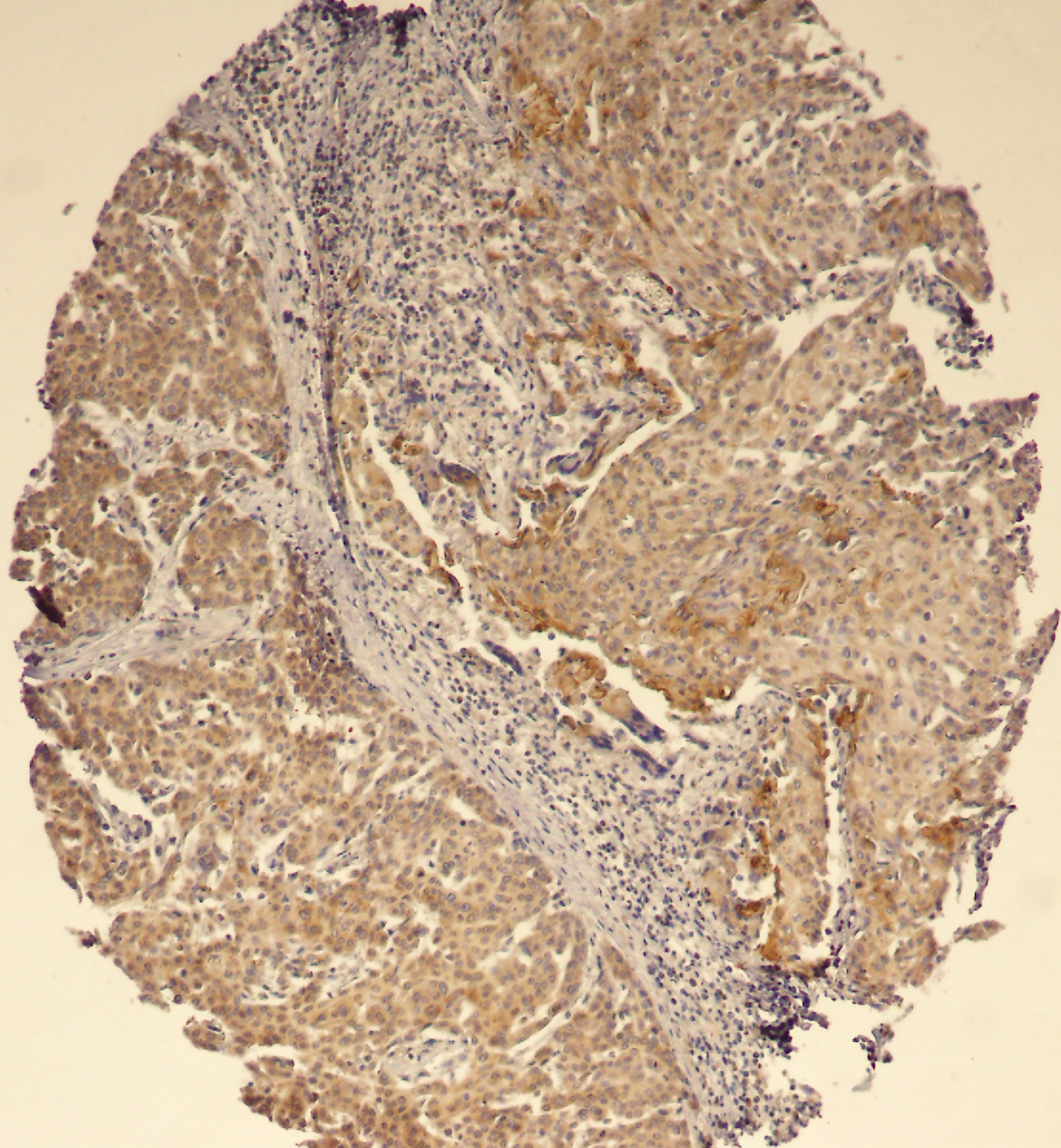

Supplement: S11 File — (ZIP) [file pone.0349359.s011.zip › Figure S4A PIK3CA (++) 10x.pdf]

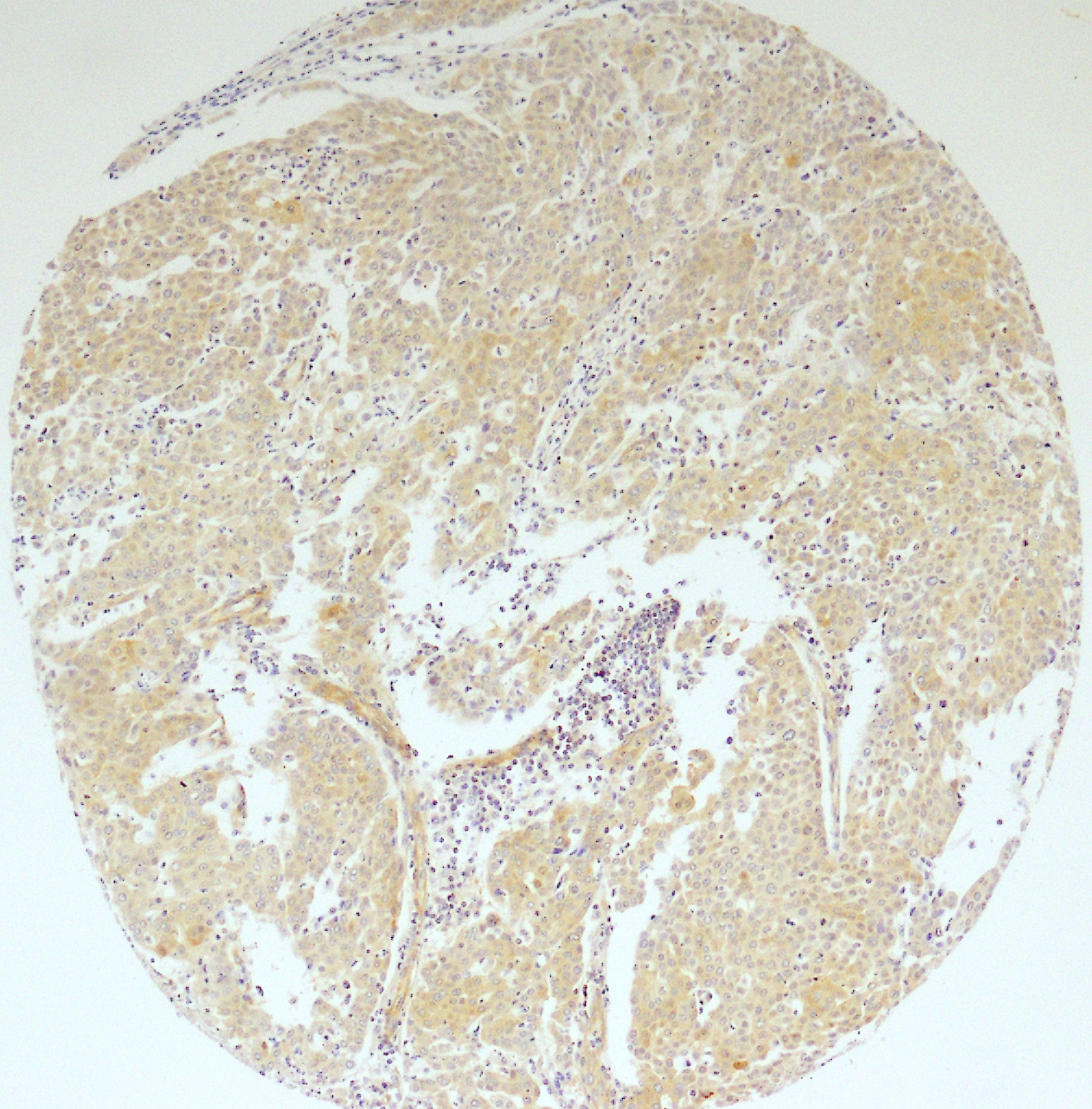

Supplement: S11 File — (ZIP) [file pone.0349359.s011.zip › Figure S4A PIK3CA (+) 10x.pdf]

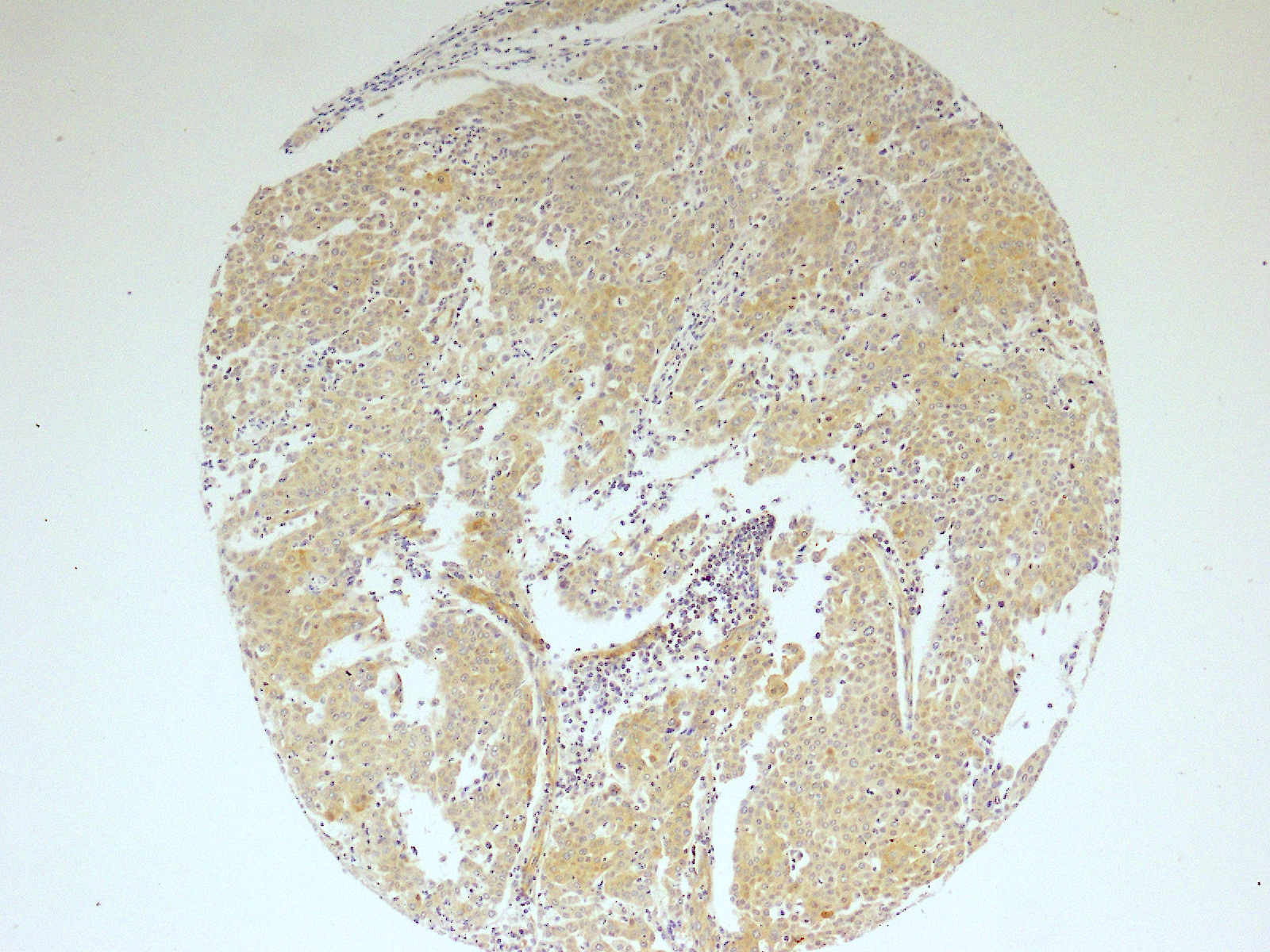

Supplement: S11 File — (ZIP) [file pone.0349359.s011.zip › Figure S4A PIK3CA (+) 10x.TIF]

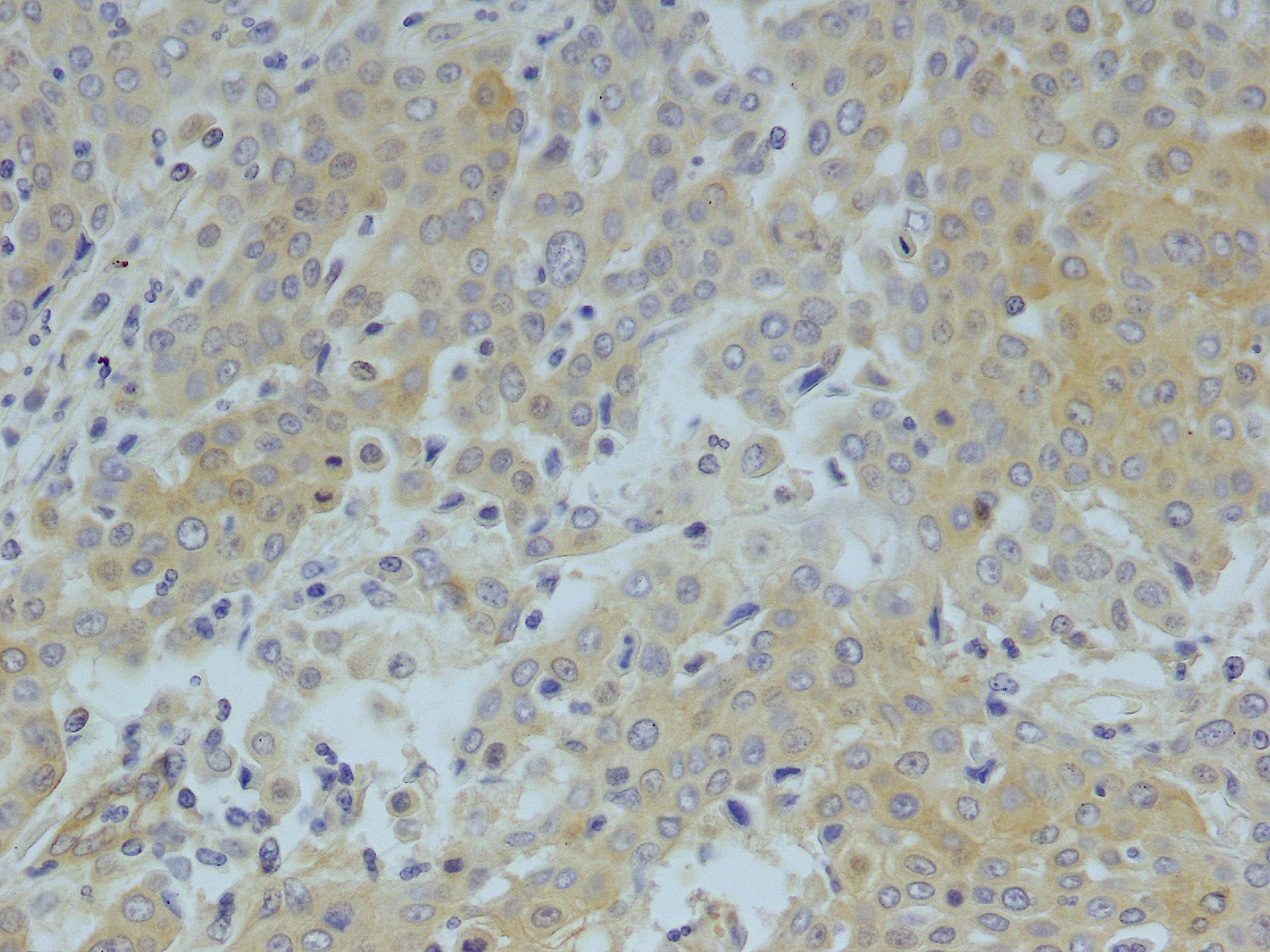

Supplement: S11 File — (ZIP) [file pone.0349359.s011.zip › Figure S4A PIK3CA (+) 40x.TIF]

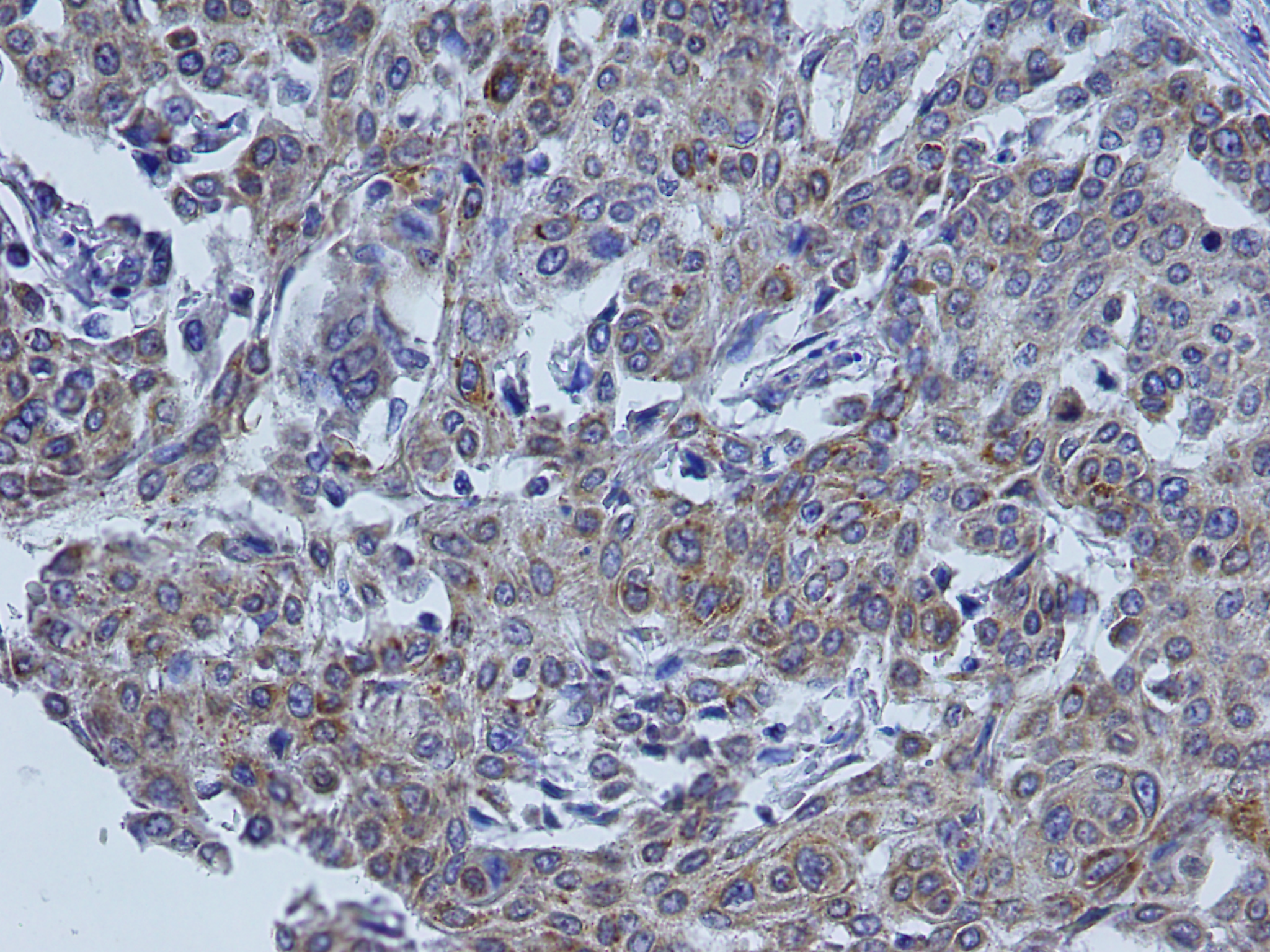

Supplement: S11 File — (ZIP) [file pone.0349359.s011.zip › Figure S4A PIK3CA (++) 40x.pdf]

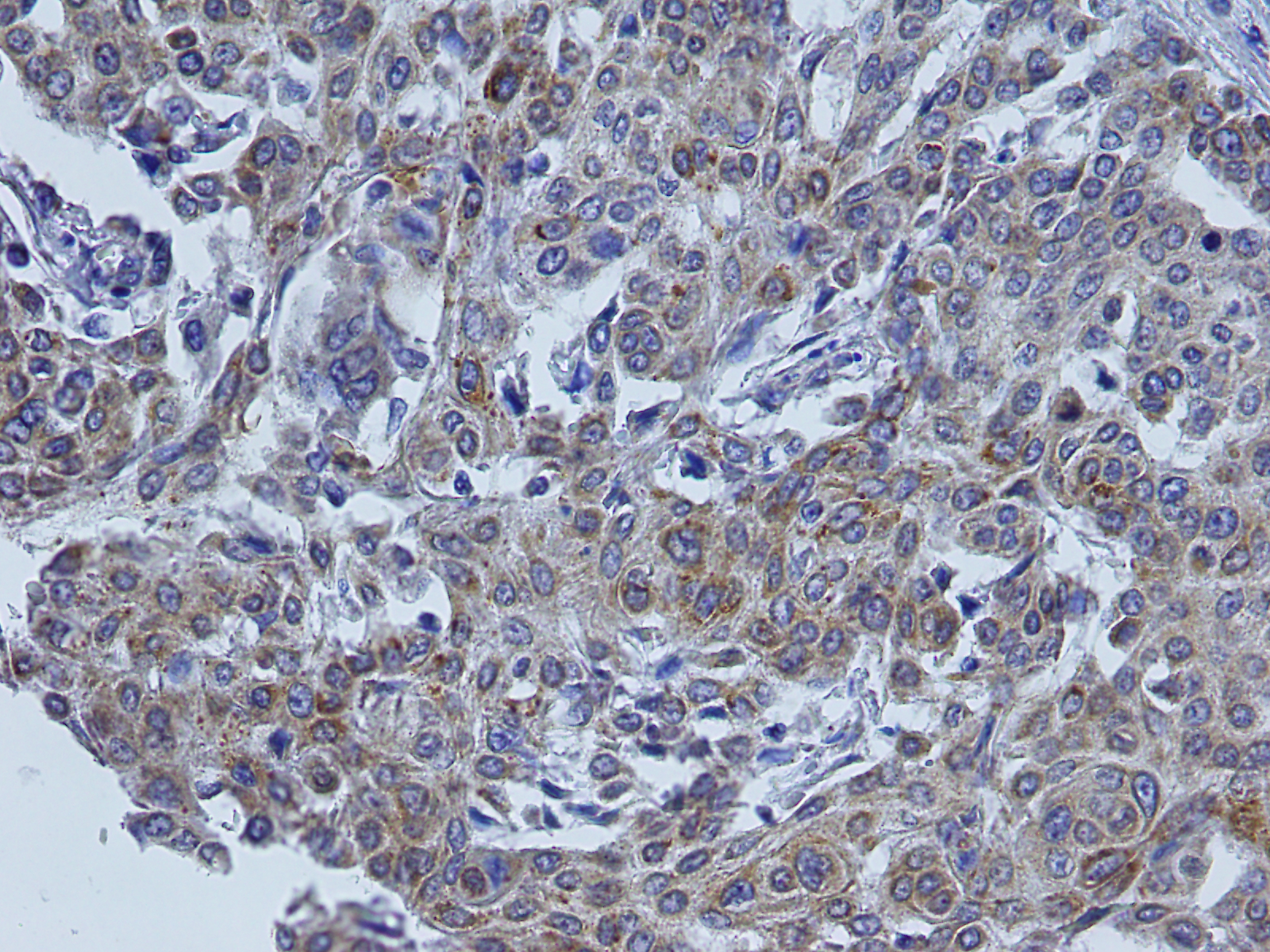

Supplement: S11 File — (ZIP) [file pone.0349359.s011.zip › Figure S4A PIK3CA (++) 40x.TIF]

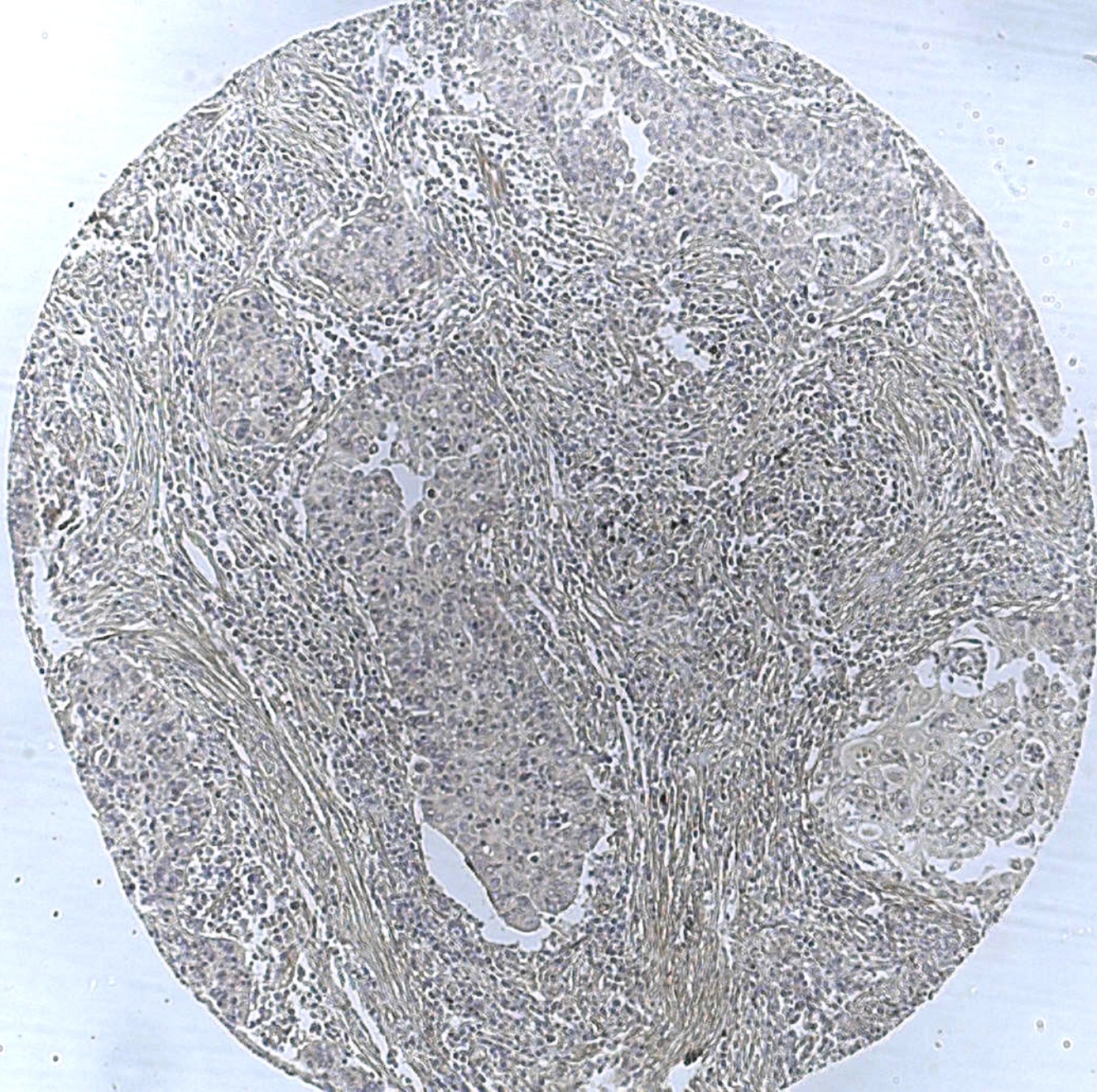

Supplement: S11 File — (ZIP) [file pone.0349359.s011.zip › Figure S4A PIK3CA left 10x.pdf]

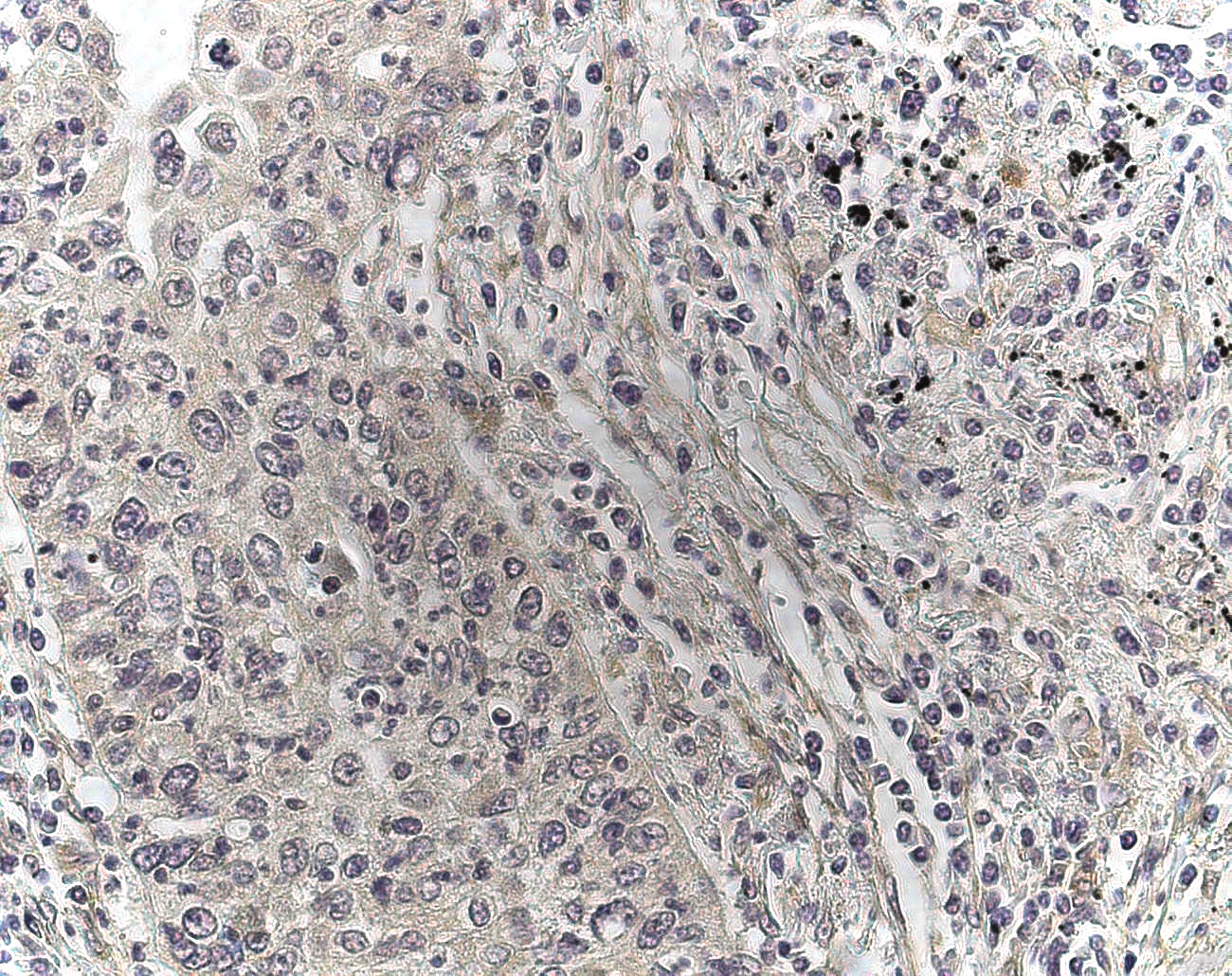

Supplement: S11 File — (ZIP) [file pone.0349359.s011.zip › Figure S4A PIK3CA left 40x.jpg]

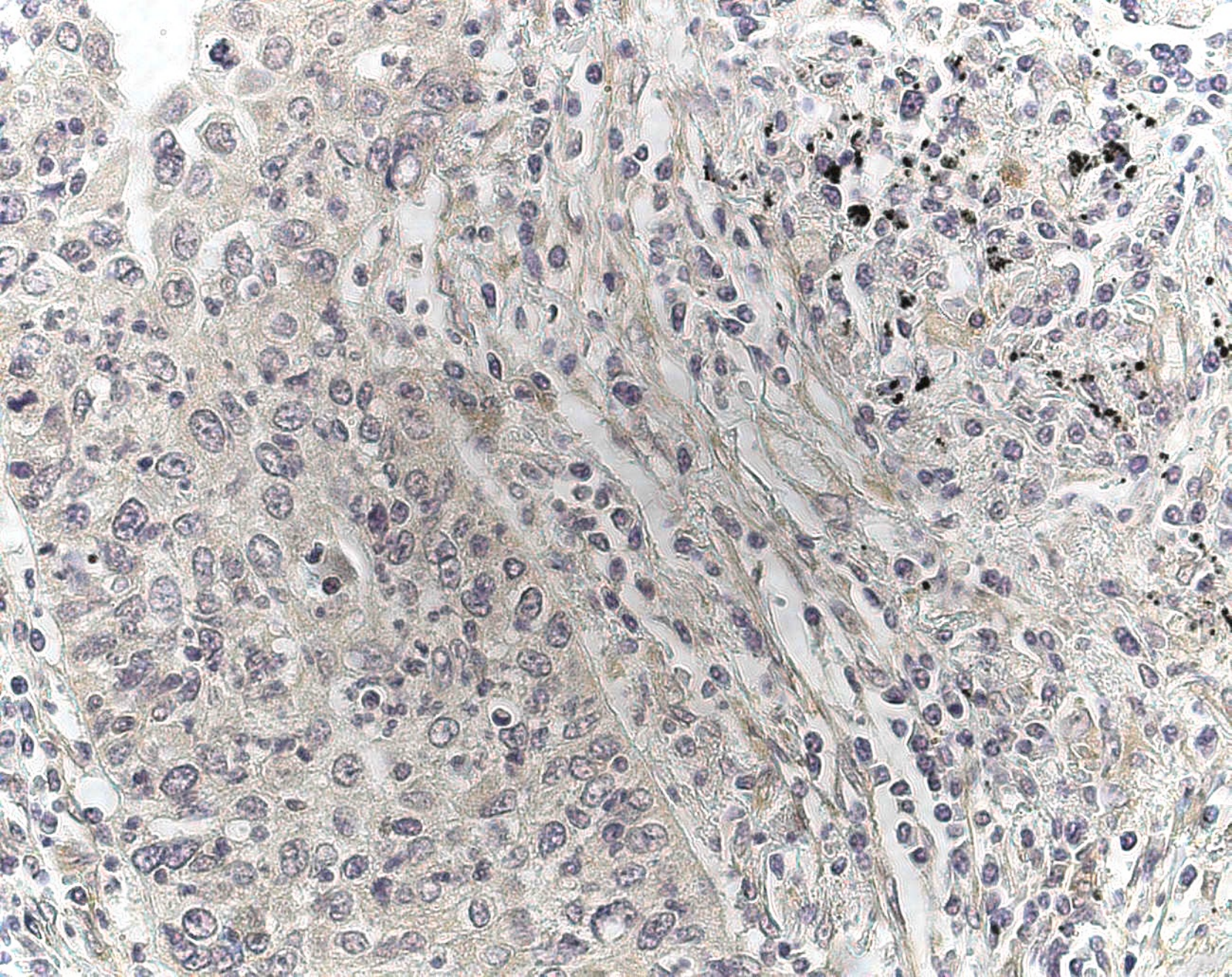

Supplement: S11 File — (ZIP) [file pone.0349359.s011.zip › Figure S4A PIK3CA left 40x.pdf]

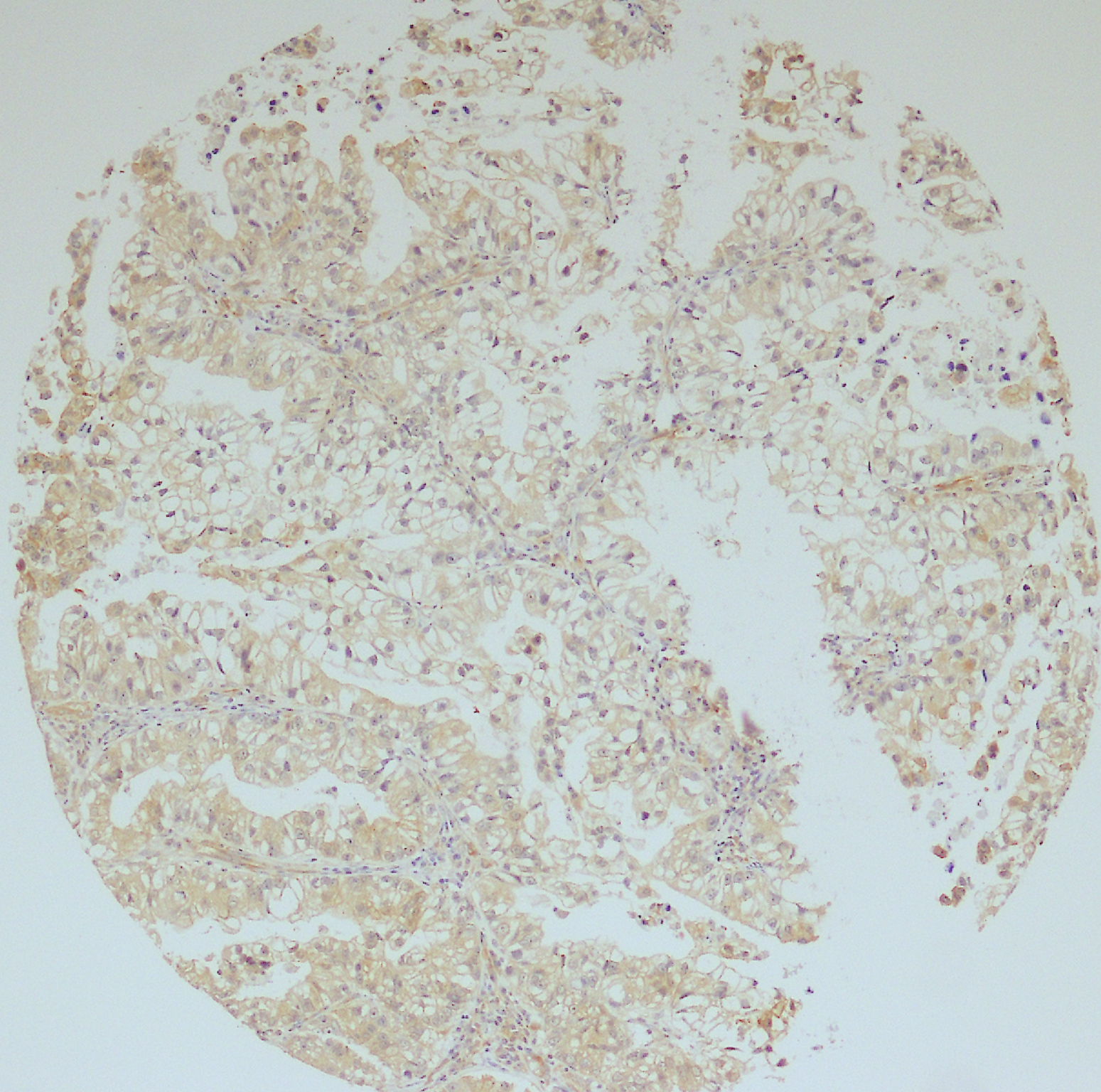

Supplement: S11 File — (ZIP) [file pone.0349359.s011.zip › Figure S4B PI3KCA (+) 10x.pdf]

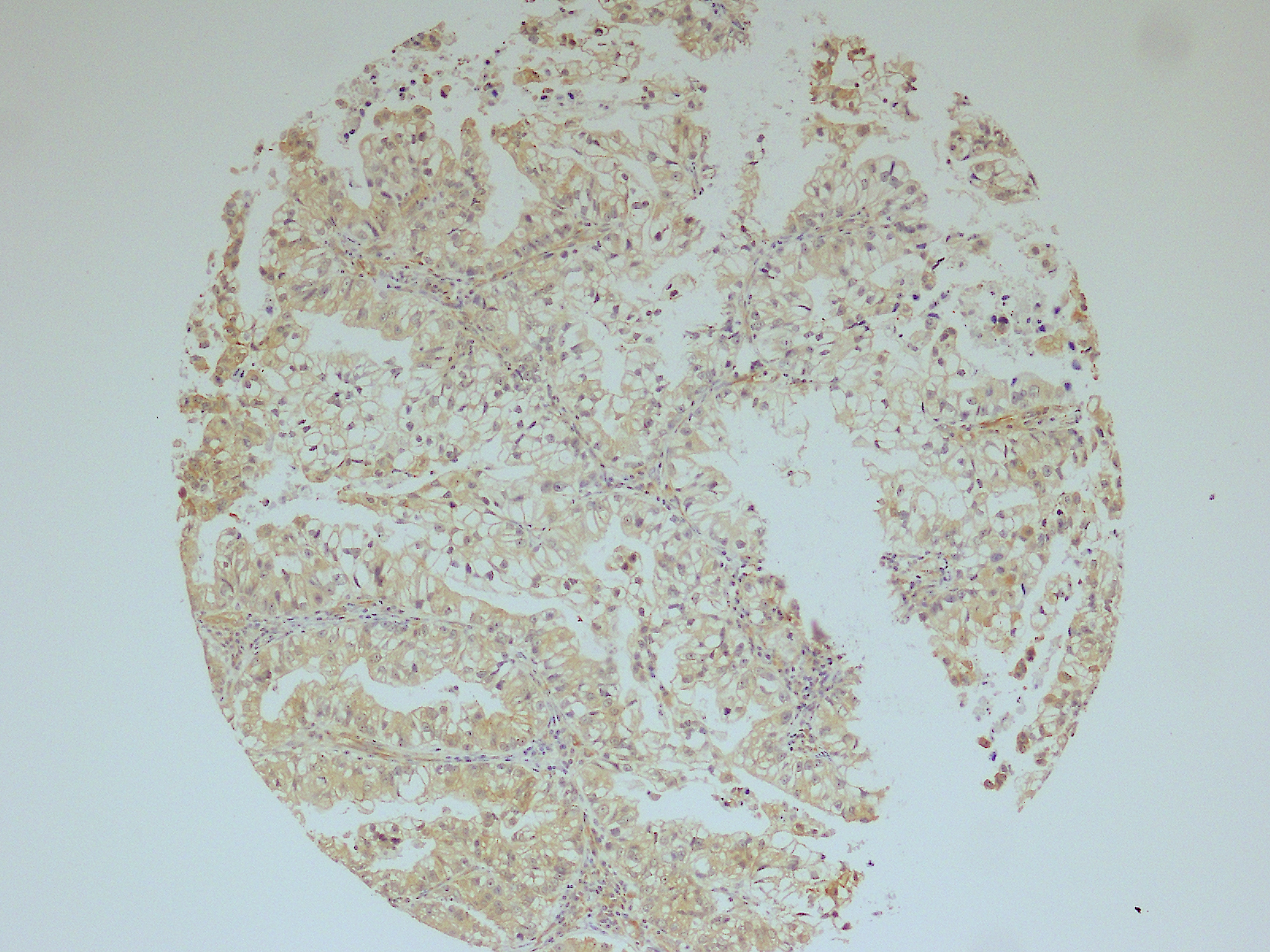

Supplement: S11 File — (ZIP) [file pone.0349359.s011.zip › Figure S4B PI3KCA (+) 10x.TIF]

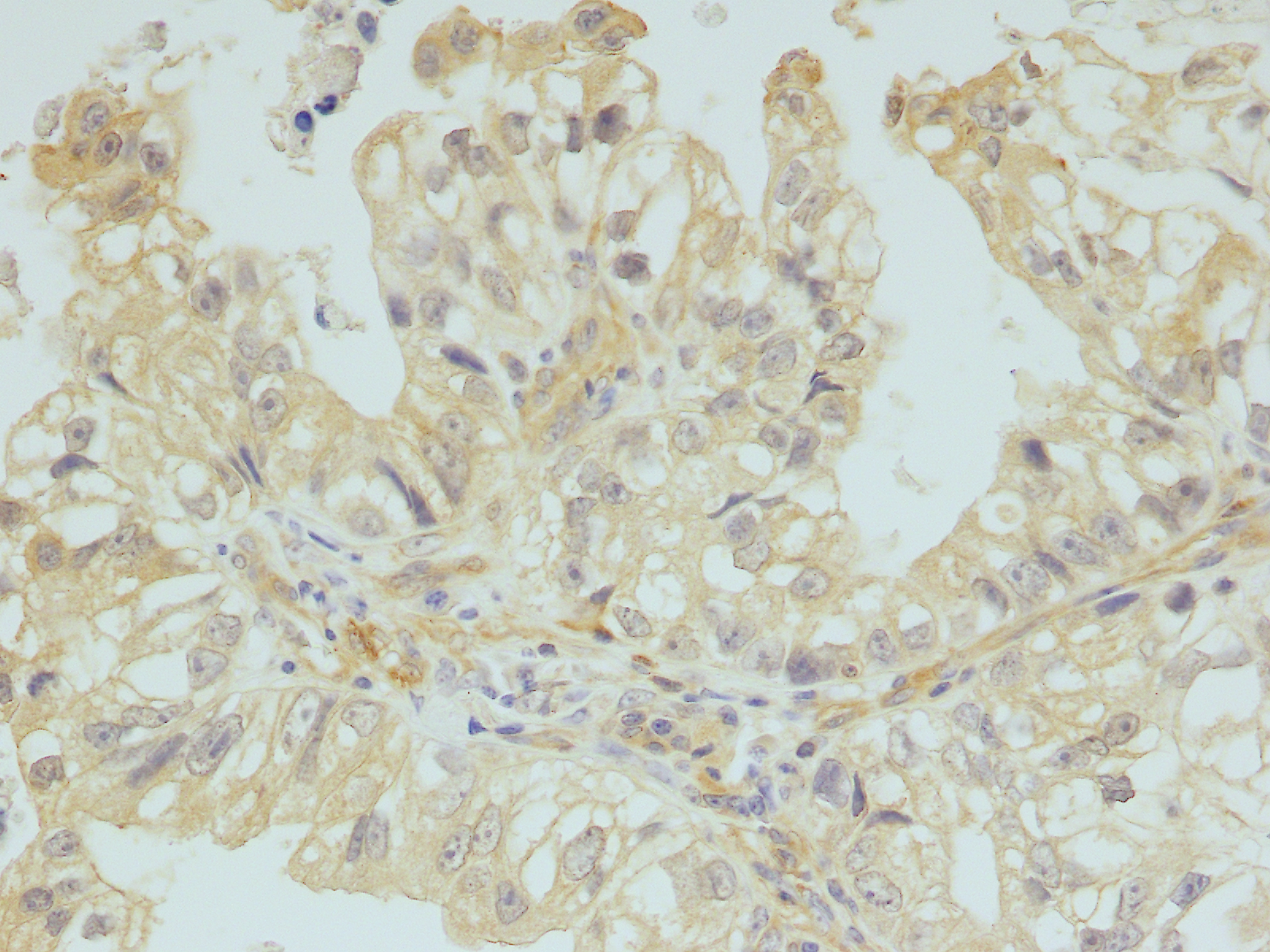

Supplement: S11 File — (ZIP) [file pone.0349359.s011.zip › Figure S4B PI3KCA (+) 40x.pdf]

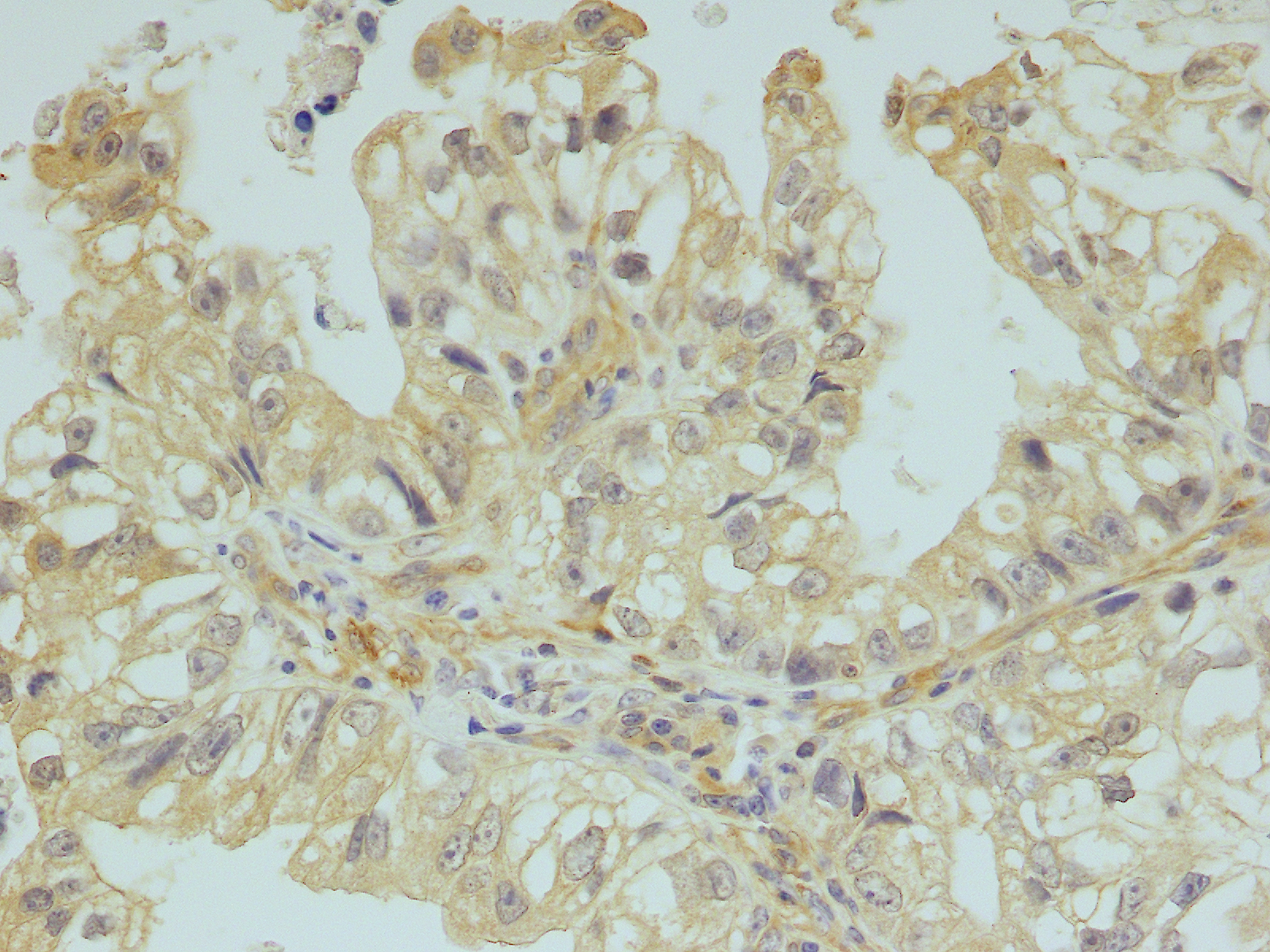

Supplement: S11 File — (ZIP) [file pone.0349359.s011.zip › Figure S4B PI3KCA (+) 40x.TIF]

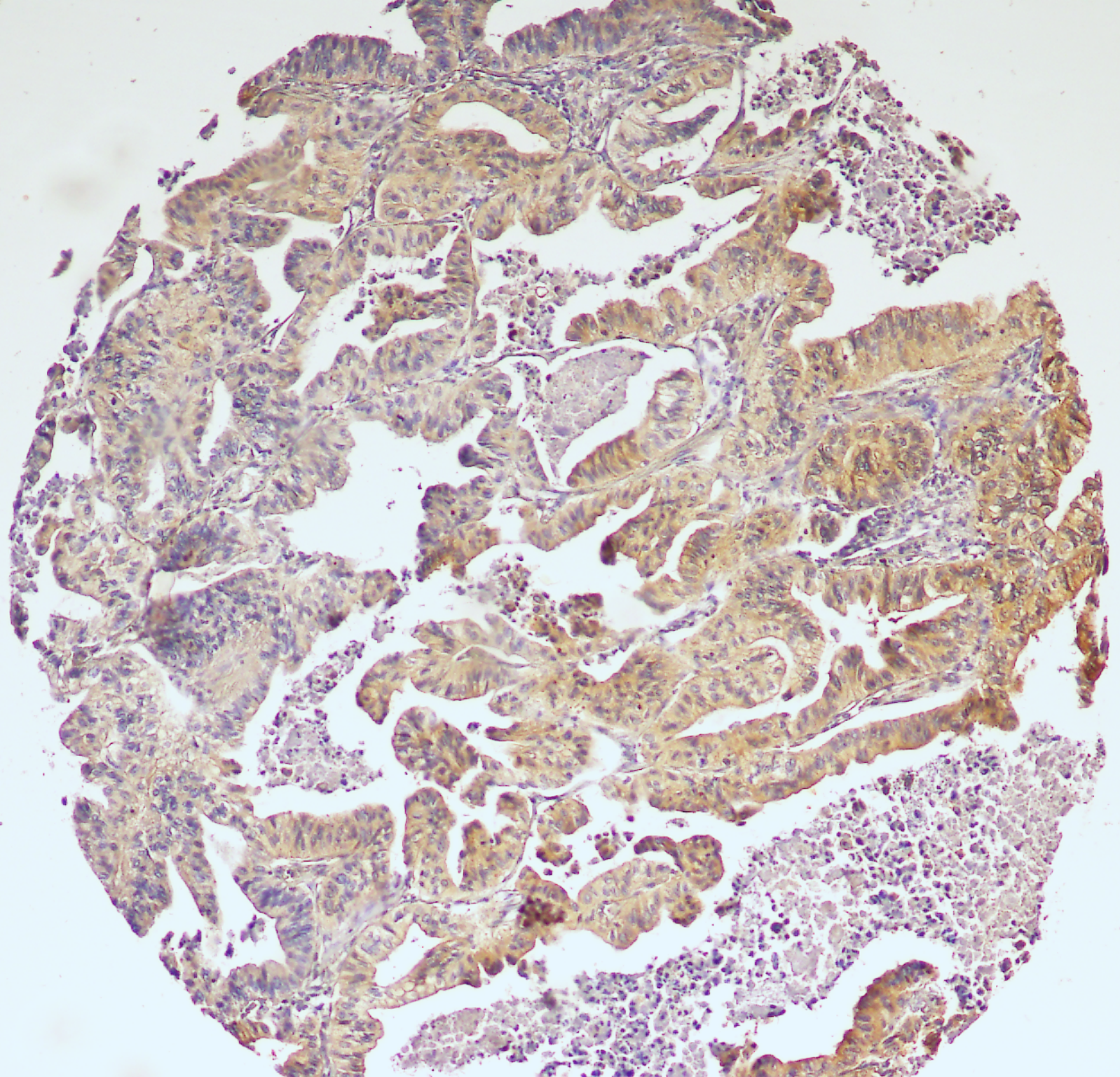

Supplement: S11 File — (ZIP) [file pone.0349359.s011.zip › Figure S4B PI3KCA (++) 10x.pdf]

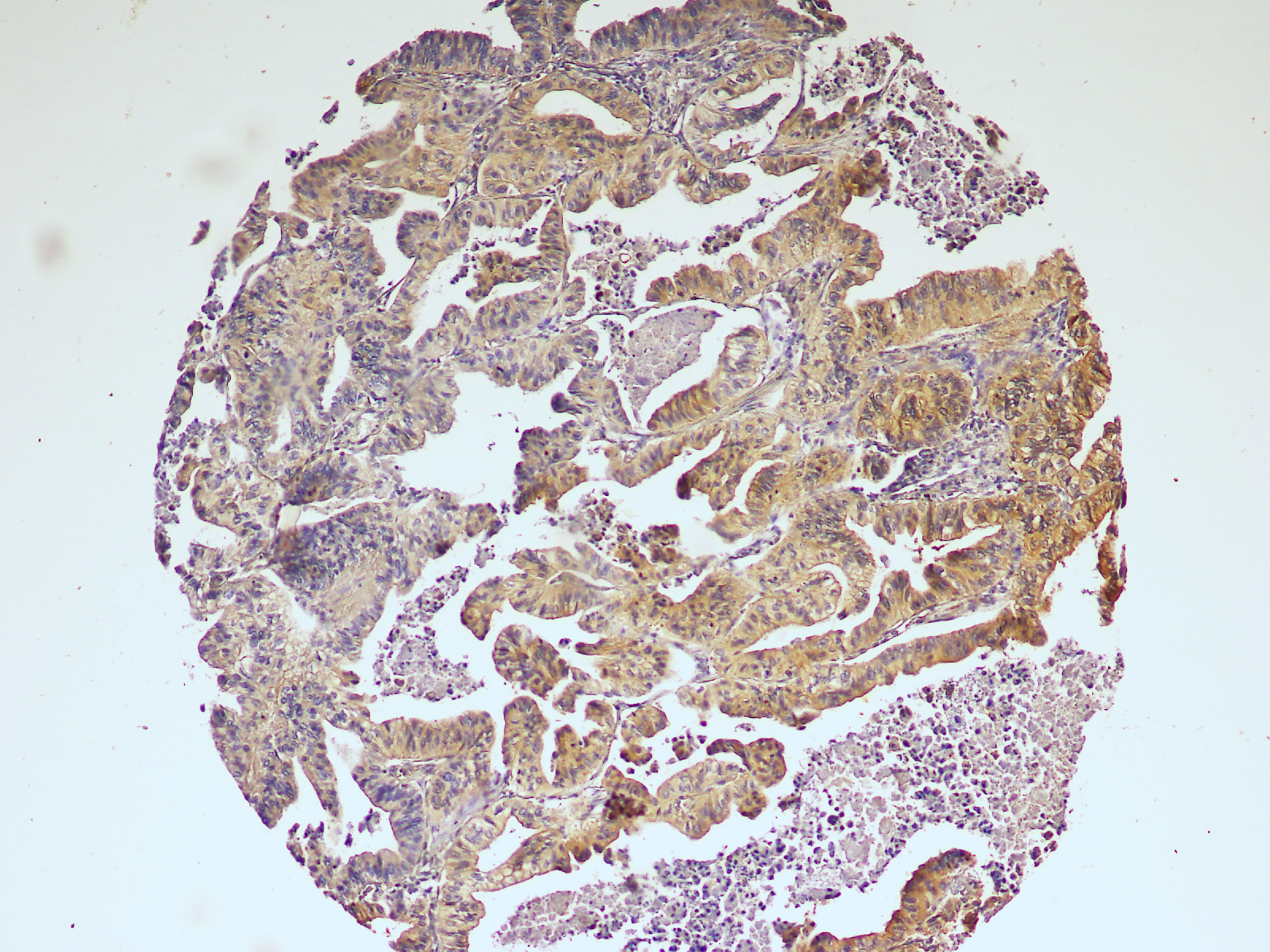

Supplement: S11 File — (ZIP) [file pone.0349359.s011.zip › Figure S4B PI3KCA (++) 10x.TIF]

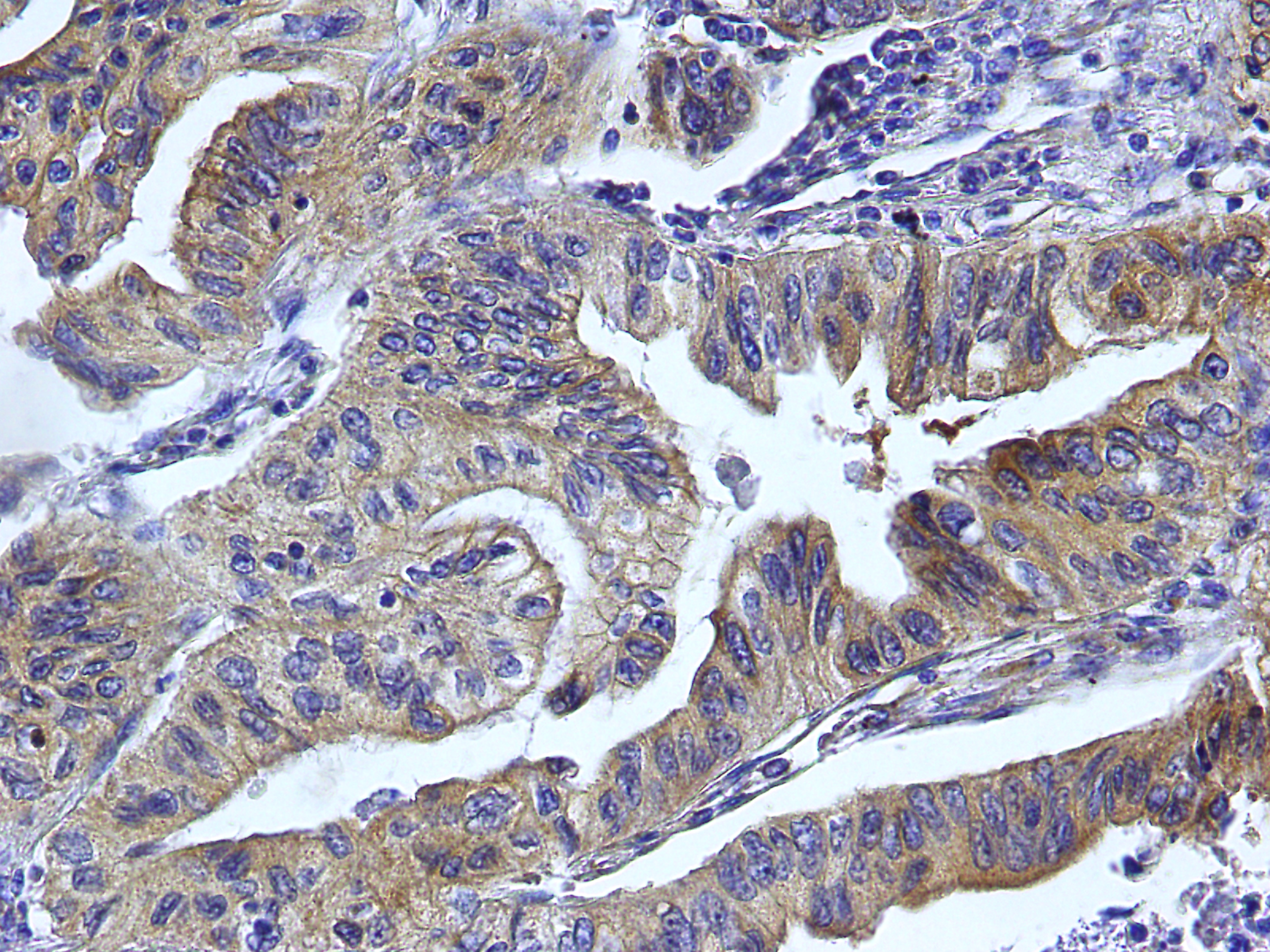

Supplement: S11 File — (ZIP) [file pone.0349359.s011.zip › Figure S4B PI3KCA (++) 40x.pdf]

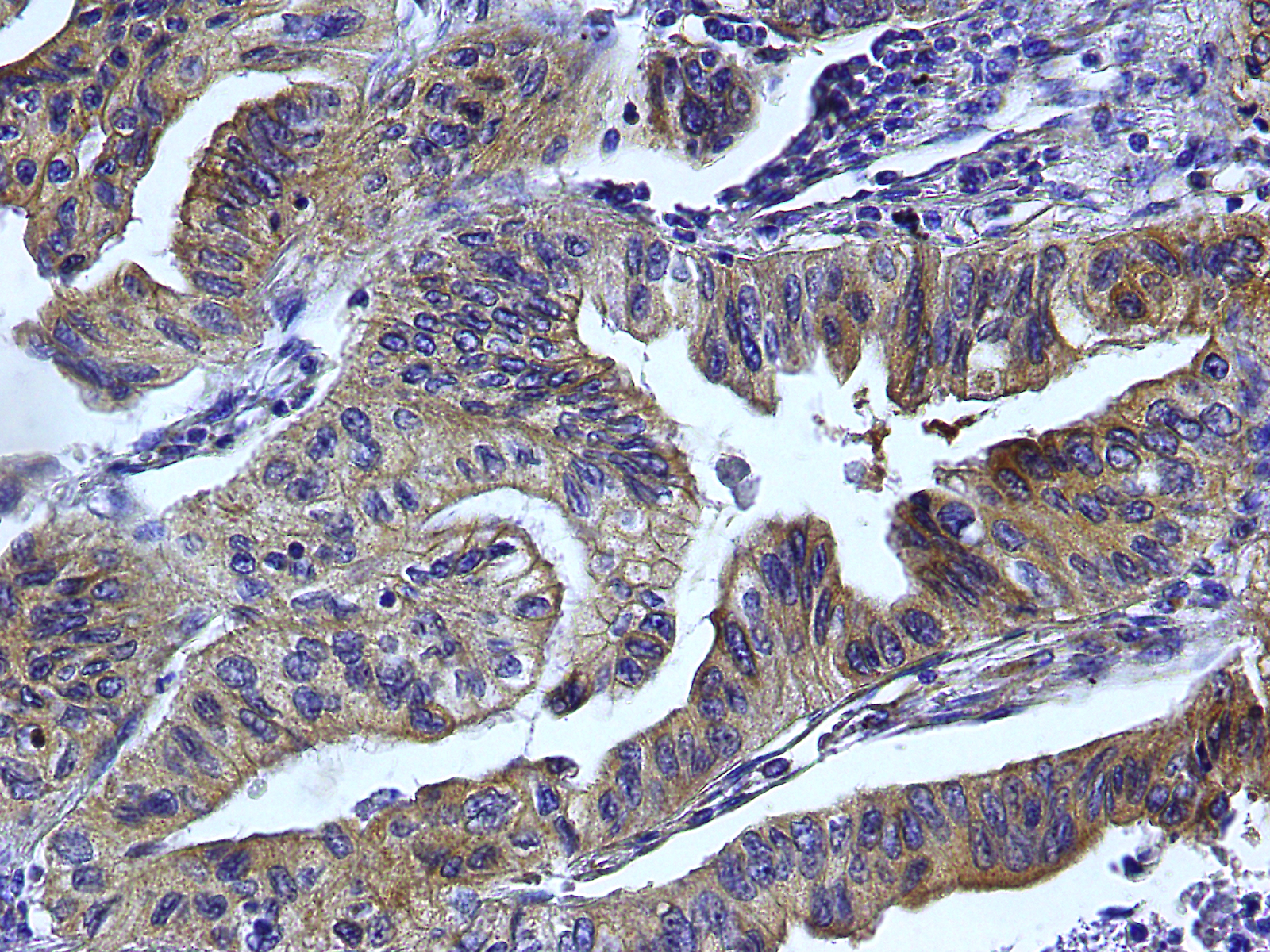

Supplement: S11 File — (ZIP) [file pone.0349359.s011.zip › Figure S4B PI3KCA (++) 40x.TIF]

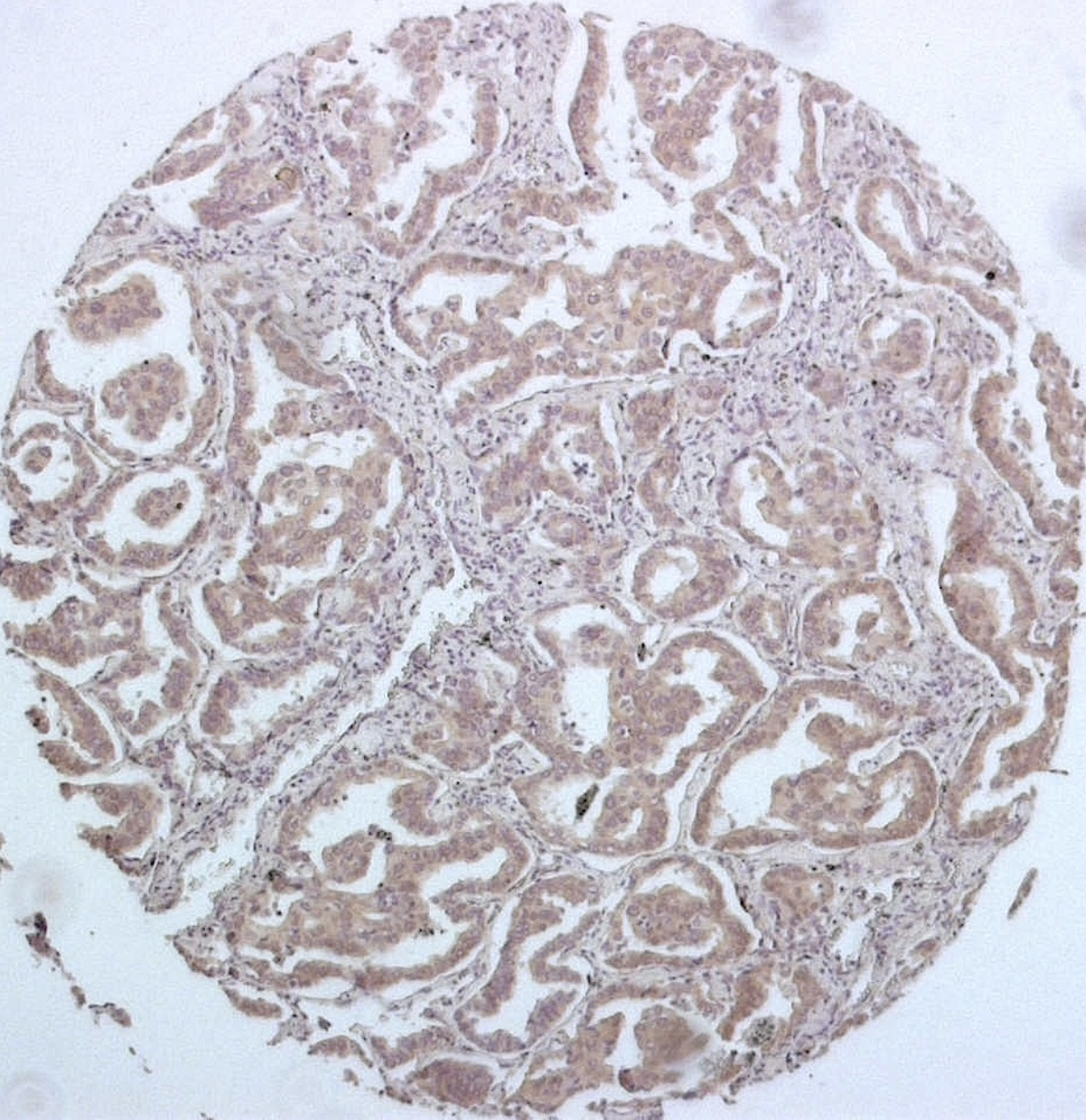

Supplement: S11 File — (ZIP) [file pone.0349359.s011.zip › Figure S4B PI3KCA right 10x.pdf]

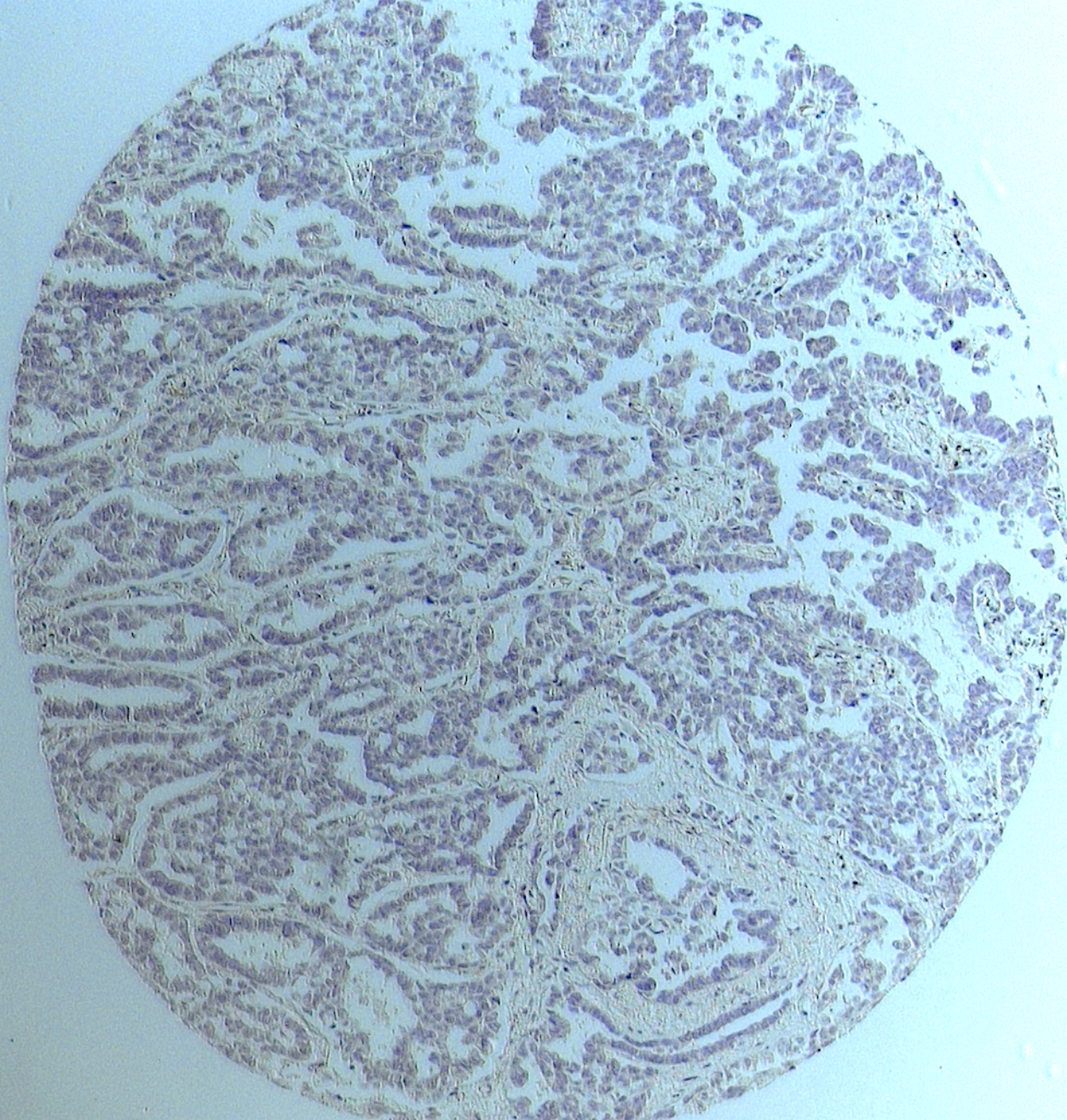

Supplement: S11 File — (ZIP) [file pone.0349359.s011.zip › Figure S4B PIK3CA left 10x.pdf]

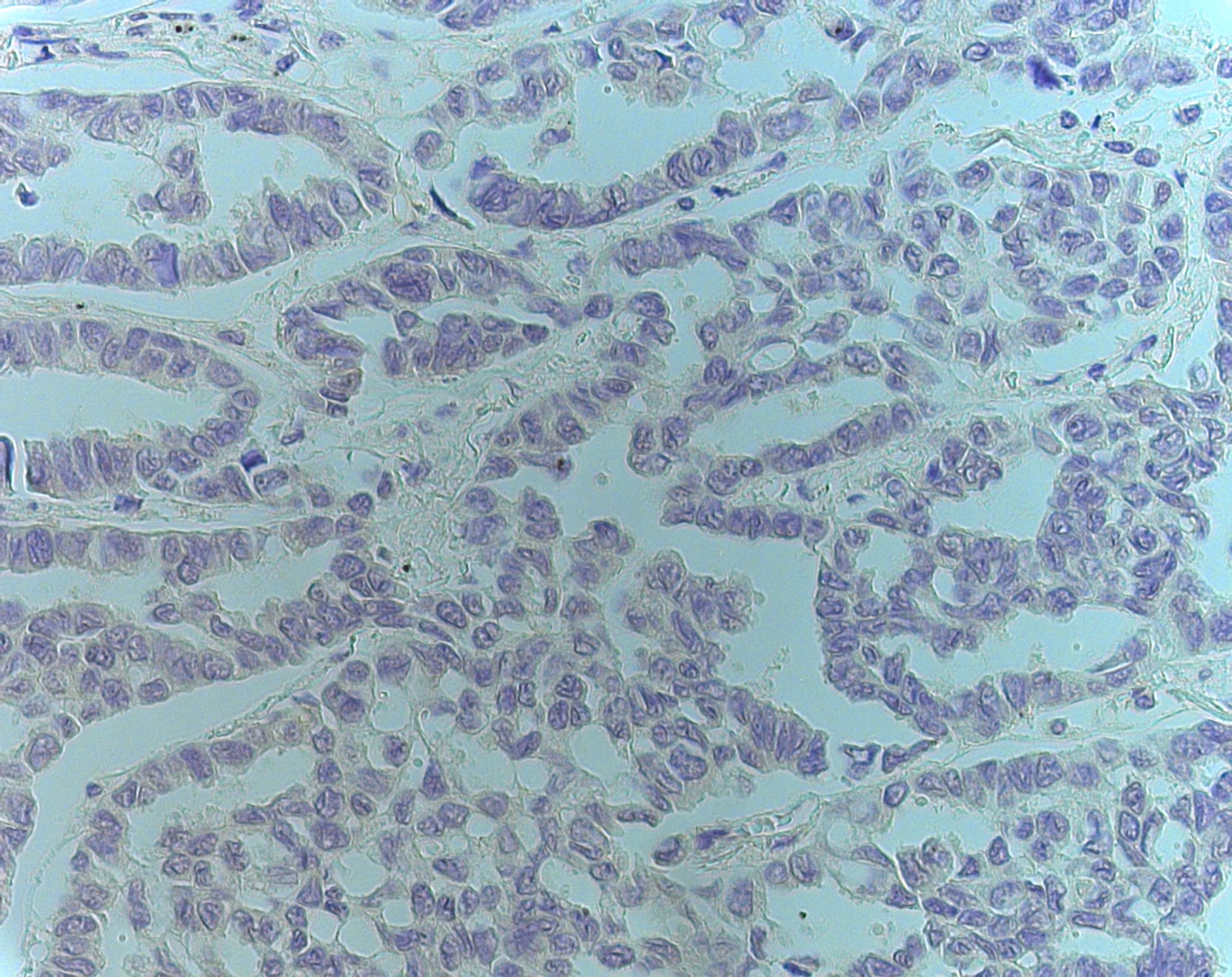

Supplement: S11 File — (ZIP) [file pone.0349359.s011.zip › Figure S4B PIK3CA left 40x.pdf]

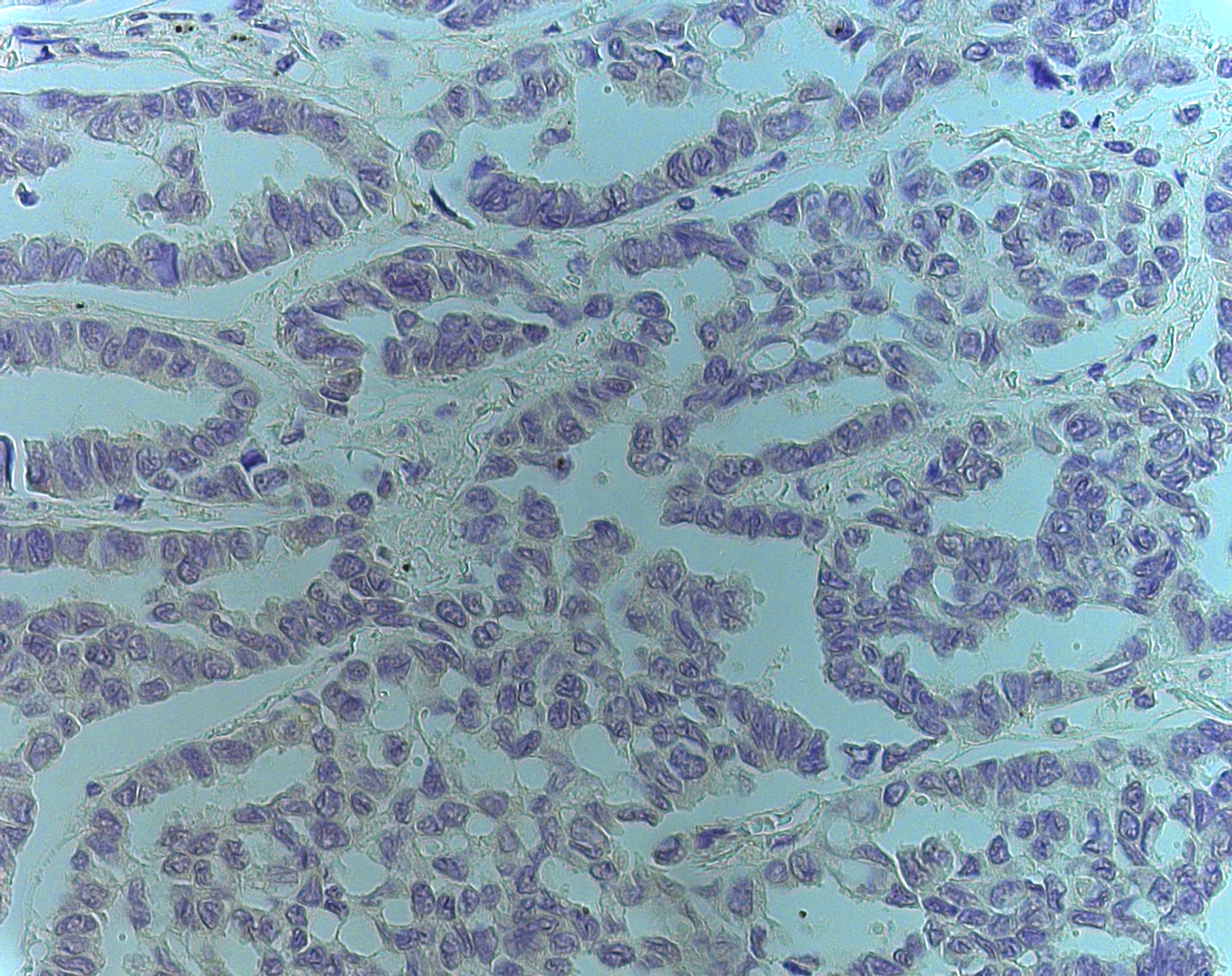

Supplement: S11 File — (ZIP) [file pone.0349359.s011.zip › Figure S4B PIK3CA left 40x.tif]

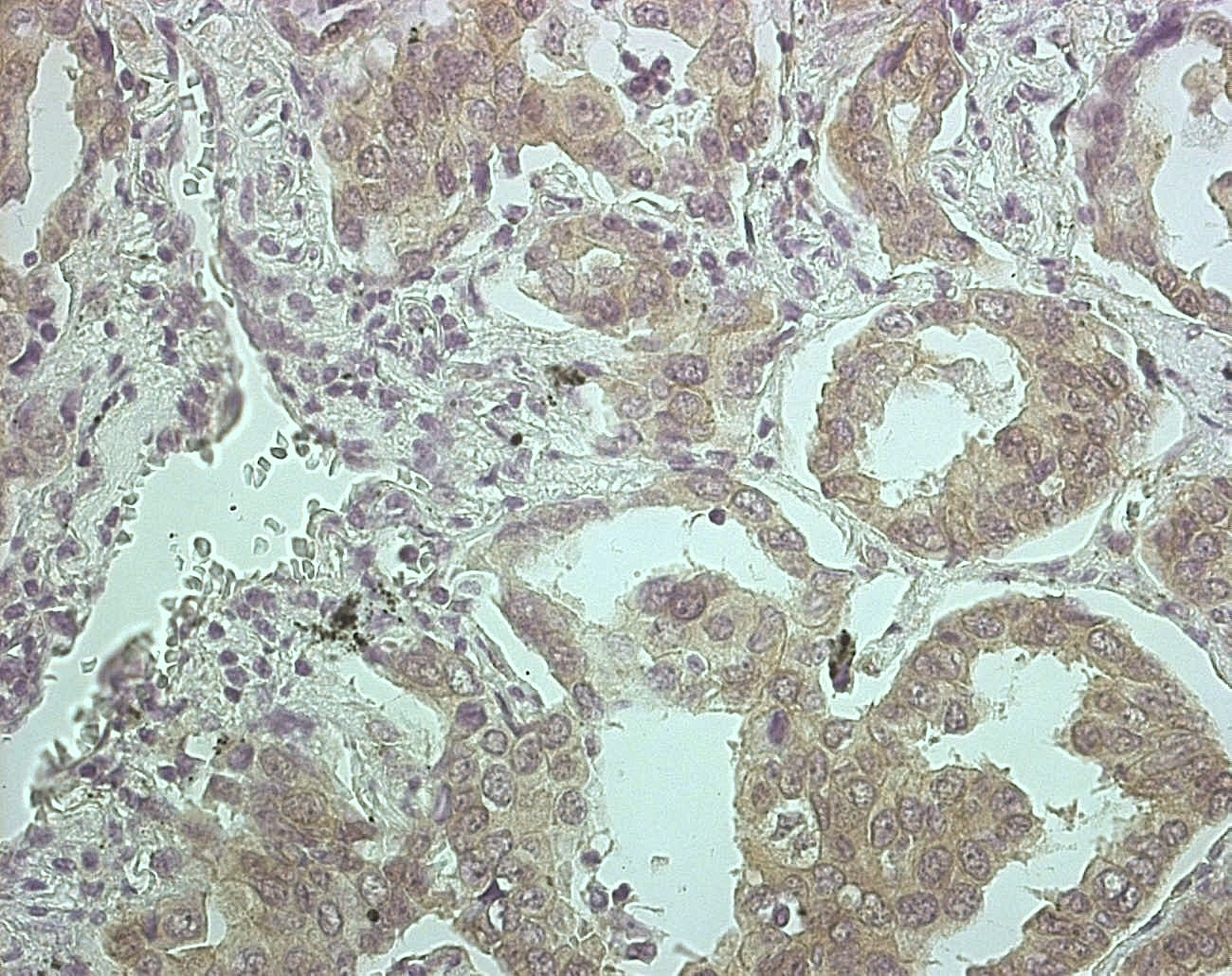

Supplement: S11 File — (ZIP) [file pone.0349359.s011.zip › Figure S4B PIK3CA right 40x.jpg]

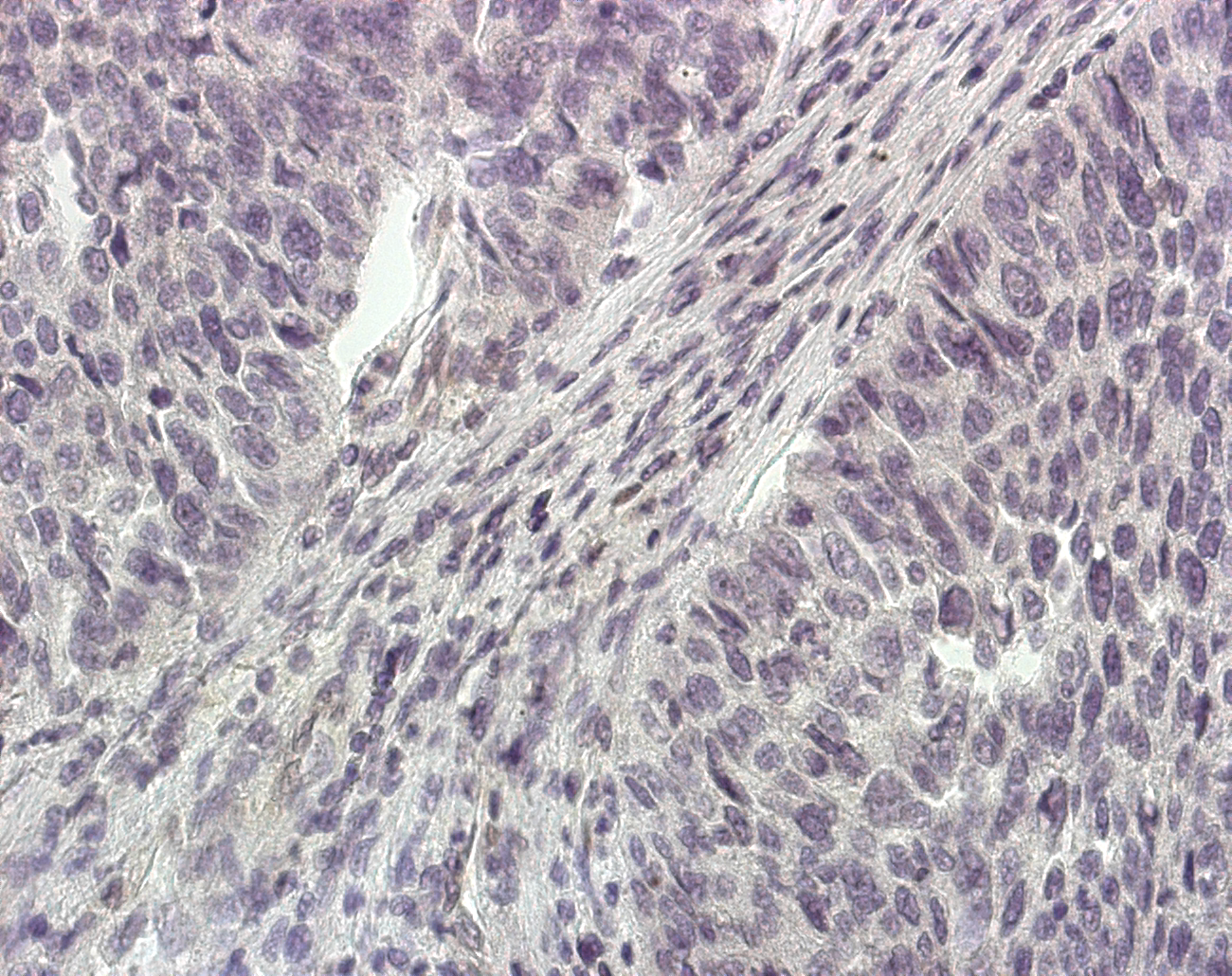

Supplement: S12 File — (ZIP) [file pone.0349359.s012.zip › FigureS5B PTEN SCC left 40x.tif]

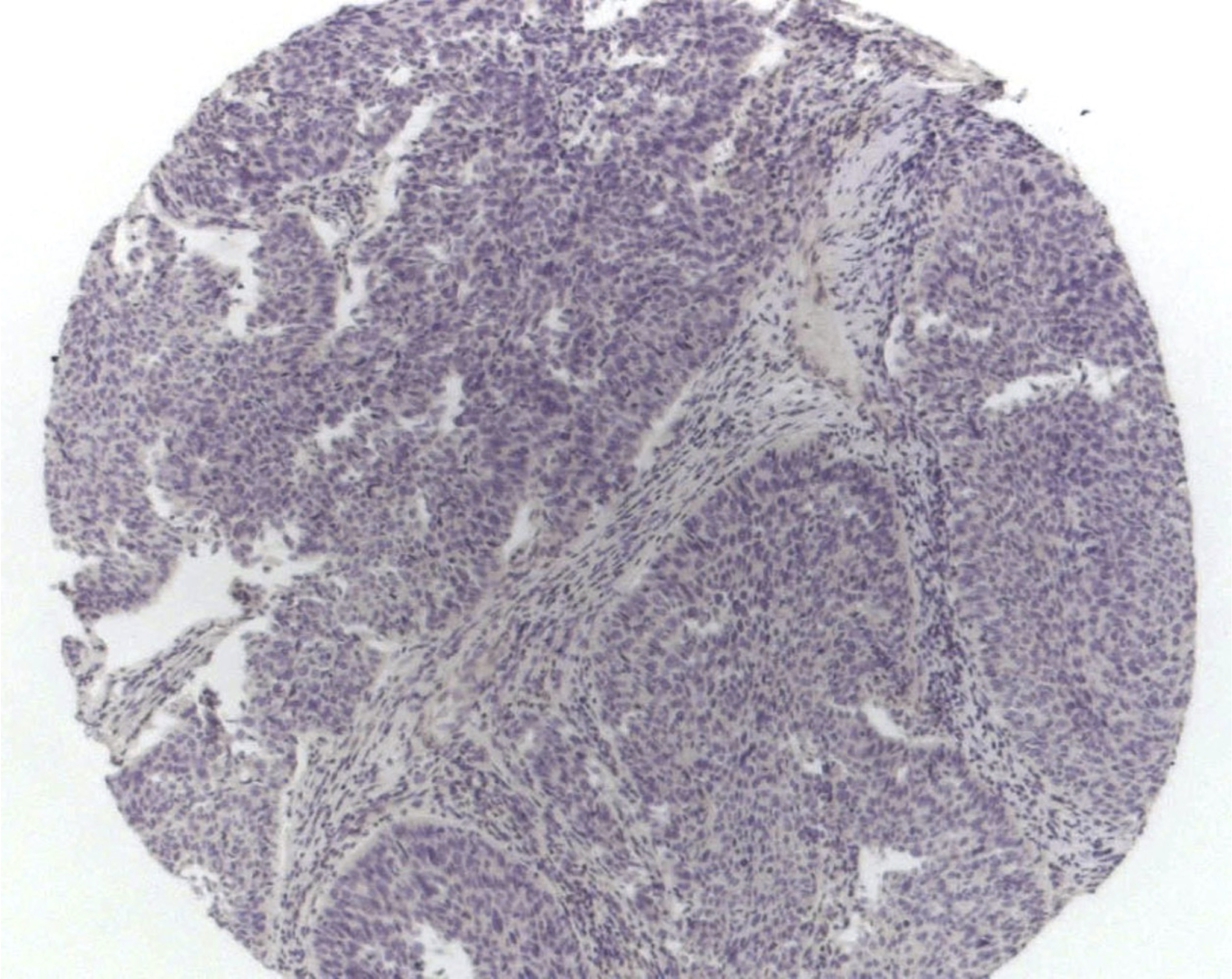

Supplement: S12 File — (ZIP) [file pone.0349359.s012.zip › Figure S5B PTEN SCC left 10x.jpg]

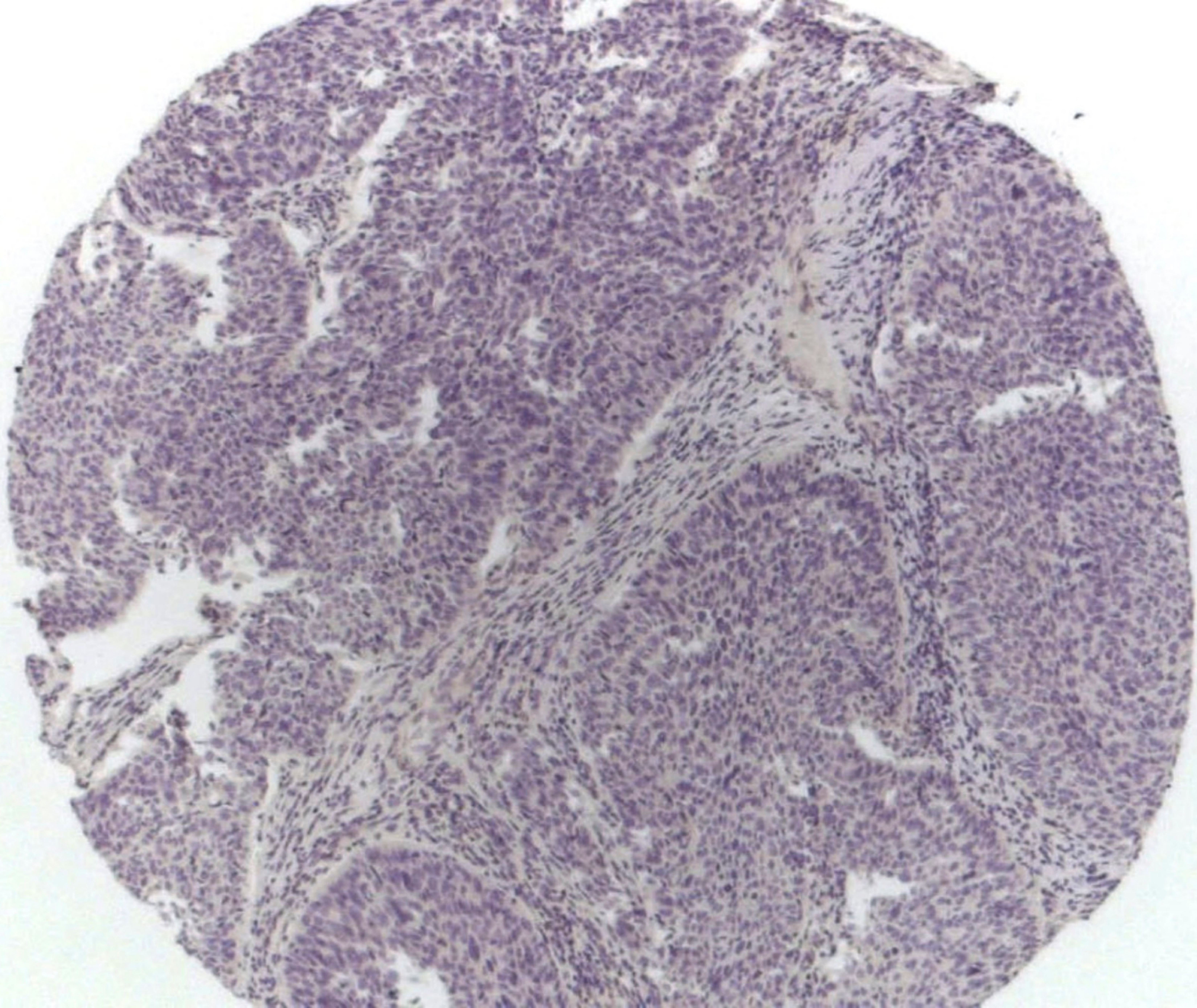

Supplement: S12 File — (ZIP) [file pone.0349359.s012.zip › Figure S5B PTEN SCC left 10x.pdf]

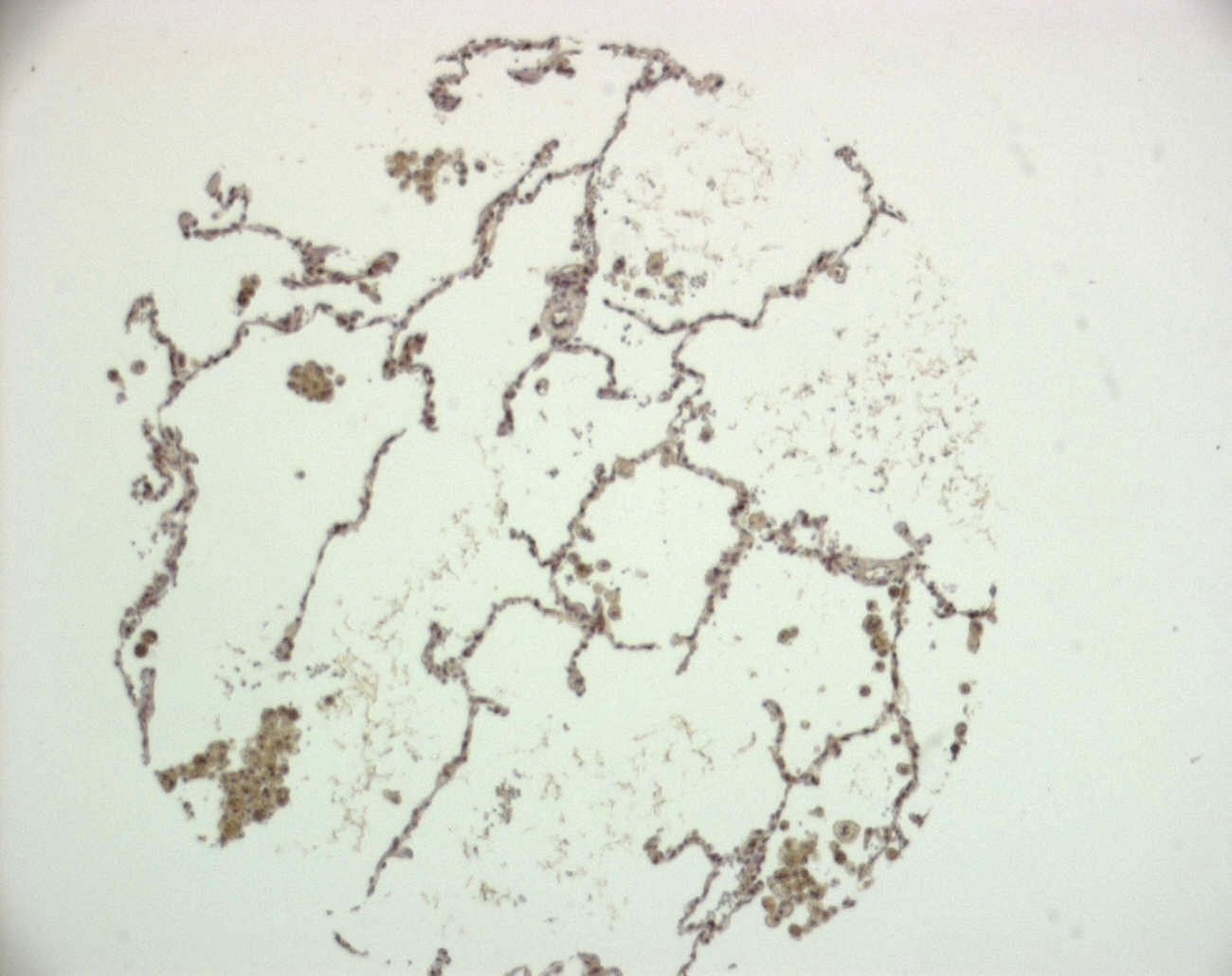

Supplement: S12 File — (ZIP) [file pone.0349359.s012.zip › FigureS5A PTEN normal 10x.jpg]

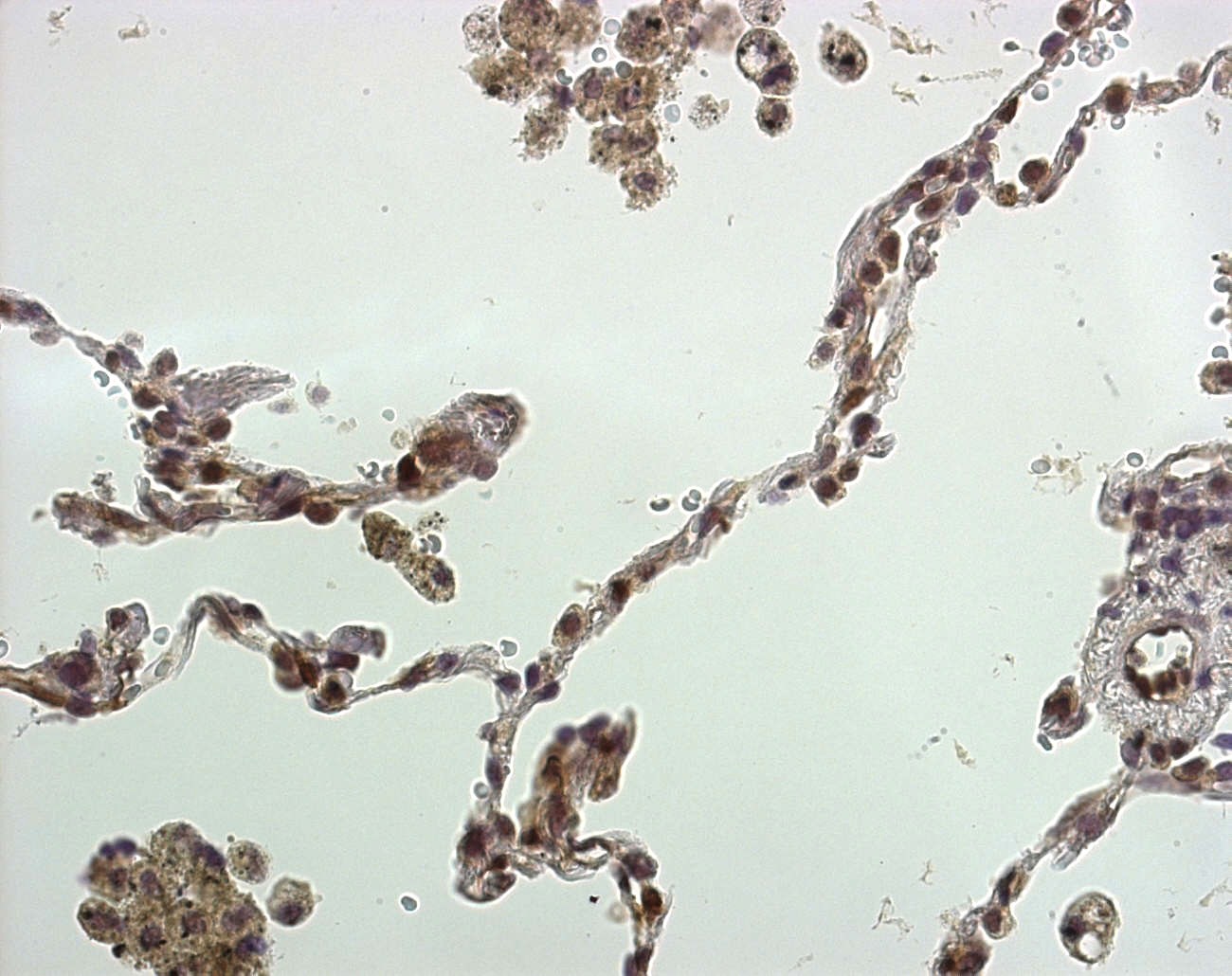

Supplement: S12 File — (ZIP) [file pone.0349359.s012.zip › FigureS5A PTEN normal 40x.jpg]

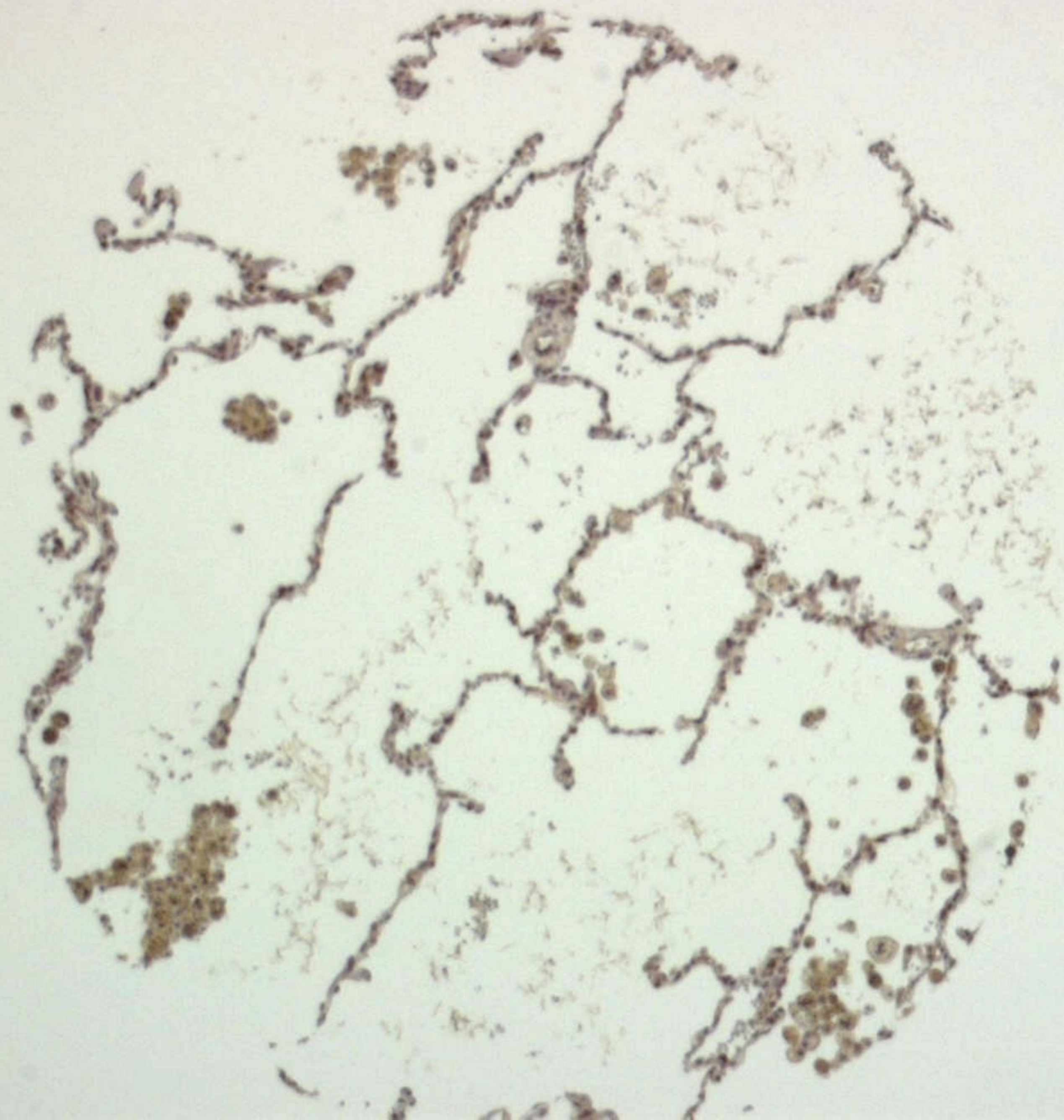

Supplement: S12 File — (ZIP) [file pone.0349359.s012.zip › FigureS5A PTEN normal 10x.pdf]

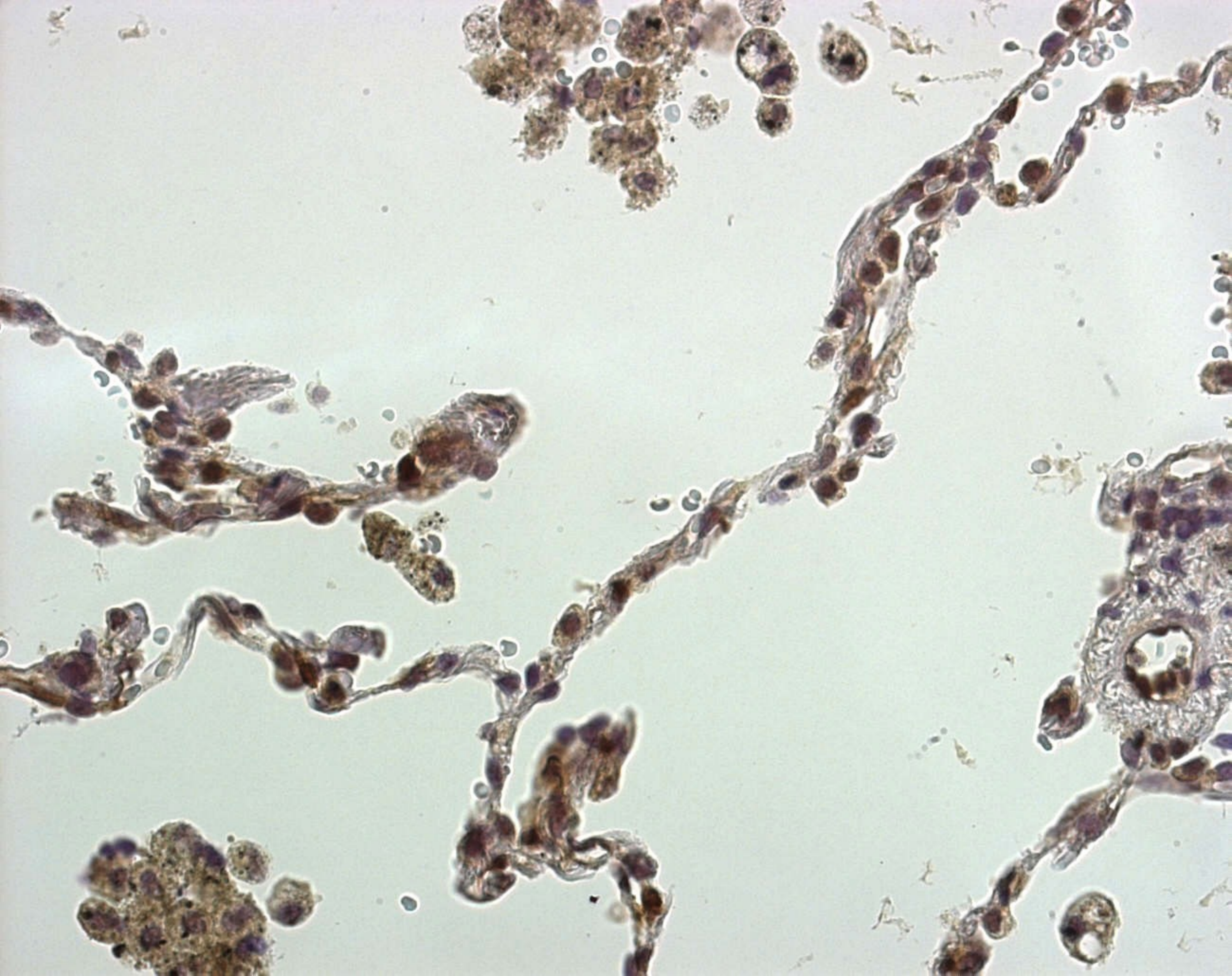

Supplement: S12 File — (ZIP) [file pone.0349359.s012.zip › FigureS5A PTEN normal 40x.pdf]

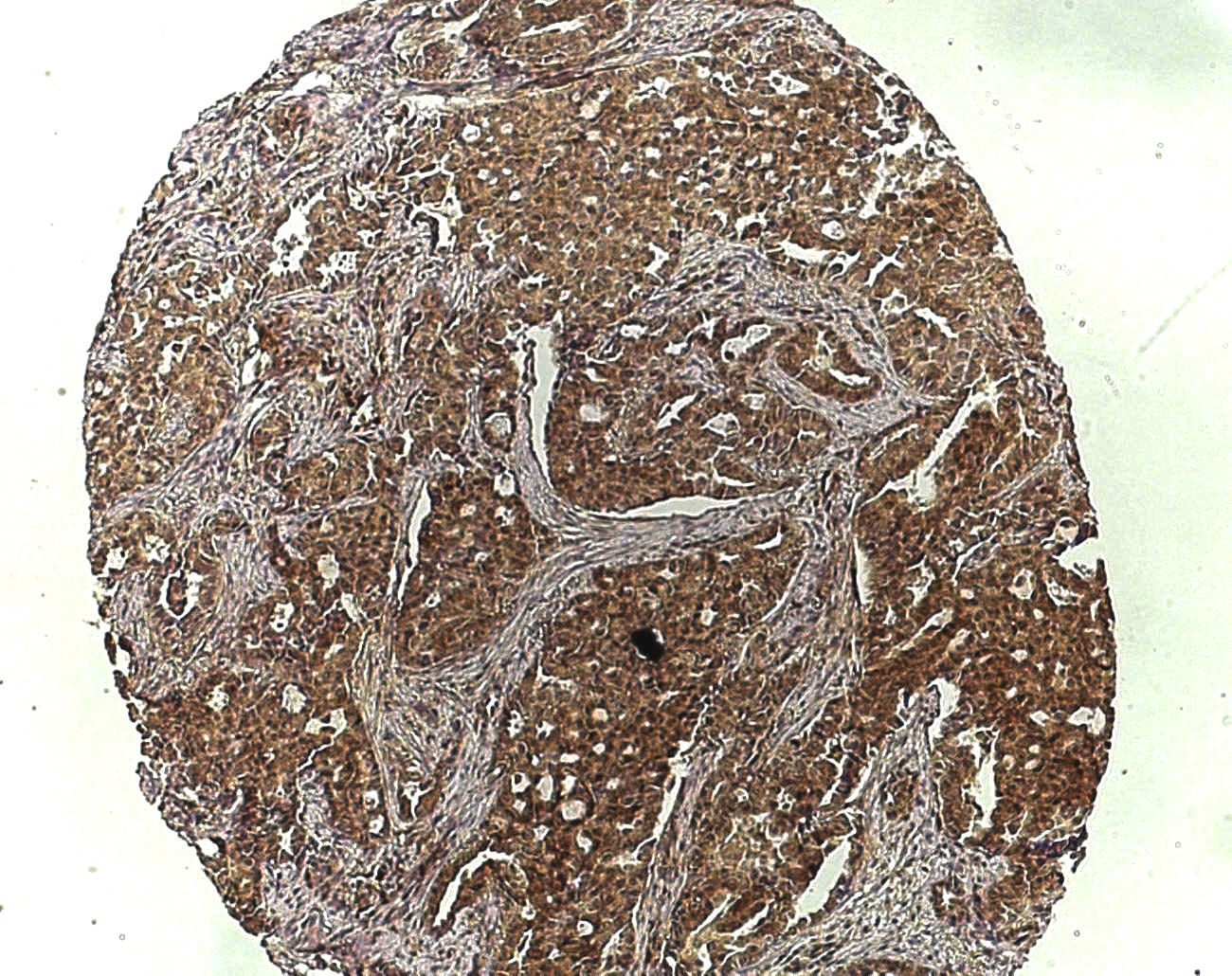

Supplement: S12 File — (ZIP) [file pone.0349359.s012.zip › FigureS5B PTEN ADC right 10x.jpg]

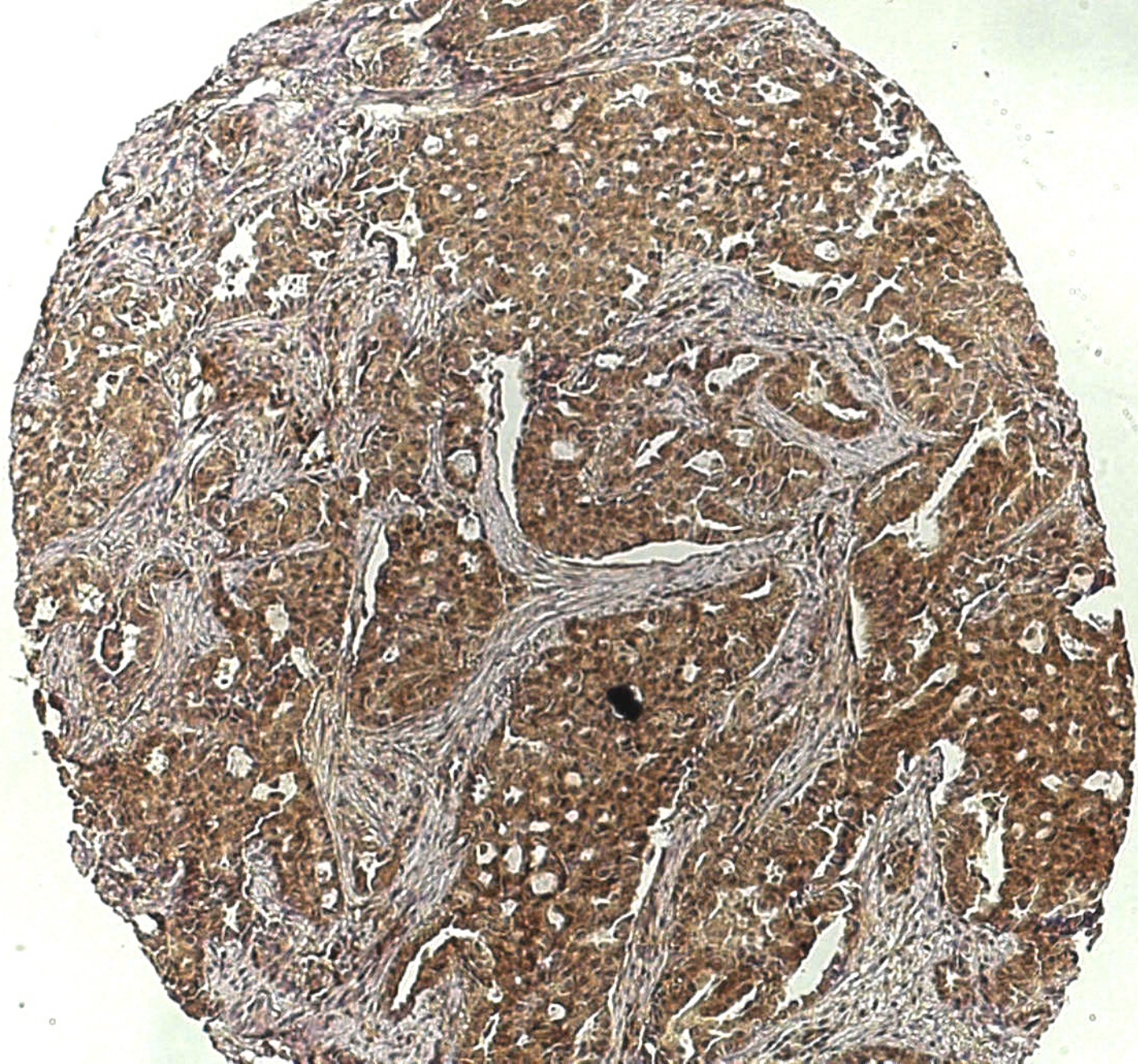

Supplement: S12 File — (ZIP) [file pone.0349359.s012.zip › FigureS5B PTEN ADC right 10x.pdf]

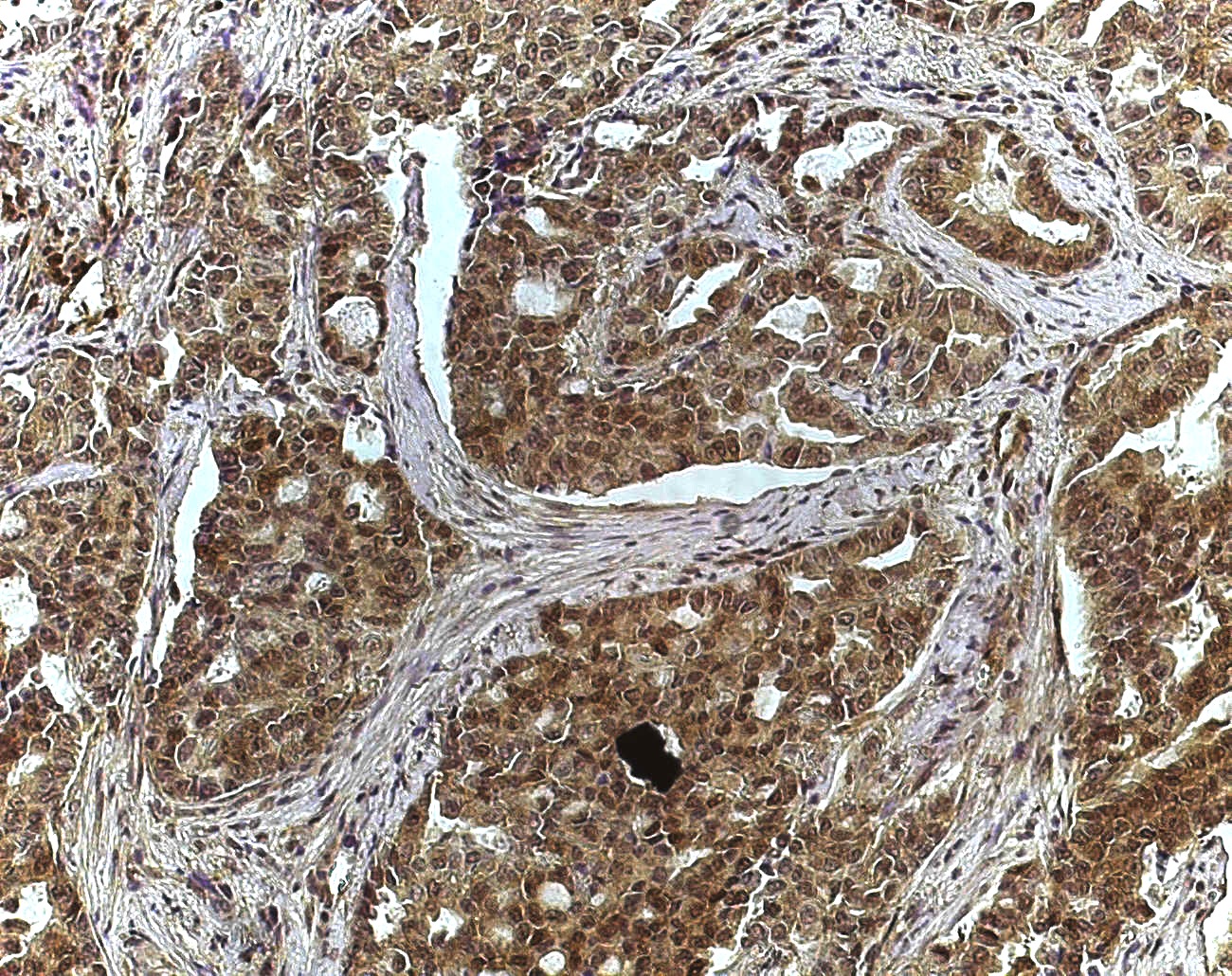

Supplement: S12 File — (ZIP) [file pone.0349359.s012.zip › FigureS5B PTEN ADC right 40x.jpg]

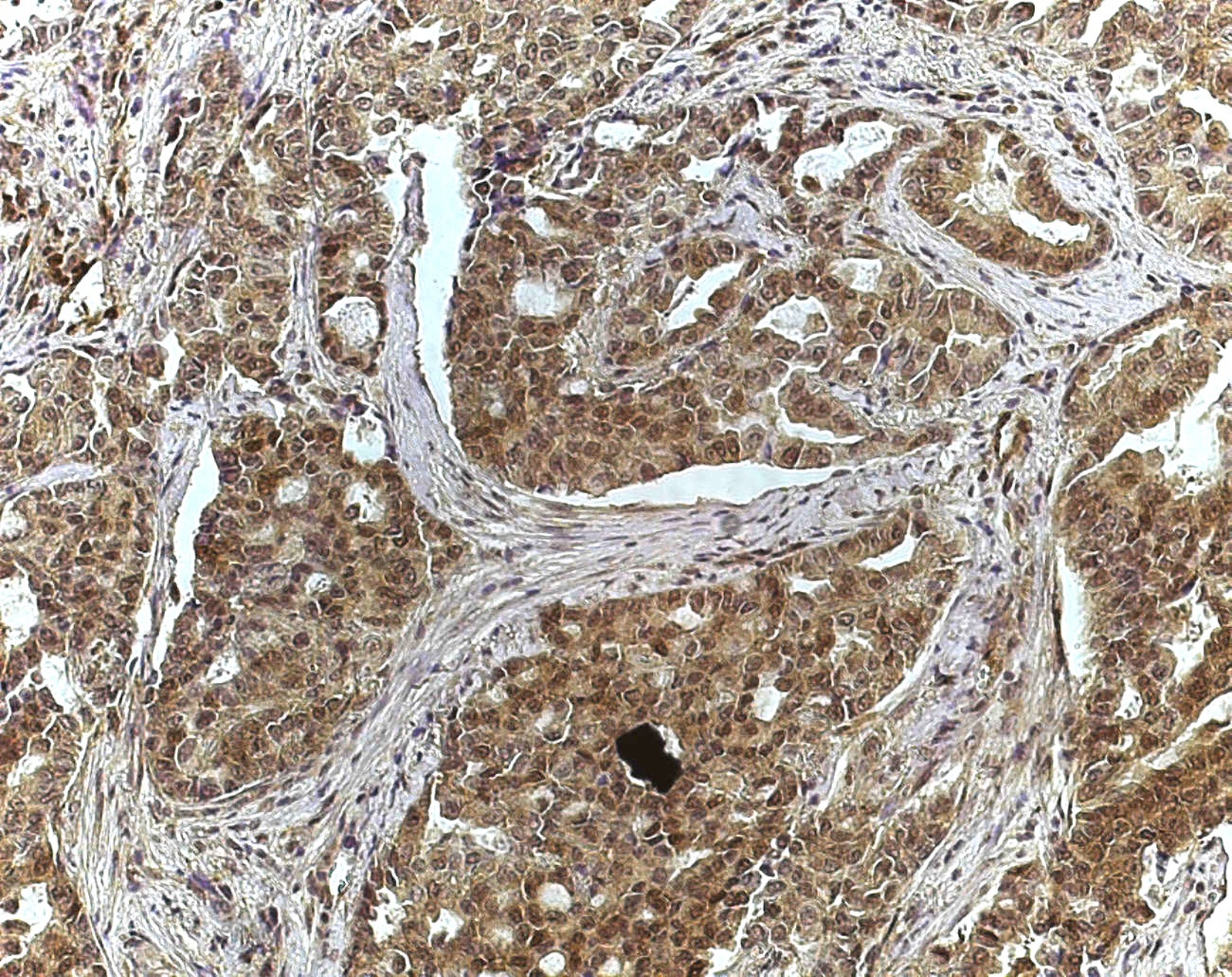

Supplement: S12 File — (ZIP) [file pone.0349359.s012.zip › FigureS5B PTEN ADC right 40x.pdf]

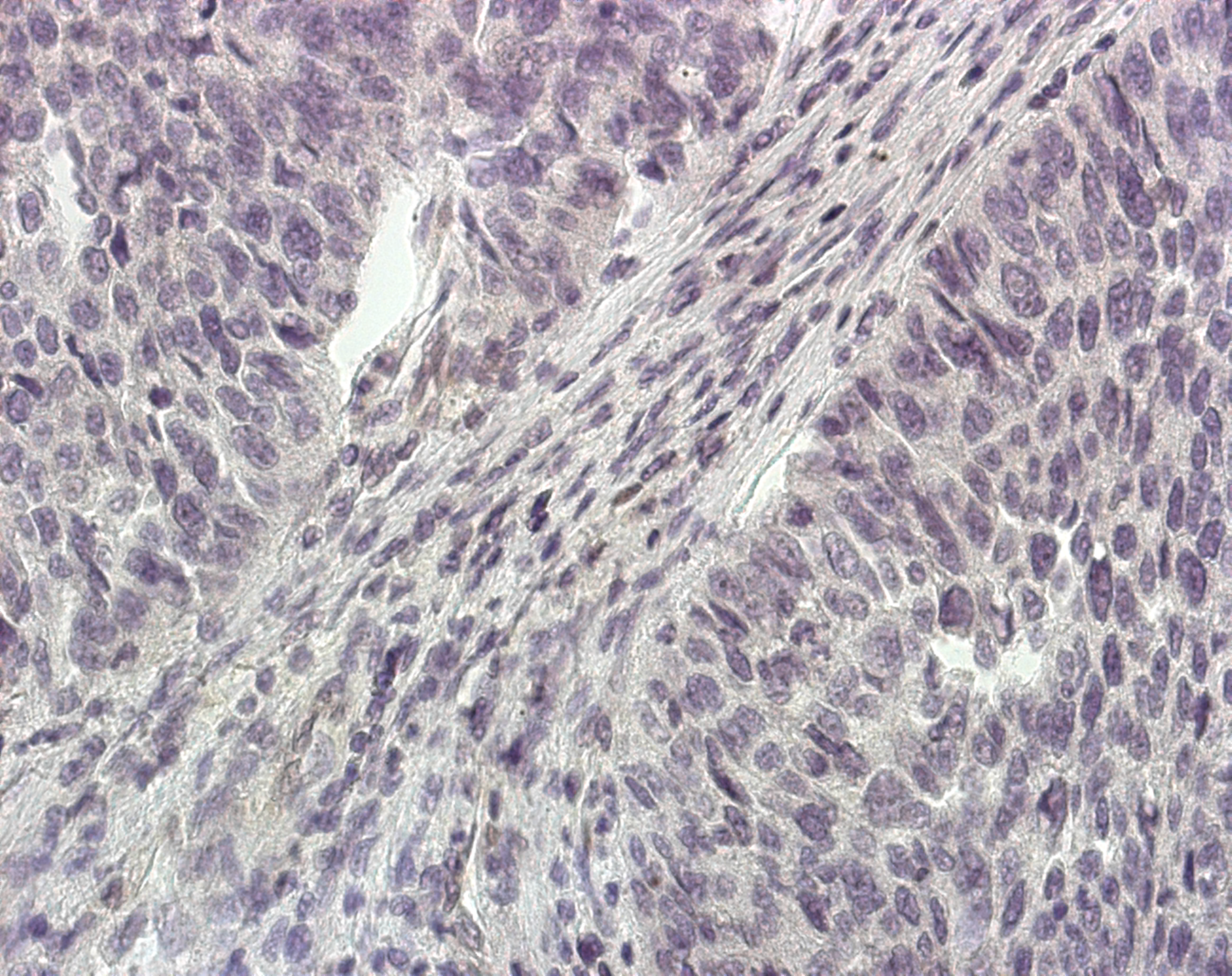

Supplement: S12 File — (ZIP) [file pone.0349359.s012.zip › FigureS5B PTEN SCC left 40x.pdf]
