# Supplementary material for: Expression of Concern: Signaling Networks Associated with AKT Activation in Non-Small Cell Lung Cancer (NSCLC): New Insights on the Role of Phosphatydil-Inositol-3 kinase
Source: PLoS One. 2026 May 14;21(5):e0349359. doi: 10.1371/journal.pone.0349359 (PMC13175380; doi:10.1371/journal.pone.0349359)
Supplement: S11 File — (ZIP) [file pone.0349359.s011.zip › Figure S4 list of contents.docx]

Figure S4A PI3KCA right 40x.jpg

Figure S4A PI3KCA right 40x.pdf

Figure S4A PI3KCA(++) 10x.TIF

Figure S4A PIK3CA (+) 40x.pdf

Figure S4A PIK3CA (++) 10x.pdf

Figure S4A PIK3CA (+) 10x.pdf

Figure S4A PIK3CA (+) 10x.TIF

Figure S4A PIK3CA (+) 40x.TIF

Figure S4A PIK3CA (++) 40x.pdf

Figure S4A PIK3CA (++) 40x.TIF

Figure S4A PIK3CA left 10x.pdf

Figure S4A PIK3CA left 10x.jpg

Figure S4A PIK3CA left 40x.pdf

Figure S4A PIK3CA left 40x.jpg

Figure S4A PIK3CA right 10x.jpg

Figure S4A PIK3CA right 10x.pdf

Figure S4B PI3KCA (+) 10x.pdf

Figure S4B PI3KCA (+) 10x.TIF

Figure S4B PI3KCA (+) 40x.pdf

Figure S4B PI3KCA (+) 40x.TIF

Figure S4B PI3KCA (++) 10x.pdf

Figure S4B PI3KCA (++) 10x.TIF

Figure S4B PI3KCA (++) 40x.pdf

Figure S4B PI3KCA (++) 40x.TIF

Figure S4B PI3KCA right 10x.pdf

Figure S4B PIK3CA left 10x.pdf

Figure S4B PIK3CA left 10x.tif

Figure S4B PIK3CA left 40x.pdf

Figure S4B PIK3CA left 40x.tif

Figure S4B PIK3CA right 10x.jpg

Figure S4B PIK3CA right 40x.pdf

Figure S4B PIK3CA right 40x.jpg

SUPPORTING FIGURES FOR SUBMISSION.ppt
