# Supplementary material for: Expression of Concern: Signaling Networks Associated with AKT Activation in Non-Small Cell Lung Cancer (NSCLC): New Insights on the Role of Phosphatydil-Inositol-3 kinase
Source: PLoS One. 2026 May 14;21(5):e0349359. doi: 10.1371/journal.pone.0349359 (PMC13175380; doi:10.1371/journal.pone.0349359)
Supplement: S12 File — (ZIP) [file pone.0349359.s012.zip › Figure S5 list of contents.docx]

Figure S5B PTEN SCC left 10x.jpg

Figure S5B PTEN SCC left 10x.pdf

FigureS5A PTEN normal 10x.jpg

FigureS5A PTEN normal 40x.jpg

FigureS5A PTEN normal 10x.pdf

FigureS5A PTEN normal 40x.pdf

FigureS5B PTEN ADC right 10x.jpg

FigureS5B PTEN ADC right 10x.pdf

FigureS5B PTEN ADC right 40x.jpg

FigureS5B PTEN ADC right 40x.pdf

FigureS5B PTEN SCC left 40x.pdf

FigureS5B PTEN SCC left 40x.tif

SUPPORTING FIGURES FOR SUBMISSION.ppt
